# Temporal Trends in the Prevalence of Child Undernutrition in China From 2000 to 2019, With Projections of Prevalence in 2030: Cross-Sectional Analysis

**DOI:** 10.2196/58564

**Published:** 2024-10-09

**Authors:** Zeyu Zhang, Sijia Li, Zidan Zhai, Ting Qiu, Yu Zhou, Heng Zhang

**Affiliations:** 1 Wuxi School of Medicine Jiangnan University Wuxi China; 2 Department of Child Health Care Wuxi Maternity and Child Health Care Hospital Wuxi China

**Keywords:** child growth failure, undernutrition, stunting, wasting, underweight, trends, projections

## Abstract

**Background:**

Although the problem of malnutrition among children in China has greatly improved in recent years, there is a gap compared to developed countries, and there are differences between provinces. Research on long-term comprehensive trends in child growth failure (CGF) in China is needed for further improvement.

**Objective:**

The purpose of this study was to examine trends in stunting, wasting, and underweight among children younger than 5 years in China from 2000 to 2019, and predict CGF till 2030.

**Methods:**

We conducted a cross-sectional analysis using data from the local burden of disease (LBD) database. Using Joinpoint Regression Software, we examined trends in CGF among children younger than 5 years in China from 2000 to 2019, and predicted the trends of prevalence in 2030, using the Holt-Winters model with trends but without seasonal components. The assessment was performed with Stata 17 (StataCorp). Data were analyzed from October 17, 2023, to November 22, 2023.

**Results:**

In 2019, the prevalences of stunting, wasting, and underweight decreased to 12%, 3%, and 4%, respectively (decreases of 36.9%, 25.0%, and 42.9%, respectively, compared with the values in 2000). The prevalence of CGF decreased rapidly from 2000 to 2010, and the downward trend slowed down after 2010. Most provinces had stagnated processes of trends after 2017. The age group with the highest stunting prevalence was children aged 1 to 4 years, and the highest prevalence of wasting and underweight was noted in early neonatal infants. From 2000 to 2019, the prevalence of CGF declined in all age groups of children. The largest relative decrease in stunting and underweight was noted in children aged 1 to 4 years, and the largest decrease in wasting was noted in early neonatal infants. The prevalences of stunting, wasting, and underweight in China are estimated to decrease to 11.4%, 3.2%, and 4.1%, respectively, by 2030. China has nationally met the World Health Organization’s Global Nutrition Targets for 2030 for stunting but not for wasting.

**Conclusions:**

This study provides data on the prevalence and trends of CGF among children younger than 5 years and reports declines in CGF. There remain areas with slow progress in China. Most units have achieved the goal for stunting prevalence but not wasting prevalence.

## Introduction

Nutritional status during childhood has an effect on children’s short-term and long-term health and survival [1-4]. Child growth failure (CGF), expressed as stunting, wasting, and underweight in children younger than 5 years (age 0-59 months) [5], is a specific subset of undernutrition [5], which is related to high morbidity and mortality globally [6]. According to estimates from the Global Burden of Diseases, Injuries, and Risk Factors Study (GBD) 2021, a decline in CGF has contributed to a decrease in global child mortality [7]. The report “The State of Food Security and Nutrition in the World 2023” stated that at least one in every 3 children worldwide experienced CGF between 2000 and 2022 [8]. Until 2022, approximately 148.1 million children younger than 5 years worldwide experienced stunting. Although the wasting prevalence has decreased, over 6.8% (45 million) of children younger than 5 years have still experienced wasting [8]. Another study showed that the worldwide prevalences of stunting, wasting, and underweight among children were approximately 24.1%, 7.41%, and 14.7%, respectively, in 2020 [9]. Overall, child undernutrition remains a major global health concern [6].

Low-income and lower middle–income countries (LMICs) had the heaviest burden of CGF [6,8,10]. Although the prevalence of stunting and wasting has been decreasing in LMICs in recent years, progress is slow, and regional differences are significant [5,11]. In addition, only 26.7% of LMICs will achieve national-level targets for both stunting and wasting by 2025, and only 4.8% will achieve these 2 goals globally [5], remaining far from the World Health Organization (WHO) Global Nutrition Targets (GNTs) to reduce stunting by 40% and wasting to less than 5% by 2025 [5,12]. It was estimated that the COVID-19 pandemic increased the child mortality rate associated with wasting by more than 20% [8]. Therefore, overcoming this global health challenge has become even more urgent.

China is one of the low- and middle-income countries. Over the past 30 years, the problem of undernutrition among children in China has been significantly improved [13]. However, there is still a gap compared to developed countries [14]. In addition, a survey found that the Chinese official data on malnutrition are inconsistent with the results of academic surveys owing to sampling and measurement factors [15]. Although a study has mapped high spatial-resolution estimates of CGF indicators from 2000 to 2017 across 105 LMICs [5], there is no detailed introduction to the Chinese region. No long-term comprehensive trends in CGF in China have been reported, and thus, long-term CGF research of children younger than 5 years throughout China is needed. In this study, we used the local burden of disease (LBD) 2019 database to analyze the previously unknown overall and regional trends in CGF in China from 2000 to 2019 and predict CGF by 2030.

## Methods

### Overview and Data Collection

The study collected standardized disease definitions, available data, and prevalence information on children younger than 5 years in the provinces and cities of China. The study included data from the Institute for Health Metrics and Evaluation (IHME) gathered by LBD CGF collaborators, which were available in public online repositories, data that were publicly available upon request from the data provider, and data that were not publicly available due to restrictions by the data provider and which were used under license for this study. More information about each data source is available on the Global Health Data Exchange (GHDx) [5,16,17]. The analysis covered relevant metrics in national, 33 provincial-level, and 362 municipal-level administrative divisions in mainland China, Hong Kong, and Macao from 2000 to 2019. Data were analyzed from October 17, 2023, to November 22, 2023.

LBD compiled an extensive geopositioned dataset for the study, which included data from 460 household surveys and reports representing 4.6 million children. The data covered estimates of annual prevalence in 105 LMICs for each year between 2000 to 2019. The collaborators first comprehensively mapped high spatial-resolution estimates of CGF indicators from 2000 to 2017 across 105 LMICs, providing an accurate public health tool to support efficient targeting of local-level interventions to vulnerable populations. Their maps identified high-prevalence areas even within nations otherwise succeeding in reducing overall CGF prevalence. They highlighted where the highest-need populations reside to support decision makers in planning interventions adapted locally and in efficiently directing resources toward reducing CGF and its health implications [5].

### CGF Assessment

CGF, expressed as stunting, wasting, and underweight in children younger than 5 years (age 0-59 months), is a specific subset of malnutrition characterized by insufficient height or weight against age-specific growth reference standards [5]. Owing to the fact that WHO Child Growth Standards can reflect the true growth status and potential of children younger than 5 years and that the reference population is well representative, we mainly adopted them based on the comparability requirements between studies in China.

Height/length-for-age z-score (HAZ) can be used to assess the chronic malnutrition status of children. Stunting is defined as a HAZ that is more than two SDs below the median of the WHO standards for child growth [18].

Weight-for-height/length z-score (WHZ) can be used to investigate acute malnutrition in children. Wasting is defined as a WHZ that is more than two SDs below the median of the WHO standards for child growth. Severe wasting is defined as a WHZ that is more than three SDs below the median of the WHO standards for child growth [18].

Weight-for-age z-score (WAZ) can be used to determine the short-term and long-term nutritional status of children. Underweight is defined as a WAZ that is more than two SDs below the median of the WHO standards for child growth [18].

### Statistical Analysis

The prevalence of stunting, wasting, or underweight in children younger than 5 years in China was the main indicator used to reflect the health situation of children. Each prevalence is reported with 95% uncertainty interval (UI) according to the LBD algorithm. There are 4 age groups among children younger than 5 years, including early, late, and postneonatal infants, and children aged 1 to 4 years. All relevant metrics were estimated separately for the 2 sexes and various age groups (ie, 0-6 days, 7-27 days, 28 days through 11 months, 1-4 years, and 0-5 years) from 2000 to 2019. We used Excel 2017 (Microsoft Corp) to organize the data, established time series data files in CSV format between 2000 to 2019, and used Joinpoint Regression Software (Version 5.0.2; National Cancer Institute) for statistical analysis. In addition, Joinpoint regression (JPR) program was used to estimate the annual prevalence of CGF in China. Joinpoint is a statistical software for the analysis of trends using Joinpoint models, usually used to examine changes in trends. The reason we chose JPR was that JPR has certain advantages in processing long-term disease data from multiple trend segments. Moreover, JPR divides trend changes into several statistically significant trend segments through model fitting, and thus, this data processing method of segmentation is more reasonable than manual segmentation. After segmentation, researchers can clearly see the ascending segment, rapid ascending segment, gentle segment, descending segment, and rapid descending segment. Furthermore, the background and reasons for the generation of segmentation points can be analyzed, providing a basis for future medical policy formulation and resource allocation [19].

We used the normality test on the dependent variable, which followed a Poisson distribution. Therefore, we used a logarithmic linear model for analysis. The annual percentage change (APC), average annual percentage change (AAPC), and 95% CI are the main indicators for Joinpoint analysis. The number of joinpoints we set was three. APC is used to evaluate the trend of changes in various time periods, while AAPC is used to evaluate the trend of changes throughout the period, showing that the overall prevalence trend in the area increases or decreases monotonously between 2000 to 2019. If APC is <0, it indicates that the prevalence trend is decreasing year by year, while if it is >0, it indicates that the prevalence trend is increasing. All calculations were performed using Stata 17 (StataCorp). All *P* values were 2-sided, and *P*<.001 was considered statistically significant. In addition, we used the Holt-Winters model with trends but without seasonal components to predict the trends of prevalence in 2030, and the assessment was performed with Stata 17.

### Ethical Considerations

This study used data from the IHME, which were collected by LBD CGF collaborators, and this has been approved by the institutional review board of the University of Washington School of Medicine. Our research is based on the secondary analysis of data derived from the IHME. As this is a secondary analysis of existing data, no additional human participant research ethics review or informed consent was required. Study data were anonymized and deidentified to protect the privacy and confidentiality of the study participants. Our analysis complied with the Guidelines on Accurate and Transparent Health Estimate Reporting [20].

## Results

### Trend of Stunting Prevalence in 2000 and 2019 in Children Younger Than 5 Years

**Figure 1:**
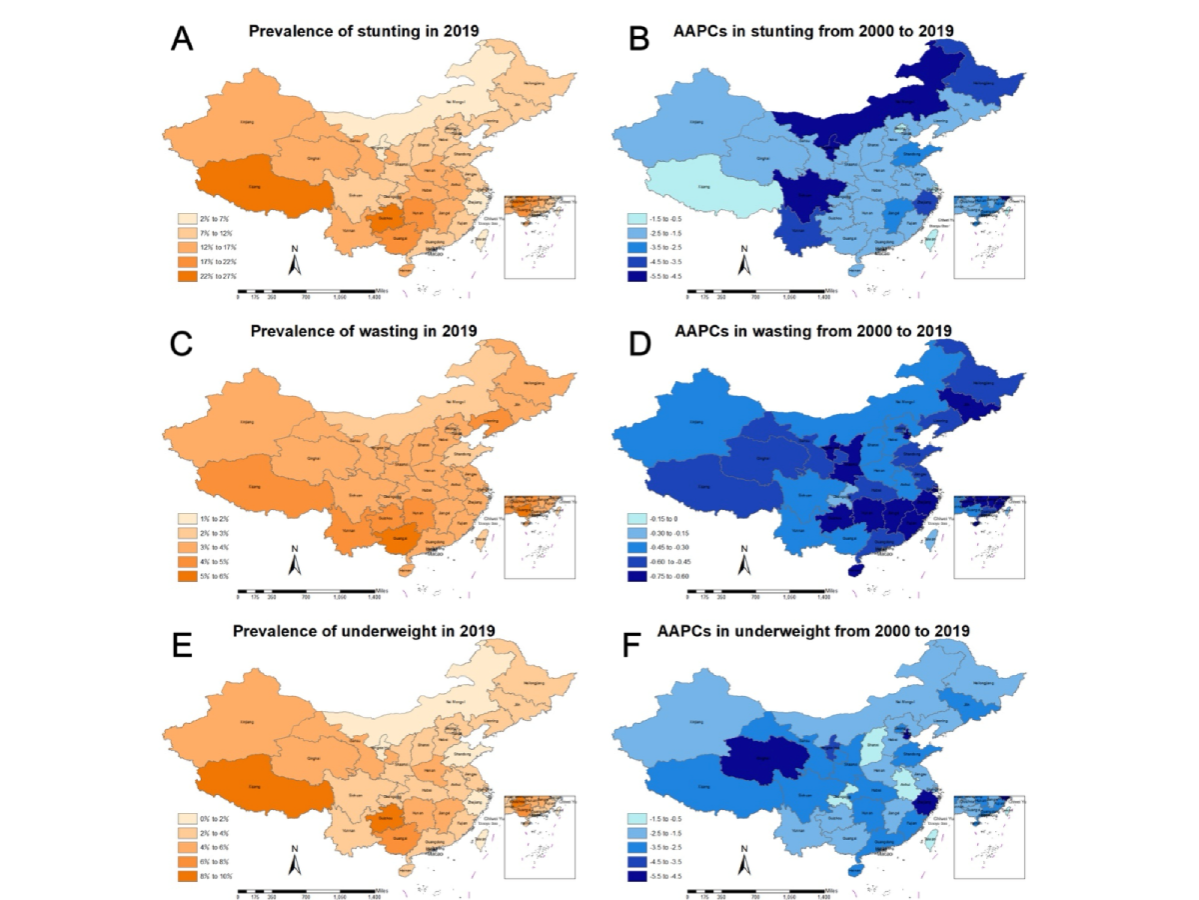
Prevalence of child growth failure in children younger than 5 years in China in 2019 and AAPCs from 2000 to 2019. (A) Prevalence of stunting in 2019; (B) AAPC in stunting from 2000 to 2019; (C) Prevalence of wasting in 2019; (D) AAPC in wasting from 2000 to 2019; (E) Prevalence of underweight in 2019; (F) AAPC in underweight from 2000 to 2019. AAPC: average annual percentage change.

Overall, the estimated stunting prevalence in children younger than 5 years across China decreased by 36.9% from 19% (95% UI 17%-21%) in 2000 to 12% (95% UI 2%-27%) in 2019 (Multimedia Appendix 1). In 2019, the prevalence was similar between boys and girls (Multimedia Appendix 2). The 3 provinces with the highest prevalence were Guizhou (26%), Xizang (22%), and Hunan (20%) (Figure 1A; Multimedia Appendix 1).

**Figure 2:**
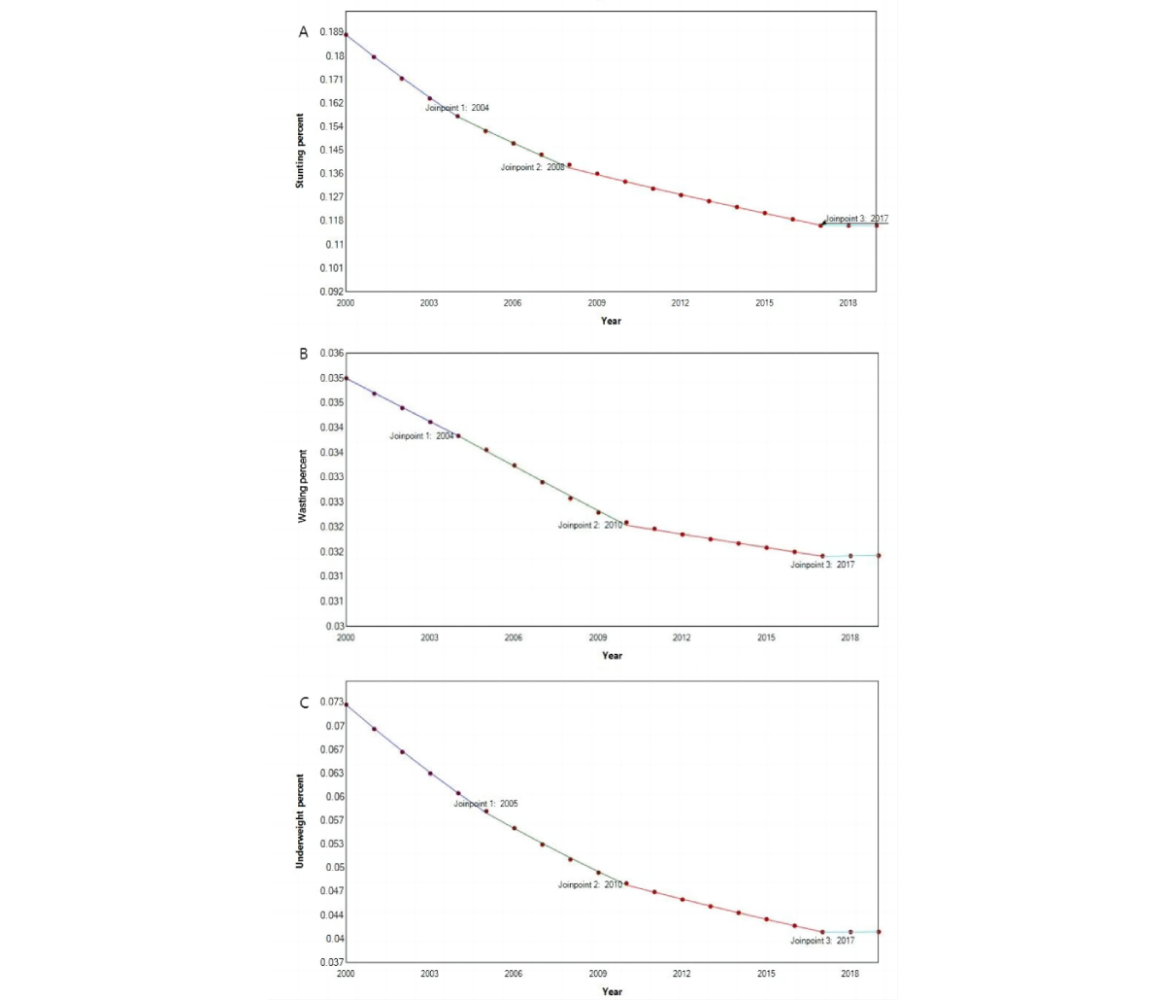
Joinpoint regression analysis of trends in child growth failure in children younger than 5 years in China from 2000 to 2019. (A) Stunting trends; (B) Wasting trends; (C) Underweight trends.

From 2000 to 2019, the stunting prevalence in China showed a significant downward trend (AAPC –2.48%, 95% CI –2.62% to –2.34%; *P*<.001; Multimedia Appendix 1). JPR analysis showed that the steepest slope occurred in 2000-2004 (APC –4.34%, 95% CI –4.50% to –4.19%; *P*<.001) in both males and females. From 2004 to 2019, the rate of decline slowed down (Tables 1-4; Figure 2A; Multimedia Appendix 3). At the provincial level, the trends of stunting prevalence varied significantly across the 33 provinces (Figure 1B; Multimedia Appendix 1), with the highest AAPC in Ningxia (AAPC –5.08%, 95% UI –5.38% to –4.79%; *P*<.001) and the lowest AAPC in Shanghai (AAPC –0.72%, 95% UI –1.08% to –0.35%; *P*<.001). There were various directions in the trends of stunting in children younger than 5 years by province (Tables 1-4). Among the provinces, 25 showed significant downward trends of stunting in children younger than 5 years at all joinpoints, while the other 8 did not, and 6 provinces (Anhui, Jilin, Ningxia, Shanxi, Yunnan, and Macao) showed a gradual increase since 2017 (Tables 1-4). The disparities between their highest- and lowest-prevalence units from 2000 to 2019 reduced in most provinces, and densely populated areas may have a relatively low stunting prevalence (Multimedia Appendix 1). At the municipal level, the stunting prevalence in 98.9% (n=358) of units showed downward trends (Multimedia Appendix 4), and the 3 with the highest AAPC were Alxa (AAPC –7.23%; *P*<.001), Baynnur (AAPC –7.11%; *P*<.001), and Wuhai (AAPC –6.98%; *P*<.001). Four units with upward trends were Xiamen (AAPC 1.54%; *P*=.66), Shenzhen (AAPC 1.07%; *P*=.66), Zhuhai (AAPC 0.16%; *P*=.95), and Central and Western Hong Kong (AAPC 0.22%; *P*=.89).

From 2000 to 2019, the stunting prevalence declined in all age groups of children (Multimedia Appendix 5), and the prevalence was similar between boys and girls (Multimedia Appendix 6). The largest decline (AAPC –1.95%; *P*<.001) occurred in children aged 1 to 4 years. The decreasing trend in prevalence was the lowest among postneonatal infants (AAPC –1.95%; *P*<.001; Multimedia Appendix 5). The trends of stunting prevalence were similar in the early and late neonatal periods (Multimedia Appendix 5).

**Table 1:** Annual percentage changes in child growth failure in children younger than 5 years in China.

Location	Stunting				Wasting				Underweight		
	Year	APC^a^ (95% CI)	*P* value	Year		APC (95% CI)	*P* value	Year		APC (95% CI)	*P* value
China	2000-2004	–4.34 (–4.50 to –4.19)	<.001	2000-2004		–0.85 (–0.88 to –0.82)	<.001	2000-2005		–4.52 (–4.58 to –4.46)	<.001
China	2004-2008	–3.18 (–3.51 to –2.85)	<.001	2004-2010		–0.92 (–0.94 to –0.90)	<.001	2005-2010		–3.74 (–3.84 to –3.64)	<.001
China	2008-2017	–1.85 (–1.87 to –1.84)	<.001	2010-2017		–0.29 (–0.30 to –0.27)	<.001	2010-2017		–2.08 (–2.15 to –2.01)	<.001
China	2017-2019	–0.08 (–1.48 to 1.34)	.91	2017-2019		0.03 (–0.16 to 0.21)	.75	2017-2019		0.03 (–0.75 to 0.82)	.93

^a^APC: annual percentage change.

**Table 2:** Annual percentage changes in child growth failure in children younger than 5 years in Anhui, Beijing, Chongqing, Fujian, Gansu, Guangdong, Guangxi, Guizhou, Hainan, Hebei, and Heilongjiang provinces in China.

Location	Stunting				Wasting				Underweight		
	Year	APC^a^ (95% CI)	*P* value	Year		APC (95% CI)	*P* value	Year		APC (95% CI)	*P* value
Anhui	2000-2004	–3.43 (–3.63 to –3.23)	<.001	2000-2002		–0.76 (–0.80 to –0.72)	<.001	2000-2004		–3.37 (–3.57 to –3.17)	<.001
Anhui	2004-2009	–1.07 (–1.15 to –0.99)	<.001	2002-2010		–0.58 (–0.58 to –0.57)	<.001	2004-2010		–1.93 (–2.09 to –1.76)	<.001
Anhui	2009-2015	–3.16 (–3.27 to –3.05)	<.001	2010-2017		–0.51 (–0.51 to –0.50)	<.001	2010-2017		–3.35 (–3.48 to –3.21)	<.001
Anhui	2015-2019	0.44 (–1.08 to 1.98)	.54	2017-2019		0.00 (–0.06 to 0.06)	.89	2017-2019		0.41 (–0.87 to 1.70)	.49
Beijing	2000-2004	–4.74 (–4.91 to –4.58)	<.001	2000-2002		–0.60 (–0.75 to –0.45)	<.001	2000-2004		–4.25 (–4.42 to –4.08)	<.001
Beijing	2004-2011	0.00 (–0.50 to 0.49)	.99	2002-2009		–0.55 (–0.58 to –0.52)	<.001	2004-2007		–0.92 (–1.49 to –0.35)	.005
Beijing	2011-2017	0.94 (0.22 to 1.66)	.02	2009-2015		–0.01 (–0.05 to 0.03)	.69	2007-2014		–0.35 (–0.46 to –0.23)	<.001
Beijing	2017-2019	–0.21 (–0.27 to –0.14)	<.001	2015-2019		–0.10 (–0.16 to –0.05)	.003	2014-2019		–0.46 (–0.65 to –0.27)	<.001
Chongqing	2000-2004	–3.86 (–4.01 to –3.71)	<.001	2000-2005		–0.84 (–0.89 to –0.79)	<.001	2000-2006		–3.57 (–3.66 to –3.48)	<.001
Chongqing	2004-2007	–2.82 (–2.92 to –2.73)	<.001	2005-2009		–1.09 (–1.24 to –0.94)	<.001	2006-2010		–3.78 (–4.12 to –3.44)	<.001
Chongqing	2007-2010	–2.18 (–2.20 to –2.16)	<.001	2009-2012		–0.43 (–0.76 to –0.10)	.02	2010-2017		–2.56 (–2.70 to –2.42)	<.001
Chongqing	2010-2019	–0.09 (–0.83 to 0.66)	.79	2012-2019		–0.21 (–0.26 to –0.16)	<.001	2017-2019		0.06 (–1.07 to 1.21)	.91
Fujian	2000-2003	–6.00 (–6.90 to –5.09)	<.001	2000-2009		–0.90 (–0.91 to –0.89)	<.001	2000-2004		–5.17 (–5.51 to –4.83)	<.001
Fujian	2003-2008	–4.01 (–5.00 to –3.01)	<.001	2009-2012		–0.40 (–0.55 to –0.26)	<.001	2004-2008		–3.00 (–3.58 to –2.42)	<.001
Fujian	2008-2017	–1.31 (–1.35 to –1.27)	<.001	2012-2017		–0.27 (–0.31 to –0.23)	<.001	2008-2017		–1.73 (–1.79 to –1.67)	<.001
Fujian	2017-2019	–0.44 (–2.42 to 1.59)	.63	2017-2019		–0.08 (–0.28 to 0.12)	.40	2017-2019		–0.14 (–1.86 to 1.62)	.86
Gansu	2000-2002	–3.69 (–3.97 to –3.41)	<.001	2000-2005		–0.69 (–0.72 to –0.66)	<.001	2000-2003		–3.94 (–4.07 to –3.82)	<.001
Gansu	2002-2005	–3.08 (–3.42 to –2.74)	<.001	2005-2010		–0.84 (–0.89 to –0.80)	<.001	2003-2010		–3.47 (–3.51 to –3.42)	<.001
Gansu	2005-2017	–2.08 (–2.10 to –2.07)	<.001	2010-2017		–0.27 (–0.30 to –0.25)	<.001	2010-2017		–2.53 (–2.58 to –2.47)	<.001
Gansu	2017-2019	–0.04 (–0.74 to 0.67)	.91	2017-2019		0.00 (–0.21 to 0.20)	.96	2017-2019		0.12 (–0.25 to 0.50)	.48
Guangdong	2000-2002	–4.88 (–4.98 to –4.77)	<.001	2000-2005		–0.57 (–0.60 to –0.54)	<.001	2000-2004		–4.75 (–4.91 to –4.59)	<.001
Guangdong	2002-2005	–2.29 (–2.62 to –1.96)	<.001	2005-2009		–0.94 (–1.02 to –0.87)	<.001	2004-2009		–3.12 (–3.31 to –2.92)	<.001
Guangdong	2005-2017	–1.27 (–1.28 to –1.25)	<.001	2009-2012		–0.13 (–0.30 to 0.04)	.12	2009-2017		–1.54 (–1.61 to –1.48)	<.001
Guangdong	2017-2019	–0.11 (–0.88 to 0.67)	.76	2012-2019		–0.03 (–0.06 to –0.01)	.02	2017-2019		0.08 (–0.93 to 1.11)	.86
Guangxi	2000-2004	–4.27 (–4.41 to –4.13)	<.001	2000-2005		–0.91 (–0.95 to –0.86)	<.001	2000-2004		–3.63 (–3.76 to –3.50)	<.001
Guangxi	2004-2007	–2.65 (–3.07 to –2.22)	<.001	2005-2009		–1.25 (–1.38 to –1.13)	<.001	2004-2007		–2.40 (–2.88 to –1.91)	<.001
Guangxi	2007-2017	–1.72 (–1.75 to –1.69)	<.001	2009-2012		–0.46 (–0.76 to –0.16)	.007	2007-2017		–1.53 (–1.58 to –1.48)	<.001
Guangxi	2017-2019	–0.48 (–1.64 to 0.69)	.38	2012-2019		–0.19 (–0.24 to –0.15)	<.001	2017-2019		–0.42 (–1.56 to 0.74)	.43
Guizhou	2000-2004	–1.97 (–2.01 to –1.94)	<.001	2000-2005		–0.76 (–0.78 to –0.74)	<.001	2000-2005		–2.61 (–2.66 to –2.56)	<.001
Guizhou	2004-2007	–2.41 (–2.44 to –2.37)	<.001	2005-2010		–1.03 (–1.06 to –1.01)	<.001	2005-2010		–4.25 (–4.34 to –4.16)	<.001
Guizhou	2007-2017	–2.56 (–2.97 to –2.15)	<.001	2010-2017		–0.48 (–0.50 to –0.46)	<.001	2010-2017		–3.44 (–3.53 to –3.36)	<.001
Guizhou	2017-2019	–0.30 (–1.14 to 0.54)	.43	2017-2019		–0.03 (–0.19 to 0.13)	.64	2017-2019		–0.17 (–0.94 to 0.60)	.63
Hainan	2000-2005	–3.43 (–3.62 to –3.24)	<.001	2000-2005		–0.27 (–0.30 to –0.23)	<.001	2000-2005		–3.15 (–3.26 to –3.05)	<.001
Hainan	2005-2014	–2.57 (–2.66 to –2.48)	<.001	2005-2009		–1.20 (–1.30 to –1.10)	<.001	2005-2010		–3.70 (–3.88 to –3.51)	<.001
Hainan	2014-2017	–1.54 (–1.57 to –1.50)	<.001	2009-2012		–0.36 (–0.60 to –0.12)	.008	2010-2017		–1.78 (–1.90 to –1.66)	<.001
Hainan	2017-2019	–0.28 (–1.19 to 0.63)	.50	2012-2019		–0.12 (–0.16 to –0.08)	<.001	2017-2019		–0.23 (–1.46 to 1.01)	.68
Hebei	2000-2003	–4.62 (–4.77 to –4.46)	<.001	2000-2005		–0.84 (–0.88 to –0.81)	<.001	2000-2004		–4.21 (–4.34 to –4.07)	<.001
Hebei	2003-2009	–2.43 (–2.68 to –2.19)	<.001	2005-2009		–1.11 (–1.21 to –1.00)	<.001	2004-2009		–2.85 (–3.01 to –2.69)	<.001
Hebei	2009-2017	–1.12 (–1.13 to –1.10)	<.001	2009-2012		–0.38 (–0.59 to –0.17)	.003	2009-2017		–1.44 (–1.51 to –1.37)	<.001
Hebei	2017-2019	–0.22 (–0.92 to 0.49)	.51	2012-2019		–0.14 (–0.17 to –0.10)	<.001	2017-2019		0.02 (–0.94 to 0.99)	.96
Heilongjiang	2000-2003	–7.59 (–7.77 to –7.42)	<.001	2000-2003		–0.99 (–1.13 to –0.86)	<.001	2000-2004		–4.88 (–5.06 to –4.69)	<.001
Heilongjiang	2003-2006	–5.20 (–5.74 to –4.65)	<.001	2003-2009		–0.78 (–0.84 to –0.71)	<.001	2004-2008		–3.15 (–3.50 to –2.80)	<.001
Heilongjiang	2006-2017	–2.49 (–2.76 to –2.23)	<.001	2009-2013		–0.01 (–0.16 to 0.14)	.90	2008-2012		–1.55 (–1.95 to –1.14)	<.001
Heilongjiang	2017-2019	–0.92 (–1.25 to –0.59)	<.001	2013-2019		0.19 (0.13 to 0.25)	<.001	2012-2019		–0.57 (–0.71 to –0.42)	<.001

^a^APC: annual percentage change.

**Table 3:** Annual percentage changes in child growth failure in children younger than 5 years in Henan, Hubei, Hunan, Jiangsu, Jiangxi, Jilin, Liaoning, Inner Mongolia, Ningxia, Qinghai, and Shaanxi provinces in China.

Location	Stunting				Wasting				Underweight		
	Year	APC^a^ (95% CI)	*P* value	Year		APC (95% CI)	*P* value	Year		APC (95% CI)	*P* value
Henan	2000-2005	–2.93 (–2.98 to –2.89)	<.001	2000-2007		–0.99 (–1.01 to –0.98)	<.001	2000-2005		–3.21 (–3.26 to –3.16)	<.001
Henan	2005-2009	–2.25 (–2.83 to –1.67)	<.001	2007-2010		–0.70 (–0.87 to –0.53)	<.001	2005-2010		–3.95 (–4.02 to –3.87)	<.001
Henan	2009-2014	–1.50 (–1.56 to –1.44)	<.001	2010-2017		–0.28 (–0.30 to –0.25)	<.001	2010-2017		–1.89 (–1.94 to –1.85)	<.001
Henan	2014-2019	–0.18 (–1.17 to 0.82)	.70	2017-2019		–0.04 (–0.30 to 0.21)	.71	2017-2019		–0.10 (–0.46 to 0.28)	.58
Hubei	2000-2007	–4.72 (–5.12 to –4.33)	<.001	2000-2009		–1.04 (–1.04 to –1.03)	<.001	2000-2004		–5.90 (–6.02 to –5.78)	<.001
Hubei	2007-2010	–1.52 (–2.71 to –0.33)	.02	2009-2012		–0.47 (–0.58 to –0.35)	<.001	2004-2007		–3.50 (–3.91 to –3.09)	<.001
Hubei	2010-2017	–0.56 (–2.12 to 1.03)	.44	2012-2017		–0.37 (–0.40 to –0.35)	<.001	2007-2017		–2.08 (–2.12 to –2.03)	<.001
Hubei	2017-2019	–1.40 (–1.56 to –1.24)	<.001	2017-2019		–0.07 (–0.23 to 0.08)	.31	2017-2019		0.01 (–0.70 to 0.73)	.97
Hunan	2000-2004	–2.88 (–3.00 to –2.77)	<.001	2000-2005		–0.90 (–0.91 to –0.88)	<.001	2000-2004		–3.29 (–3.36 to –3.22)	<.001
Hunan	2004-2007	–2.01 (–2.27 to –1.74)	<.001	2005-2010		–0.68 (–0.70 to –0.66)	<.001	2004-2009		–2.88 (–2.97 to –2.78)	<.001
Hunan	2007-2010	–1.23 (–1.25 to –1.20)	<.001	2010-2017		–0.35 (–0.36 to –0.34)	<.001	2009-2017		–1.79 (–1.83 to –1.75)	<.001
Hunan	2010-2019	–0.24 (–1.22 to 0.75)	.60	2017-2019		–0.03 (–0.17 to 0.11)	.65	2017-2019		–0.11 (–0.64 to 0.43)	.66
Jiangsu	2000-2004	–3.96 (–4.01 to –3.90)	<.001	2000-2009		–0.98 (–0.99 to –0.97)	<.001	2000-2005		–3.78 (–3.85 to –3.71)	<.001
Jiangsu	2004-2008	–2.23 (–2.35 to –2.12)	<.001	2009-2012		–0.51 (–0.61 to –0.40)	<.001	2005-2010		–3.09 (–3.21 to –2.96)	<.001
Jiangsu	2008-2017	–1.31 (–1.37 to –1.26)	<.001	2012-2017		–0.35 (–0.37 to –0.32)	<.001	2010-2017		–1.46 (–1.54 to –1.39)	<.001
Jiangsu	2017-2019	–0.02 (–1.02 to 0.99)	.97	2017-2019		–0.02 (–0.15 to 0.11)	.77	2017-2019		–0.04 (–0.77 to 0.70)	.91
Jiangxi	2000-2005	–4.15 (–4.30 to –4.01)	<.001	2000-2007		–1.22 (–1.24 to –1.21)	<.001	2000-2007		–4.41 (–4.49 to –4.34)	<.001
Jiangxi	2005-2010	–3.08 (–3.41 to –2.74)	<.001	2007-2010		–0.97 (–1.06 to –0.88)	<.001	2007-2010		–3.74 (–4.30 to –3.17)	<.001
Jiangxi	2010-2017	–2.04 (–2.07 to –2.02)	<.001	2010-2017		–0.30 (–0.32 to –0.29)	<.001	2010-2017		–2.40 (–2.50 to –2.29)	<.001
Jiangxi	2017-2019	–0.14 (–1.24 to 0.98)	.79	2017-2019		0.01 (–0.14 to 0.15)	.93	2017-2019		–0.02 (–1.08 to 1.05)	.97
Jilin	2000-2004	–3.74 (–3.93 to –3.55)	<.001	2000-2006		–0.85 (–0.87 to –0.84)	<.001	2000-2003		–3.80 (–4.00 to –3.60)	<.001
Jilin	2004-2008	–1.53 (–1.67 to –1.38)	<.001	2006-2009		–1.00 (–1.11 to –0.89)	<.001	2003-2009		–2.85 (–2.97 to –2.74)	<.001
Jilin	2008-2017	–1.76 (–1.85 to –1.67)	<.001	2009-2014		–0.43 (–0.47 to –0.40)	<.001	2009-2016		–2.20 (–2.31 to –2.10)	<.001
Jilin	2017-2019	0.29 (–0.83 to 1.43)	.57	2014-2019		–0.02 (–0.05 to 0.01)	.23	2016-2019		–0.29 (–0.67 to 0.10)	.12
Liaoning	2000-2004	–5.11 (–5.31 to –4.92)	<.001	2000-2004		–0.98 (–1.01 to –0.94)	<.001	2000-2005		–4.89 (–4.98 to –4.80)	<.001
Liaoning	2004-2010	–2.74 (–3.31 to –2.16)	<.001	2004-2008		–0.69 (–0.75 to –0.63)	<.001	2005-2009		–3.01 (–3.32 to –2.71)	<.001
Liaoning	2010-2017	–1.25 (–1.57 to –0.93)	<.001	2008-2011		–0.31 (–0.44 to –0.18)	<.001	2009-2014		–1.40 (–1.60 to –1.20)	<.001
Liaoning	2017-2019	–0.57 (–0.67 to –0.46)	<.001	2011-2019		–0.03 (–0.04 to –0.01)	.006	2014-2019		–0.49 (–0.67 to –0.30)	<.001
Inner Mongolia	2000-2004	–7.74 (–7.82 to –7.67)	<.001	2000-2005		–0.92 (–0.94 to –0.90)	<.001	2000-2004		–8.00 (–8.25 to –7.75)	<.001
Inner Mongolia	2004-2007	–5.10 (–6.02 to –4.17)	<.001	2005-2009		–1.38 (–1.44 to –1.32)	<.001	2004-2009		–6.28 (–6.60 to –5.96)	<.001
Inner Mongolia	2007-2011	–2.53 (–2.94 to –2.12)	<.001	2009-2013		–0.47 (–0.52 to –0.42)	<.001	2009-2014		–2.72 (–3.06 to –2.38)	<.001
Inner Mongolia	2011-2019	–0.69 (–1.85 to 0.49)	.22	2013-2019		–0.05 (–0.07 to –0.02)	.004	2014-2019		–1.02 (–1.38 to –0.67)	<.001
Ningxia	2000-2008	–8.16 (–8.28 to –8.03)	<.001	2000-2005		–0.99 (–1.01 to –0.97)	<.001	2000-2007		–8.68 (–8.80 to –8.55)	<.001
Ningxia	2008-2011	–4.89 (–5.99 to –3.77)	<.001	2005-2009		–1.26 (–1.32 to –1.20)	<.001	2007-2010		–6.64 (–7.69 to –5.58)	<.001
Ningxia	2011-2016	–2.64 (–2.88 to –2.40)	<.001	2009-2012		–0.22 (–0.33 to –0.12)	.001	2010-2017		–2.24 (–2.44 to –2.03)	<.001
Ningxia	2016-2019	0.04 (–2.69 to 2.85)	.97	2012-2019		–0.05 (–0.07 to –0.04)	<.001	2017-2019		0.28 (–1.28 to 1.87)	.70
Qinghai	2000-2008	–3.19 (–3.26 to –3.11)	<.001	2000-2005		–0.85 (–0.90 to –0.80)	<.001	2000-2009		–3.67 (–3.69 to –3.65)	<.001
Qinghai	2008-2011	–2.65 (–2.80 to –2.51)	<.001	2005-2009		–1.15 (–1.29 to –1.01)	<.001	2009-2012		–2.96 (–3.22 to –2.69)	<.001
Qinghai	2011-2017	–2.07 (–2.08 to –2.05)	<.001	2009-2012		–0.46 (–0.72 to –0.20)	.003	2012-2017		–2.53 (–2.62 to –2.43)	<.001
Qinghai	2017-2019	–0.13 (–0.50 to 0.24)	.45	2012-2019		–0.16 (–0.20 to –0.12)	<.001	2017-2019		–0.02 (–0.35 to 0.31)	.89
Shaanxi	2000-2003	–4.35 (–4.50 to –4.20)	<.001	2000-2005		–0.69 (–0.72 to –0.66)	<.001	2000-2004		–4.37 (–4.51 to –4.24)	<.001
Shaanxi	2003-2006	–2.91 (–3.18 to –2.64)	<.001	2005-2010		–0.95 (–1.00 to –0.90)	<.001	2004-2010		–3.75 (–3.85 to –3.65)	<.001
Shaanxi	2006-2017	–1.67 (–1.70 to –1.64)	<.001	2010-2017		–0.27 (–0.29 to –0.24)	<.001	2010-2017		–2.00 (–2.08 to –1.92)	<.001
Shaanxi	2017-2019	–0.04 (–1.02 to 0.95)	.93	2017-2019		0.04 (–0.18 to 0.27)	.66	2017-2019		0.10 (–0.54 to 0.74)	.74

^a^APC: annual percentage change.

**Table 4:** Annual percentage changes in child growth failure in children younger than 5 years in Shandong, Shanghai Shanxi, Sichuan, Tianjin, Xinjiang, Xizang, Yunnan, Zhejiang, Hong Kong, and Macao provinces in China.

Location	Stunting				Wasting				Underweight		
	Year	APC^a^ (95% CI)	*P* value	Year		APC (95% CI)	*P* value	Year		APC (95% CI)	*P* value
Shandong	2000-2004	–7.83 (–9.11 to –6.53)	<.001	2000-2004		–1.54 (–1.60 to –1.48)	<.001	2000-2004		–6.08 (–6.24 to –5.92)	<.001
Shandong	2004-2008	–5.33 (–6.96 to –3.67)	<.001	2004-2007		–0.96 (–1.15 to –0.77)	<.001	2004-2007		–2.70 (–3.33 to –2.08)	<.001
Shandong	2008-2017	–2.13 (–2.19 to –2.06)	<.001	2007-2010		–0.56 (–0.76 to –0.35)	<.001	2007-2017		–1.29 (–1.35 to –1.22)	<.001
Shandong	2017-2019	–0.30 (–3.65 to 3.18)	.85	2010-2019		–0.11 (–0.13 to –0.09)	<.001	2017-2019		–0.07 (–1.20 to 1.08)	.90
Shanghai	2000-2002	–5.58 (–7.20 to –3.93)	<.001	2000-2005		–0.57 (–0.60 to –0.55)	<.001	2000-2004		–4.51 (–4.79 to –4.22)	<.001
Shanghai	2002-2005	–2.99 (–5.11 to –0.82)	.01	2005-2009		–0.67 (–0.74 to –0.59)	<.001	2004-2007		–0.08 (–1.16 to 1.01)	.87
Shanghai	2005-2017	1.52 (1.18 to 1.86)	<.001	2009-2015		–0.02 (–0.06 to 0.01)	.16	2007-2015		0.17 (0.01 to 0.32)	.04
Shanghai	2017-2019	–0.27 (–0.53 to –0.01)	.045	2015-2019		–0.11 (–0.16 to –0.07)	<.001	2015-2019		–1.07 (–1.50 to –0.63)	<.001
Shanxi	2000-2002	–4.10 (–4.31 to –3.89)	<.001	2000-2005		–0.66 (–0.69 to –0.63)	<.001	2000-2003		–4.23 (–4.46 to –4.00)	<.001
Shanxi	2002-2005	–2.77 (–3.32 to –2.21)	<.001	2005-2009		–1.00 (–1.09 to –0.91)	<.001	2003-2010		–3.50 (–3.60 to –3.41)	<.001
Shanxi	2005-2011	–1.72 (–1.76 to –1.68)	<.001	2009-2012		–0.28 (–0.44 to –0.11)	.004	2010-2017		–1.90 (–2.00 to –1.81)	<.001
Shanxi	2011-2019	0.25 (–1.17 to 1.69)	.70	2012-2019		–0.03 (–0.06 to 0.00)	.02	2017-2019		0.34 (–0.51 to 1.19)	.39
Sichuan	2000-2004	–6.16 (–7.23 to –5.07)	<.001	2000-2009		–1.01 (–1.03 to –1.00)	<.001	2000-2005		–7.35 (–7.48 to –7.21)	<.001
Sichuan	2004-2007	–6.92 (–7.08 to –6.75)	<.001	2009-2012		–0.40 (–0.62 to –0.17)	.004	2005-2010		–8.53 (–8.74 to –8.32)	<.001
Sichuan	2007-2017	–3.87 (–4.50 to –3.25)	<.001	2012-2017		–0.21 (–0.27 to –0.15)	<.001	2010-2016		–3.27 (–3.49 to –3.05)	<.001
Sichuan	2017-2019	–1.41 (–1.96 to –0.85)	<.001	2017-2019		–0.02 (–0.31 to 0.28)	.91	2016-2019		–0.66 (–1.26 to –0.06)	.04
Tianjin	2000-2002	–5.66 (–5.83 to –5.49)	<.001	2000-2006		–0.71 (–0.74 to –0.69)	<.001	2000-2004		–5.31 (–5.45 to –5.18)	<.001
Tianjin	2002-2010	–1.19 (–1.68 to –0.70)	<.001	2006-2009		–0.77 (–1.01 to –0.54)	<.001	2004-2008		–2.31 (–2.55 to –2.08)	<.001
Tianjin	2010-2015	–0.23 (–0.50 to 0.03)	.08	2009-2012		–0.27 (–0.48 to –0.06)	.02	2008-2017		–0.90 (–0.96 to –0.84)	<.001
Tianjin	2015-2019	–0.59 (–0.67 to –0.50)	<.001	2012-2019		–0.11 (–0.14 to –0.07)	<.001	2017-2019		–0.06 (–0.89 to 0.78)	.88
Xinjiang	2000-2004	–3.54 (–3.70 to –3.38)	<.001	2000-2005		–0.68 (–0.73 to –0.63)	<.001	2000-2010		–3.74 (–3.78 to –3.71)	<.001
Xinjiang	2004-2007	–2.44 (–2.65 to –2.23)	<.001	2005-2009		–1.15 (–1.28 to –1.01)	<.001	2010-2014		–1.58 (–1.83 to –1.32)	<.001
Xinjiang	2007-2011	–1.43 (–1.51 to –1.34)	<.001	2009-2012		–0.31 (–0.56 to –0.07)	.02	2014-2017		–2.24 (–2.76 to –1.71)	<.001
Xinjiang	2011-2019	–0.52 (–1.52 to 0.49)	.27	2012-2019		–0.15 (–0.18 to –0.11)	<.001	2017-2019		–0.09 (–0.71 to 0.53)	.75
Xizang	2000-2004	–2.11 (–2.23 to –1.99)	<.001	2000-2005		–0.36 (–0.41 to –0.32)	<.001	2000-2005		–2.60 (–2.67 to –2.53)	<.001
Xizang	2004-2009	–0.72 (–0.77 to –0.67)	<.001	2005-2009		–0.89 (–0.99 to –0.80)	<.001	2005-2014		–1.67 (–1.72 to –1.63)	<.001
Xizang	2009-2017	–1.55 (–1.82 to –1.28)	<.001	2009-2012		–0.32 (–0.51 to –0.12)	.005	2014-2017		–2.10 (–2.60 to –1.61)	<.001
Xizang	2017-2019	–0.13 (–0.87 to 0.61)	.70	2012-2019		–0.12 (–0.15 to –0.09)	<.001	2017-2019		–0.13 (–0.70 to 0.45)	.63
Yunnan	2000-2004	–5.06 (–5.19 to –4.93)	<.001	2000-2005		–0.81 (–0.83 to –0.79)	<.001	2000-2004		–6.62 (–6.79 to –6.45)	<.001
Yunnan	2004-2013	–6.02 (–6.11 to –5.92)	<.001	2005-2010		–1.12 (–1.15 to –1.09)	<.001	2004-2010		–7.62 (–7.75 to –7.49)	<.001
Yunnan	2013-2017	–3.26 (–3.34 to –3.19)	<.001	2010-2017		–0.34 (–0.36 to –0.32)	<.001	2010-2017		–3.37 (–3.49 to –3.25)	<.001
Yunnan	2017-2019	0.17 (–0.82 to 1.17)	.71	2017-2019		–0.03 (–0.19 to 0.14)	.74	2017-2019		0.23 (–0.79 to 1.26)	.63
Zhejiang	2000-2004	–7.83 (–8.16 to –7.49)	<.001	2000-2005		–0.86 (–0.89 to –0.84)	<.001	2000-2004		–7.90 (–8.25 to –7.56)	<.001
Zhejiang	2004-2010	–5.71 (–6.19 to –5.22)	<.001	2005-2009		–0.91 (–0.97 to –0.84)	<.001	2004-2009		–5.98 (–6.35 to –5.61)	<.001
Zhejiang	2010-2017	–2.70 (–3.02 to –2.38)	<.001	2009-2012		–0.05 (–0.18 to 0.08)	.37	2009-2013		–2.29 (–2.89 to –1.68)	<.001
Zhejiang	2017-2019	–1.14 (–1.55 to –0.73)	<.001	2012-2019		0.02 (0.00 to 0.04)	.04	2013-2019		–1.03 (–1.31 to –0.75)	<.001
Hong Kong	2000-2004	–0.22 (–0.26 to –0.18)	<.001	2000-2007		1.91 (1.75 to 2.07)	<.001	2000-2005		–0.79 (–0.84 to –0.74)	<.001
Hong Kong	2004-2009	–1.09 (–1.16 to –1.02)	<.001	2007-2011		0.50 (–0.21 to 1.22)	.14	2005-2013		–1.21 (–1.24 to –1.18)	<.001
Hong Kong	2009-2014	–2.27 (–2.31 to –2.23)	<.001	2011-2017		–2.47 (–2.70 to –2.25)	<.001	2013-2017		–1.04 (–1.15 to –0.93)	<.001
Hong Kong	2014-2019	–0.17 (–0.73 to 0.39)	.51	2017-2019		–0.61 (–2.62 to 1.45)	.52	2017-2019		0.03 (–0.33 to 0.39)	.86
Macao	2000-2006	–3.14 (–3.24 to –3.05)	<.001	2000-2005		0.70 (0.63 to 0.78)	<.001	2000-2005		–2.11 (–2.15 to –2.06)	<.001
Macao	2006-2011	–0.11 (–0.15 to –0.08)	<.001	2005-2010		–0.22 (–0.31 to –0.13)	<.001	2005-2010		–0.98 (–1.04 to –0.93)	<.001
Macao	2011-2017	–0.92 (–0.96 to –0.88)	<.001	2010-2017		–0.85 (–0.90 to –0.79)	<.001	2010-2016		–1.39 (–1.43 to –1.34)	<.001
Macao	2017-2019	0.13 (–0.49 to 0.76)	.65	2017-2019		0.03 (–0.55 to 0.61)	.91	2016-2019		–0.26 (–0.41 to –0.11)	.003

^a^APC: annual percentage change.

### Trend of Wasting Prevalence in 2000 and 2019 in Children Younger Than 5 Years

Childhood wasting was less widespread than stunting, affecting 4% (3%-5%) of children younger than 5 years in China in 2000 and 3% (1%-8%) by 2019 (Multimedia Appendix 1). In 2019, the prevalence was similar between boys and girls (Multimedia Appendix 2). Among the 33 provinces, the wasting prevalence was the highest in Guangxi (5%) (Figure 1C; Multimedia Appendix 1).

From 2000 to 2019, there was a slow downward trend (AAPC –0.57%, 95% CI –0.59% to –0.55%; *P*<.001) in the wasting prevalence, which was smoother compared to the stunting prevalence in China (Multimedia Appendix 1). The analysis showed that the greatest increase occurred in 2004-2010 (APC –0.92%, 95% CI –0.94% to –0.90%; *P*<.001), and from 2010 to 2019, there was a steady trend in China as a whole and in most provinces (Tables 1-4; Figure 2B; Multimedia Appendix 3). At the provincial level, the trends of wasting prevalence varied significantly across the 33 provinces (Figure 1D; Multimedia Appendix 1), with the highest AAPC in Jilin (AAPC –0.72%, 95% UI –0.74% to –0.70%; *P*<.001) and the lowest AAPC in Macao (AAPC –0.05%, 95% UI –0.29% to 0.19%; *P*=.67). There were various directions in the trends of wasting in children younger than 5 years by province (Tables 1-4). Among the provinces, 25 showed significant downward trends of wasting in children younger than 5 years at all joinpoints, while the other 8 did not, and 3 provinces (Jiangxi, Shanxi, and Macao) showed a gradual increase since 2017, while 2 (Anhui and Gansu) remained stable since 2017 (Tables 1-4). There was a noticeable increase in the wasting prevalence from 2000 to 2004 in the Hong Kong province (APC 1.91%, 95% UI 1.75%-2.07%; *P*<.001), with an upward trend in the 11 years spanning from 2000 to 2011 (Table 4). At the municipal level, the wasting prevalence in 90.0% (n=326) of units showed downward trends (Multimedia Appendix 4), and the 3 with the highest AAPC were Chengde (AAPC –2.18%; *P*=.01), Zhangjiakou (AAPC –2.11%; *P*=.06), and Baicheng (AAPC –1.89%; *P*<.001). Three units with the most significant upward trends were Jixi (AAPC 0.95%; *P*=.07), Yanbian Korean Autonomous Prefecture (AAPC 0.90%; *P*=.02), and Shuangyashan (AAPC 0.86%; *P*=.03), while those with stable trends were Jilin (AAPC 0.00%; *P*=.99) and Wong Tai Sin of Hong Kong province (AAPC 0.00%; *P*=.99).

The prevalence was the highest among early neonatal infants in both 2000 and 2019 (16% and 11%, respectively) (Multimedia Appendix 5). From 2000 to 2019, the wasting prevalence declined in all age groups of children (Multimedia Appendix 5), and the prevalence was similar between boys and girls (Multimedia Appendix 6). The largest decline (AAPC –1.95%; *P*<.001) occurred in the early neonatal period. The trends of wasting prevalence were similar in the late and postneonatal periods, as well as in children aged 1 to 4 years and those aged younger than 5 years (Multimedia Appendix 5).

### Trend of Underweight Prevalence in 2000 and 2019 in Children Younger Than 5 Years

During the same period, the estimated underweight prevalence in children younger than 5 years decreased by 42.9% from 7% (6%-9%) in 2000 to 4% (2%-9%) in 2019 (Multimedia Appendix 1). The 3 provinces with the highest prevalence were Xizang (9%), Guizhou (9%), and Guangxi (8%) (Figure 1E; Multimedia Appendix 1). The prevalence was similar between boys and girls in 2019 (Multimedia Appendix 2).

From 2000 to 2019, the underweight prevalence in China demonstrated a significant downward trend (AAPC –2.95%, 95% CI –3.02% to –2.87%; *P*<.001) (Multimedia Appendix 1). JPR analysis showed that the most substantial changes occurred in 2000-2005 (APC –4.52%, 95% CI –4.58% to –4.46%; *P*<.001) in China. The trend decreased steadily from 2005 to 2017 and remained stable after 2017 (Tables 1-4; Figure 2C; Multimedia Appendix 3). At the provincial level, the trends of underweight prevalence varied significantly across the 33 provinces (Figure 1F; Multimedia Appendix 1), with the highest AAPC in Sichuan (AAPC –5.36%, 95% UI –5.47% to –5.25%; *P*<.001) and the lowest AAPC in Hong Kong (AAPC –0.93%, 95% UI –0.98% to 0.89%; *P*<.001). There were various directions in the trends of underweight in children younger than 5 years by province (Tables 1-4). Most provinces showed significant downward trends of underweight in children younger than 5 years before 2010, and then, the downward trend slowed down (Tables 1-4). At the municipal level, the underweight prevalence in 95.9% (n=347) of units showed downward trends (Multimedia Appendix 4), and the 3 units with the highest AAPC were Kunming (AAPC –7.87%; *P*<.001), Qujing (AAPC –7.97%; *P*<.001), and Zhaotong (AAPC –8.69%; *P*<.001), which are all in Yunnan province. Three units with the most significant upward trends were Yanbian Korean Autonomous Prefecture (AAPC 1.28%; *P*=.003), Turfan (AAPC 0.98%; *P*=.63), and Changji Hui Autonomous Prefecture (AAPC 0.93%; *P*=.57).

The prevalence was the highest among early neonatal infants in both 2000 and 2019 (15% and 9%, respectively) (Multimedia Appendix 5). From 2000 to 2019, the underweight prevalence declined in all age groups of children (Multimedia Appendix 5), which was similar between boys and girls (Multimedia Appendix 6). The largest decline (AAPC –3.03%; *P*<.001) occurred in children aged 1 to 4 years. The downward trend in prevalence was the lowest among early neonatal infants (AAPC –2.36%; *P*<.001; Multimedia Appendix 5). The trends of underweight prevalence were similar in the late and postneonatal periods, as well as in children aged 1 to 4 years and those aged younger than 5 years (Multimedia Appendix 5).

### Prospects for Reaching 2030 Targets in Children Younger Than 5 Years

**Figure 3:**
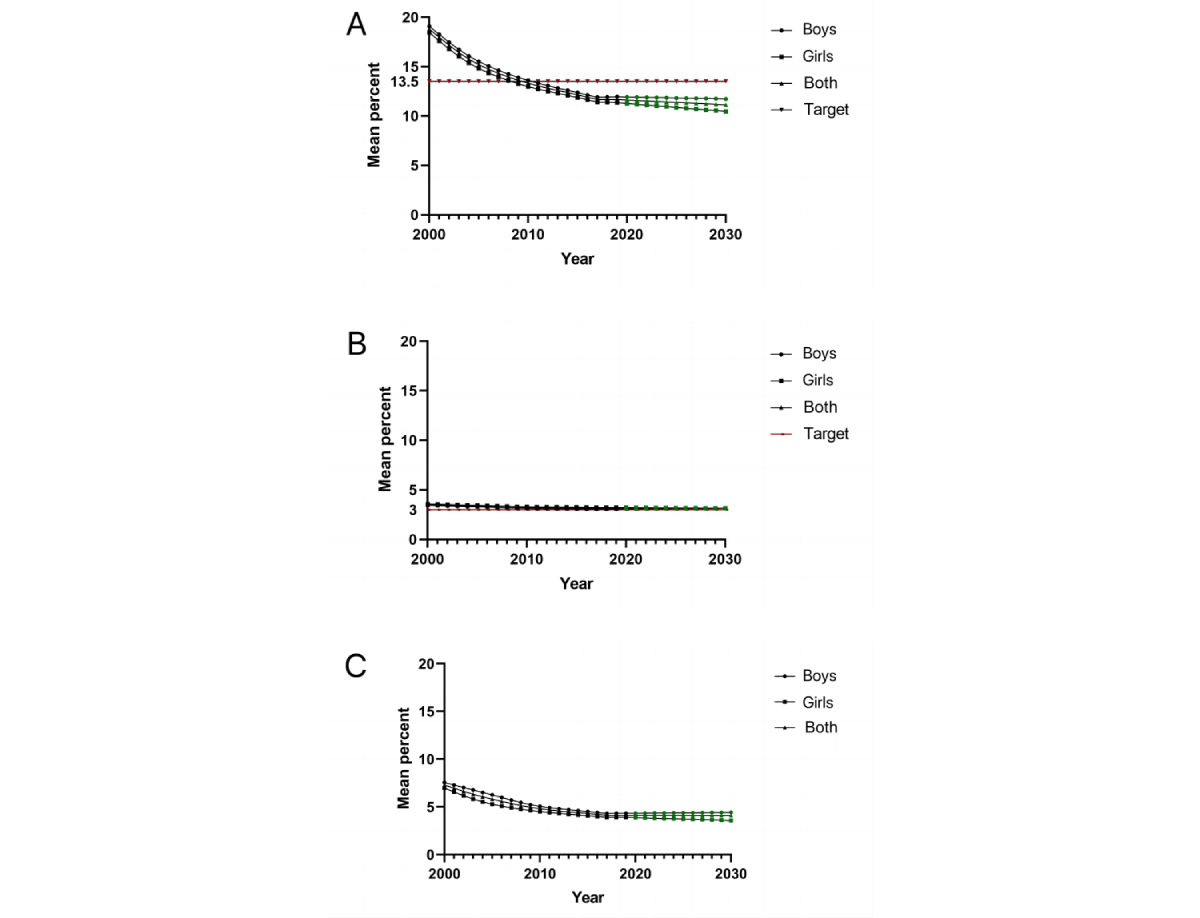
Trends and predictions of child growth failure in children younger than 5 years in China from 2000 to 2030. (A) Stunting trends and predictions; (B) Wasting trends and predictions; (C) Underweight trends and predictions.

It was estimated that the stunting prevalence in children younger than 5 years would decrease to 11.1% (boys: 11.7%, girls: 10.5%) by 2030 (Table 5; Figure 3A). China will achieve the WHO GNT (50% reduction in the number of children with stunting from the baseline in 2012) of 13.5% for stunting (Figure 3A). In fact, China had achieved the target by 2010 (Table 5) at the national level. Although China will achieve the WHO GNT for stunting, it will not meet the target (<5%) of the China National Program for Child Development (2021-2030) based on current trajectories. At the provincial level, 82% of provinces (27/33) will achieve the WHO GNT, and only 9% (3/33; Ningxia, Zhejiang, and Hong Kong) will meet the target (<5%) of the China National Program for Child Development (2021-2030) (Multimedia Appendix 7). The variation of temporary trends according to sex was similar (Table 5; Figure 3). The wasting prevalence is estimated to decrease to 3.2% (boys: 3.2%, girls: 3.2%) in 2030. China might not nationally meet the WHO GNT for wasting (<3%) by 2030 (Table 5; Figure 3B). At the provincial level, 36% of provinces (12/33) will achieve the WHO GNT for wasting (Multimedia Appendix 7). The underweight prevalence will decrease to 4.1% (boys: 4.4%, girls: 3.6%) in 2030 (Figure 3C). Only 33% (11/33) of provinces will meet the WHO GNTs for both stunting and wasting by 2030. The stunting prevalence in all age groups had achieved the 2030 WHO target in 2019. In contrast, the wasting prevalence in all age groups has not achieved the WHO target.

**Table 5:** Projections of child growth failure in children younger than 5 years in China by 2025 and 2030.

Year	Stunting, %				Wasting, %				Underweight, %		
	Male	Female	Both	Male		Female	Both	Male		Female	Both
2025	11.8	10.9	11.4	3.1		3.2	3.2	4.4		3.7	4.1
2030	11.7	10.5	11.1	3.2		3.2	3.2	4.4		3.6	4.1

## Discussion

### Principal Findings

In this study, the prevalences of stunting, wasting, and underweight decreased by 36.9%, 25.0%, and 42.9%, respectively. In addition, the prevalence of CGF showed downward trends from 2000 to 2019 in China as a whole, in all 33 provinces, and in the vast majority of cities, which is in concordance with a previous study based on 2 national surveys between 2002 and 2013 [21].

The explanation for the observed downward trends is likely complex. The crude exclusive breastfeeding rate under 6 months was 20.7% in 2013 [22]. A survey report on the influencing factors of breastfeeding in China showed that in 2019, the exclusive breastfeeding rate under 6 months was 29.2% [23]. A number of national programs promoted healthful food choices and increased support for families and communities. For example, a series of the China National Program for Child Development (2001-2010, 2011-2020, and 2021-2030) whose implementation would provide important guarantees for the realization of children’s rights to survival, development, protection, and participation [24]. The 2000 Dietary Guidelines for Chinese helped the people make beneficial and healthy dietary choices and behavioral changes [25]. Steady progress is seen on mother’s education, the nutrition of women before and during pregnancy, the development of the economy, levels of exclusive breastfeeding, and the implementation of the policy, which are likely to have contributed to these changes [26-29]. Therefore, providing in-depth analysis of the major drivers behind these concerning trends and evidence-based policy recommendations to address them is important.

The downward trend slowed down during the assessment period. Most provinces had stagnated processes of trends after 2017. The signs of increasing hunger globally that first began to appear since 2017 may have led to the trends of CGF in this study. In 2017, China had a relatively low prevalence of national CGF in Asia [5]. Therefore, it is understandable that the downward trend has slowed down.

In first administrative-level units, most provinces reduced disparities between their highest- and lowest-prevalence units between 2000 and 2019. A possible explanation for this might be that health plans (“pilot project to improve children’s nutrition in poor areas”) have been released by the government to reduce the gap between provinces [30]. To improve the health level of children in impoverished areas, the central government has specially arranged subsidy funds for the children’s nutrition improvement project, providing Yingyangbao (YYB; complementary food supplement) with protein, vitamins, and minerals for infants and young children aged 6 to 23 months in concentrated and contiguous poor areas every day [31]. Although most provinces successfully reduced the prevalence of CGF, there remain areas with slow progress. In second administrative-level units, the declines were still uneven. The further reduction of inequality will require targeted strategies to lessen CGF in each province and will need further and much more detailed analysis.

### Findings Among Age Groups

The age group with the highest stunting prevalence was children aged 1 to 4 years, and the highest prevalence of wasting and underweight was noted in early neonatal infants. The prevalence of stunting and underweight declined significantly in all age groups of children from 2000 to 2019. The largest decline in the wasting prevalence occurred in early neonatal infants, and the downward trend among late and postneonatal infants was relatively low. A recent study showed that early postnatal stunting predisposed children to subsequent and persistent stunting [32]. Children who were wasted before the age of 6 months had faster recovery and shorter episodes than did children who were wasted at older ages. The findings of these studies suggest that the period from the prenatal stage to 6 months should be the focus of preventive wasting interventions [33,34]. Thus, early intervention is important. A guideline for healthy nurturing and care of infants and young children younger than 3 years was released in 2022, which targeted children younger than 3 years. Targeted strategies are needed to reduce CGF in children younger than 1 year, and more attention should be paid to late and postneonatal infants.

### Public Health Implications

According to the predictions in this study, China has achieved the goal regarding stunting prevalence. However, the goal regarding wasting prevalence has not been achieved. A national assessment of the patterns of CGF may help policymakers to develop appropriate prevention and management strategies.

### Limitations

This study has some limitations. The analysis heavily depended on data from LBD. There were differences between the data estimated through complex modeling in this study and in earlier studies [15]. In addition, the data were gathered before the popularity of COVID-19. Owing to the potential impacts of the pandemic, predictions based on prepandemic data may be affected. A study showed that the relatively high stunting rate in children younger than 3 years in 2021 may be associated with the influence of COVID-19 [35]. In the future, the data based on China after the pandemic should be modeled.

### Conclusions

Over the past 20 years, the prevalence of CGF has gradually decreased. The downward trend has slowed down, and there remain areas with slow progress. The findings of this cross-sectional study suggest that although widespread declines in CGF have occurred in China, local underachievement and stagnated processes of trends after 2017 are still present. China has nationally met the WHO GNTs for stunting but not for wasting. Furthermore, early interventions for children are crucial.

These findings can be used by governments at the subnational level to identify major problems of CGF and facilitate priority setting. The findings can help to evaluate the effectiveness of national and provincial public health policies and intervention programs in the same period based on the investigation of trends at the provincial level in China in the past 2 decades. Although densely populated areas may have relatively low prevalence of CGF, the absolute number of affected children may still be high; thus, both relative and absolute estimates are important to determine where additional attention is needed.

## Appendix

Multimedia Appendix 1 Prevalence and trends of child growth failure in children younger than 5 years in China from 2000 to 2019.

Multimedia Appendix 2 Prevalence and trends of child growth failure according to sex in children younger than 5 years in China from 2000 to 2019.

Multimedia Appendix 3 Prevalence and trends of child growth failure in children younger than 5 years in 362 municipal-level administrative divisions of China from 2000 to 2019.

Multimedia Appendix 4 Prevalence and trends of child growth failure in boys and girls younger than 5 years in China from 2000 to 2019.

Multimedia Appendix 5 Prevalence and trends of child growth failure in 5 age groups in China from 2000 to 2019.

Multimedia Appendix 6 Prevalence and trends of child growth failure in 5 age groups by sex in China from 2000 to 2019.

Multimedia Appendix 7 Projections of stunting and wasting by 2025 and 2030.

## Abbreviations

AAPC: average annual percentage change
APC: annual percentage change
CGF: child growth failure
GNT: Global Nutrition Target
HAZ: height/length-for-age z-score
IHME: Institute for Health Metrics and Evaluation
JPR: joinpoint regression
LBD: local burden of disease
LMICs: low-income and lower middle–income countries
UI: uncertainty interval
WAZ: weight-for-age z-score
WHO: World Health Organization
WHZ: weight-for-height/length z-score

## Data Availability

The full output of the analyses and the underlying data used in the analyses are publicly available via the Global Health Data Exchange [17]. To download the data used in these analyses, please visit the Institute for Health Metrics and Evaluation customized online data visualization tools [36]. Only publicly available data were used in this study. The datasets generated or analyzed during this study are available from the corresponding author upon reasonable request.
