# Supplementary material for: Temporal Trends in the Prevalence of Child Undernutrition in China From 2000 to 2019, With Projections of Prevalence in 2030: Cross-Sectional Analysis
Source: JMIR Public Health Surveill. 2024 Oct 9;10:e58564. doi: 10.2196/58564 (PMC11499720; doi:10.2196/58564)
Supplement: Multimedia Appendix 1 [file publichealth_v10i1e58564_app1.docx]

**Multimedia Appendix 1.** Prevalence and trends of child growth failure in children younger than 5 years in China from 2000 to 2019.

| **Location** | 2000 | 2019 | AAPC | P-Value |
| --- | --- | --- | --- | --- |
|  | **Stunting** | | | |
| China* | 19 (17 to 21) | 12 (2 to 27) | -2.48  (-2.62 to -2.34) | <.001 |
| China |  |  |  |  |
| Anhui | 18 (13 to 23) | 12 (2 to 32) | -2.08  (-2.22 to -1.93) | <.001 |
| Beijing | 9 (7 to 12) | 8 (1 to 24) | -0.97  (-1.09 to -0.84) | <.001 |
| Chongqing | 17 (13 to 21) | 11 (2 to 27) | -2.40  (-2.47 to -2.33) | <.001 |
| Fujian | 15 (9 to 23) | 10 (1 to 28) | -2.15  (-2.40 to -1.91) | <.001 |
| Gansu | 22 (15 to 28) | 14 (3 to 34) | -2.20  (-2.28 to -2.12) | <.001 |
| Guangdong | 14 (10 to 18) | 9 (1 to 26) | -2.08  (-2.16 to -1.99) | <.001 |
| Guangxi | 26 (23 to 29) | 17 (2 to 43) | -2.28  (-2.40 to -2.16) | <.001 |
| Guizhou | 38 (34 to 43) | 26 (5 to 63) | -2.10  (-2.19 to -2.00) | <.001 |
| Hainan | 17 (15 to 21) | 12 (1 to 36) | -2.03  (-2.12 to -1.94) | <.001 |
| Hebei | 16 (13 to 18) | 11 (2 to 29) | -1.79  (-1.87 to -1.72) | <.001 |
| Heilongjiang | 14 (8 to 24) | 7 (1 to 20) | -4.03  (-4.17 to -3.88) | <.001 |
| Henan | 23 (17 to 29) | 16 (3 to 37) | -2.01  (-2.13 to -1.89) | <.001 |
| Hubei | 21 (15 to 26) | 14 (2 to 37) | -2.00  (-2.28 to -1.71) | <.001 |
| Hunan | 27 (23 to 31) | 20 (3 to 52) | -1.64  (-1.74 to -1.54) | <.001 |
| Jiangsu | 13 (10 to 16) | 9 (1 to 26) | -2.12  (-2.22 to -2.03) | <.001 |
| Jiangxi | 20 (15 to 27) | 12 (2 to 31) | -2.51  (-2.63 to -2.39) | <.001 |
| Jilin | 15 (9 to 22) | 10 (1 to 28) | -1.89  (-2.01 to -1.78) | <.001 |
| Liaoning | 10 (8 to 13) | 7 (1 to 20) | -2.03  (-2.14 to -1.91) | <.001 |
| Inner Mongolia | 14 (12 to 17) | 5 (1 to 15) | -4.88  (-5.10 to -4.66) | <.001 |
| Ningxia | 13 (9 to 17) | 5 (1 to 12) | -5.08  (-5.38 to -4.79) | <.001 |
| Qinghai | 22 (15 to 30) | 15 (3 to 35) | -2.13  (-2.18 to -2.09) | <.001 |
| Shaanxi | 17 (11 to 23) | 11 (2 to 26) | -2.33  (-2.44 to -2.23) | <.001 |
| Shandong | 14 (12 to 17) | 8 (1 to 22) | -3.06  (-3.47 to -2.66) | <.001 |
| Shanghai | 10 (6 to 15) | 9 (1 to 30) | -0.72  (-1.08 to -0.35) | <.001 |
| Shanxi | 17 (12 to 22) | 11 (2 to 28) | -2.19  (-2.34 to -2.03) | <.001 |
| Sichuan | 21 (15 to 27) | 8 (2 to 20) | -4.90  (-5.11 to -4.69) | <.001 |
| Tianjin | 11 (9 to 14) | 8 (1 to 26) | -1.70  (-1.79 to -1.61) | <.001 |
| Xinjiang | 20 (7 to 32) | 13 (2 to 41) | -2.05  (-2.16 to -1.94) | <.001 |
| Xizang | 27 (11 to 40) | 22 (3 to 55) | -1.13  (-1.22 to -1.04) | <.001 |
| Yunnan | 26 (19 to 34) | 12 (2 to 27) | -4.17  (-4.26 to -4.07) | <.001 |
| Zhejiang | 8 (5 to 13) | 4 (0 to 11) | -4.19  (-4.37 to -4.02) | <.001 |
| Hong Kong | 4 (2 to 7) | 3 (0 to 12) | -1.10  (-1.15 to -1.04) | <.001 |
| Macao | 12 (6 to 19) | 10 (1 to 37) | -0.99  (-1.05 to -0.93) | <.001 |
|  | **Wasting** | | | |
| China* | 4 (3 to 5) | 3 (1 to 8) | -0.57  (-0.59 to -0.55) | <.001 |
| China |  |  |  |  |
| Anhui | 3 (2 to 6) | 3 (1 to 8) | -0.33  (-0.39 to -0.27) | <.001 |
| Beijing | 3 (2 to 5) | 3 (1 to 8) | -0.51  (-0.52 to -0.50) | <.001 |
| Chongqing | 3 (2 to 5) | 3 (1 to 8) | -0.29  (-0.31 to -0.27) | <.001 |
| Fujian | 3 (1 to 6) | 3 (1 to 9) | -0.60  (-0.65 to -0.54) | <.001 |
| Gansu | 4 (2 to 6) | 3 (1 to 8) | -0.57  (-0.60 to -0.54) | <.001 |
| Guangdong | 3 (2 to 5) | 3 (1 to 9) | -0.51  (-0.53 to -0.48) | <.001 |
| Guangxi | 6 (4 to 8) | 5 (1 to 15) | -0.38  (-0.41 to -0.35) | <.001 |
| Guizhou | 5 (3 to 7) | 4 (1 to 11) | -0.65  (-0.70 to -0.60) | <.001 |
| Hainan | 3 (2 to 5) | 3 (0 to 10) | -0.65  (-0.67 to -0.64) | <.001 |
| Hebei | 3 (2 to 5) | 3 (1 to 8) | -0.42  (-0.46 to -0.38) | <.001 |
| Heilongjiang | 3 (1 to 6) | 3 (1 to 9) | -0.57  (-0.60 to -0.53) | <.001 |
| Henan | 3 (2 to 5) | 3 (1 to 7) | -0.34  (-0.39 to -0.30) | <.001 |
| Hubei | 3 (2 to 5) | 3 (1 to 8) | -0.58  (-0.62 to -0.55) | <.001 |
| Hunan | 5 (3 to 7) | 4 (1 to 12) | -0.67  (-0.69 to -0.65) | <.001 |
| Jiangsu | 3 (2 to 5) | 3 (1 to 7) | -0.55  (-0.56 to -0.53) | <.001 |
| Jiangxi | 4 (2 to 6) | 3 (1 to 9) | -0.64  (-0.66 to -0.62) | <.001 |
| Jilin | 3 (2 to 6) | 3 (1 to 10) | -0.72  (-0.74 to -0.70) | <.001 |
| Liaoning | 4 (3 to 7) | 4 (1 to 12) | -0.55  (-0.57 to -0.53) | <.001 |
| Inner Mongolia | 3 (2 to 4) | 2 (1 to 6) | -0.41  (-0.43 to -0.39) | <.001 |
| Ningxia | 3 (1 to 5) | 3 (1 to 7) | -0.65  (-0.66 to -0.63) | <.001 |
| Qinghai | 4 (2 to 7) | 3 (1 to 8) | -0.58  (-0.60 to -0.56) | <.001 |
| Shaanxi | 3 (2 to 6) | 3 (1 to 8) | -0.60  (-0.65 to -0.55) | <.001 |
| Shandong | 2 (1 to 4) | 2 (1 to 6) | -0.53  (-0.55 to -0.50) | <.001 |
| Shanghai | 3 (1 to 6) | 3 (1 to 8) | -0.62  (-0.66 to -0.58) | <.001 |
| Shanxi | 3 (2 to 6) | 3 (1 to 8) | -0.32  (-0.34 to -0.30) | <.001 |
| Sichuan | 3 (2 to 6) | 3 (1 to 8) | -0.44  (-0.47 to -0.41) | <.001 |
| Tianjin | 3 (2 to 5) | 3 (1 to 8) | -0.60  (-0.64 to -0.56) | <.001 |
| Xinjiang | 4 (1 to 9) | 3 (1 to 9) | -0.43  (-0.47 to -0.38) | <.001 |
| Xizang | 4 (1 to 9) | 4 (1 to 10) | -0.53  (-0.57 to -0.48) | <.001 |
| Yunnan | 5 (2 to 8) | 4 (1 to 12) | -0.38  (-0.41 to -0.34) | <.001 |
| Zhejiang | 3 (1 to 5) | 3 (1 to 8) | -0.64  (-0.66 to -0.62) | <.001 |
| Hong Kong | 2 (1 to 3) | 2 (0 to 5) | -0.42  (-0.44 to -0.40) | <.001 |
| Macao | 3 (2 to 6) | 3 (1 to 10) | -0.05  (-0.29 to 0.19) | 0.674 |
|  | **Underweight** | | | |
| China* | 7 (6 to 9) | 4 (2 to 9) | -2.95  (-3.02 to -2.87) | <.001 |
| China |  |  |  |  |
| Anhui | 7 (4 to 11) | 4 (1 to 13) | -2.51  (-2.65 to -2.38) | <.001 |
| Beijing | 3 (2 to 6) | 3 (1 to 8) | -1.30  (-1.40 to -1.20) | <.001 |
| Chongqing | 7 (4 to 10) | 4 (1 to 11) | -2.87  (-3.00 to -2.74) | <.001 |
| Fujian | 6 (2 to 12) | 4 (1 to 10) | -2.57  (-2.77 to -2.37) | <.001 |
| Gansu | 9 (4 to 16) | 5 (2 to 13) | -2.82  (-2.87 to -2.78) | <.001 |
| Guangdong | 5 (3 to 9) | 3 (1 to 9) | -2.47  (-2.58 to -2.37) | <.001 |
| Guangxi | 11 (9 to 14) | 8 (2 to 19) | -2.00  (-2.12 to -1.87) | <.001 |
| Guizhou | 17 (13 to 21) | 9 (2 to 25) | -3.10  (-3.18 to -3.02) | <.001 |
| Hainan | 7 (5 to 10) | 4 (1 to 12) | -2.49  (-2.62 to -2.36) | <.001 |
| Hebei | 6 (4 to 9) | 4 (1 to 11) | -2.25  (-2.35 to -2.15) | <.001 |
| Heilongjiang | 5 (2 to 8) | 3 (1 to 10) | -2.24  (-2.35 to -2.13) | <.001 |
| Henan | 8 (4 to 14) | 5 (1 to 13) | -2.60  (-2.64 to -2.56) | <.001 |
| Hubei | 7 (4 to 11) | 4 (1 to 10) | -2.90  (-2.99 to -2.81) | <.001 |
| Hunan | 9 (6 to 12) | 6 (2 to 15) | -2.22  (-2.28 to -2.16) | <.001 |
| Jiangsu | 6 (4 to 9) | 4 (1 to 10) | -2.36  (-2.44 to -2.28) | <.001 |
| Jiangxi | 8 (5 to 14) | 5 (1 to 11) | -3.11  (-3.24 to -2.98) | <.001 |
| Jilin | 6 (3 to 10) | 4 (1 to 11) | -2.36  (-2.44 to -2.29) | <.001 |
| Liaoning | 4 (3 to 7) | 3 (1 to 9) | -2.43  (-2.52 to -2.35) | <.001 |
| Inner Mongolia | 4 (3 to 6) | 2 (1 to 5) | -4.36  (-4.50 to -4.22) | <.001 |
| Ningxia | 5 (2 to 9) | 2 (0 to 5) | -5.10  (-5.31 to -4.88) | <.001 |
| Qinghai | 10 (4 to 16) | 6 (2 to 13) | -2.88  (-2.93 to -2.83) | <.001 |
| Shaanxi | 7 (3 to 12) | 4 (1 to 10) | -2.84  (-2.91 to -2.77) | <.001 |
| Shandong | 3 (2 to 5) | 2 (1 to 5) | -2.41  (-2.55 to -2.27) | <.001 |
| Shanghai | 4 (2 to 8) | 3 (1 to 11) | -1.13  (-1.32 to -0.95) | <.001 |
| Shanxi | 7 (4 to 11) | 4 (1 to 12) | -2.63  (-2.73 to -2.54) | <.001 |
| Sichuan | 8 (5 to 13) | 3 (1 to 8) | -5.36  (-5.47 to -5.25) | <.001 |
| Tianjin | 4 (3 to 7) | 3 (1 to 8) | -2.05  (-2.15 to -1.96) | <.001 |
| Xinjiang | 8 (2 to 19) | 5 (1 to 16) | -2.67  (-2.78 to -2.57) | <.001 |
| Xizang | 12 (5 to 22) | 9 (2 to 27) | -1.83  (-1.91 to -1.74) | <.001 |
| Yunnan | 12 (7 to 19) | 4 (1 to 12) | -5.05  (-5.16 to -4.95) | <.001 |
| Zhejiang | 4 (2 to 7) | 2 (0 to 6) | -4.09  (-4.26 to -3.91) | <.001 |
| Hong Kong | 3 (1 to 5) | 2 (0 to 6) | -0.93  (-0.98 to -0.89) | <.001 |
| Macao | 5 (2 to 9) | 4 (1 to 10) | -1.30  (-1.32 to -1.27) | <.001 |
| *AAPC is expressed as 95 CIs. | | | | |
| *The data of China here covers 31 provinces in mainland China. | | | | |
