# Supplementary material for: Temporal Trends in the Prevalence of Child Undernutrition in China From 2000 to 2019, With Projections of Prevalence in 2030: Cross-Sectional Analysis
Source: JMIR Public Health Surveill. 2024 Oct 9;10:e58564. doi: 10.2196/58564 (PMC11499720; doi:10.2196/58564)
Supplement: Multimedia Appendix 2 [file publichealth_v10i1e58564_app2.docx]

**Multimedia Appendix 2.** Prevalence and trends of child growth failure according to sex in children younger than 5 years in China from 2000 to 2019.

|  | Male | | | | Female | | | | Both | | | |
| --- | --- | --- | --- | --- | --- | --- | --- | --- | --- | --- | --- | --- |
| **Location** | 2000 | 2019 | AAPC | *P*-Value | 2000 | 2019 | AAPC | *P*-Value | 2000 | 2019 | AAPC | *P*-Value |
|  | **Stunting** | | | | | | | | | | | |
| China* | 19  (17 to 21) | 12  (2 to 28) | -2.44  (-2.58 to -2.29 ) | < 0.001 | 18  (17 to 21) | 11  (2 to 26) | -2.53  (-2.68 to -2.38 ) | < 0.001 | 19  (17 to 21) | 12  (2 to 27) | -2.48  (-2.62 to -2.34) | < 0.001 |
| China |  |  |  |  |  |  |  |  |  |  |  |  |
| Anhui | 18  (14 to 23) | 12  (2 to 33) | -2.04  (-2.18 to -1.90 ) | < 0.001 | 17  (13 to 22) | 12  (2 to 31) | -2.13  (-2.27 to -1.99 ) | < 0.001 | 18  (13 to 23) | 12  (2 to 32) | -2.08  (-2.22 to -1.93) | < 0.001 |
| Beijing | 9  (7 to 12) | 8  (1 to 24) | -0.95  (-1.07 to -0.83 ) | < 0.001 | 9  (7 to 12) | 7  (1 to 23) | -0.99  (-1.15 to -0.82 ) | < 0.001 | 9  (7 to 12) | 8  (1 to 24) | -0.97  (-1.09 to -0.84) | < 0.001 |
| Chongqing | 17  (13 to 22) | 11  (2 to 28) | -2.36  (-2.43 to -2.29 ) | < 0.001 | 17  (12 to 21) | 11  (2 to 26) | -2.45  (-2.54 to -2.37 ) | < 0.001 | 17  (13 to 21) | 11  (2 to 27) | -2.40  (-2.47 to -2.33) | < 0.001 |
| Fujian | 15  (9 to 24) | 10  (1 to 28) | -2.10  (-2.30 to -1.90 ) | < 0.001 | 14  (9 to 23) | 9  (1 to 27) | -2.26  (-2.44 to -2.08 ) | < 0.001 | 15  (9 to 23) | 10  (1 to 28) | -2.15  (-2.40 to -1.91) | < 0.001 |
| Gansu | 22  (15 to 29) | 14  (3 to 35) | -2.16  (-2.26 to -2.06 ) | < 0.001 | 21  (15 to 28) | 14  (3 to 33) | -2.25  (-2.31 to -2.19 ) | < 0.001 | 22  (15 to 28) | 14  (3 to 34) | -2.20  (-2.28 to -2.12) | < 0.001 |
| Guangdong | 14  (10 to 19) | 10  (1 to 26) | -2.04  (-2.12 to -1.96 ) | < 0.001 | 14  (10 to 18) | 9  (1 to 25) | -2.13  (-2.23 to -2.03 ) | < 0.001 | 14  (10 to 18) | 9  (1 to 26) | -2.08  (-2.16 to -1.99) | < 0.001 |
| Guangxi | 26  (23 to 30) | 17  (3 to 44) | -2.24  (-2.35 to -2.12 ) | < 0.001 | 25  (23 to 29) | 16  (2 to 41) | -2.33  (-2.47 to -2.20 ) | < 0.001 | 26  (23 to 29) | 17  (2 to 43) | -2.28  (-2.40 to -2.16) | < 0.001 |
| Guizhou | 39  (35 to 44) | 26  (5 to 65) | -2.06  (-2.14 to -1.97 ) | < 0.001 | 38  (34 to 42) | 25  (4 to 62) | -2.15  (-2.27 to -2.03 ) | < 0.001 | 38  (34 to 43) | 26  (5 to 63) | -2.10  (-2.19 to -2.00) | < 0.001 |
| Hainan | 18  (15 to 21) | 12  (1 to 37) | -1.99  (-2.07 to -1.91 ) | < 0.001 | 17  (14 to 21) | 11  (1 to 35) | -2.09  (-2.19 to -1.98 ) | < 0.001 | 17  (15 to 21) | 12  (1 to 36) | -2.03  (-2.12 to -1.94) | < 0.001 |
| Hebei | 16  (13 to 18) | 11  (2 to 29) | -1.75  (-1.81 to -1.69 ) | < 0.001 | 15  (13 to 18) | 11  (2 to 28) | -1.84  (-1.93 to -1.75 ) | < 0.001 | 16  (13 to 18) | 11  (2 to 29) | -1.79  (-1.87 to -1.72) | < 0.001 |
| Heilongjiang | 15  (8 to 24) | 7  (1 to 21) | -3.96  (-4.09 to -3.83 ) | < 0.001 | 14  (7 to 24) | 7  (1 to 20) | -4.06  (-4.19 to -3.94 ) | < 0.001 | 14  (8 to 24) | 7  (1 to 20) | -4.03  (-4.17 to -3.88) | < 0.001 |
| Henan | 23  (17 to 30) | 16  (3 to 38) | -1.97  (-2.05 to -1.89 ) | < 0.001 | 23  (17 to 29) | 15  (3 to 36) | -2.06  (-2.23 to -1.89 ) | < 0.001 | 23  (17 to 29) | 16  (3 to 37) | -2.01  (-2.13 to -1.89) | < 0.001 |
| Hubei | 21  (15 to 27) | 15  (2 to 38) | -1.85  (-1.93 to -1.76 ) | < 0.001 | 20  (15 to 26) | 14  (2 to 36) | -2.01  (-2.24 to -1.79 ) | < 0.001 | 21  (15 to 26) | 14  (2 to 37) | -2.00  (-2.28 to -1.71) | < 0.001 |
| Hunan | 28  (23 to 32) | 20  (4 to 54) | -1.60  (-1.69 to -1.50 ) | < 0.001 | 27  (22 to 31) | 19  (3 to 51) | -1.69  (-1.81 to -1.57 ) | < 0.001 | 27  (23 to 31) | 20  (3 to 52) | -1.64  (-1.74 to -1.54) | < 0.001 |
| Jiangsu | 13  (10 to 17) | 9  (1 to 27) | -2.08  (-2.17 to -1.99 ) | < 0.001 | 13  (10 to 16) | 9  (1 to 25) | -2.25  (-2.39 to -2.11 ) | < 0.001 | 13  (10 to 16) | 9  (1 to 26) | -2.12  (-2.22 to -2.03) | < 0.001 |
| Jiangxi | 21  (15 to 27) | 13  (2 to 32) | -2.47  (-2.58 to -2.35 ) | < 0.001 | 20  (15 to 27) | 12  (2 to 30) | -2.56  (-2.69 to -2.43 ) | < 0.001 | 20  (15 to 27) | 12  (2 to 31) | -2.51  (-2.63 to -2.39) | < 0.001 |
| Jilin | 15  (9 to 22) | 10  (1 to 29) | -1.85  (-1.97 to -1.73 ) | < 0.001 | 14  (9 to 21) | 10  (1 to 27) | -1.94  (-2.06 to -1.83 ) | < 0.001 | 15  (9 to 22) | 10  (1 to 28) | -1.89  (-2.01 to -1.78) | < 0.001 |
| Liaoning | 10  (8 to 13) | 7  (1 to 21) | -2.01  (-2.16 to -1.85 ) | < 0.001 | 10  (8 to 13) | 7  (1 to 20) | -2.04  (-2.10 to -1.99 ) | < 0.001 | 10  (8 to 13) | 7  (1 to 20) | -2.03  (-2.14 to -1.91) | < 0.001 |
| Inner Mongolia | 14  (12 to 17) | 6  (1 to 15) | -4.84  (-5.03 to -4.66 ) | < 0.001 | 14  (12 to 16) | 5  (1 to 15) | -4.99  (-5.14 to -4.84 ) | < 0.001 | 14  (12 to 17) | 5  (1 to 15) | -4.88  (-5.10 to -4.66) | < 0.001 |
| Ningxia | 13  (9 to 18) | 5  (1 to 13) | -5.04  (-5.30 to -4.79 ) | < 0.001 | 13  (8 to 17) | 5  (1 to 12) | -5.13  (-5.44 to -4.81 ) | < 0.001 | 13  (9 to 17) | 5  (1 to 12) | -5.08  (-5.38 to -4.79) | < 0.001 |
| Qinghai | 23  (15 to 31) | 15  (3 to 36) | -2.09  (-2.12 to -2.06 ) | < 0.001 | 22  (14 to 30) | 15  (3 to 34) | -2.19  (-2.24 to -2.13 ) | < 0.001 | 22  (15 to 30) | 15  (3 to 35) | -2.13  (-2.18 to -2.09) | < 0.001 |
| Shaanxi | 17  (11 to 23) | 11  (2 to 26) | -2.29  (-2.39 to -2.19 ) | < 0.001 | 16  (11 to 22) | 10  (2 to 25) | -2.38  (-2.49 to -2.27 ) | < 0.001 | 17  (11 to 23) | 11  (2 to 26) | -2.33  (-2.44 to -2.23) | < 0.001 |
| Shandong | 14  (12 to 17) | 8  (1 to 23) | -3.02  (-3.18 to -2.86 ) | < 0.001 | 14  (11 to 16) | 8  (1 to 21) | -3.28  (-3.66 to -2.90 ) | < 0.001 | 14  (12 to 17) | 8  (1 to 22) | -3.06  (-3.47 to -2.66) | < 0.001 |
| Shanghai | 10  (6 to 16) | 9  (1 to 31) | -0.70  (-1.04 to -0.36 ) | < 0.001 | 10  (6 to 15) | 8  (1 to 30) | -0.74  (-1.14 to -0.33 ) | < 0.001 | 10  (6 to 15) | 9  (1 to 30) | -0.72  (-1.08 to -0.35) | < 0.001 |
| Shanxi | 17  (12 to 22) | 11  (2 to 29) | -2.15  (-2.29 to -2.00 ) | < 0.001 | 17  (12 to 22) | 11  (2 to 27) | -2.24  (-2.37 to -2.11 ) | < 0.001 | 17  (12 to 22) | 11  (2 to 28) | -2.19  (-2.34 to -2.03) | < 0.001 |
| Sichuan | 22  (16 to 28) | 9  (2 to 20) | -4.87  (-5.07 to -4.67 ) | < 0.001 | 21  (15 to 27) | 8  (2 to 19) | -4.88  (-5.44 to -4.33 ) | < 0.001 | 21  (15 to 27) | 8  (2 to 20) | -4.90  (-5.11 to -4.69) | < 0.001 |
| Tianjin | 11  (9 to 14) | 8  (1 to 26) | -1.68  (-1.80 to -1.57 ) | < 0.001 | 11  (8 to 14) | 8  (1 to 25) | -1.73  (-1.80 to -1.67 ) | < 0.001 | 11  (9 to 14) | 8  (1 to 26) | -1.70  (-1.79 to -1.61) | < 0.001 |
| Xinjiang | 20  (7 to 32) | 14  (2 to 42) | -2.00  (-2.11 to -1.90 ) | < 0.001 | 19  (7 to 31) | 13  (1 to 40) | -2.10  (-2.22 to -1.98 ) | < 0.001 | 20  (7 to 32) | 13  (2 to 41) | -2.05  (-2.16 to -1.94) | < 0.001 |
| Xizang | 27  (11 to 41) | 22  (3 to 56) | -1.09  (-1.15 to -1.03 ) | < 0.001 | 26  (11 to 39) | 21  (3 to 53) | -1.18  (-1.26 to -1.10 ) | < 0.001 | 27  (11 to 40) | 22  (3 to 55) | -1.13  (-1.22 to -1.04) | < 0.001 |
| Yunnan | 26  (19 to 34) | 12  (2 to 27) | -4.13  (-4.23 to -4.02 ) | < 0.001 | 26  (18 to 33) | 11  (2 to 26) | -4.22  (-4.31 to -4.13 ) | < 0.001 | 26  (19 to 34) | 12  (2 to 27) | -4.17  (-4.26 to -4.07) | < 0.001 |
| Zhejiang | 8  (5 to 13) | 4  (0 to 12) | -4.17  (-4.36 to -3.98 ) | < 0.001 | 8  (5 to 12) | 4  (0 to 11) | -4.22  (-4.38 to -4.07 ) | < 0.001 | 8  (5 to 13) | 4  (0 to 11) | -4.19  (-4.37 to -4.02) | < 0.001 |
| Hong Kong | 4  (2 to 7) | 4  (0 to 13) | -1.06  (-1.11 to -1.00 ) | < 0.001 | 4  (2 to 7) | 3  (0 to 12) | -1.15  (-1.21 to -1.08 ) | < 0.001 | 4  (2 to 7) | 3  (0 to 12) | -1.10  (-1.15 to -1.04) | < 0.001 |
| Macao | 12  (6 to 19) | 10  (1 to 38) | -0.94  (-1.01 to -0.87 ) | < 0.001 | 12  (6 to 19) | 9  (1 to 36) | -1.04  (-1.09 to -0.99 ) | < 0.001 | 12  (6 to 19) | 10  (1 to 37) | -0.99  (-1.05 to -0.93) | < 0.001 |
|  | **Wasting** | | | | | | | | | | | |
| China* | 3  (3 to 5) | 3  (1 to 7) | -0.59  (-0.61 to -0.57 ) | < 0.001 | 4  (3 to 5) | 3  (1 to 8) | -0.56  (-0.59 to -0.53 ) | < 0.001 | 4  (3 to 5) | 3  (1 to 8) | -0.57  (-0.59 to -0.55) | < 0.001 |
| China |  |  |  |  |  |  |  |  |  |  |  |  |
| Anhui | 3  (2 to 6) | 3  (1 to 8) | -0.53  (-0.54 to -0.52 ) | < 0.001 | 4  (2 to 6) | 3  (1 to 9) | -0.49  (-0.50 to -0.48 ) | < 0.001 | 3  (2 to 6) | 3  (1 to 8) | -0.33  (-0.39 to -0.27) | < 0.001 |
| Beijing | 3  (2 to 5) | 3  (1 to 8) | -0.31  (-0.34 to -0.27 ) | < 0.001 | 3  (2 to 5) | 3  (1 to 8) | -0.27  (-0.28 to -0.25 ) | < 0.001 | 3  (2 to 5) | 3  (1 to 8) | -0.51  (-0.52 to -0.50) | < 0.001 |
| Chongqing | 3  (2 to 5) | 3  (1 to 8) | -0.60  (-0.63 to -0.58 ) | < 0.001 | 4  (2 to 6) | 3  (1 to 9) | -0.56  (-0.60 to -0.53 ) | < 0.001 | 3  (2 to 5) | 3  (1 to 8) | -0.29  (-0.31 to -0.27) | < 0.001 |
| Fujian | 3  (1 to 6) | 3  (1 to 8) | -0.59  (-0.62 to -0.56 ) | < 0.001 | 3  (1 to 6) | 3  (1 to 9) | -0.55  (-0.58 to -0.52 ) | < 0.001 | 3  (1 to 6) | 3  (1 to 9) | -0.60  (-0.65 to -0.54) | < 0.001 |
| Gansu | 4  (2 to 6) | 3  (1 to 8) | -0.52  (-0.55 to -0.50 ) | < 0.001 | 4  (2 to 6) | 3  (1 to 9) | -0.49  (-0.52 to -0.45 ) | < 0.001 | 4  (2 to 6) | 3  (1 to 8) | -0.57  (-0.60 to -0.54) | < 0.001 |
| Guangdong | 3  (2 to 5) | 3  (1 to 9) | -0.41  (-0.46 to -0.36 ) | < 0.001 | 3  (2 to 6) | 3  (1 to 9) | -0.36  (-0.38 to -0.34 ) | < 0.001 | 3  (2 to 5) | 3  (1 to 9) | -0.51  (-0.53 to -0.48) | < 0.001 |
| Guangxi | 6  (4 to 8) | 5  (1 to 14) | -0.65  (-0.67 to -0.63 ) | < 0.001 | 6  (4 to 8) | 5  (1 to 15) | -0.61  (-0.63 to -0.59 ) | < 0.001 | 6  (4 to 8) | 5  (1 to 15) | -0.38  (-0.41 to -0.35) | < 0.001 |
| Guizhou | 4  (3 to 7) | 4  (1 to 11) | -0.67  (-0.69 to -0.66 ) | < 0.001 | 5  (3 to 7) | 4  (1 to 12) | -0.63  (-0.66 to -0.61 ) | < 0.001 | 5  (3 to 7) | 4  (1 to 11) | -0.65  (-0.70 to -0.60) | < 0.001 |
| Hainan | 3  (2 to 5) | 3  (0 to 10) | -0.46  (-0.53 to -0.38 ) | < 0.001 | 3  (2 to 6) | 3  (1 to 10) | -0.39  (-0.42 to -0.36 ) | < 0.001 | 3  (2 to 5) | 3  (0 to 10) | -0.65  (-0.67 to -0.64) | < 0.001 |
| Hebei | 3  (2 to 5) | 3  (1 to 8) | -0.59  (-0.62 to -0.55 ) | < 0.001 | 3  (2 to 5) | 3  (1 to 9) | -0.53  (-0.55 to -0.52 ) | < 0.001 | 3  (2 to 5) | 3  (1 to 8) | -0.42  (-0.46 to -0.38) | < 0.001 |
| Heilongjiang | 3  (1 to 6) | 3  (1 to 9) | -0.37  (-0.39 to -0.34 ) | < 0.001 | 3  (1 to 6) | 3  (1 to 9) | -0.32  (-0.39 to -0.24 ) | < 0.001 | 3  (1 to 6) | 3  (1 to 9) | -0.57  (-0.60 to -0.53) | < 0.001 |
| Henan | 3  (2 to 5) | 3  (1 to 7) | -0.60  (-0.63 to -0.58 ) | < 0.001 | 3  (2 to 5) | 3  (1 to 8) | -0.57  (-0.59 to -0.54 ) | < 0.001 | 3  (2 to 5) | 3  (1 to 7) | -0.34  (-0.39 to -0.30) | < 0.001 |
| Hubei | 3  (2 to 5) | 3  (1 to 8) | -0.69  (-0.72 to -0.67 ) | < 0.001 | 3  (2 to 5) | 3  (1 to 8) | -0.66  (-0.70 to -0.62 ) | < 0.001 | 3  (2 to 5) | 3  (1 to 8) | -0.58  (-0.62 to -0.55) | < 0.001 |
| Hunan | 5  (3 to 7) | 4  (1 to 12) | -0.57  (-0.58 to -0.56 ) | < 0.001 | 5  (3 to 7) | 4  (1 to 12) | -0.54  (-0.57 to -0.51 ) | < 0.001 | 5  (3 to 7) | 4  (1 to 12) | -0.67  (-0.69 to -0.65) | < 0.001 |
| Jiangsu | 3  (2 to 5) | 3  (1 to 7) | -0.66  (-0.69 to -0.63 ) | < 0.001 | 3  (2 to 5) | 3  (1 to 7) | -0.62  (-0.65 to -0.59 ) | < 0.001 | 3  (2 to 5) | 3  (1 to 7) | -0.55  (-0.56 to -0.53) | < 0.001 |
| Jiangxi | 4  (2 to 6) | 3  (1 to 9) | -0.73  (-0.75 to -0.72 ) | < 0.001 | 4  (2 to 6) | 3  (1 to 9) | -0.70  (-0.73 to -0.68 ) | < 0.001 | 4  (2 to 6) | 3  (1 to 9) | -0.64  (-0.66 to -0.62) | < 0.001 |
| Jilin | 3  (2 to 6) | 3  (1 to 10) | -0.57  (-0.60 to -0.55 ) | < 0.001 | 3  (2 to 6) | 3  (1 to 11) | -0.52  (-0.55 to -0.49 ) | < 0.001 | 3  (2 to 6) | 3  (1 to 10) | -0.72  (-0.74 to -0.70) | < 0.001 |
| Liaoning | 4  (3 to 7) | 4  (1 to 12) | -0.43  (-0.45 to -0.42 ) | < 0.001 | 5  (3 to 7) | 4  (1 to 13) | -0.38  (-0.41 to -0.34 ) | < 0.001 | 4  (3 to 7) | 4  (1 to 12) | -0.55  (-0.57 to -0.53) | < 0.001 |
| Inner Mongolia | 2  (2 to 4) | 2  (1 to 6) | -0.67  (-0.69 to -0.64 ) | < 0.001 | 3  (2 to 4) | 2  (1 to 6) | -0.62  (-0.65 to -0.59 ) | < 0.001 | 3  (2 to 4) | 2  (1 to 6) | -0.41  (-0.43 to -0.39) | < 0.001 |
| Ningxia | 3  (1 to 5) | 3  (1 to 7) | -0.61  (-0.63 to -0.58 ) | < 0.001 | 3  (1 to 5) | 3  (1 to 7) | -0.55  (-0.58 to -0.52 ) | < 0.001 | 3  (1 to 5) | 3  (1 to 7) | -0.65  (-0.66 to -0.63) | < 0.001 |
| Qinghai | 4  (2 to 6) | 3  (1 to 8) | -0.61  (-0.63 to -0.59 ) | < 0.001 | 4  (2 to 7) | 3  (1 to 8) | -0.57  (-0.58 to -0.55 ) | < 0.001 | 4  (2 to 7) | 3  (1 to 8) | -0.58  (-0.60 to -0.56) | < 0.001 |
| Shaanxi | 3  (2 to 6) | 3  (1 to 8) | -0.55  (-0.57 to -0.52 ) | < 0.001 | 3  (2 to 6) | 3  (1 to 9) | -0.51  (-0.54 to -0.48 ) | < 0.001 | 3  (2 to 6) | 3  (1 to 8) | -0.60  (-0.65 to -0.55) | < 0.001 |
| Shandong | 2  (1 to 4) | 2  (0 to 5) | -0.63  (-0.65 to -0.60 ) | < 0.001 | 2  (2 to 4) | 2  (1 to 6) | -0.58  (-0.60 to -0.56 ) | < 0.001 | 2  (1 to 4) | 2  (1 to 6) | -0.53  (-0.55 to -0.50) | < 0.001 |
| Shanghai | 3  (1 to 6) | 3  (1 to 8) | -0.34  (-0.35 to -0.32 ) | < 0.001 | 3  (1 to 6) | 3  (1 to 8) | -0.30  (-0.33 to -0.26 ) | < 0.001 | 3  (1 to 6) | 3  (1 to 8) | -0.62  (-0.66 to -0.58) | < 0.001 |
| Shanxi | 3  (2 to 6) | 3  (1 to 8) | -0.46  (-0.49 to -0.43 ) | < 0.001 | 3  (2 to 6) | 3  (1 to 8) | -0.41  (-0.45 to -0.36 ) | < 0.001 | 3  (2 to 6) | 3  (1 to 8) | -0.32  (-0.34 to -0.30) | < 0.001 |
| Sichuan | 3  (2 to 6) | 3  (1 to 8) | -0.62  (-0.67 to -0.57 ) | < 0.001 | 4  (2 to 6) | 3  (1 to 9) | -0.58  (-0.61 to -0.56 ) | < 0.001 | 3  (2 to 6) | 3  (1 to 8) | -0.44  (-0.47 to -0.41) | < 0.001 |
| Tianjin | 3  (2 to 5) | 3  (1 to 8) | -0.44  (-0.46 to -0.42 ) | < 0.001 | 3  (2 to 5) | 3  (1 to 8) | -0.40  (-0.42 to -0.38 ) | < 0.001 | 3  (2 to 5) | 3  (1 to 8) | -0.60  (-0.64 to -0.56) | < 0.001 |
| Xinjiang | 3  (1 to 9) | 3  (1 to 9) | -0.54  (-0.55 to -0.52 ) | < 0.001 | 4  (1 to 9) | 3  (1 to 10) | -0.49  (-0.52 to -0.47 ) | < 0.001 | 4  (1 to 9) | 3  (1 to 9) | -0.43  (-0.47 to -0.38) | < 0.001 |
| Xizang | 4  (1 to 9) | 4  (1 to 10) | -0.39  (-0.41 to -0.38 ) | < 0.001 | 4  (1 to 9) | 4  (1 to 10) | -0.35  (-0.37 to -0.32 ) | < 0.001 | 4  (1 to 9) | 4  (1 to 10) | -0.53  (-0.57 to -0.48) | < 0.001 |
| Yunnan | 5  (2 to 8) | 4  (1 to 11) | -0.66  (-0.67 to -0.64 ) | < 0.001 | 5  (2 to 8) | 4  (1 to 12) | -0.62  (-0.66 to -0.58 ) | < 0.001 | 5  (2 to 8) | 4  (1 to 12) | -0.38  (-0.41 to -0.34) | < 0.001 |
| Zhejiang | 3  (1 to 5) | 3  (1 to 7) | -0.45  (-0.49 to -0.41 ) | < 0.001 | 3  (1 to 6) | 3  (1 to 8) | -0.40  (-0.43 to -0.38 ) | < 0.001 | 3  (1 to 5) | 3  (1 to 8) | -0.64  (-0.66 to -0.62) | < 0.001 |
| Hong Kong | 2  (1 to 3) | 2  (0 to 5) | -0.07  (-0.31 to 0.17 ) | 0.559 | 2  (1 to 3) | 2  (0 to 6) | -0.03  (-0.27 to 0.21 ) | 0.806 | 2  (1 to 3) | 2  (0 to 5) | -0.42  (-0.44 to -0.40) | < 0.001 |
| Macao | 3  (2 to 6) | 3  (1 to 10) | -0.20  (-0.26 to -0.14 ) | < 0.001 | 3  (2 to 6) | 3  (1 to 10) | -0.16  (-0.22 to -0.10 ) | < 0.001 | 3  (2 to 6) | 3  (1 to 10) | -0.05  (-0.29 to 0.19) | 0.674 |
|  | **Underweight** | | | | | | | | | | | |
| China* | 8  (6 to 9) | 4  (2 to 10) | -2.90  (-2.96 to -2.84 ) | < 0.001 | 7  (6 to 9) | 4  (2 to 9) | -3.01  (-3.16 to -2.86 ) | < 0.001 | 7  (6 to 9) | 4  (2 to 9) | -2.95  (-3.02 to -2.87) | < 0.001 |
| China |  |  |  |  |  |  |  |  |  |  |  |  |
| Anhui | 7  (4 to 12) | 5  (1 to 13) | -2.47  (-2.59 to -2.34 ) | < 0.001 | 7  (4 to 11) | 4  (1 to 12) | -2.58  (-2.77 to -2.39 ) | < 0.001 | 7  (4 to 11) | 4  (1 to 13) | -2.51  (-2.65 to -2.38) | < 0.001 |
| Beijing | 4  (2 to 6) | 3  (1 to 8) | -1.24  (-1.30 to -1.17 ) | < 0.001 | 3  (2 to 6) | 3  (1 to 7) | -1.36  (-1.45 to -1.28 ) | < 0.001 | 3  (2 to 6) | 3  (1 to 8) | -1.30  (-1.40 to -1.20) | < 0.001 |
| Chongqing | 7  (4 to 11) | 4  (1 to 11) | -2.82  (-2.88 to -2.77 ) | < 0.001 | 7  (4 to 10) | 4  (1 to 10) | -2.93  (-3.04 to -2.83 ) | < 0.001 | 7  (4 to 10) | 4  (1 to 11) | -2.87  (-3.00 to -2.74) | < 0.001 |
| Fujian | 6  (2 to 12) | 4  (1 to 10) | -2.52  (-2.62 to -2.41 ) | < 0.001 | 6  (2 to 11) | 3  (1 to 9) | -2.65  (-2.76 to -2.54 ) | < 0.001 | 6  (2 to 12) | 4  (1 to 10) | -2.57  (-2.77 to -2.37) | < 0.001 |
| Gansu | 9  (4 to 17) | 5  (2 to 13) | -2.78  (-2.82 to -2.74 ) | < 0.001 | 9  (4 to 15) | 5  (2 to 12) | -2.88  (-2.95 to -2.81 ) | < 0.001 | 9  (4 to 16) | 5  (2 to 13) | -2.82  (-2.87 to -2.78) | < 0.001 |
| Guangdong | 6  (3 to 9) | 4  (1 to 9) | -2.42  (-2.56 to -2.28 ) | < 0.001 | 5  (3 to 8) | 3  (1 to 8) | -2.53  (-2.71 to -2.35 ) | < 0.001 | 5  (3 to 9) | 3  (1 to 9) | -2.47  (-2.58 to -2.37) | < 0.001 |
| Guangxi | 12  (9 to 14) | 8  (2 to 20) | -1.95  (-2.04 to -1.85 ) | < 0.001 | 11  (9 to 13) | 7  (2 to 18) | -2.13  (-2.34 to -1.92 ) | < 0.001 | 11  (9 to 14) | 8  (2 to 19) | -2.00  (-2.12 to -1.87) | < 0.001 |
| Guizhou | 18  (14 to 22) | 10  (3 to 27) | -3.05  (-3.22 to -2.89 ) | < 0.001 | 16  (13 to 20) | 9  (2 to 24) | -3.16  (-3.24 to -3.08 ) | < 0.001 | 17  (13 to 21) | 9  (2 to 25) | -3.10  (-3.18 to -3.02) | < 0.001 |
| Hainan | 7  (5 to 10) | 5  (1 to 13) | -2.44  (-2.52 to -2.37 ) | < 0.001 | 7  (5 to 9) | 4  (1 to 12) | -2.56  (-2.64 to -2.47 ) | < 0.001 | 7  (5 to 10) | 4  (1 to 12) | -2.49  (-2.62 to -2.36) | < 0.001 |
| Hebei | 6  (4 to 9) | 4  (1 to 12) | -2.20  (-2.31 to -2.09 ) | < 0.001 | 6  (4 to 9) | 4  (1 to 11) | -2.31  (-2.45 to -2.17 ) | < 0.001 | 6  (4 to 9) | 4  (1 to 11) | -2.25  (-2.35 to -2.15) | < 0.001 |
| Heilongjiang | 5  (2 to 9) | 3  (1 to 11) | -2.19  (-2.30 to -2.09 ) | < 0.001 | 5  (2 to 8) | 3  (1 to 10) | -2.29  (-2.40 to -2.17 ) | < 0.001 | 5  (2 to 8) | 3  (1 to 10) | -2.24  (-2.35 to -2.13) | < 0.001 |
| Henan | 8  (5 to 14) | 5  (2 to 13) | -2.56  (-2.64 to -2.48 ) | < 0.001 | 8  (4 to 13) | 5  (1 to 12) | -2.65  (-2.71 to -2.59 ) | < 0.001 | 8  (4 to 14) | 5  (1 to 13) | -2.60  (-2.64 to -2.56) | < 0.001 |
| Hubei | 7  (4 to 11) | 4  (1 to 11) | -2.85  (-2.91 to -2.80 ) | < 0.001 | 7  (4 to 10) | 4  (1 to 10) | -2.98  (-3.13 to -2.83 ) | < 0.001 | 7  (4 to 11) | 4  (1 to 10) | -2.90  (-2.99 to -2.81) | < 0.001 |
| Hunan | 9  (7 to 12) | 6  (2 to 15) | -2.17  (-2.23 to -2.12 ) | < 0.001 | 9  (6 to 11) | 6  (2 to 14) | -2.28  (-2.37 to -2.20 ) | < 0.001 | 9  (6 to 12) | 6  (2 to 15) | -2.22  (-2.28 to -2.16) | < 0.001 |
| Jiangsu | 6  (4 to 9) | 4  (1 to 11) | -2.31  (-2.36 to -2.26 ) | < 0.001 | 5  (3 to 8) | 3  (1 to 10) | -2.42  (-2.54 to -2.29 ) | < 0.001 | 6  (4 to 9) | 4  (1 to 10) | -2.36  (-2.44 to -2.28) | < 0.001 |
| Jiangxi | 9  (5 to 14) | 5  (1 to 12) | -3.07  (-3.13 to -3.02 ) | < 0.001 | 8  (5 to 13) | 4  (1 to 11) | -3.18  (-3.33 to -3.02 ) | < 0.001 | 8  (5 to 14) | 5  (1 to 11) | -3.11  (-3.24 to -2.98) | < 0.001 |
| Jilin | 6  (3 to 10) | 4  (1 to 12) | -2.32  (-2.40 to -2.23 ) | < 0.001 | 6  (3 to 9) | 3  (1 to 11) | -2.43  (-2.56 to -2.31 ) | < 0.001 | 6  (3 to 10) | 4  (1 to 11) | -2.36  (-2.44 to -2.29) | < 0.001 |
| Liaoning | 5  (3 to 7) | 3  (1 to 9) | -2.38  (-2.52 to -2.24 ) | < 0.001 | 4  (3 to 6) | 3  (1 to 8) | -2.50  (-2.60 to -2.40 ) | < 0.001 | 4  (3 to 7) | 3  (1 to 9) | -2.43  (-2.52 to -2.35) | < 0.001 |
| Inner Mongolia | 5  (3 to 6) | 2  (1 to 5) | -4.24  (-4.42 to -4.07 ) | < 0.001 | 4  (3 to 6) | 2  (1 to 5) | -4.43  (-4.61 to -4.25 ) | < 0.001 | 4  (3 to 6) | 2  (1 to 5) | -4.36  (-4.50 to -4.22) | < 0.001 |
| Ningxia | 5  (2 to 10) | 2  (0 to 5) | -5.10  (-5.37 to -4.84 ) | < 0.001 | 5  (2 to 9) | 2  (0 to 5) | -5.25  (-5.57 to -4.93 ) | < 0.001 | 5  (2 to 9) | 2  (0 to 5) | -5.10  (-5.31 to -4.88) | < 0.001 |
| Qinghai | 10  (4 to 17) | 6  (2 to 13) | -2.84  (-2.89 to -2.79 ) | < 0.001 | 9  (4 to 15) | 5  (1 to 12) | -2.93  (-3.03 to -2.84 ) | < 0.001 | 10  (4 to 16) | 6  (2 to 13) | -2.88  (-2.93 to -2.83) | < 0.001 |
| Shaanxi | 7  (3 to 13) | 4  (1 to 11) | -2.80  (-2.85 to -2.75 ) | < 0.001 | 6  (3 to 12) | 4  (1 to 10) | -2.90  (-3.01 to -2.79 ) | < 0.001 | 7  (3 to 12) | 4  (1 to 10) | -2.84  (-2.91 to -2.77) | < 0.001 |
| Shandong | 3  (2 to 5) | 2  (1 to 6) | -2.37  (-2.45 to -2.29 ) | < 0.001 | 3  (2 to 5) | 2  (1 to 5) | -2.49  (-2.64 to -2.35 ) | < 0.001 | 3  (2 to 5) | 2  (1 to 5) | -2.41  (-2.55 to -2.27) | < 0.001 |
| Shanghai | 4  (2 to 8) | 3  (1 to 12) | -1.09  (-1.24 to -0.93 ) | < 0.001 | 4  (2 to 7) | 3  (1 to 10) | -1.18  (-1.39 to -0.96 ) | < 0.001 | 4  (2 to 8) | 3  (1 to 11) | -1.13  (-1.32 to -0.95) | < 0.001 |
| Shanxi | 7  (4 to 11) | 4  (1 to 12) | -2.58  (-2.67 to -2.49 ) | < 0.001 | 6  (3 to 10) | 4  (1 to 11) | -2.73  (-2.85 to -2.61 ) | < 0.001 | 7  (4 to 11) | 4  (1 to 12) | -2.63  (-2.73 to -2.54) | < 0.001 |
| Sichuan | 9  (5 to 14) | 3  (1 to 8) | -5.32  (-5.47 to -5.17 ) | < 0.001 | 8  (5 to 13) | 3  (1 to 7) | -5.37  (-5.55 to -5.18 ) | < 0.001 | 8  (5 to 13) | 3  (1 to 8) | -5.36  (-5.47 to -5.25) | < 0.001 |
| Tianjin | 4  (3 to 7) | 3  (1 to 9) | -2.01  (-2.06 to -1.95 ) | < 0.001 | 4  (2 to 7) | 3  (1 to 8) | -2.16  (-2.28 to -2.03 ) | < 0.001 | 4  (3 to 7) | 3  (1 to 8) | -2.05  (-2.15 to -1.96) | < 0.001 |
| Xinjiang | 9  (2 to 19) | 5  (1 to 16) | -2.64  (-2.73 to -2.55 ) | < 0.001 | 8  (2 to 18) | 5  (1 to 15) | -2.75  (-2.86 to -2.63 ) | < 0.001 | 8  (2 to 19) | 5  (1 to 16) | -2.67  (-2.78 to -2.57) | < 0.001 |
| Xizang | 13  (5 to 23) | 9  (2 to 28) | -1.79  (-1.87 to -1.71 ) | < 0.001 | 12  (5 to 21) | 8  (2 to 25) | -1.89  (-2.11 to -1.67 ) | < 0.001 | 12  (5 to 22) | 9  (2 to 27) | -1.83  (-1.91 to -1.74) | < 0.001 |
| Yunnan | 12  (7 to 20) | 5  (1 to 12) | -5.02  (-5.18 to -4.86 ) | < 0.001 | 11  (7 to 18) | 4  (1 to 11) | -5.11  (-5.20 to -5.02 ) | < 0.001 | 12  (7 to 19) | 4  (1 to 12) | -5.05  (-5.16 to -4.95) | < 0.001 |
| Zhejiang | 4  (2 to 8) | 2  (0 to 6) | -3.95  (-4.14 to -3.75 ) | < 0.001 | 4  (2 to 7) | 2  (0 to 5) | -4.16  (-4.40 to -3.91 ) | < 0.001 | 4  (2 to 7) | 2  (0 to 6) | -4.09  (-4.26 to -3.91) | < 0.001 |
| Hong Kong | 3  (1 to 5) | 2  (0 to 6) | -0.89  (-0.94 to -0.85 ) | < 0.001 | 2  (1 to 5) | 2  (0 to 5) | -1.00  (-1.06 to -0.93 ) | < 0.001 | 3  (1 to 5) | 2  (0 to 6) | -0.93  (-0.98 to -0.89) | < 0.001 |
| Macao | 5  (2 to 9) | 4  (1 to 10) | -1.24  (-1.28 to -1.20 ) | < 0.001 | 4  (2 to 8) | 3  (1 to 9) | -1.34  (-1.41 to -1.27 ) | < 0.001 | 5  (2 to 9) | 4  (1 to 10) | -1.30  (-1.32 to -1.27) | < 0.001 |
| *AAPC is expressed as 95 CIs. | | | | | | | | | | | | |
| *The data of China here covers 31 provinces in mainland China. | | | | | | | | | | | | |
