# Supplementary material for: Temporal Trends in the Prevalence of Child Undernutrition in China From 2000 to 2019, With Projections of Prevalence in 2030: Cross-Sectional Analysis
Source: JMIR Public Health Surveill. 2024 Oct 9;10:e58564. doi: 10.2196/58564 (PMC11499720; doi:10.2196/58564)
Supplement: Multimedia Appendix 3 [file publichealth_v10i1e58564_app3.docx]

**Multimedia Appendix 3.** Prevalence and trends of child growth failure in children younger than 5 years in 362 municipal-level administrative divisions of China from 2000 to 2019.

|  | Mean percent | | | | | | | | | | | |
| --- | --- | --- | --- | --- | --- | --- | --- | --- | --- | --- | --- | --- |
|  | Boys | | | | Girls | | | | Both | | | |
| **Location** | 2000 | 2019 | AAPC | *P*-Value | 2000 | 2019 | AAPC | *P*-Value | 2000 | 2019 | AAPC | *P*-Value |
|  | **Stunting** | | | | | | | | | | | |
| Anhui |  |  |  |  |  |  |  |  |  |  |  |  |
| Anqing | 21 (14 to 28 ) | 12 (2 to 34 ) | -2.32 (-3.02 to -1.61 ) | <.001 | 20 (13 to 27 ) | 11 (1 to 32 ) | -2.39 (-3.11 to -1.67 ) | <.001 | 20 (14 to 27 ) | 11 (1 to 33 ) | -2.35 (-3.06 to -1.64 ) | <.001 |
| Bengbu | 15 (11 to 20 ) | 12 (2 to 31 ) | -1.59 (-2.86 to -0.31 ) | 0.015 | 15 (11 to 19 ) | 11 (2 to 30 ) | -1.67 (-2.69 to -0.64 ) | 0.002 | 15 (11 to 20 ) | 11 (2 to 31 ) | -1.63 (-2.90 to -0.34 ) | 0.013 |
| Bozhou | 19 (13 to 24 ) | 13 (2 to 34 ) | -1.60 (-3.96 to 0.83 ) | 0.194 | 18 (13 to 24 ) | 13 (2 to 32 ) | -2.36 (-4.48 to -0.19 ) | 0.033 | 18 (13 to 24 ) | 13 (2 to 33 ) | -1.64 (-4.03 to 0.82 ) | 0.189 |
| Chaohu | 18 (13 to 25 ) | 12 (2 to 33 ) | -1.93 (-2.89 to -0.97 ) | <.001 | 17 (12 to 24 ) | 11 (1 to 31 ) | -2.01 (-2.73 to -1.29 ) | <.001 | 18 (13 to 24 ) | 11 (1 to 32 ) | -1.97 (-2.69 to -1.25 ) | <.001 |
| Chizhou | 20 (14 to 27 ) | 12 (1 to 34 ) | -2.08 (-2.91 to -1.25 ) | <.001 | 20 (13 to 26 ) | 11 (1 to 32 ) | -2.16 (-3.00 to -1.31 ) | <.001 | 20 (14 to 27 ) | 12 (1 to 33 ) | -2.12 (-2.95 to -1.28 ) | <.001 |
| Chuzhou | 16 (12 to 21 ) | 12 (2 to 34 ) | -1.34 (-2.73 to 0.06 ) | 0.060 | 16 (12 to 20 ) | 12 (2 to 32 ) | -1.41 (-2.77 to -0.04 ) | 0.044 | 16 (12 to 21 ) | 12 (2 to 33 ) | -1.38 (-2.75 to 0.02 ) | 0.053 |
| Fuyang | 22 (15 to 28 ) | 14 (2 to 36 ) | -2.61 (-3.60 to -1.61 ) | <.001 | 21 (15 to 27 ) | 14 (2 to 34 ) | -2.68 (-3.67 to -1.68 ) | <.001 | 22 (15 to 28 ) | 14 (2 to 35 ) | -2.64 (-3.63 to -1.64 ) | <.001 |
| Hefei | 15 (11 to 21 ) | 11 (2 to 30 ) | -1.58 (-3.10 to -0.03 ) | 0.046 | 15 (11 to 20 ) | 10 (1 to 29 ) | -1.65 (-3.13 to -0.13 ) | 0.033 | 15 (11 to 21 ) | 11 (1 to 29 ) | -1.61 (-3.12 to -0.08 ) | 0.040 |
| Huaibei | 14 (10 to 18 ) | 11 (2 to 30 ) | -1.29 (-4.04 to 1.54 ) | 0.368 | 13 (10 to 18 ) | 10 (1 to 28 ) | -1.38 (-4.15 to 1.46 ) | 0.337 | 14 (10 to 18 ) | 10 (1 to 29 ) | -1.33 (-4.09 to 1.50 ) | 0.353 |
| Huainan | 18 (13 to 23 ) | 12 (2 to 32 ) | -1.98 (-2.75 to -1.21 ) | <.001 | 17 (13 to 23 ) | 12 (2 to 31 ) | -2.06 (-2.82 to -1.29 ) | <.001 | 17 (13 to 23 ) | 12 (2 to 31 ) | -2.02 (-2.78 to -1.25 ) | <.001 |
| Huangshan | 19 (13 to 27 ) | 12 (1 to 35 ) | -1.73 (-4.63 to 1.24 ) | 0.251 | 19 (12 to 26 ) | 12 (1 to 33 ) | -1.80 (-4.67 to 1.16 ) | 0.231 | 19 (13 to 27 ) | 12 (1 to 34 ) | -1.76 (-4.64 to 1.21 ) | 0.242 |
| Lu'an | 21 (15 to 28 ) | 13 (2 to 35 ) | -2.20 (-2.83 to -1.57 ) | <.001 | 20 (14 to 27 ) | 13 (2 to 33 ) | -2.28 (-2.90 to -1.64 ) | <.001 | 21 (15 to 27 ) | 13 (2 to 34 ) | -2.24 (-2.86 to -1.60 ) | <.001 |
| Ma'anshan | 14 (10 to 19 ) | 10 (1 to 30 ) | -1.44 (-3.13 to 0.28 ) | 0.100 | 14 (10 to 18 ) | 9 (1 to 29 ) | -1.51 (-3.17 to 0.18 ) | 0.080 | 14 (10 to 19 ) | 10 (1 to 29 ) | -1.47 (-3.15 to 0.24 ) | 0.091 |
| Suzhou | 14 (10 to 18 ) | 11 (1 to 30 ) | -1.25 (-3.56 to 1.12 ) | 0.298 | 13 (10 to 17 ) | 10 (1 to 29 ) | -1.34 (-3.64 to 1.02 ) | 0.263 | 13 (10 to 17 ) | 11 (1 to 30 ) | -1.29 (-3.60 to 1.07 ) | 0.281 |
| Tongling | 16 (11 to 22 ) | 10 (1 to 30 ) | -1.72 (-2.63 to -0.79 ) | <.001 | 16 (11 to 22 ) | 9 (1 to 29 ) | -1.79 (-2.73 to -0.85 ) | <.001 | 16 (11 to 22 ) | 10 (1 to 29 ) | -1.75 (-2.68 to -0.82 ) | <.001 |
| Wuhu | 16 (11 to 21 ) | 10 (1 to 31 ) | -1.62 (-3.00 to -0.21 ) | 0.024 | 15 (11 to 21 ) | 10 (1 to 30 ) | -1.68 (-3.06 to -0.28 ) | 0.019 | 16 (11 to 21 ) | 10 (1 to 30 ) | -1.65 (-3.03 to -0.25 ) | 0.021 |
| Xuancheng | 17 (12 to 23 ) | 11 (1 to 34 ) | -1.38 (-3.43 to 0.71 ) | 0.194 | 16 (11 to 23 ) | 11 (1 to 32 ) | -1.75 (-4.70 to 1.29 ) | 0.257 | 17 (12 to 23 ) | 11 (1 to 33 ) | -1.72 (-4.65 to 1.30 ) | 0.262 |
| Beijing |  |  |  |  |  |  |  |  |  |  |  |  |
| Beijing | 9 (7 to 12 ) | 8 (1 to 24 ) | -0.95 (-1.07 to -0.83 ) | <.001 | 9 (7 to 12 ) | 7 (1 to 23 ) | -0.99 (-1.15 to -0.82 ) | <.001 | 9 (7 to 12 ) | 8 (1 to 24 ) | -0.97 (-1.09 to -0.84 ) | <.001 |
| Chongqing |  |  |  |  |  |  |  |  |  |  |  |  |
| Chongqing | 17 (13 to 22 ) | 11 (2 to 28 ) | -2.36 (-2.43 to -2.29 ) | <.001 | 17 (12 to 21 ) | 11 (2 to 26 ) | -2.45 (-2.54 to -2.37 ) | <.001 | 17 (13 to 21 ) | 11 (2 to 27 ) | -2.40 (-2.47 to -2.33 ) | <.001 |
| Fujian |  |  |  |  |  |  |  |  |  |  |  |  |
| Fuzhou | 10 (5 to 19 ) | 6 (1 to 22 ) | -2.43 (-3.45 to -1.39 ) | <.001 | 10 (5 to 18 ) | 6 (1 to 20 ) | -2.48 (-3.53 to -1.43 ) | <.001 | 10 (5 to 18 ) | 6 (1 to 21 ) | -2.45 (-3.49 to -1.41 ) | <.001 |
| Longyan | 23 (15 to 32 ) | 16 (2 to 39 ) | -2.19 (-5.55 to 1.30 ) | 0.215 | 22 (15 to 31 ) | 15 (2 to 37 ) | -2.26 (-5.62 to 1.23 ) | 0.202 | 23 (15 to 32 ) | 16 (2 to 39 ) | -2.22 (-5.58 to 1.27 ) | 0.209 |
| Nanping | 22 (15 to 31 ) | 16 (2 to 39 ) | -1.40 (-5.97 to 3.41 ) | 0.563 | 22 (14 to 30 ) | 16 (2 to 37 ) | -1.47 (-6.22 to 3.52 ) | 0.557 | 22 (14 to 30 ) | 16 (2 to 38 ) | -1.43 (-6.08 to 3.46 ) | 0.560 |
| Ningde | 17 (9 to 26 ) | 11 (1 to 31 ) | -2.07 (-3.47 to -0.65 ) | 0.004 | 16 (9 to 26 ) | 11 (1 to 30 ) | -2.14 (-3.59 to -0.67 ) | 0.005 | 16 (9 to 26 ) | 11 (1 to 31 ) | -2.10 (-3.52 to -0.66 ) | 0.004 |
| Putian | 12 (6 to 21 ) | 7 (1 to 24 ) | -2.21 (-4.76 to 0.41 ) | 0.098 | 11 (6 to 20 ) | 7 (1 to 23 ) | -2.25 (-4.85 to 0.42 ) | 0.098 | 12 (6 to 21 ) | 7 (1 to 24 ) | -2.23 (-4.80 to 0.42 ) | 0.098 |
| Quanzhou | 11 (6 to 20 ) | 7 (1 to 25 ) | -1.64 (-3.47 to 0.22 ) | 0.084 | 11 (6 to 19 ) | 7 (1 to 23 ) | -1.68 (-3.57 to 0.24 ) | 0.086 | 11 (6 to 19 ) | 7 (1 to 24 ) | -1.66 (-3.51 to 0.23 ) | 0.085 |
| Sanming | 22 (15 to 31 ) | 16 (2 to 39 ) | -1.93 (-4.25 to 0.45 ) | 0.111 | 21 (14 to 30 ) | 15 (2 to 37 ) | -1.99 (-4.31 to 0.39 ) | 0.100 | 22 (14 to 31 ) | 16 (2 to 38 ) | -1.95 (-4.27 to 0.42 ) | 0.106 |
| Xiamen | 8 (3 to 14 ) | 10 (2 to 25 ) | 1.58 (-5.05 to 8.66 ) | 0.649 | 7 (3 to 14 ) | 10 (2 to 23 ) | 1.50 (-5.10 to 8.57 ) | 0.664 | 7 (3 to 14 ) | 10 (2 to 24 ) | 1.54 (-5.07 to 8.62 ) | 0.656 |
| Zhangzhou | 16 (8 to 25 ) | 11 (1 to 31 ) | -1.53 (-3.77 to 0.77 ) | 0.190 | 15 (8 to 25 ) | 10 (1 to 30 ) | -1.56 (-3.88 to 0.81 ) | 0.196 | 15 (8 to 25 ) | 10 (1 to 31 ) | -1.54 (-3.82 to 0.79 ) | 0.192 |
| Gansu |  |  |  |  |  |  |  |  |  |  |  |  |
| Baiyin | 20 (12 to 28 ) | 13 (2 to 33 ) | -2.35 (-3.48 to -1.20 ) | <.001 | 19 (12 to 27 ) | 12 (2 to 31 ) | -2.40 (-3.55 to -1.24 ) | <.001 | 19 (12 to 27 ) | 12 (2 to 32 ) | -2.37 (-3.51 to -1.22 ) | <.001 |
| Dingxi | 24 (16 to 32 ) | 16 (3 to 37 ) | -2.23 (-2.53 to -1.92 ) | <.001 | 23 (16 to 31 ) | 15 (3 to 36 ) | -2.28 (-2.57 to -1.98 ) | <.001 | 23 (16 to 31 ) | 15 (3 to 37 ) | -2.25 (-2.55 to -1.95 ) | <.001 |
| Gannan Tibetan | 26 (17 to 34 ) | 18 (4 to 40 ) | -1.96 (-2.49 to -1.42 ) | <.001 | 25 (17 to 33 ) | 17 (3 to 38 ) | -2.04 (-3.49 to -0.58 ) | 0.007 | 25 (17 to 33 ) | 18 (3 to 39 ) | -1.99 (-2.53 to -1.46 ) | <.001 |
| Jiayuguan | 17 (10 to 26 ) | 9 (1 to 27 ) | -3.62 (-8.08 to 1.07 ) | 0.128 | 17 (9 to 25 ) | 8 (1 to 26 ) | -3.71 (-8.16 to 0.96 ) | 0.118 | 17 (9 to 25 ) | 8 (1 to 26 ) | -3.66 (-8.12 to 1.02 ) | 0.124 |
| Jinchang | 22 (14 to 30 ) | 13 (2 to 35 ) | -2.58 (-5.04 to -0.05 ) | 0.046 | 21 (14 to 29 ) | 13 (2 to 33 ) | -2.64 (-5.27 to 0.05 ) | 0.054 | 22 (14 to 30 ) | 13 (2 to 34 ) | -2.61 (-5.14 to -0.01 ) | 0.049 |
| Jiuquan | 21 (13 to 29 ) | 13 (2 to 34 ) | -2.61 (-8.23 to 3.35 ) | 0.383 | 21 (13 to 28 ) | 12 (2 to 32 ) | -2.71 (-8.32 to 3.25 ) | 0.366 | 21 (13 to 28 ) | 12 (2 to 33 ) | -2.65 (-8.27 to 3.31 ) | 0.375 |
| Lanzhou | 19 (11 to 27 ) | 11 (2 to 29 ) | -2.75 (-3.58 to -1.92 ) | <.001 | 18 (11 to 26 ) | 10 (2 to 28 ) | -2.84 (-3.67 to -1.99 ) | <.001 | 18 (11 to 27 ) | 11 (2 to 29 ) | -2.79 (-3.62 to -1.95 ) | <.001 |
| Linxia Hui | 26 (18 to 34 ) | 18 (4 to 40 ) | -2.02 (-4.63 to 0.65 ) | 0.137 | 25 (17 to 33 ) | 17 (3 to 38 ) | -2.33 (-4.31 to -0.31 ) | 0.024 | 26 (18 to 33 ) | 17 (3 to 39 ) | -2.30 (-4.29 to -0.27 ) | 0.026 |
| Longnan | 22 (15 to 30 ) | 15 (3 to 36 ) | -2.02 (-4.29 to 0.31 ) | 0.089 | 22 (14 to 29 ) | 15 (3 to 35 ) | -2.12 (-4.39 to 0.21 ) | 0.074 | 22 (15 to 29 ) | 15 (3 to 36 ) | -2.06 (-4.34 to 0.26 ) | 0.082 |
| Pingliang | 20 (13 to 28 ) | 14 (3 to 34 ) | -1.75 (-4.26 to 0.83 ) | 0.183 | 20 (13 to 27 ) | 13 (2 to 32 ) | -1.79 (-4.33 to 0.82 ) | 0.176 | 20 (13 to 27 ) | 14 (2 to 33 ) | -1.77 (-4.29 to 0.82 ) | 0.179 |
| Qingyang | 18 (11 to 25 ) | 13 (2 to 33 ) | -1.26 (-4.84 to 2.45 ) | 0.501 | 17 (11 to 24 ) | 12 (2 to 31 ) | -1.31 (-4.89 to 2.39 ) | 0.482 | 17 (11 to 25 ) | 13 (2 to 32 ) | -1.28 (-4.86 to 2.42 ) | 0.492 |
| Tianshui | 23 (15 to 30 ) | 15 (3 to 36 ) | -2.06 (-3.40 to -0.70 ) | 0.003 | 22 (15 to 29 ) | 14 (2 to 34 ) | -2.11 (-3.48 to -0.71 ) | 0.003 | 22 (15 to 30 ) | 14 (3 to 35 ) | -2.08 (-3.44 to -0.71 ) | 0.003 |
| Wuwei | 24 (16 to 32 ) | 16 (3 to 38 ) | -2.21 (-4.35 to -0.01 ) | 0.049 | 23 (15 to 31 ) | 15 (2 to 36 ) | -2.27 (-4.60 to 0.12 ) | 0.063 | 23 (16 to 31 ) | 15 (3 to 37 ) | -2.24 (-4.47 to 0.05 ) | 0.055 |
| Zhangye | 24 (15 to 31 ) | 15 (3 to 37 ) | -2.36 (-5.74 to 1.15 ) | 0.184 | 23 (15 to 30 ) | 14 (2 to 35 ) | -2.43 (-5.98 to 1.25 ) | 0.192 | 23 (15 to 31 ) | 14 (2 to 36 ) | -2.39 (-5.85 to 1.19 ) | 0.188 |
| Guangdong |  |  |  |  |  |  |  |  |  |  |  |  |
| Chaozhou | 16 (9 to 26 ) | 9 (1 to 26 ) | -2.36 (-5.07 to 0.43 ) | 0.096 | 16 (9 to 25 ) | 9 (1 to 25 ) | -2.42 (-5.10 to 0.34 ) | 0.085 | 16 (9 to 25 ) | 9 (1 to 25 ) | -2.39 (-5.08 to 0.39 ) | 0.091 |
| Dongguan | 8 (5 to 12 ) | 7 (1 to 23 ) | -0.03 (-3.53 to 3.61 ) | 0.988 | 8 (4 to 12 ) | 7 (1 to 22 ) | -0.08 (-3.65 to 3.62 ) | 0.966 | 8 (4 to 12 ) | 7 (1 to 23 ) | -0.05 (-3.58 to 3.61 ) | 0.978 |
| Foshan | 10 (6 to 14 ) | 8 (1 to 24 ) | -1.00 (-3.29 to 1.34 ) | 0.397 | 10 (6 to 14 ) | 7 (1 to 23 ) | -1.05 (-3.39 to 1.34 ) | 0.386 | 10 (6 to 14 ) | 7 (1 to 24 ) | -1.03 (-3.33 to 1.34 ) | 0.392 |
| Guangzhou | 11 (7 to 15 ) | 10 (1 to 27 ) | -0.29 (-0.85 to 0.27 ) | 0.308 | 10 (7 to 15 ) | 10 (1 to 26 ) | -0.37 (-0.86 to 0.13 ) | 0.146 | 11 (7 to 15 ) | 10 (1 to 26 ) | -0.35 (-0.85 to 0.16 ) | 0.183 |
| Heyuan | 20 (14 to 28 ) | 14 (2 to 32 ) | -1.95 (-5.87 to 2.14 ) | 0.346 | 19 (13 to 27 ) | 13 (2 to 30 ) | -2.01 (-6.10 to 2.25 ) | 0.349 | 20 (13 to 27 ) | 13 (2 to 31 ) | -1.98 (-5.97 to 2.19 ) | 0.347 |
| Huizhou | 13 (8 to 19 ) | 10 (1 to 27 ) | -1.30 (-1.97 to -0.62 ) | <.001 | 13 (8 to 18 ) | 9 (1 to 25 ) | -1.34 (-2.06 to -0.62 ) | <.001 | 13 (8 to 19 ) | 10 (1 to 26 ) | -1.32 (-2.01 to -0.62 ) | <.001 |
| Jiangmen | 11 (7 to 15 ) | 9 (1 to 26 ) | -1.40 (-3.45 to 0.70 ) | 0.189 | 11 (7 to 15 ) | 8 (1 to 24 ) | -1.49 (-3.54 to 0.62 ) | 0.165 | 11 (7 to 15 ) | 8 (1 to 25 ) | -1.44 (-3.49 to 0.66 ) | 0.177 |
| Jieyang | 16 (9 to 26 ) | 9 (1 to 27 ) | -2.72 (-5.09 to -0.28 ) | 0.029 | 16 (9 to 25 ) | 9 (1 to 26 ) | -2.77 (-5.17 to -0.30 ) | 0.028 | 16 (9 to 25 ) | 9 (1 to 26 ) | -2.74 (-5.13 to -0.29 ) | 0.028 |
| Maoming | 17 (14 to 20 ) | 11 (2 to 28 ) | -2.14 (-4.67 to 0.45 ) | 0.105 | 16 (13 to 20 ) | 10 (1 to 26 ) | -2.18 (-4.73 to 0.44 ) | 0.103 | 17 (13 to 20 ) | 11 (2 to 27 ) | -2.16 (-4.69 to 0.45 ) | 0.104 |
| Meizhou | 20 (13 to 28 ) | 12 (2 to 31 ) | -2.52 (-4.59 to -0.40 ) | 0.020 | 19 (12 to 27 ) | 12 (2 to 29 ) | -2.59 (-4.64 to -0.49 ) | 0.016 | 20 (12 to 28 ) | 12 (2 to 30 ) | -2.55 (-4.62 to -0.44 ) | 0.018 |
| Qingyuan | 20 (16 to 25 ) | 15 (3 to 33 ) | -1.52 (-5.54 to 2.66 ) | 0.470 | 20 (15 to 24 ) | 14 (2 to 31 ) | -1.60 (-5.81 to 2.80 ) | 0.470 | 20 (16 to 25 ) | 15 (3 to 32 ) | -1.56 (-5.66 to 2.72 ) | 0.469 |
| Shantou | 15 (8 to 25 ) | 8 (1 to 25 ) | -2.74 (-4.74 to -0.71 ) | 0.008 | 15 (8 to 24 ) | 8 (1 to 24 ) | -2.81 (-4.77 to -0.80 ) | 0.006 | 15 (8 to 24 ) | 8 (1 to 24 ) | -2.77 (-4.75 to -0.75 ) | 0.007 |
| Shanwei | 14 (8 to 22 ) | 9 (1 to 26 ) | -1.94 (-4.61 to 0.81 ) | 0.165 | 13 (7 to 21 ) | 9 (1 to 24 ) | -1.98 (-4.70 to 0.82 ) | 0.164 | 13 (7 to 21 ) | 9 (1 to 25 ) | -1.96 (-4.65 to 0.81 ) | 0.165 |
| Shaoguan | 20 (15 to 26 ) | 15 (3 to 32 ) | -1.66 (-6.23 to 3.14 ) | 0.491 | 20 (15 to 25 ) | 14 (2 to 31 ) | -1.73 (-6.43 to 3.21 ) | 0.486 | 20 (15 to 25 ) | 15 (3 to 32 ) | -1.69 (-6.32 to 3.17 ) | 0.488 |
| Shenzhen | 5 (3 to 9 ) | 5 (0 to 19 ) | 1.10 (-3.51 to 5.93 ) | 0.647 | 5 (3 to 8 ) | 5 (0 to 18 ) | 1.05 (-3.62 to 5.94 ) | 0.665 | 5 (3 to 8 ) | 5 (0 to 18 ) | 1.07 (-3.56 to 5.93 ) | 0.655 |
| Yangjiang | 16 (12 to 20 ) | 11 (2 to 29 ) | -2.10 (-5.09 to 0.98 ) | 0.180 | 15 (11 to 19 ) | 11 (2 to 27 ) | -2.18 (-5.11 to 0.84 ) | 0.156 | 16 (12 to 19 ) | 11 (2 to 28 ) | -2.14 (-5.10 to 0.92 ) | 0.169 |
| Yunfu | 17 (13 to 21 ) | 12 (2 to 30 ) | -2.17 (-3.94 to -0.37 ) | 0.018 | 16 (12 to 20 ) | 11 (2 to 29 ) | -2.25 (-3.99 to -0.46 ) | 0.014 | 16 (13 to 20 ) | 11 (2 to 29 ) | -2.21 (-3.97 to -0.41 ) | 0.016 |
| Zhanjiang | 13 (10 to 15 ) | 8 (1 to 23 ) | -2.73 (-6.16 to 0.84 ) | 0.132 | 12 (10 to 15 ) | 7 (1 to 22 ) | -2.92 (-5.18 to -0.62 ) | 0.013 | 13 (10 to 15 ) | 7 (1 to 22 ) | -2.91 (-5.17 to -0.59 ) | 0.014 |
| Zhaoqing | 19 (14 to 22 ) | 13 (2 to 31 ) | -2.06 (-3.82 to -0.26 ) | 0.025 | 18 (14 to 22 ) | 12 (2 to 30 ) | -2.12 (-3.85 to -0.35 ) | 0.019 | 18 (14 to 22 ) | 13 (2 to 31 ) | -2.09 (-3.83 to -0.31 ) | 0.022 |
| Zhongshan | 8 (5 to 12 ) | 7 (1 to 23 ) | -0.50 (-4.22 to 3.35 ) | 0.795 | 8 (5 to 12 ) | 7 (1 to 22 ) | -0.55 (-4.32 to 3.38 ) | 0.782 | 8 (5 to 12 ) | 7 (1 to 23 ) | -0.52 (-4.26 to 3.36 ) | 0.788 |
| Zhuhai | 7 (4 to 11 ) | 6 (1 to 22 ) | 0.18 (-4.83 to 5.46 ) | 0.944 | 7 (4 to 11 ) | 6 (1 to 21 ) | 0.14 (-4.95 to 5.50 ) | 0.958 | 7 (4 to 11 ) | 6 (1 to 21 ) | 0.16 (-4.88 to 5.48 ) | 0.951 |
| Guangxi |  |  |  |  |  |  |  |  |  |  |  |  |
| Baise | 29 (24 to 34 ) | 17 (3 to 42 ) | -2.96 (-5.41 to -0.45 ) | 0.021 | 28 (23 to 33 ) | 16 (2 to 40 ) | -3.00 (-5.42 to -0.52 ) | 0.018 | 29 (24 to 34 ) | 16 (3 to 41 ) | -2.98 (-5.42 to -0.48 ) | 0.020 |
| Beihai | 20 (17 to 25 ) | 12 (1 to 38 ) | -2.34 (-8.21 to 3.91 ) | 0.455 | 20 (16 to 24 ) | 12 (1 to 36 ) | -2.40 (-8.30 to 3.87 ) | 0.444 | 20 (17 to 24 ) | 12 (1 to 37 ) | -2.37 (-8.25 to 3.89 ) | 0.450 |
| Chongzuo | 25 (19 to 30 ) | 15 (2 to 42 ) | -2.67 (-6.05 to 0.84 ) | 0.135 | 24 (19 to 29 ) | 14 (2 to 40 ) | -2.76 (-6.15 to 0.75 ) | 0.122 | 24 (19 to 30 ) | 14 (2 to 41 ) | -2.71 (-6.09 to 0.80 ) | 0.129 |
| Fangchenggang | 25 (20 to 31 ) | 15 (2 to 43 ) | -2.83 (-3.66 to -1.99 ) | <.001 | 24 (20 to 30 ) | 15 (2 to 41 ) | -2.90 (-3.72 to -2.08 ) | <.001 | 25 (20 to 30 ) | 15 (2 to 42 ) | -2.86 (-3.68 to -2.03 ) | <.001 |
| Guigang | 27 (23 to 32 ) | 18 (3 to 46 ) | -2.09 (-3.35 to -0.82 ) | 0.001 | 26 (22 to 30 ) | 17 (3 to 44 ) | -2.14 (-3.41 to -0.86 ) | 0.001 | 27 (23 to 31 ) | 18 (3 to 45 ) | -2.11 (-3.38 to -0.84 ) | 0.001 |
| Guilin | 25 (21 to 29 ) | 18 (3 to 45 ) | -2.14 (-4.13 to -0.12 ) | 0.038 | 24 (20 to 28 ) | 17 (3 to 43 ) | -2.18 (-4.10 to -0.22 ) | 0.030 | 25 (21 to 29 ) | 17 (3 to 44 ) | -2.16 (-4.12 to -0.16 ) | 0.034 |
| Hechi | 29 (24 to 34 ) | 18 (3 to 45 ) | -2.80 (-4.73 to -0.82 ) | 0.006 | 28 (24 to 33 ) | 17 (3 to 43 ) | -2.46 (-5.95 to 1.16 ) | 0.181 | 28 (24 to 33 ) | 18 (3 to 44 ) | -2.41 (-5.98 to 1.30 ) | 0.199 |
| Hezhou | 29 (24 to 35 ) | 21 (4 to 50 ) | -1.75 (-2.54 to -0.96 ) | <.001 | 29 (24 to 33 ) | 20 (4 to 48 ) | -1.82 (-2.62 to -1.02 ) | <.001 | 29 (24 to 34 ) | 20 (4 to 49 ) | -1.78 (-2.57 to -0.99 ) | <.001 |
| Laibin | 24 (20 to 28 ) | 17 (3 to 44 ) | -1.76 (-3.79 to 0.31 ) | 0.096 | 23 (19 to 27 ) | 16 (2 to 42 ) | -2.07 (-3.48 to -0.64 ) | 0.005 | 24 (20 to 28 ) | 17 (2 to 43 ) | -2.05 (-3.47 to -0.61 ) | 0.005 |
| Liuzhou | 25 (21 to 29 ) | 16 (3 to 42 ) | -2.18 (-3.43 to -0.92 ) | 0.001 | 24 (20 to 28 ) | 16 (3 to 40 ) | -2.27 (-3.52 to -0.99 ) | 0.001 | 24 (21 to 28 ) | 16 (3 to 41 ) | -2.22 (-3.47 to -0.95 ) | 0.001 |
| Nanning | 24 (20 to 27 ) | 15 (2 to 41 ) | -2.46 (-3.72 to -1.19 ) | <.001 | 23 (19 to 26 ) | 14 (2 to 39 ) | -2.52 (-3.72 to -1.29 ) | <.001 | 23 (20 to 27 ) | 15 (2 to 40 ) | -2.49 (-3.72 to -1.23 ) | <.001 |
| Qinzhou | 26 (22 to 31 ) | 17 (2 to 45 ) | -2.39 (-3.68 to -1.09 ) | <.001 | 26 (22 to 30 ) | 16 (2 to 43 ) | -2.44 (-3.72 to -1.15 ) | <.001 | 26 (22 to 31 ) | 16 (2 to 44 ) | -2.42 (-3.70 to -1.12 ) | <.001 |
| Wuzhou | 27 (23 to 32 ) | 19 (3 to 47 ) | -1.63 (-2.51 to -0.74 ) | <.001 | 27 (22 to 31 ) | 18 (3 to 45 ) | -1.74 (-3.11 to -0.35 ) | 0.014 | 27 (22 to 32 ) | 19 (3 to 46 ) | -1.71 (-3.06 to -0.34 ) | 0.015 |
| Yulin | 28 (24 to 33 ) | 19 (3 to 48 ) | -1.98 (-3.12 to -0.81 ) | 0.001 | 27 (23 to 31 ) | 18 (3 to 45 ) | -2.24 (-3.28 to -1.20 ) | <.001 | 28 (23 to 32 ) | 18 (3 to 47 ) | -2.19 (-3.24 to -1.13 ) | <.001 |
| Guizhou |  |  |  |  |  |  |  |  |  |  |  |  |
| Anshun | 43 (37 to 48 ) | 28 (5 to 69 ) | -2.30 (-3.27 to -1.31 ) | <.001 | 41 (36 to 46 ) | 27 (5 to 65 ) | -2.33 (-3.29 to -1.37 ) | <.001 | 42 (36 to 47 ) | 27 (5 to 67 ) | -2.31 (-3.28 to -1.34 ) | <.001 |
| Bijie | 43 (37 to 49 ) | 27 (5 to 66 ) | -2.31 (-4.92 to 0.37 ) | 0.090 | 41 (35 to 47 ) | 26 (5 to 63 ) | -2.40 (-4.86 to 0.13 ) | 0.063 | 42 (36 to 48 ) | 26 (5 to 65 ) | -2.35 (-4.98 to 0.36 ) | 0.088 |
| Guiyang | 34 (29 to 38 ) | 23 (4 to 59 ) | -2.08 (-3.18 to -0.96 ) | <.001 | 33 (28 to 37 ) | 21 (3 to 56 ) | -2.15 (-3.79 to -0.48 ) | 0.012 | 33 (28 to 38 ) | 22 (4 to 58 ) | -2.11 (-3.21 to -0.98 ) | <.001 |
| Liupanshui | 43 (36 to 50 ) | 27 (5 to 66 ) | -2.42 (-4.11 to -0.71 ) | 0.006 | 41 (35 to 49 ) | 26 (5 to 63 ) | -2.46 (-4.11 to -0.78 ) | 0.004 | 42 (36 to 49 ) | 27 (5 to 65 ) | -2.44 (-4.11 to -0.74 ) | 0.005 |
| Qiandongnan Miao and Dong | 40 (35 to 45 ) | 31 (5 to 74 ) | -1.18 (-3.89 to 1.60 ) | 0.401 | 39 (34 to 44 ) | 29 (5 to 71 ) | -1.24 (-3.96 to 1.55 ) | 0.380 | 40 (34 to 44 ) | 30 (5 to 73 ) | -1.21 (-3.92 to 1.58 ) | 0.391 |
| Qiannan Buyei and Miao | 40 (35 to 45 ) | 29 (5 to 71 ) | -1.70 (-3.67 to 0.32 ) | 0.098 | 39 (34 to 43 ) | 28 (5 to 67 ) | -1.78 (-3.79 to 0.27 ) | 0.088 | 39 (34 to 44 ) | 29 (5 to 69 ) | -1.73 (-3.72 to 0.29 ) | 0.093 |
| Qianxinan Buyei and Miao | 41 (34 to 48 ) | 27 (5 to 68 ) | -2.06 (-3.39 to -0.71 ) | 0.003 | 40 (33 to 47 ) | 26 (4 to 65 ) | -2.10 (-3.43 to -0.74 ) | 0.002 | 41 (34 to 48 ) | 27 (4 to 67 ) | -2.08 (-3.41 to -0.73 ) | 0.003 |
| Tongren | 36 (30 to 42 ) | 26 (4 to 67 ) | -2.19 (-4.19 to -0.15 ) | 0.036 | 35 (29 to 41 ) | 25 (4 to 64 ) | -2.23 (-4.21 to -0.20 ) | 0.031 | 35 (29 to 41 ) | 25 (4 to 66 ) | -2.21 (-4.20 to -0.17 ) | 0.034 |
| Zunyi | 34 (28 to 40 ) | 23 (4 to 60 ) | -2.19 (-4.05 to -0.30 ) | 0.023 | 33 (27 to 39 ) | 21 (3 to 57 ) | -2.27 (-4.11 to -0.39 ) | 0.018 | 34 (27 to 39 ) | 22 (4 to 58 ) | -2.23 (-4.08 to -0.34 ) | 0.021 |
| Hainan |  |  |  |  |  |  |  |  |  |  |  |  |
| Haikou | 14 (11 to 17 ) | 9 (1 to 31 ) | -2.22 (-4.41 to 0.03 ) | 0.053 | 13 (11 to 16 ) | 8 (1 to 30 ) | -2.27 (-4.51 to 0.02 ) | 0.052 | 13 (11 to 17 ) | 8 (1 to 30 ) | -2.24 (-4.46 to 0.02 ) | 0.052 |
| Hainan | 19 (16 to 23 ) | 14 (2 to 40 ) | -1.87 (-2.47 to -1.25 ) | <.001 | 18 (15 to 22 ) | 13 (1 to 38 ) | -1.93 (-2.50 to -1.35 ) | <.001 | 19 (16 to 22 ) | 13 (2 to 39 ) | -1.89 (-2.49 to -1.30 ) | <.001 |
| Sanya | 12 (9 to 16 ) | 9 (1 to 31 ) | -1.20 (-3.14 to 0.79 ) | 0.235 | 12 (9 to 15 ) | 8 (1 to 29 ) | -1.42 (-5.58 to 2.92 ) | 0.515 | 12 (9 to 16 ) | 9 (1 to 30 ) | -1.23 (-3.17 to 0.75 ) | 0.222 |
| Hebei |  |  |  |  |  |  |  |  |  |  |  |  |
| Baoding | 18 (14 to 21 ) | 12 (2 to 31 ) | -2.30 (-3.24 to -1.36 ) | <.001 | 17 (14 to 21 ) | 11 (2 to 29 ) | -2.35 (-3.23 to -1.46 ) | <.001 | 17 (14 to 21 ) | 12 (2 to 30 ) | -2.32 (-3.24 to -1.40 ) | <.001 |
| Cangzhou | 13 (10 to 15 ) | 9 (1 to 27 ) | -1.50 (-3.15 to 0.17 ) | 0.079 | 12 (10 to 15 ) | 9 (1 to 25 ) | -1.56 (-3.20 to 0.11 ) | 0.067 | 12 (10 to 15 ) | 9 (1 to 26 ) | -1.53 (-3.18 to 0.14 ) | 0.073 |
| Chengde | 17 (13 to 22 ) | 11 (2 to 32 ) | -2.69 (-5.41 to 0.12 ) | 0.060 | 17 (13 to 21 ) | 11 (2 to 31 ) | -2.74 (-5.42 to 0.03 ) | 0.052 | 17 (13 to 22 ) | 11 (2 to 31 ) | -2.71 (-5.42 to 0.08 ) | 0.057 |
| Handan | 16 (12 to 20 ) | 11 (2 to 29 ) | -1.80 (-4.35 to 0.81 ) | 0.174 | 16 (12 to 19 ) | 11 (2 to 27 ) | -1.88 (-4.42 to 0.74 ) | 0.158 | 16 (12 to 20 ) | 11 (2 to 28 ) | -1.84 (-4.38 to 0.77 ) | 0.166 |
| Hengshui | 14 (11 to 17 ) | 11 (2 to 29 ) | -1.02 (-2.76 to 0.75 ) | 0.258 | 13 (11 to 16 ) | 10 (2 to 27 ) | -1.10 (-2.81 to 0.64 ) | 0.215 | 13 (11 to 16 ) | 10 (2 to 28 ) | -1.04 (-2.78 to 0.72 ) | 0.245 |
| Langfang | 13 (10 to 16 ) | 10 (1 to 29 ) | -1.85 (-3.27 to -0.41 ) | 0.012 | 13 (10 to 16 ) | 10 (1 to 27 ) | -1.89 (-3.26 to -0.49 ) | 0.008 | 13 (10 to 16 ) | 10 (1 to 28 ) | -1.87 (-3.27 to -0.45 ) | 0.010 |
| Qinhuangdao | 15 (11 to 18 ) | 12 (2 to 33 ) | -0.99 (-2.76 to 0.82 ) | 0.281 | 14 (11 to 18 ) | 12 (2 to 31 ) | -1.43 (-2.71 to -0.14 ) | 0.030 | 15 (11 to 18 ) | 12 (2 to 32 ) | -1.03 (-2.82 to 0.80 ) | 0.268 |
| Shijiazhuang | 17 (13 to 21 ) | 12 (2 to 30 ) | -1.80 (-2.98 to -0.61 ) | 0.003 | 16 (13 to 20 ) | 11 (2 to 29 ) | -1.98 (-2.43 to -1.53 ) | <.001 | 17 (13 to 20 ) | 12 (2 to 30 ) | -1.95 (-2.43 to -1.47 ) | <.001 |
| Tangshan | 15 (11 to 19 ) | 12 (2 to 32 ) | -1.30 (-2.98 to 0.41 ) | 0.137 | 14 (11 to 18 ) | 11 (2 to 30 ) | -1.67 (-2.78 to -0.55 ) | 0.004 | 15 (11 to 18 ) | 11 (2 to 31 ) | -1.65 (-2.78 to -0.50 ) | 0.005 |
| Xingtai | 15 (12 to 18 ) | 11 (2 to 29 ) | -1.32 (-3.14 to 0.53 ) | 0.160 | 14 (11 to 17 ) | 11 (2 to 28 ) | -1.40 (-3.35 to 0.59 ) | 0.168 | 15 (11 to 18 ) | 11 (2 to 29 ) | -1.36 (-3.23 to 0.55 ) | 0.162 |
| Zhangjiakou | 20 (16 to 25 ) | 12 (2 to 31 ) | -3.33 (-6.49 to -0.06 ) | 0.046 | 19 (15 to 24 ) | 11 (2 to 29 ) | -3.38 (-6.51 to -0.15 ) | 0.041 | 20 (16 to 24 ) | 11 (2 to 30 ) | -3.35 (-6.50 to -0.10 ) | 0.044 |
| Heilongjiang |  |  |  |  |  |  |  |  |  |  |  |  |
| Daqing | 17 (9 to 27 ) | 6 (1 to 19 ) | -5.16 (-5.75 to -4.56 ) | <.001 | 16 (9 to 26 ) | 6 (1 to 18 ) | -5.24 (-5.90 to -4.58 ) | <.001 | 17 (9 to 26 ) | 6 (1 to 18 ) | -5.21 (-5.87 to -4.55 ) | <.001 |
| Daxing'anling | 19 (6 to 41 ) | 7 (1 to 21 ) | -5.21 (-8.81 to -1.48 ) | 0.007 | 18 (6 to 40 ) | 6 (1 to 20 ) | -5.27 (-8.88 to -1.51 ) | 0.006 | 19 (6 to 41 ) | 7 (1 to 20 ) | -5.24 (-8.84 to -1.49 ) | 0.006 |
| Harbin | 14 (7 to 24 ) | 7 (1 to 20 ) | -3.57 (-4.03 to -3.11 ) | <.001 | 14 (7 to 23 ) | 7 (1 to 19 ) | -3.62 (-4.12 to -3.11 ) | <.001 | 14 (7 to 23 ) | 7 (1 to 20 ) | -3.59 (-4.07 to -3.11 ) | <.001 |
| Hegang | 11 (4 to 24 ) | 7 (1 to 22 ) | -2.55 (-3.36 to -1.75 ) | <.001 | 11 (4 to 24 ) | 7 (1 to 21 ) | -2.64 (-3.40 to -1.87 ) | <.001 | 11 (4 to 24 ) | 7 (1 to 21 ) | -2.59 (-3.38 to -1.80 ) | <.001 |
| Heihe | 14 (6 to 27 ) | 6 (1 to 20 ) | -4.11 (-5.50 to -2.69 ) | <.001 | 14 (6 to 26 ) | 6 (1 to 19 ) | -4.18 (-5.61 to -2.72 ) | <.001 | 14 (6 to 27 ) | 6 (1 to 19 ) | -4.14 (-5.55 to -2.70 ) | <.001 |
| Jiamusi | 12 (4 to 26 ) | 7 (1 to 23 ) | -2.67 (-3.41 to -1.93 ) | <.001 | 12 (4 to 26 ) | 7 (1 to 21 ) | -2.75 (-3.45 to -2.05 ) | <.001 | 12 (4 to 26 ) | 7 (1 to 22 ) | -2.71 (-3.43 to -1.98 ) | <.001 |
| Jixi | 12 (3 to 27 ) | 8 (1 to 23 ) | -2.30 (-2.88 to -1.72 ) | <.001 | 11 (3 to 26 ) | 7 (1 to 22 ) | -2.49 (-3.21 to -1.77 ) | <.001 | 12 (3 to 26 ) | 7 (1 to 23 ) | -2.34 (-2.92 to -1.76 ) | <.001 |
| Mudanjiang | 13 (5 to 25 ) | 8 (1 to 23 ) | -2.59 (-3.15 to -2.03 ) | <.001 | 12 (5 to 24 ) | 7 (1 to 22 ) | -2.64 (-3.15 to -2.14 ) | <.001 | 12 (5 to 24 ) | 7 (1 to 22 ) | -2.62 (-3.15 to -2.08 ) | <.001 |
| Qiqihar | 19 (10 to 31 ) | 7 (1 to 21 ) | -5.48 (-5.87 to -5.10 ) | <.001 | 18 (10 to 30 ) | 6 (1 to 20 ) | -5.69 (-6.03 to -5.34 ) | <.001 | 19 (10 to 30 ) | 6 (1 to 20 ) | -5.53 (-5.91 to -5.14 ) | <.001 |
| Qitaihe | 11 (4 to 22 ) | 7 (1 to 21 ) | -2.46 (-2.81 to -2.11 ) | <.001 | 10 (3 to 22 ) | 6 (1 to 20 ) | -2.55 (-2.90 to -2.20 ) | <.001 | 11 (4 to 22 ) | 6 (1 to 21 ) | -2.50 (-2.85 to -2.15 ) | <.001 |
| Shuangyashan | 11 (3 to 26 ) | 7 (1 to 22 ) | -2.27 (-2.68 to -1.87 ) | <.001 | 11 (3 to 25 ) | 7 (1 to 21 ) | -2.36 (-2.78 to -1.94 ) | <.001 | 11 (3 to 25 ) | 7 (1 to 22 ) | -2.31 (-2.72 to -1.90 ) | <.001 |
| Suihua | 15 (8 to 26 ) | 7 (1 to 20 ) | -4.44 (-4.71 to -4.18 ) | <.001 | 15 (8 to 25 ) | 6 (1 to 19 ) | -4.51 (-4.75 to -4.26 ) | <.001 | 15 (8 to 26 ) | 6 (1 to 20 ) | -4.47 (-4.73 to -4.22 ) | <.001 |
| Yichun | 14 (5 to 25 ) | 7 (1 to 21 ) | -3.24 (-3.92 to -2.56 ) | <.001 | 13 (5 to 24 ) | 7 (1 to 20 ) | -3.32 (-3.99 to -2.65 ) | <.001 | 13 (5 to 25 ) | 7 (1 to 21 ) | -3.28 (-3.95 to -2.60 ) | <.001 |
| Henan |  |  |  |  |  |  |  |  |  |  |  |  |
| Anyang | 22 (17 to 28 ) | 16 (2 to 39 ) | -1.78 (-5.30 to 1.87 ) | 0.335 | 21 (16 to 27 ) | 15 (2 to 37 ) | -2.16 (-3.98 to -0.31 ) | 0.022 | 22 (16 to 27 ) | 15 (2 to 38 ) | -2.13 (-3.96 to -0.27 ) | 0.025 |
| Hebi | 21 (16 to 27 ) | 15 (2 to 38 ) | -2.05 (-3.02 to -1.07 ) | <.001 | 21 (15 to 26 ) | 15 (2 to 36 ) | -2.10 (-3.02 to -1.17 ) | <.001 | 21 (16 to 27 ) | 15 (2 to 37 ) | -2.07 (-3.02 to -1.12 ) | <.001 |
| Jiaozuo | 23 (16 to 30 ) | 17 (3 to 40 ) | -1.60 (-2.89 to -0.29 ) | 0.017 | 22 (15 to 29 ) | 16 (3 to 38 ) | -1.65 (-2.98 to -0.30 ) | 0.017 | 22 (16 to 30 ) | 17 (3 to 39 ) | -1.62 (-2.93 to -0.30 ) | 0.017 |
| Jiyuan shi | 22 (15 to 31 ) | 16 (3 to 38 ) | -1.81 (-3.41 to -0.20 ) | 0.028 | 21 (14 to 30 ) | 15 (2 to 37 ) | -1.87 (-3.47 to -0.23 ) | 0.025 | 21 (15 to 30 ) | 15 (2 to 38 ) | -1.84 (-3.44 to -0.21 ) | 0.027 |
| Kaifeng | 22 (17 to 30 ) | 16 (3 to 39 ) | -1.81 (-2.46 to -1.15 ) | <.001 | 22 (16 to 29 ) | 15 (3 to 37 ) | -1.84 (-2.47 to -1.20 ) | <.001 | 22 (16 to 29 ) | 16 (3 to 38 ) | -1.82 (-2.47 to -1.17 ) | <.001 |
| Luohe | 26 (18 to 34 ) | 19 (4 to 43 ) | -1.89 (-3.28 to -0.47 ) | 0.009 | 25 (17 to 33 ) | 18 (3 to 41 ) | -1.95 (-3.39 to -0.49 ) | 0.009 | 25 (18 to 33 ) | 18 (4 to 42 ) | -1.91 (-3.33 to -0.48 ) | 0.009 |
| Luoyang | 21 (13 to 30 ) | 14 (2 to 37 ) | -1.99 (-3.24 to -0.73 ) | 0.002 | 20 (13 to 29 ) | 14 (2 to 35 ) | -1.89 (-3.64 to -0.11 ) | 0.037 | 20 (13 to 30 ) | 14 (2 to 36 ) | -2.01 (-3.27 to -0.75 ) | 0.002 |
| Nanyang | 27 (19 to 37 ) | 17 (3 to 41 ) | -2.40 (-3.15 to -1.64 ) | <.001 | 26 (18 to 35 ) | 16 (3 to 39 ) | -2.44 (-3.16 to -1.70 ) | <.001 | 27 (19 to 36 ) | 17 (3 to 40 ) | -2.42 (-3.16 to -1.67 ) | <.001 |
| Pingdingshan | 21 (14 to 30 ) | 16 (3 to 38 ) | -1.49 (-2.37 to -0.59 ) | 0.001 | 20 (14 to 29 ) | 15 (3 to 36 ) | -1.55 (-2.33 to -0.77 ) | <.001 | 21 (14 to 29 ) | 16 (3 to 37 ) | -1.52 (-2.35 to -0.68 ) | <.001 |
| Puyang | 23 (18 to 29 ) | 15 (2 to 38 ) | -2.49 (-4.09 to -0.87 ) | 0.003 | 22 (17 to 28 ) | 14 (2 to 36 ) | -2.54 (-4.13 to -0.92 ) | 0.002 | 23 (17 to 28 ) | 15 (2 to 37 ) | -2.51 (-4.11 to -0.89 ) | 0.002 |
| Sanmenxia | 23 (14 to 33 ) | 14 (3 to 37 ) | -2.55 (-4.38 to -0.69 ) | 0.007 | 22 (14 to 32 ) | 14 (2 to 35 ) | -2.60 (-4.44 to -0.72 ) | 0.007 | 22 (14 to 32 ) | 14 (2 to 36 ) | -2.57 (-4.41 to -0.71 ) | 0.007 |
| Shangqiu | 22 (16 to 28 ) | 15 (2 to 38 ) | -2.25 (-3.84 to -0.64 ) | 0.006 | 21 (16 to 27 ) | 14 (2 to 36 ) | -2.31 (-3.84 to -0.75 ) | 0.004 | 22 (16 to 27 ) | 15 (2 to 37 ) | -2.28 (-3.84 to -0.69 ) | 0.005 |
| Xinxiang | 22 (16 to 29 ) | 16 (3 to 39 ) | -1.79 (-2.44 to -1.14 ) | <.001 | 21 (15 to 28 ) | 15 (3 to 37 ) | -1.84 (-2.45 to -1.23 ) | <.001 | 22 (15 to 28 ) | 16 (3 to 38 ) | -1.82 (-2.45 to -1.18 ) | <.001 |
| Xinyang | 25 (18 to 34 ) | 15 (2 to 39 ) | -2.20 (-3.49 to -0.88 ) | 0.001 | 25 (17 to 33 ) | 14 (2 to 37 ) | -2.27 (-3.57 to -0.95 ) | 0.001 | 25 (18 to 33 ) | 15 (2 to 38 ) | -2.23 (-3.53 to -0.91 ) | 0.001 |
| Xuchang | 26 (19 to 36 ) | 20 (4 to 44 ) | -1.64 (-2.97 to -0.29 ) | 0.018 | 26 (18 to 34 ) | 19 (4 to 42 ) | -1.70 (-3.07 to -0.31 ) | 0.017 | 26 (18 to 35 ) | 20 (4 to 43 ) | -1.66 (-3.01 to -0.30 ) | 0.017 |
| Zhengzhou | 17 (12 to 24 ) | 13 (2 to 33 ) | -1.27 (-3.52 to 1.03 ) | 0.277 | 17 (11 to 23 ) | 13 (2 to 32 ) | -1.33 (-3.62 to 1.03 ) | 0.267 | 17 (12 to 24 ) | 13 (2 to 33 ) | -1.30 (-3.57 to 1.03 ) | 0.272 |
| Zhoukou | 25 (18 to 33 ) | 17 (3 to 41 ) | -2.06 (-4.25 to 0.17 ) | 0.070 | 24 (18 to 32 ) | 16 (3 to 39 ) | -2.14 (-4.32 to 0.08 ) | 0.059 | 25 (18 to 32 ) | 17 (3 to 40 ) | -2.10 (-4.28 to 0.13 ) | 0.064 |
| Zhumadian | 24 (17 to 32 ) | 16 (3 to 39 ) | -2.24 (-3.58 to -0.88 ) | 0.001 | 24 (17 to 31 ) | 15 (3 to 37 ) | -2.29 (-3.64 to -0.92 ) | 0.001 | 24 (17 to 32 ) | 16 (3 to 38 ) | -2.26 (-3.61 to -0.90 ) | 0.001 |
| Hubei |  |  |  |  |  |  |  |  |  |  |  |  |
| Enshi Tujia and Miao | 24 (17 to 31 ) | 15 (2 to 38 ) | -2.22 (-4.99 to 0.63 ) | 0.125 | 23 (17 to 30 ) | 14 (2 to 36 ) | -2.26 (-5.06 to 0.63 ) | 0.125 | 23 (17 to 30 ) | 15 (2 to 37 ) | -2.24 (-5.02 to 0.63 ) | 0.125 |
| Ezhou | 20 (14 to 28 ) | 15 (2 to 41 ) | -1.47 (-2.27 to -0.67 ) | <.001 | 20 (13 to 27 ) | 14 (2 to 39 ) | -1.54 (-2.34 to -0.73 ) | <.001 | 20 (14 to 28 ) | 14 (2 to 40 ) | -1.50 (-2.30 to -0.70 ) | <.001 |
| Huanggang | 23 (16 to 31 ) | 16 (2 to 43 ) | -1.79 (-2.40 to -1.17 ) | <.001 | 22 (15 to 30 ) | 15 (2 to 41 ) | -1.86 (-2.46 to -1.25 ) | <.001 | 23 (15 to 30 ) | 16 (2 to 42 ) | -1.82 (-2.43 to -1.21 ) | <.001 |
| Huangshi | 22 (16 to 30 ) | 16 (2 to 44 ) | -1.33 (-2.93 to 0.31 ) | 0.111 | 22 (15 to 29 ) | 16 (2 to 41 ) | -1.40 (-2.99 to 0.23 ) | 0.091 | 22 (15 to 29 ) | 16 (2 to 43 ) | -1.36 (-2.96 to 0.27 ) | 0.101 |
| Jingmen | 20 (14 to 28 ) | 14 (2 to 39 ) | -2.15 (-3.27 to -1.02 ) | <.001 | 20 (13 to 27 ) | 13 (2 to 37 ) | -2.18 (-3.24 to -1.11 ) | <.001 | 20 (14 to 27 ) | 14 (2 to 38 ) | -2.17 (-3.26 to -1.06 ) | <.001 |
| Jingzhou | 20 (14 to 26 ) | 14 (2 to 40 ) | -1.43 (-3.46 to 0.65 ) | 0.177 | 19 (14 to 25 ) | 14 (2 to 38 ) | -1.48 (-3.61 to 0.68 ) | 0.178 | 20 (14 to 25 ) | 14 (2 to 39 ) | -1.45 (-3.53 to 0.66 ) | 0.177 |
| Qianjiang | 20 (14 to 26 ) | 14 (2 to 40 ) | -1.64 (-2.99 to -0.26 ) | 0.020 | 19 (14 to 26 ) | 14 (2 to 38 ) | -1.69 (-3.13 to -0.23 ) | 0.023 | 20 (14 to 26 ) | 14 (2 to 39 ) | -1.66 (-3.05 to -0.25 ) | 0.021 |
| Shennongjia | 20 (13 to 29 ) | 14 (2 to 38 ) | -1.92 (-4.87 to 1.13 ) | 0.215 | 20 (12 to 28 ) | 13 (2 to 36 ) | -1.99 (-5.00 to 1.11 ) | 0.206 | 20 (13 to 28 ) | 14 (2 to 37 ) | -1.95 (-4.93 to 1.12 ) | 0.210 |
| Shiyan | 25 (17 to 35 ) | 17 (3 to 43 ) | -1.80 (-5.90 to 2.47 ) | 0.403 | 25 (16 to 34 ) | 16 (3 to 41 ) | -1.88 (-6.10 to 2.54 ) | 0.399 | 25 (17 to 35 ) | 17 (3 to 42 ) | -1.84 (-5.99 to 2.50 ) | 0.401 |
| Suizhou Shi | 20 (13 to 28 ) | 14 (2 to 38 ) | -1.98 (-3.37 to -0.57 ) | 0.006 | 19 (13 to 27 ) | 14 (2 to 37 ) | -2.03 (-3.39 to -0.66 ) | 0.004 | 20 (13 to 27 ) | 14 (2 to 38 ) | -2.01 (-3.38 to -0.61 ) | 0.005 |
| Tianmen | 20 (14 to 27 ) | 14 (2 to 40 ) | -1.66 (-3.56 to 0.28 ) | 0.093 | 19 (13 to 26 ) | 14 (2 to 38 ) | -1.71 (-3.67 to 0.28 ) | 0.092 | 20 (14 to 27 ) | 14 (2 to 39 ) | -1.68 (-3.61 to 0.28 ) | 0.093 |
| Wuhan | 14 (9 to 20 ) | 11 (1 to 34 ) | -1.15 (-2.49 to 0.21 ) | 0.096 | 14 (9 to 20 ) | 10 (1 to 32 ) | -1.22 (-2.56 to 0.14 ) | 0.079 | 14 (9 to 20 ) | 11 (1 to 33 ) | -1.18 (-2.52 to 0.18 ) | 0.088 |
| Xiangfan | 22 (15 to 31 ) | 16 (3 to 41 ) | -2.24 (-3.04 to -1.43 ) | <.001 | 22 (14 to 30 ) | 15 (3 to 39 ) | -2.31 (-3.11 to -1.51 ) | <.001 | 22 (14 to 30 ) | 16 (3 to 40 ) | -2.27 (-3.07 to -1.47 ) | <.001 |
| Xianning | 22 (16 to 29 ) | 17 (2 to 44 ) | -1.05 (-3.23 to 1.19 ) | 0.356 | 21 (15 to 28 ) | 16 (2 to 42 ) | -1.09 (-3.29 to 1.16 ) | 0.341 | 21 (15 to 28 ) | 17 (2 to 43 ) | -1.07 (-3.26 to 1.17 ) | 0.348 |
| Xiantao | 19 (13 to 26 ) | 14 (2 to 39 ) | -1.38 (-3.10 to 0.37 ) | 0.122 | 18 (13 to 25 ) | 13 (2 to 37 ) | -1.43 (-3.23 to 0.40 ) | 0.125 | 19 (13 to 25 ) | 14 (2 to 38 ) | -1.40 (-3.16 to 0.38 ) | 0.123 |
| Xiaogan | 19 (13 to 27 ) | 14 (2 to 38 ) | -1.76 (-2.86 to -0.66 ) | 0.002 | 19 (12 to 26 ) | 13 (2 to 36 ) | -1.85 (-2.96 to -0.72 ) | 0.001 | 19 (13 to 26 ) | 14 (2 to 37 ) | -1.80 (-2.90 to -0.68 ) | 0.002 |
| Yichang | 22 (15 to 30 ) | 15 (3 to 40 ) | -2.22 (-3.27 to -1.16 ) | <.001 | 22 (15 to 29 ) | 14 (2 to 38 ) | -2.25 (-3.31 to -1.18 ) | <.001 | 22 (15 to 30 ) | 14 (2 to 39 ) | -2.24 (-3.29 to -1.17 ) | <.001 |
| Hunan |  |  |  |  |  |  |  |  |  |  |  |  |
| Changde | 28 (21 to 34 ) | 20 (3 to 51 ) | -1.91 (-4.43 to 0.67 ) | 0.146 | 27 (21 to 33 ) | 19 (3 to 48 ) | -1.99 (-4.55 to 0.64 ) | 0.137 | 28 (21 to 34 ) | 19 (3 to 49 ) | -1.95 (-4.49 to 0.66 ) | 0.142 |
| Changsha | 23 (18 to 29 ) | 18 (3 to 50 ) | -1.26 (-2.24 to -0.27 ) | 0.013 | 22 (17 to 28 ) | 17 (2 to 47 ) | -1.46 (-2.73 to -0.17 ) | 0.027 | 23 (18 to 28 ) | 17 (2 to 48 ) | -1.43 (-2.71 to -0.13 ) | 0.031 |
| Chenzhou | 29 (23 to 35 ) | 24 (4 to 58 ) | -1.05 (-3.45 to 1.42 ) | 0.402 | 28 (22 to 34 ) | 22 (4 to 55 ) | -1.12 (-3.47 to 1.28 ) | 0.358 | 28 (22 to 35 ) | 23 (4 to 56 ) | -1.08 (-3.46 to 1.36 ) | 0.382 |
| Hengyang | 27 (21 to 33 ) | 21 (3 to 54 ) | -1.09 (-3.26 to 1.12 ) | 0.331 | 26 (20 to 31 ) | 20 (3 to 52 ) | -1.14 (-3.30 to 1.06 ) | 0.307 | 26 (21 to 32 ) | 20 (3 to 53 ) | -1.12 (-3.28 to 1.09 ) | 0.319 |
| Huaihua | 33 (27 to 38 ) | 22 (4 to 55 ) | -1.92 (-4.02 to 0.23 ) | 0.079 | 32 (26 to 36 ) | 21 (4 to 52 ) | -1.95 (-4.07 to 0.21 ) | 0.076 | 32 (27 to 37 ) | 22 (4 to 53 ) | -1.94 (-4.04 to 0.22 ) | 0.078 |
| Loudi | 27 (21 to 32 ) | 20 (3 to 53 ) | -1.76 (-2.51 to -1.01 ) | <.001 | 26 (20 to 31 ) | 19 (3 to 51 ) | -1.80 (-2.53 to -1.07 ) | <.001 | 26 (21 to 31 ) | 19 (3 to 52 ) | -1.78 (-2.52 to -1.04 ) | <.001 |
| Shaoyang | 29 (23 to 33 ) | 20 (3 to 52 ) | -2.00 (-2.65 to -1.34 ) | <.001 | 28 (23 to 32 ) | 19 (3 to 50 ) | -2.03 (-2.67 to -1.40 ) | <.001 | 28 (23 to 33 ) | 19 (3 to 51 ) | -2.01 (-2.66 to -1.37 ) | <.001 |
| Xiangtan | 26 (20 to 31 ) | 20 (3 to 54 ) | -1.25 (-2.62 to 0.15 ) | 0.079 | 25 (20 to 30 ) | 19 (3 to 51 ) | -1.31 (-2.66 to 0.07 ) | 0.063 | 25 (20 to 31 ) | 20 (3 to 53 ) | -1.28 (-2.64 to 0.11 ) | 0.071 |
| Xiangxi Tujia and Miao | 32 (25 to 38 ) | 20 (3 to 51 ) | -2.42 (-5.03 to 0.26 ) | 0.077 | 31 (24 to 37 ) | 19 (3 to 48 ) | -2.46 (-5.06 to 0.22 ) | 0.071 | 31 (25 to 37 ) | 20 (3 to 49 ) | -2.44 (-5.04 to 0.24 ) | 0.074 |
| Yiyang | 25 (20 to 31 ) | 18 (3 to 51 ) | -1.65 (-3.16 to -0.11 ) | 0.036 | 24 (19 to 30 ) | 18 (3 to 48 ) | -1.72 (-3.25 to -0.17 ) | 0.030 | 25 (19 to 30 ) | 18 (3 to 50 ) | -1.68 (-3.20 to -0.14 ) | 0.033 |
| Yongzhou | 28 (23 to 34 ) | 22 (4 to 56 ) | -1.40 (-2.82 to 0.04 ) | 0.057 | 27 (22 to 33 ) | 21 (3 to 53 ) | -1.47 (-2.94 to 0.02 ) | 0.052 | 28 (22 to 33 ) | 21 (4 to 55 ) | -1.43 (-2.87 to 0.03 ) | 0.054 |
| Yueyang | 24 (18 to 31 ) | 18 (3 to 50 ) | -1.55 (-2.55 to -0.54 ) | 0.003 | 23 (18 to 30 ) | 17 (3 to 47 ) | -1.63 (-2.61 to -0.63 ) | 0.001 | 24 (18 to 30 ) | 18 (3 to 48 ) | -1.58 (-2.58 to -0.58 ) | 0.002 |
| Zhangjiajie | 30 (23 to 37 ) | 20 (3 to 51 ) | -2.10 (-6.69 to 2.72 ) | 0.387 | 29 (22 to 35 ) | 19 (3 to 48 ) | -2.17 (-6.79 to 2.68 ) | 0.375 | 29 (22 to 36 ) | 20 (3 to 50 ) | -2.13 (-6.73 to 2.70 ) | 0.381 |
| Zhuzhou | 27 (20 to 33 ) | 22 (3 to 56 ) | -0.83 (-1.88 to 0.23 ) | 0.125 | 26 (19 to 32 ) | 21 (3 to 53 ) | -0.89 (-1.93 to 0.16 ) | 0.095 | 26 (20 to 33 ) | 21 (3 to 55 ) | -0.86 (-1.90 to 0.20 ) | 0.110 |
| Jiangsu |  |  |  |  |  |  |  |  |  |  |  |  |
| Changzhou | 12 (9 to 17 ) | 8 (1 to 25 ) | -2.26 (-5.00 to 0.57 ) | 0.117 | 12 (8 to 16 ) | 8 (1 to 24 ) | -2.34 (-5.02 to 0.42 ) | 0.096 | 12 (9 to 16 ) | 8 (1 to 25 ) | -2.29 (-5.01 to 0.50 ) | 0.107 |
| Huai'an | 14 (11 to 18 ) | 10 (1 to 29 ) | -1.81 (-3.34 to -0.26 ) | 0.022 | 14 (10 to 17 ) | 10 (1 to 27 ) | -1.89 (-3.60 to -0.15 ) | 0.033 | 14 (10 to 18 ) | 10 (1 to 28 ) | -1.85 (-3.46 to -0.22 ) | 0.027 |
| Lianyungang | 13 (10 to 17 ) | 9 (1 to 27 ) | -1.78 (-4.12 to 0.63 ) | 0.147 | 13 (10 to 16 ) | 9 (1 to 26 ) | -1.87 (-4.20 to 0.53 ) | 0.126 | 13 (10 to 17 ) | 9 (1 to 27 ) | -1.82 (-4.16 to 0.58 ) | 0.137 |
| Nanjing | 11 (8 to 15 ) | 8 (1 to 25 ) | -1.51 (-3.75 to 0.77 ) | 0.193 | 11 (8 to 14 ) | 8 (1 to 24 ) | -1.57 (-3.84 to 0.76 ) | 0.186 | 11 (8 to 15 ) | 8 (1 to 24 ) | -1.54 (-3.79 to 0.77 ) | 0.190 |
| Nantong | 14 (9 to 18 ) | 9 (1 to 27 ) | -1.99 (-3.75 to -0.21 ) | 0.028 | 13 (9 to 18 ) | 8 (1 to 25 ) | -2.06 (-3.83 to -0.25 ) | 0.026 | 14 (9 to 18 ) | 9 (1 to 26 ) | -2.02 (-3.78 to -0.23 ) | 0.027 |
| Suqian | 14 (11 to 18 ) | 10 (1 to 29 ) | -1.81 (-3.73 to 0.14 ) | 0.068 | 14 (10 to 18 ) | 10 (1 to 28 ) | -2.17 (-3.75 to -0.56 ) | 0.008 | 14 (11 to 18 ) | 10 (1 to 28 ) | -2.14 (-3.73 to -0.51 ) | 0.010 |
| Suzhou | 11 (7 to 15 ) | 7 (1 to 22 ) | -2.53 (-5.76 to 0.80 ) | 0.135 | 11 (7 to 15 ) | 6 (1 to 21 ) | -2.63 (-7.25 to 2.22 ) | 0.283 | 11 (7 to 15 ) | 7 (1 to 22 ) | -2.57 (-5.79 to 0.76 ) | 0.129 |
| Taizhou | 15 (11 to 19 ) | 10 (1 to 29 ) | -1.83 (-3.83 to 0.22 ) | 0.080 | 14 (11 to 19 ) | 9 (1 to 27 ) | -1.88 (-3.93 to 0.21 ) | 0.078 | 15 (11 to 19 ) | 10 (1 to 28 ) | -1.85 (-3.88 to 0.22 ) | 0.079 |
| Wuxi | 11 (8 to 16 ) | 7 (1 to 23 ) | -2.41 (-5.69 to 0.99 ) | 0.163 | 11 (8 to 15 ) | 7 (1 to 22 ) | -2.49 (-5.69 to 0.83 ) | 0.140 | 11 (8 to 15 ) | 7 (1 to 23 ) | -2.44 (-5.69 to 0.91 ) | 0.152 |
| Xuzhou | 15 (11 to 18 ) | 10 (1 to 29 ) | -2.21 (-4.06 to -0.33 ) | 0.021 | 14 (10 to 18 ) | 10 (1 to 27 ) | -2.28 (-3.87 to -0.66 ) | 0.006 | 14 (11 to 18 ) | 10 (1 to 28 ) | -2.24 (-4.07 to -0.37 ) | 0.019 |
| Yancheng | 14 (10 to 18 ) | 10 (1 to 29 ) | -2.14 (-3.40 to -0.87 ) | 0.001 | 13 (10 to 17 ) | 9 (1 to 27 ) | -2.20 (-3.42 to -0.96 ) | 0.001 | 13 (10 to 17 ) | 9 (1 to 28 ) | -2.17 (-3.41 to -0.91 ) | 0.001 |
| Yangzhou | 14 (10 to 18 ) | 10 (1 to 29 ) | -1.82 (-3.10 to -0.52 ) | 0.006 | 13 (10 to 17 ) | 9 (1 to 27 ) | -1.91 (-3.21 to -0.60 ) | 0.004 | 13 (10 to 17 ) | 9 (1 to 28 ) | -1.86 (-3.15 to -0.56 ) | 0.005 |
| Zhenjiang | 13 (10 to 18 ) | 9 (1 to 27 ) | -2.04 (-4.10 to 0.06 ) | 0.057 | 13 (9 to 17 ) | 9 (1 to 26 ) | -2.12 (-4.17 to -0.03 ) | 0.046 | 13 (9 to 17 ) | 9 (1 to 26 ) | -2.08 (-4.13 to 0.01 ) | 0.051 |
| Jiangxi |  |  |  |  |  |  |  |  |  |  |  |  |
| Fuzhou | 21 (14 to 29 ) | 13 (2 to 32 ) | -2.47 (-3.83 to -1.08 ) | 0.001 | 20 (14 to 28 ) | 12 (2 to 31 ) | -2.53 (-3.90 to -1.14 ) | <.001 | 21 (14 to 29 ) | 12 (2 to 31 ) | -2.50 (-3.86 to -1.11 ) | <.001 |
| Ganzhou | 23 (16 to 30 ) | 14 (2 to 34 ) | -2.24 (-3.26 to -1.21 ) | <.001 | 22 (16 to 29 ) | 13 (2 to 32 ) | -2.27 (-3.37 to -1.16 ) | <.001 | 22 (16 to 30 ) | 14 (2 to 33 ) | -2.25 (-3.31 to -1.19 ) | <.001 |
| Ji'an | 20 (14 to 27 ) | 13 (2 to 33 ) | -1.92 (-2.98 to -0.86 ) | <.001 | 19 (13 to 26 ) | 13 (2 to 31 ) | -1.96 (-3.04 to -0.87 ) | <.001 | 19 (14 to 26 ) | 13 (2 to 32 ) | -1.94 (-3.01 to -0.86 ) | <.001 |
| Jingdezhen | 23 (17 to 31 ) | 13 (2 to 33 ) | -2.89 (-6.17 to 0.51 ) | 0.095 | 23 (16 to 30 ) | 13 (2 to 31 ) | -2.97 (-6.29 to 0.48 ) | 0.090 | 23 (16 to 31 ) | 13 (2 to 32 ) | -2.92 (-6.22 to 0.50 ) | 0.093 |
| Jiujiang | 19 (13 to 25 ) | 12 (2 to 31 ) | -2.46 (-4.76 to -0.11 ) | 0.040 | 18 (13 to 24 ) | 11 (2 to 29 ) | -3.25 (-5.63 to -0.82 ) | 0.009 | 19 (13 to 25 ) | 12 (2 to 30 ) | -2.50 (-4.81 to -0.13 ) | 0.039 |
| Nanchang | 16 (10 to 23 ) | 9 (1 to 28 ) | -2.76 (-4.26 to -1.23 ) | <.001 | 15 (10 to 22 ) | 9 (1 to 26 ) | -2.81 (-4.32 to -1.27 ) | <.001 | 16 (10 to 23 ) | 9 (1 to 27 ) | -2.78 (-4.29 to -1.25 ) | <.001 |
| Pingxiang | 18 (13 to 24 ) | 13 (2 to 33 ) | -1.80 (-2.84 to -0.74 ) | 0.001 | 18 (13 to 23 ) | 12 (2 to 31 ) | -1.84 (-2.90 to -0.76 ) | 0.001 | 18 (13 to 23 ) | 13 (2 to 32 ) | -1.82 (-2.87 to -0.75 ) | 0.001 |
| Shangrao | 22 (15 to 30 ) | 13 (2 to 32 ) | -2.90 (-3.78 to -2.01 ) | <.001 | 21 (15 to 29 ) | 12 (2 to 31 ) | -2.97 (-3.84 to -2.10 ) | <.001 | 22 (15 to 30 ) | 13 (2 to 31 ) | -2.93 (-3.80 to -2.05 ) | <.001 |
| Xinyu | 19 (13 to 26 ) | 13 (2 to 32 ) | -2.07 (-3.07 to -1.07 ) | <.001 | 19 (13 to 25 ) | 12 (2 to 30 ) | -2.12 (-3.20 to -1.03 ) | <.001 | 19 (13 to 25 ) | 12 (2 to 31 ) | -2.09 (-3.13 to -1.05 ) | <.001 |
| Yichun | 20 (14 to 26 ) | 13 (2 to 33 ) | -2.39 (-3.12 to -1.66 ) | <.001 | 19 (14 to 26 ) | 12 (2 to 31 ) | -2.46 (-2.91 to -2.00 ) | <.001 | 20 (14 to 26 ) | 13 (2 to 32 ) | -2.40 (-3.11 to -1.69 ) | <.001 |
| Yingtan | 23 (15 to 32 ) | 14 (2 to 33 ) | -2.86 (-3.72 to -2.00 ) | <.001 | 22 (15 to 31 ) | 13 (2 to 31 ) | -2.90 (-3.70 to -2.09 ) | <.001 | 22 (15 to 31 ) | 13 (2 to 32 ) | -2.88 (-3.71 to -2.05 ) | <.001 |
| Jilin |  |  |  |  |  |  |  |  |  |  |  |  |
| Baicheng | 19 (11 to 29 ) | 9 (1 to 28 ) | -3.71 (-4.50 to -2.91 ) | <.001 | 19 (11 to 28 ) | 9 (1 to 27 ) | -3.80 (-4.60 to -3.00 ) | <.001 | 19 (11 to 28 ) | 9 (1 to 28 ) | -3.75 (-4.54 to -2.95 ) | <.001 |
| Baishan | 14 (7 to 22 ) | 11 (1 to 30 ) | -1.08 (-1.78 to -0.38 ) | 0.003 | 13 (7 to 22 ) | 10 (1 to 28 ) | -1.24 (-2.54 to 0.09 ) | 0.067 | 13 (7 to 22 ) | 11 (1 to 29 ) | -1.11 (-1.82 to -0.40 ) | 0.002 |
| Changchun | 13 (8 to 20 ) | 10 (1 to 27 ) | -1.52 (-1.79 to -1.25 ) | <.001 | 13 (7 to 19 ) | 9 (1 to 25 ) | -1.60 (-1.88 to -1.33 ) | <.001 | 13 (8 to 19 ) | 9 (1 to 26 ) | -1.56 (-1.83 to -1.28 ) | <.001 |
| Jilin | 14 (7 to 22 ) | 11 (1 to 30 ) | -1.10 (-1.65 to -0.54 ) | <.001 | 14 (7 to 22 ) | 11 (1 to 29 ) | -1.19 (-1.76 to -0.61 ) | <.001 | 14 (7 to 22 ) | 11 (1 to 29 ) | -1.14 (-1.70 to -0.57 ) | <.001 |
| Liaoyuan | 17 (11 to 26 ) | 11 (1 to 30 ) | -2.11 (-2.49 to -1.72 ) | <.001 | 17 (10 to 25 ) | 11 (1 to 29 ) | -2.19 (-2.57 to -1.80 ) | <.001 | 17 (10 to 25 ) | 11 (1 to 30 ) | -2.14 (-2.53 to -1.76 ) | <.001 |
| Siping | 17 (11 to 25 ) | 10 (1 to 29 ) | -2.51 (-3.18 to -1.84 ) | <.001 | 16 (10 to 24 ) | 10 (1 to 28 ) | -2.61 (-3.27 to -1.94 ) | <.001 | 16 (11 to 24 ) | 10 (1 to 28 ) | -2.55 (-3.22 to -1.88 ) | <.001 |
| Songyuan | 15 (9 to 24 ) | 10 (1 to 29 ) | -2.33 (-2.92 to -1.74 ) | <.001 | 15 (9 to 23 ) | 9 (1 to 27 ) | -2.41 (-2.99 to -1.83 ) | <.001 | 15 (9 to 24 ) | 10 (1 to 28 ) | -2.36 (-2.95 to -1.77 ) | <.001 |
| Tonghua | 19 (11 to 28 ) | 13 (2 to 32 ) | -2.00 (-2.55 to -1.44 ) | <.001 | 18 (10 to 27 ) | 12 (1 to 30 ) | -2.08 (-2.63 to -1.53 ) | <.001 | 19 (11 to 28 ) | 13 (2 to 31 ) | -2.04 (-2.59 to -1.48 ) | <.001 |
| Yanbian Korean | 11 (4 to 21 ) | 11 (1 to 32 ) | -0.05 (-0.73 to 0.64 ) | 0.890 | 11 (4 to 20 ) | 10 (1 to 30 ) | -0.13 (-0.80 to 0.55 ) | 0.711 | 11 (4 to 21 ) | 11 (1 to 31 ) | -0.08 (-0.76 to 0.60 ) | 0.808 |
| Liaoning |  |  |  |  |  |  |  |  |  |  |  |  |
| Anshan | 10 (7 to 14 ) | 8 (1 to 22 ) | -1.42 (-1.57 to -1.28 ) | <.001 | 10 (7 to 13 ) | 7 (1 to 21 ) | -1.49 (-1.66 to -1.31 ) | <.001 | 10 (7 to 13 ) | 7 (1 to 21 ) | -1.45 (-1.61 to -1.29 ) | <.001 |
| Benxi | 8 (5 to 11 ) | 7 (1 to 21 ) | -0.67 (-1.90 to 0.58 ) | 0.291 | 8 (5 to 11 ) | 7 (1 to 20 ) | -0.87 (-1.77 to 0.04 ) | 0.062 | 8 (5 to 11 ) | 7 (1 to 20 ) | -0.70 (-1.97 to 0.58 ) | 0.283 |
| Chaoyang | 15 (11 to 20 ) | 7 (1 to 22 ) | -3.74 (-5.15 to -2.31 ) | <.001 | 15 (11 to 19 ) | 7 (1 to 21 ) | -3.83 (-5.22 to -2.42 ) | <.001 | 15 (11 to 20 ) | 7 (1 to 22 ) | -3.78 (-5.18 to -2.36 ) | <.001 |
| Dalian | 6 (4 to 9 ) | 5 (1 to 17 ) | -0.71 (-4.00 to 2.68 ) | 0.676 | 6 (4 to 9 ) | 5 (1 to 16 ) | -0.77 (-4.10 to 2.67 ) | 0.656 | 6 (4 to 9 ) | 5 (1 to 17 ) | -0.74 (-4.05 to 2.68 ) | 0.667 |
| Dandong | 10 (7 to 14 ) | 9 (1 to 23 ) | -0.89 (-1.43 to -0.35 ) | 0.001 | 10 (6 to 14 ) | 8 (1 to 22 ) | -1.10 (-1.81 to -0.39 ) | 0.003 | 10 (6 to 14 ) | 8 (1 to 22 ) | -1.05 (-1.76 to -0.33 ) | 0.004 |
| Fushun | 9 (6 to 13 ) | 8 (1 to 22 ) | -0.91 (-2.63 to 0.85 ) | 0.309 | 9 (6 to 12 ) | 7 (1 to 21 ) | -0.96 (-2.61 to 0.72 ) | 0.262 | 9 (6 to 12 ) | 8 (1 to 21 ) | -0.93 (-2.62 to 0.79 ) | 0.287 |
| Fuxin | 11 (8 to 15 ) | 7 (1 to 21 ) | -2.63 (-3.48 to -1.76 ) | <.001 | 10 (7 to 14 ) | 6 (1 to 20 ) | -2.68 (-3.48 to -1.86 ) | <.001 | 10 (7 to 15 ) | 7 (1 to 20 ) | -2.65 (-3.48 to -1.81 ) | <.001 |
| Huludao | 13 (10 to 17 ) | 7 (1 to 22 ) | -2.85 (-4.11 to -1.57 ) | <.001 | 13 (9 to 17 ) | 7 (1 to 21 ) | -3.03 (-4.63 to -1.40 ) | <.001 | 13 (9 to 17 ) | 7 (1 to 21 ) | -3.01 (-4.56 to -1.43 ) | <.001 |
| Jinzhou | 10 (8 to 14 ) | 6 (1 to 20 ) | -2.54 (-2.96 to -2.11 ) | <.001 | 10 (7 to 13 ) | 6 (1 to 19 ) | -2.57 (-2.96 to -2.18 ) | <.001 | 10 (7 to 14 ) | 6 (1 to 20 ) | -2.55 (-2.96 to -2.15 ) | <.001 |
| Liaoyang | 11 (8 to 15 ) | 9 (1 to 23 ) | -1.44 (-2.00 to -0.88 ) | <.001 | 11 (8 to 15 ) | 8 (1 to 22 ) | -1.50 (-2.03 to -0.98 ) | <.001 | 11 (8 to 15 ) | 9 (1 to 23 ) | -1.47 (-2.01 to -0.92 ) | <.001 |
| Panjin | 13 (9 to 17 ) | 8 (1 to 23 ) | -2.38 (-2.68 to -2.07 ) | <.001 | 12 (9 to 16 ) | 8 (1 to 22 ) | -2.44 (-2.74 to -2.14 ) | <.001 | 12 (9 to 17 ) | 8 (1 to 22 ) | -2.40 (-2.70 to -2.10 ) | <.001 |
| Shenyang | 9 (6 to 13 ) | 7 (1 to 21 ) | -1.08 (-2.68 to 0.56 ) | 0.196 | 9 (6 to 12 ) | 7 (1 to 20 ) | -1.32 (-2.37 to -0.26 ) | 0.015 | 9 (6 to 12 ) | 7 (1 to 20 ) | -1.29 (-2.37 to -0.20 ) | 0.021 |
| Tieling | 10 (7 to 14 ) | 8 (1 to 22 ) | -1.62 (-2.43 to -0.81 ) | <.001 | 10 (7 to 13 ) | 7 (1 to 21 ) | -1.68 (-2.42 to -0.93 ) | <.001 | 10 (7 to 14 ) | 7 (1 to 21 ) | -1.65 (-2.43 to -0.86 ) | <.001 |
| Inner Mongolia |  |  |  |  |  |  |  |  |  |  |  |  |
| Alxa | 18 (12 to 25 ) | 5 (1 to 14 ) | -7.21 (-9.32 to -5.04 ) | <.001 | 18 (12 to 24 ) | 5 (1 to 13 ) | -7.25 (-9.32 to -5.12 ) | <.001 | 18 (12 to 24 ) | 5 (1 to 13 ) | -7.23 (-9.32 to -5.08 ) | <.001 |
| Baotou | 15 (10 to 21 ) | 4 (1 to 13 ) | -6.64 (-7.52 to -5.76 ) | <.001 | 14 (10 to 20 ) | 4 (1 to 13 ) | -6.69 (-7.59 to -5.78 ) | <.001 | 15 (10 to 20 ) | 4 (1 to 13 ) | -6.66 (-7.55 to -5.77 ) | <.001 |
| Baynnur | 20 (15 to 26 ) | 6 (1 to 16 ) | -6.78 (-8.60 to -4.91 ) | <.001 | 20 (14 to 25 ) | 5 (1 to 15 ) | -7.13 (-8.72 to -5.51 ) | <.001 | 20 (14 to 26 ) | 5 (1 to 15 ) | -7.11 (-8.74 to -5.45 ) | <.001 |
| Chifeng | 16 (12 to 20 ) | 7 (1 to 19 ) | -4.14 (-4.99 to -3.27 ) | <.001 | 15 (11 to 19 ) | 7 (1 to 18 ) | -4.24 (-5.07 to -3.41 ) | <.001 | 15 (11 to 20 ) | 7 (1 to 19 ) | -4.16 (-5.02 to -3.30 ) | <.001 |
| Hohhot | 17 (12 to 22 ) | 5 (1 to 15 ) | -6.03 (-6.46 to -5.58 ) | <.001 | 16 (12 to 21 ) | 5 (1 to 14 ) | -6.07 (-6.54 to -5.60 ) | <.001 | 17 (12 to 22 ) | 5 (1 to 15 ) | -6.05 (-6.50 to -5.59 ) | <.001 |
| Hulunbuir | 8 (4 to 13 ) | 5 (1 to 16 ) | -3.22 (-6.29 to -0.05 ) | 0.047 | 8 (4 to 12 ) | 5 (1 to 15 ) | -3.27 (-6.27 to -0.18 ) | 0.038 | 8 (4 to 12 ) | 5 (1 to 16 ) | -3.24 (-6.28 to -0.11 ) | 0.043 |
| Ordos | 16 (11 to 21 ) | 4 (1 to 14 ) | -6.14 (-7.10 to -5.17 ) | <.001 | 15 (10 to 20 ) | 4 (1 to 13 ) | -6.49 (-7.34 to -5.63 ) | <.001 | 15 (10 to 21 ) | 4 (1 to 13 ) | -6.17 (-7.14 to -5.19 ) | <.001 |
| Tongliao | 10 (7 to 14 ) | 6 (1 to 18 ) | -2.56 (-3.28 to -1.83 ) | <.001 | 10 (7 to 14 ) | 6 (1 to 17 ) | -2.62 (-3.35 to -1.89 ) | <.001 | 10 (7 to 14 ) | 6 (1 to 18 ) | -2.59 (-3.31 to -1.86 ) | <.001 |
| Ulaan Chab | 16 (12 to 21 ) | 5 (1 to 15 ) | -5.62 (-7.21 to -4.01 ) | <.001 | 16 (12 to 20 ) | 5 (1 to 15 ) | -5.69 (-7.01 to -4.34 ) | <.001 | 16 (12 to 21 ) | 5 (1 to 15 ) | -5.66 (-6.96 to -4.34 ) | <.001 |
| Wuhai | 18 (11 to 25 ) | 5 (1 to 14 ) | -6.96 (-8.20 to -5.70 ) | <.001 | 17 (11 to 24 ) | 4 (1 to 13 ) | -7.01 (-8.25 to -5.75 ) | <.001 | 17 (11 to 24 ) | 5 (1 to 14 ) | -6.98 (-8.22 to -5.72 ) | <.001 |
| Xilin Gol | 17 (14 to 21 ) | 5 (1 to 15 ) | -5.88 (-6.70 to -5.05 ) | <.001 | 16 (13 to 20 ) | 5 (1 to 14 ) | -5.97 (-6.79 to -5.14 ) | <.001 | 17 (13 to 20 ) | 5 (1 to 15 ) | -5.92 (-6.74 to -5.09 ) | <.001 |
| Xing'an | 9 (5 to 14 ) | 6 (1 to 18 ) | -2.93 (-4.63 to -1.19 ) | 0.001 | 9 (5 to 13 ) | 6 (1 to 17 ) | -3.06 (-4.28 to -1.82 ) | <.001 | 9 (5 to 14 ) | 6 (1 to 17 ) | -2.95 (-4.62 to -1.25 ) | 0.001 |
| Ningxia |  |  |  |  |  |  |  |  |  |  |  |  |
| Guyuan | 13 (8 to 18 ) | 5 (1 to 13 ) | -4.93 (-6.08 to -3.76 ) | <.001 | 13 (8 to 17 ) | 5 (1 to 12 ) | -4.97 (-6.12 to -3.80 ) | <.001 | 13 (8 to 18 ) | 5 (1 to 12 ) | -4.95 (-6.10 to -3.78 ) | <.001 |
| Shizuishan | 13 (8 to 17 ) | 5 (1 to 13 ) | -5.46 (-6.57 to -4.34 ) | <.001 | 12 (8 to 17 ) | 4 (1 to 12 ) | -5.52 (-6.58 to -4.44 ) | <.001 | 12 (8 to 17 ) | 5 (1 to 12 ) | -5.49 (-6.58 to -4.39 ) | <.001 |
| Wuzhong | 13 (9 to 18 ) | 5 (1 to 13 ) | -5.06 (-5.38 to -4.72 ) | <.001 | 13 (8 to 17 ) | 5 (1 to 12 ) | -5.14 (-5.51 to -4.77 ) | <.001 | 13 (9 to 18 ) | 5 (1 to 12 ) | -5.09 (-5.44 to -4.75 ) | <.001 |
| Yinchuan | 13 (9 to 18 ) | 5 (1 to 13 ) | -5.16 (-6.37 to -3.94 ) | <.001 | 13 (9 to 17 ) | 5 (1 to 12 ) | -5.50 (-6.08 to -4.93 ) | <.001 | 13 (9 to 18 ) | 5 (1 to 12 ) | -5.19 (-6.43 to -3.93 ) | <.001 |
| Zhongwei | 13 (8 to 18 ) | 5 (1 to 13 ) | -5.06 (-5.34 to -4.78 ) | <.001 | 13 (8 to 17 ) | 5 (1 to 12 ) | -5.15 (-5.44 to -4.87 ) | <.001 | 13 (8 to 18 ) | 5 (1 to 12 ) | -5.10 (-5.38 to -4.82 ) | <.001 |
| Qinghai |  |  |  |  |  |  |  |  |  |  |  |  |
| Golog Tibetan | 25 (15 to 33 ) | 19 (4 to 42 ) | -1.63 (-2.69 to -0.56 ) | 0.003 | 24 (15 to 32 ) | 18 (3 to 40 ) | -1.68 (-2.70 to -0.66 ) | 0.001 | 25 (15 to 33 ) | 18 (3 to 41 ) | -1.65 (-2.69 to -0.60 ) | 0.002 |
| Gyêgu Tibetan | 24 (12 to 34 ) | 15 (3 to 38 ) | -2.49 (-2.97 to -2.00 ) | <.001 | 23 (12 to 33 ) | 14 (2 to 36 ) | -2.57 (-3.06 to -2.07 ) | <.001 | 24 (12 to 34 ) | 15 (3 to 37 ) | -2.52 (-3.01 to -2.03 ) | <.001 |
| Haibei Tibetan | 22 (14 to 30 ) | 16 (3 to 39 ) | -1.75 (-2.33 to -1.16 ) | <.001 | 22 (14 to 29 ) | 16 (3 to 37 ) | -1.81 (-2.34 to -1.27 ) | <.001 | 22 (14 to 30 ) | 16 (3 to 38 ) | -1.77 (-2.33 to -1.21 ) | <.001 |
| Haidong | 23 (15 to 31 ) | 16 (3 to 38 ) | -1.98 (-2.85 to -1.10 ) | <.001 | 22 (14 to 30 ) | 15 (3 to 36 ) | -2.05 (-2.92 to -1.18 ) | <.001 | 22 (14 to 30 ) | 15 (3 to 37 ) | -2.01 (-2.88 to -1.14 ) | <.001 |
| Hainan Tibetan | 24 (15 to 32 ) | 17 (3 to 39 ) | -1.92 (-2.28 to -1.56 ) | <.001 | 23 (15 to 31 ) | 16 (3 to 37 ) | -2.01 (-2.38 to -1.64 ) | <.001 | 24 (15 to 31 ) | 16 (3 to 38 ) | -1.96 (-2.33 to -1.59 ) | <.001 |
| Haixi Mongol and Tibetan | 21 (11 to 32 ) | 13 (2 to 33 ) | -2.77 (-4.42 to -1.09 ) | 0.001 | 20 (11 to 30 ) | 12 (2 to 31 ) | -2.86 (-4.47 to -1.21 ) | 0.001 | 21 (11 to 31 ) | 12 (2 to 32 ) | -2.81 (-4.45 to -1.15 ) | 0.001 |
| Huangnan Tibetan | 24 (16 to 32 ) | 18 (3 to 41 ) | -1.67 (-2.12 to -1.21 ) | <.001 | 24 (16 to 31 ) | 17 (3 to 39 ) | -1.82 (-2.37 to -1.28 ) | <.001 | 24 (16 to 32 ) | 17 (3 to 40 ) | -1.77 (-2.35 to -1.19 ) | <.001 |
| Xining | 22 (14 to 30 ) | 14 (3 to 35 ) | -2.31 (-2.65 to -1.97 ) | <.001 | 21 (13 to 29 ) | 14 (2 to 33 ) | -2.41 (-2.83 to -1.99 ) | <.001 | 22 (14 to 29 ) | 14 (2 to 34 ) | -2.34 (-2.67 to -2.01 ) | <.001 |
| Shaanxi |  |  |  |  |  |  |  |  |  |  |  |  |
| Ankang | 18 (12 to 24 ) | 12 (2 to 28 ) | -2.05 (-3.98 to -0.08 ) | 0.042 | 17 (11 to 23 ) | 11 (2 to 26 ) | -2.11 (-4.07 to -0.12 ) | 0.038 | 18 (12 to 24 ) | 11 (2 to 27 ) | -2.08 (-4.02 to -0.10 ) | 0.040 |
| Baoji | 20 (13 to 27 ) | 12 (2 to 28 ) | -2.82 (-4.58 to -1.03 ) | 0.002 | 20 (13 to 26 ) | 12 (2 to 26 ) | -2.87 (-4.66 to -1.06 ) | 0.002 | 20 (13 to 27 ) | 12 (2 to 27 ) | -2.84 (-4.61 to -1.04 ) | 0.002 |
| Hanzhong | 22 (15 to 28 ) | 13 (3 to 29 ) | -2.70 (-4.69 to -0.67 ) | 0.009 | 21 (15 to 27 ) | 12 (3 to 27 ) | -2.73 (-4.73 to -0.69 ) | 0.009 | 22 (15 to 28 ) | 13 (3 to 28 ) | -2.71 (-4.70 to -0.68 ) | 0.009 |
| Shangluo | 14 (9 to 21 ) | 11 (2 to 27 ) | -1.08 (-2.71 to 0.58 ) | 0.201 | 14 (9 to 20 ) | 10 (2 to 26 ) | -1.15 (-2.74 to 0.47 ) | 0.164 | 14 (9 to 20 ) | 11 (2 to 26 ) | -1.11 (-2.73 to 0.53 ) | 0.183 |
| Tongchuan | 12 (7 to 19 ) | 8 (1 to 23 ) | -2.09 (-4.42 to 0.29 ) | 0.084 | 12 (7 to 18 ) | 8 (1 to 22 ) | -2.16 (-4.46 to 0.19 ) | 0.072 | 12 (7 to 18 ) | 8 (1 to 22 ) | -2.12 (-4.44 to 0.24 ) | 0.078 |
| Weinan | 15 (10 to 22 ) | 11 (2 to 27 ) | -1.76 (-3.26 to -0.24 ) | 0.023 | 15 (9 to 21 ) | 10 (2 to 26 ) | -1.82 (-3.29 to -0.32 ) | 0.018 | 15 (10 to 22 ) | 11 (2 to 26 ) | -1.79 (-3.27 to -0.28 ) | 0.021 |
| Xi'an | 15 (9 to 22 ) | 10 (2 to 26 ) | -1.87 (-2.57 to -1.16 ) | <.001 | 14 (9 to 21 ) | 10 (2 to 24 ) | -1.93 (-2.65 to -1.20 ) | <.001 | 15 (9 to 21 ) | 10 (2 to 25 ) | -1.90 (-2.61 to -1.18 ) | <.001 |
| Xianyang | 17 (11 to 24 ) | 11 (2 to 26 ) | -2.25 (-2.96 to -1.54 ) | <.001 | 17 (10 to 23 ) | 11 (2 to 25 ) | -2.41 (-2.86 to -1.95 ) | <.001 | 17 (10 to 24 ) | 11 (2 to 26 ) | -2.37 (-2.82 to -1.91 ) | <.001 |
| Yan'an | 17 (11 to 24 ) | 10 (2 to 25 ) | -2.81 (-4.76 to -0.81 ) | 0.006 | 16 (10 to 23 ) | 9 (2 to 24 ) | -2.84 (-4.78 to -0.87 ) | 0.005 | 16 (11 to 23 ) | 10 (2 to 25 ) | -2.82 (-4.77 to -0.84 ) | 0.005 |
| Yulin | 17 (12 to 24 ) | 9 (2 to 25 ) | -3.58 (-5.67 to -1.43 ) | 0.001 | 17 (11 to 23 ) | 9 (2 to 24 ) | -3.63 (-5.71 to -1.50 ) | 0.001 | 17 (11 to 23 ) | 9 (2 to 24 ) | -3.60 (-5.69 to -1.47 ) | 0.001 |
| Shandong |  |  |  |  |  |  |  |  |  |  |  |  |
| Binzhou | 16 (12 to 20 ) | 10 (1 to 26 ) | -2.65 (-3.47 to -1.81 ) | <.001 | 15 (12 to 19 ) | 9 (1 to 25 ) | -2.71 (-3.54 to -1.87 ) | <.001 | 16 (12 to 19 ) | 9 (1 to 25 ) | -2.67 (-3.50 to -1.84 ) | <.001 |
| Dezhou | 15 (12 to 18 ) | 9 (1 to 25 ) | -2.61 (-3.40 to -1.80 ) | <.001 | 14 (11 to 18 ) | 9 (1 to 23 ) | -2.67 (-3.46 to -1.87 ) | <.001 | 15 (12 to 18 ) | 9 (1 to 24 ) | -2.63 (-3.43 to -1.83 ) | <.001 |
| Dongying | 14 (11 to 19 ) | 8 (1 to 25 ) | -2.92 (-3.70 to -2.14 ) | <.001 | 14 (11 to 18 ) | 8 (1 to 24 ) | -3.00 (-3.77 to -2.22 ) | <.001 | 14 (11 to 18 ) | 8 (1 to 24 ) | -2.96 (-3.73 to -2.18 ) | <.001 |
| Heze | 18 (14 to 23 ) | 9 (1 to 23 ) | -3.87 (-5.71 to -1.99 ) | <.001 | 17 (13 to 22 ) | 9 (1 to 22 ) | -3.90 (-5.70 to -2.06 ) | <.001 | 18 (14 to 22 ) | 9 (1 to 23 ) | -3.88 (-5.71 to -2.02 ) | <.001 |
| Jinan | 12 (9 to 15 ) | 7 (1 to 19 ) | -2.91 (-3.33 to -2.48 ) | <.001 | 11 (9 to 14 ) | 6 (1 to 18 ) | -2.95 (-3.39 to -2.51 ) | <.001 | 11 (9 to 14 ) | 7 (1 to 19 ) | -2.93 (-3.36 to -2.50 ) | <.001 |
| Jining | 18 (14 to 22 ) | 9 (1 to 24 ) | -3.62 (-4.23 to -3.01 ) | <.001 | 17 (13 to 22 ) | 9 (1 to 23 ) | -3.70 (-4.31 to -3.10 ) | <.001 | 18 (14 to 22 ) | 9 (1 to 24 ) | -3.66 (-4.26 to -3.05 ) | <.001 |
| Laiwu | 14 (11 to 17 ) | 8 (1 to 22 ) | -3.29 (-4.48 to -2.09 ) | <.001 | 14 (11 to 17 ) | 7 (1 to 21 ) | -3.33 (-4.46 to -2.17 ) | <.001 | 14 (11 to 17 ) | 8 (1 to 22 ) | -3.31 (-4.47 to -2.13 ) | <.001 |
| Liaocheng | 17 (14 to 21 ) | 9 (1 to 24 ) | -3.29 (-5.14 to -1.40 ) | 0.001 | 17 (13 to 20 ) | 9 (1 to 23 ) | -3.32 (-5.18 to -1.42 ) | 0.001 | 17 (14 to 21 ) | 9 (1 to 23 ) | -3.30 (-5.16 to -1.41 ) | 0.001 |
| Linyi | 14 (11 to 18 ) | 8 (1 to 23 ) | -2.94 (-4.21 to -1.65 ) | <.001 | 14 (11 to 17 ) | 8 (1 to 22 ) | -3.01 (-4.25 to -1.76 ) | <.001 | 14 (11 to 17 ) | 8 (1 to 23 ) | -2.97 (-4.23 to -1.70 ) | <.001 |
| Qingdao | 8 (5 to 11 ) | 5 (1 to 18 ) | -2.04 (-3.17 to -0.90 ) | 0.001 | 8 (5 to 11 ) | 5 (0 to 17 ) | -2.11 (-3.27 to -0.93 ) | <.001 | 8 (5 to 11 ) | 5 (0 to 17 ) | -2.07 (-3.21 to -0.91 ) | <.001 |
| Rizhao | 13 (9 to 17 ) | 7 (1 to 21 ) | -2.91 (-4.21 to -1.60 ) | <.001 | 12 (9 to 16 ) | 7 (1 to 20 ) | -2.98 (-4.25 to -1.69 ) | <.001 | 12 (9 to 16 ) | 7 (1 to 21 ) | -2.94 (-4.23 to -1.64 ) | <.001 |
| Tai'an | 16 (12 to 19 ) | 8 (1 to 22 ) | -3.14 (-3.96 to -2.32 ) | <.001 | 15 (12 to 18 ) | 8 (1 to 21 ) | -3.28 (-4.06 to -2.50 ) | <.001 | 15 (12 to 19 ) | 8 (1 to 22 ) | -3.24 (-4.01 to -2.45 ) | <.001 |
| Weifang | 14 (11 to 18 ) | 8 (1 to 23 ) | -2.97 (-3.78 to -2.16 ) | <.001 | 14 (10 to 18 ) | 8 (1 to 22 ) | -3.02 (-3.84 to -2.19 ) | <.001 | 14 (11 to 18 ) | 8 (1 to 23 ) | -3.0 (-3.81 to -2.17 ) | <.001 |
| Weihai | 7 (4 to 10 ) | 6 (1 to 21 ) | -0.87 (-2.73 to 1.02 ) | 0.365 | 7 (4 to 10 ) | 6 (0 to 20 ) | -0.87 (-2.14 to 0.42 ) | 0.183 | 7 (4 to 10 ) | 6 (1 to 21 ) | -0.85 (-2.12 to 0.44 ) | 0.197 |
| Yantai | 9 (6 to 13 ) | 7 (1 to 23 ) | -1.45 (-2.72 to -0.17 ) | 0.027 | 9 (6 to 13 ) | 7 (1 to 22 ) | -1.50 (-2.77 to -0.20 ) | 0.023 | 9 (6 to 13 ) | 7 (1 to 23 ) | -1.47 (-2.74 to -0.19 ) | 0.025 |
| Zaozhuang | 14 (11 to 18 ) | 8 (1 to 22 ) | -2.84 (-4.16 to -1.50 ) | <.001 | 14 (10 to 17 ) | 8 (1 to 21 ) | -2.92 (-3.99 to -1.84 ) | <.001 | 14 (10 to 17 ) | 8 (1 to 21 ) | -2.87 (-4.19 to -1.53 ) | <.001 |
| Zibo | 14 (11 to 18 ) | 8 (1 to 22 ) | -3.10 (-3.81 to -2.38 ) | <.001 | 13 (10 to 17 ) | 7 (1 to 21 ) | -3.14 (-3.84 to -2.43 ) | <.001 | 14 (11 to 17 ) | 8 (1 to 22 ) | -3.12 (-3.82 to -2.41 ) | <.001 |
| Shanghai |  |  |  |  |  |  |  |  |  |  |  |  |
| Shanghai | 10 (6 to 16 ) | 9 (1 to 31 ) | -0.70 (-1.04 to -0.36 ) | <.001 | 10 (6 to 15 ) | 8 (1 to 30 ) | -0.74 (-1.14 to -0.33 ) | <.001 | 10 (6 to 15 ) | 9 (1 to 30 ) | -0.72 (-1.08 to -0.35 ) | <.001 |
| Shanxi |  |  |  |  |  |  |  |  |  |  |  |  |
| Changzhi | 15 (10 to 20 ) | 11 (2 to 28 ) | -1.18 (-3.36 to 1.05 ) | 0.298 | 14 (10 to 20 ) | 11 (2 to 27 ) | -1.23 (-3.41 to 1.00 ) | 0.276 | 14 (10 to 20 ) | 11 (2 to 28 ) | -1.20 (-3.38 to 1.03 ) | 0.288 |
| Datong | 18 (14 to 23 ) | 10 (2 to 29 ) | -3.16 (-4.50 to -1.80 ) | <.001 | 17 (13 to 22 ) | 10 (1 to 28 ) | -3.35 (-4.77 to -1.92 ) | <.001 | 18 (13 to 22 ) | 10 (2 to 29 ) | -3.18 (-4.50 to -1.84 ) | <.001 |
| Jincheng | 12 (8 to 16 ) | 9 (1 to 25 ) | -0.72 (-4.24 to 2.94 ) | 0.696 | 11 (7 to 16 ) | 9 (1 to 23 ) | -0.77 (-4.30 to 2.89 ) | 0.675 | 11 (7 to 16 ) | 9 (1 to 24 ) | -0.74 (-4.27 to 2.92 ) | 0.686 |
| Jinzhong | 18 (13 to 24 ) | 13 (2 to 30 ) | -2.02 (-2.69 to -1.34 ) | <.001 | 17 (13 to 23 ) | 12 (2 to 29 ) | -2.07 (-2.71 to -1.42 ) | <.001 | 18 (13 to 24 ) | 12 (2 to 30 ) | -2.04 (-2.70 to -1.38 ) | <.001 |
| Linfen | 18 (11 to 25 ) | 12 (2 to 29 ) | -2.31 (-3.00 to -1.62 ) | <.001 | 17 (11 to 24 ) | 11 (2 to 28 ) | -2.36 (-3.02 to -1.69 ) | <.001 | 17 (11 to 24 ) | 11 (2 to 29 ) | -2.33 (-3.01 to -1.65 ) | <.001 |
| Luliang | 20 (14 to 27 ) | 12 (2 to 30 ) | -2.60 (-3.94 to -1.25 ) | <.001 | 19 (13 to 26 ) | 11 (2 to 28 ) | -2.90 (-3.70 to -2.09 ) | <.001 | 20 (14 to 26 ) | 12 (2 to 29 ) | -2.87 (-3.69 to -2.04 ) | <.001 |
| Shuozhou | 21 (16 to 26 ) | 12 (2 to 31 ) | -3.33 (-4.82 to -1.82 ) | <.001 | 20 (15 to 25 ) | 11 (2 to 29 ) | -3.38 (-4.82 to -1.92 ) | <.001 | 20 (15 to 26 ) | 11 (2 to 30 ) | -3.36 (-4.82 to -1.87 ) | <.001 |
| Taiyuan | 14 (10 to 19 ) | 10 (2 to 26 ) | -2.22 (-2.63 to -1.81 ) | <.001 | 14 (10 to 19 ) | 9 (1 to 25 ) | -2.27 (-2.65 to -1.89 ) | <.001 | 14 (10 to 19 ) | 9 (2 to 25 ) | -2.25 (-2.64 to -1.85 ) | <.001 |
| Xinzhou | 19 (14 to 25 ) | 12 (2 to 31 ) | -2.79 (-4.05 to -1.51 ) | <.001 | 19 (14 to 24 ) | 11 (2 to 29 ) | -2.95 (-3.94 to -1.96 ) | <.001 | 19 (14 to 24 ) | 12 (2 to 30 ) | -2.81 (-4.05 to -1.55 ) | <.001 |
| Yangquan | 13 (10 to 17 ) | 10 (2 to 27 ) | -1.52 (-3.39 to 0.39 ) | 0.118 | 13 (9 to 17 ) | 10 (2 to 26 ) | -1.56 (-3.42 to 0.33 ) | 0.105 | 13 (9 to 17 ) | 10 (2 to 27 ) | -1.54 (-3.40 to 0.36 ) | 0.112 |
| Yuncheng | 18 (11 to 25 ) | 12 (2 to 30 ) | -1.87 (-3.91 to 0.20 ) | 0.077 | 17 (11 to 25 ) | 12 (2 to 29 ) | -1.95 (-3.98 to 0.13 ) | 0.066 | 17 (11 to 25 ) | 12 (2 to 30 ) | -1.91 (-3.94 to 0.17 ) | 0.071 |
| Sichuan* |  |  |  |  |  |  |  |  |  |  |  |  |
| Bazhong | 25 (18 to 33 ) | 10 (2 to 23 ) | -4.75 (-5.42 to -4.07 ) | <.001 | 25 (17 to 32 ) | 10 (2 to 22 ) | -4.87 (-5.62 to -4.11 ) | <.001 | 25 (17 to 32 ) | 10 (2 to 23 ) | -4.84 (-5.61 to -4.07 ) | <.001 |
| Chengdu | 21 (13 to 29 ) | 8 (1 to 18 ) | -5.25 (-6.09 to -4.40 ) | <.001 | 20 (13 to 28 ) | 7 (1 to 17 ) | -5.28 (-6.07 to -4.49 ) | <.001 | 21 (13 to 28 ) | 8 (1 to 18 ) | -5.27 (-6.09 to -4.44 ) | <.001 |
| Dazhou | 23 (17 to 30 ) | 10 (2 to 23 ) | -4.38 (-5.13 to -3.61 ) | <.001 | 23 (16 to 29 ) | 9 (2 to 22 ) | -4.45 (-5.20 to -3.70 ) | <.001 | 23 (17 to 29 ) | 10 (2 to 22 ) | -4.41 (-5.16 to -3.65 ) | <.001 |
| Deyang | 24 (16 to 31 ) | 9 (2 to 21 ) | -5.04 (-6.85 to -3.21 ) | <.001 | 23 (15 to 30 ) | 9 (2 to 20 ) | -5.09 (-6.87 to -3.28 ) | <.001 | 23 (15 to 31 ) | 9 (2 to 21 ) | -5.07 (-6.86 to -3.24 ) | <.001 |
| Garzê Tibetan | 28 (17 to 37 ) | 10 (2 to 23 ) | -5.45 (-9.31 to -1.43 ) | 0.008 | 27 (17 to 36 ) | 10 (2 to 22 ) | -5.40 (-9.89 to -0.69 ) | 0.025 | 28 (17 to 37 ) | 10 (2 to 23 ) | -5.37 (-9.84 to -0.67 ) | 0.026 |
| Guang'an | 22 (15 to 28 ) | 9 (2 to 22 ) | -4.39 (-5.88 to -2.87 ) | <.001 | 21 (15 to 27 ) | 9 (2 to 21 ) | -4.46 (-5.95 to -2.95 ) | <.001 | 21 (15 to 28 ) | 9 (2 to 21 ) | -4.42 (-5.91 to -2.90 ) | <.001 |
| Guangyuan | 24 (16 to 31 ) | 10 (2 to 23 ) | -4.68 (-5.66 to -3.68 ) | <.001 | 23 (15 to 30 ) | 9 (2 to 21 ) | -4.74 (-5.72 to -3.75 ) | <.001 | 23 (15 to 31 ) | 9 (2 to 22 ) | -4.70 (-5.69 to -3.71 ) | <.001 |
| Leshan | 20 (13 to 27 ) | 8 (2 to 20 ) | -4.74 (-6.00 to -3.46 ) | <.001 | 20 (13 to 26 ) | 8 (1 to 19 ) | -4.82 (-6.13 to -3.49 ) | <.001 | 20 (13 to 27 ) | 8 (1 to 19 ) | -4.77 (-6.06 to -3.48 ) | <.001 |
| Liangshan Yi | 25 (18 to 33 ) | 10 (2 to 23 ) | -4.95 (-6.60 to -3.28 ) | <.001 | 24 (17 to 32 ) | 9 (2 to 21 ) | -5.03 (-6.65 to -3.38 ) | <.001 | 25 (17 to 32 ) | 9 (2 to 22 ) | -4.99 (-6.62 to -3.33 ) | <.001 |
| Luzhou | 20 (15 to 25 ) | 8 (1 to 20 ) | -4.53 (-7.40 to -1.57 ) | 0.003 | 19 (15 to 24 ) | 7 (1 to 19 ) | -4.59 (-7.48 to -1.61 ) | 0.003 | 19 (15 to 24 ) | 8 (1 to 20 ) | -4.56 (-7.44 to -1.59 ) | 0.003 |
| Meishan | 21 (14 to 29 ) | 9 (2 to 20 ) | -4.66 (-5.56 to -3.74 ) | <.001 | 21 (14 to 28 ) | 8 (2 to 19 ) | -5.04 (-5.69 to -4.39 ) | <.001 | 21 (14 to 29 ) | 8 (2 to 20 ) | -5.02 (-5.71 to -4.32 ) | <.001 |
| Mianyang | 21 (14 to 30 ) | 8 (2 to 20 ) | -4.97 (-6.14 to -3.79 ) | <.001 | 21 (13 to 29 ) | 8 (1 to 19 ) | -5.16 (-5.90 to -4.42 ) | <.001 | 21 (13 to 29 ) | 8 (2 to 20 ) | -5.0 (-6.15 to -3.83 ) | <.001 |
| Nanchong | 22 (15 to 29 ) | 9 (2 to 21 ) | -4.83 (-5.22 to -4.45 ) | <.001 | 22 (15 to 28 ) | 8 (2 to 20 ) | -4.91 (-5.27 to -4.54 ) | <.001 | 22 (15 to 29 ) | 9 (2 to 21 ) | -4.87 (-5.24 to -4.49 ) | <.001 |
| Ngawa Tibetan and Qiang | 28 (19 to 36 ) | 11 (3 to 24 ) | -5.11 (-8.77 to -1.31 ) | 0.009 | 27 (19 to 35 ) | 11 (2 to 23 ) | -5.16 (-8.73 to -1.46 ) | 0.007 | 28 (19 to 36 ) | 11 (2 to 23 ) | -5.14 (-8.75 to -1.38 ) | 0.008 |
| Panzhihua | 20 (12 to 28 ) | 7 (1 to 19 ) | -5.17 (-7.00 to -3.31 ) | <.001 | 19 (12 to 27 ) | 7 (1 to 18 ) | -5.70 (-7.01 to -4.36 ) | <.001 | 20 (12 to 28 ) | 7 (1 to 19 ) | -5.67 (-7.01 to -4.31 ) | <.001 |
| Suining | 21 (14 to 28 ) | 8 (1 to 20 ) | -5.08 (-5.84 to -4.30 ) | <.001 | 20 (13 to 27 ) | 7 (1 to 19 ) | -5.16 (-5.92 to -4.40 ) | <.001 | 21 (14 to 28 ) | 8 (1 to 19 ) | -5.12 (-5.88 to -4.35 ) | <.001 |
| Ya'an | 25 (16 to 33 ) | 10 (2 to 22 ) | -5.23 (-8.03 to -2.34 ) | <.001 | 24 (16 to 32 ) | 9 (2 to 21 ) | -5.29 (-8.06 to -2.43 ) | <.001 | 24 (16 to 32 ) | 9 (2 to 22 ) | -5.26 (-8.04 to -2.38 ) | <.001 |
| Yibin | 19 (14 to 25 ) | 7 (1 to 19 ) | -4.99 (-6.61 to -3.34 ) | <.001 | 19 (14 to 24 ) | 7 (1 to 18 ) | -5.04 (-6.66 to -3.39 ) | <.001 | 19 (14 to 25 ) | 7 (1 to 19 ) | -5.01 (-6.63 to -3.36 ) | <.001 |
| Zigong | 18 (12 to 24 ) | 7 (1 to 18 ) | -4.78 (-6.85 to -2.66 ) | <.001 | 17 (12 to 23 ) | 6 (1 to 17 ) | -4.83 (-6.95 to -2.66 ) | <.001 | 17 (12 to 24 ) | 7 (1 to 18 ) | -4.80 (-6.90 to -2.66 ) | <.001 |
| Ziyang | 20 (13 to 27 ) | 8 (2 to 20 ) | -4.76 (-5.90 to -3.60 ) | <.001 | 19 (13 to 26 ) | 8 (1 to 19 ) | -4.83 (-5.98 to -3.66 ) | <.001 | 20 (13 to 27 ) | 8 (1 to 19 ) | -4.79 (-5.93 to -3.63 ) | <.001 |
| Tianjin |  |  |  |  |  |  |  |  |  |  |  |  |
| Tianjin | 11 (9 to 14 ) | 8 (1 to 26 ) | -1.68 (-1.80 to -1.57 ) | <.001 | 11 (8 to 14 ) | 8 (1 to 25 ) | -1.73 (-1.80 to -1.67 ) | <.001 | 11 (9 to 14 ) | 8 (1 to 26 ) | -1.70 (-1.79 to -1.61 ) | <.001 |
| Xinjiang |  |  |  |  |  |  |  |  |  |  |  |  |
| Aksu | 22 (6 to 35 ) | 14 (1 to 47 ) | -2.13 (-2.89 to -1.37 ) | <.001 | 21 (6 to 34 ) | 14 (1 to 44 ) | -2.20 (-2.93 to -1.46 ) | <.001 | 21 (6 to 35 ) | 14 (1 to 46 ) | -2.16 (-2.90 to -1.41 ) | <.001 |
| Altay | 20 (13 to 28 ) | 17 (2 to 47 ) | -0.88 (-3.17 to 1.47 ) | 0.460 | 20 (13 to 27 ) | 16 (2 to 45 ) | -0.96 (-3.25 to 1.38 ) | 0.417 | 20 (13 to 27 ) | 17 (2 to 46 ) | -0.92 (-3.20 to 1.43 ) | 0.440 |
| Bayin'gholin Mongol | 18 (8 to 30 ) | 15 (2 to 43 ) | -1.05 (-2.08 to -0.01 ) | 0.048 | 17 (7 to 29 ) | 14 (2 to 41 ) | -1.11 (-2.17 to -0.04 ) | 0.042 | 18 (8 to 30 ) | 14 (2 to 42 ) | -1.08 (-2.12 to -0.02 ) | 0.045 |
| Bortala Mongol | 22 (7 to 35 ) | 16 (2 to 52 ) | -1.68 (-2.51 to -0.84 ) | <.001 | 21 (7 to 34 ) | 15 (2 to 49 ) | -1.75 (-2.58 to -0.92 ) | <.001 | 22 (7 to 35 ) | 16 (2 to 50 ) | -1.71 (-2.54 to -0.88 ) | <.001 |
| Changji Hui | 17 (8 to 27 ) | 14 (2 to 44 ) | -0.97 (-2.58 to 0.66 ) | 0.241 | 17 (8 to 26 ) | 14 (2 to 42 ) | -1.02 (-2.60 to 0.60 ) | 0.216 | 17 (8 to 26 ) | 14 (2 to 43 ) | -0.99 (-2.59 to 0.63 ) | 0.229 |
| Hami | 17 (11 to 24 ) | 14 (2 to 39 ) | -0.66 (-2.48 to 1.19 ) | 0.482 | 16 (10 to 23 ) | 13 (2 to 37 ) | -0.72 (-2.55 to 1.16 ) | 0.451 | 17 (10 to 23 ) | 13 (2 to 38 ) | -0.69 (-2.51 to 1.18 ) | 0.467 |
| Ili Kazakh | 22 (7 to 35 ) | 15 (2 to 50 ) | -2.10 (-2.53 to -1.67 ) | <.001 | 22 (7 to 34 ) | 14 (2 to 48 ) | -2.18 (-2.61 to -1.75 ) | <.001 | 22 (7 to 35 ) | 15 (2 to 49 ) | -2.14 (-2.57 to -1.70 ) | <.001 |
| Karamay | 15 (5 to 28 ) | 10 (1 to 35 ) | -2.24 (-4.91 to 0.52 ) | 0.111 | 14 (5 to 27 ) | 9 (1 to 34 ) | -2.79 (-5.20 to -0.31 ) | 0.028 | 15 (5 to 28 ) | 9 (1 to 35 ) | -2.28 (-4.95 to 0.47 ) | 0.103 |
| Kashgar | 22 (3 to 37 ) | 13 (1 to 46 ) | -2.65 (-3.65 to -1.63 ) | <.001 | 21 (3 to 36 ) | 13 (1 to 43 ) | -2.82 (-3.82 to -1.80 ) | <.001 | 21 (3 to 36 ) | 13 (1 to 45 ) | -2.69 (-3.69 to -1.67 ) | <.001 |
| Khotan | 22 (5 to 36 ) | 15 (1 to 46 ) | -2.06 (-3.17 to -0.93 ) | <.001 | 21 (4 to 35 ) | 15 (1 to 44 ) | -2.12 (-3.19 to -1.03 ) | <.001 | 22 (5 to 36 ) | 15 (1 to 45 ) | -2.08 (-3.18 to -0.98 ) | <.001 |
| Kizilsu Kirghiz | 20 (3 to 37 ) | 11 (0 to 43 ) | -3.21 (-3.99 to -2.42 ) | <.001 | 19 (2 to 35 ) | 10 (0 to 40 ) | -3.30 (-4.06 to -2.52 ) | <.001 | 19 (3 to 36 ) | 10 (0 to 42 ) | -3.25 (-4.03 to -2.47 ) | <.001 |
| Shihezi | 17 (7 to 29 ) | 12 (1 to 40 ) | -1.50 (-4.28 to 1.37 ) | 0.302 | 16 (7 to 28 ) | 12 (1 to 38 ) | -1.96 (-3.48 to -0.40 ) | 0.014 | 16 (7 to 28 ) | 12 (1 to 39 ) | -1.93 (-3.50 to -0.34 ) | 0.018 |
| Tacheng | 20 (8 to 32 ) | 15 (2 to 46 ) | -1.41 (-2.35 to -0.47 ) | 0.003 | 19 (7 to 31 ) | 15 (2 to 44 ) | -1.49 (-2.42 to -0.55 ) | 0.002 | 20 (8 to 32 ) | 15 (2 to 45 ) | -1.45 (-2.38 to -0.51 ) | 0.003 |
| Turfan | 17 (8 to 26 ) | 15 (2 to 44 ) | -0.56 (-2.38 to 1.30 ) | 0.555 | 16 (7 to 25 ) | 14 (2 to 42 ) | -0.64 (-2.47 to 1.23 ) | 0.499 | 16 (8 to 25 ) | 14 (2 to 43 ) | -0.59 (-2.42 to 1.27 ) | 0.529 |
| rümqi | 12 (5 to 21 ) | 9 (1 to 33 ) | -1.33 (-3.08 to 0.46 ) | 0.144 | 11 (4 to 20 ) | 9 (1 to 32 ) | -1.37 (-2.73 to 0.02 ) | 0.054 | 12 (4 to 21 ) | 9 (1 to 33 ) | -1.35 (-3.10 to 0.43 ) | 0.136 |
| Xizang |  |  |  |  |  |  |  |  |  |  |  |  |
| Chamdo | 29 (15 to 39 ) | 23 (4 to 57 ) | -1.08 (-2.76 to 0.63 ) | 0.214 | 28 (15 to 38 ) | 22 (4 to 54 ) | -1.16 (-2.80 to 0.51 ) | 0.171 | 28 (15 to 39 ) | 23 (4 to 56 ) | -1.12 (-2.78 to 0.57 ) | 0.194 |
| Lhasa | 26 (8 to 42 ) | 21 (2 to 58 ) | -1.24 (-1.66 to -0.82 ) | <.001 | 25 (8 to 40 ) | 20 (2 to 55 ) | -1.33 (-1.76 to -0.91 ) | <.001 | 26 (8 to 41 ) | 20 (2 to 57 ) | -1.28 (-1.70 to -0.86 ) | <.001 |
| Nagchu | 27 (11 to 41 ) | 21 (3 to 55 ) | -1.25 (-1.70 to -0.80 ) | <.001 | 27 (10 to 40 ) | 20 (3 to 52 ) | -1.34 (-1.90 to -0.76 ) | <.001 | 27 (11 to 41 ) | 21 (3 to 54 ) | -1.29 (-1.84 to -0.73 ) | <.001 |
| Ngari | 26 (5 to 44 ) | 22 (2 to 65 ) | -0.88 (-4.34 to 2.70 ) | 0.624 | 25 (5 to 42 ) | 21 (1 to 61 ) | -1.34 (-3.40 to 0.77 ) | 0.211 | 25 (5 to 43 ) | 21 (1 to 63 ) | -1.30 (-3.40 to 0.84 ) | 0.230 |
| Nyingtri | 28 (12 to 40 ) | 25 (4 to 62 ) | -0.56 (-1.21 to 0.10 ) | 0.097 | 27 (12 to 39 ) | 24 (4 to 59 ) | -0.65 (-1.33 to 0.04 ) | 0.064 | 28 (12 to 40 ) | 25 (4 to 60 ) | -0.60 (-1.26 to 0.07 ) | 0.080 |
| Shannan | 26 (8 to 42 ) | 22 (2 to 62 ) | -0.88 (-1.20 to -0.57 ) | <.001 | 25 (8 to 40 ) | 21 (2 to 59 ) | -0.98 (-1.29 to -0.66 ) | <.001 | 26 (8 to 41 ) | 22 (2 to 61 ) | -0.93 (-1.24 to -0.61 ) | <.001 |
| Shigatse | 27 (7 to 43 ) | 22 (2 to 62 ) | -1.17 (-2.47 to 0.14 ) | 0.080 | 26 (7 to 42 ) | 21 (2 to 59 ) | -1.48 (-2.45 to -0.49 ) | 0.003 | 27 (7 to 43 ) | 21 (2 to 61 ) | -1.22 (-2.50 to 0.09 ) | 0.068 |
| Yunnan |  |  |  |  |  |  |  |  |  |  |  |  |
| Baoshan | 28 (13 to 43 ) | 14 (2 to 31 ) | -3.75 (-5.00 to -2.49 ) | <.001 | 27 (13 to 41 ) | 13 (2 to 30 ) | -3.82 (-4.93 to -2.69 ) | <.001 | 27 (13 to 42 ) | 13 (2 to 30 ) | -3.78 (-4.97 to -2.58 ) | <.001 |
| Chuxiong Yi | 24 (15 to 34 ) | 11 (2 to 28 ) | -3.93 (-4.85 to -3.01 ) | <.001 | 24 (14 to 33 ) | 11 (2 to 26 ) | -4.01 (-4.93 to -3.09 ) | <.001 | 24 (15 to 34 ) | 11 (2 to 27 ) | -3.97 (-4.89 to -3.04 ) | <.001 |
| Dali Bai | 26 (14 to 39 ) | 12 (2 to 28 ) | -4.17 (-5.15 to -3.19 ) | <.001 | 25 (13 to 37 ) | 11 (2 to 27 ) | -4.24 (-5.15 to -3.32 ) | <.001 | 25 (13 to 38 ) | 11 (2 to 28 ) | -4.20 (-5.14 to -3.25 ) | <.001 |
| Dehong Dai and Jingpo | 29 (14 to 45 ) | 15 (3 to 33 ) | -3.60 (-5.44 to -1.73 ) | <.001 | 28 (14 to 44 ) | 14 (3 to 31 ) | -3.67 (-5.39 to -1.91 ) | <.001 | 29 (14 to 45 ) | 15 (3 to 32 ) | -3.63 (-5.42 to -1.81 ) | <.001 |
| Dêqên Tibetan | 33 (20 to 44 ) | 15 (3 to 31 ) | -4.42 (-7.34 to -1.40 ) | 0.004 | 32 (19 to 43 ) | 14 (2 to 30 ) | -4.48 (-7.27 to -1.61 ) | 0.002 | 33 (19 to 43 ) | 14 (3 to 31 ) | -4.45 (-7.30 to -1.50 ) | 0.003 |
| Honghe Hani and Yi | 25 (16 to 34 ) | 11 (2 to 28 ) | -4.10 (-5.12 to -3.08 ) | <.001 | 24 (16 to 33 ) | 11 (2 to 26 ) | -4.19 (-4.85 to -3.52 ) | <.001 | 24 (16 to 33 ) | 11 (2 to 27 ) | -4.12 (-5.14 to -3.10 ) | <.001 |
| Kunming | 21 (14 to 29 ) | 10 (2 to 24 ) | -4.02 (-5.19 to -2.83 ) | <.001 | 20 (13 to 28 ) | 9 (1 to 23 ) | -4.20 (-5.39 to -2.99 ) | <.001 | 21 (13 to 28 ) | 9 (2 to 24 ) | -4.17 (-5.36 to -2.96 ) | <.001 |
| Lijiang | 29 (18 to 40 ) | 13 (2 to 29 ) | -4.54 (-6.58 to -2.46 ) | <.001 | 28 (17 to 39 ) | 12 (2 to 28 ) | -4.60 (-6.45 to -2.72 ) | <.001 | 29 (18 to 40 ) | 12 (2 to 29 ) | -4.57 (-6.52 to -2.57 ) | <.001 |
| Lincang | 28 (15 to 42 ) | 14 (3 to 32 ) | -3.57 (-4.47 to -2.67 ) | <.001 | 27 (15 to 40 ) | 14 (2 to 30 ) | -3.64 (-4.48 to -2.79 ) | <.001 | 28 (15 to 41 ) | 14 (2 to 31 ) | -3.60 (-4.47 to -2.73 ) | <.001 |
| Nujiang Lisu | 33 (19 to 45 ) | 16 (3 to 33 ) | -3.98 (-6.21 to -1.71 ) | 0.001 | 32 (19 to 44 ) | 15 (3 to 31 ) | -4.05 (-6.20 to -1.84 ) | <.001 | 33 (19 to 45 ) | 15 (3 to 32 ) | -4.01 (-6.20 to -1.77 ) | 0.001 |
| Pu'er | 30 (18 to 41 ) | 15 (3 to 33 ) | -3.42 (-4.91 to -1.90 ) | <.001 | 29 (17 to 40 ) | 15 (3 to 31 ) | -3.49 (-4.99 to -1.96 ) | <.001 | 29 (18 to 41 ) | 15 (3 to 32 ) | -3.45 (-4.94 to -1.93 ) | <.001 |
| Qujing | 28 (22 to 33 ) | 12 (2 to 28 ) | -4.52 (-5.59 to -3.45 ) | <.001 | 27 (21 to 32 ) | 11 (2 to 26 ) | -4.61 (-5.68 to -3.52 ) | <.001 | 27 (22 to 33 ) | 11 (2 to 27 ) | -4.56 (-5.63 to -3.48 ) | <.001 |
| Wenshan Zhuang and Miao | 25 (19 to 32 ) | 10 (2 to 26 ) | -4.68 (-7.07 to -2.22 ) | <.001 | 25 (19 to 30 ) | 10 (2 to 25 ) | -4.75 (-7.07 to -2.37 ) | <.001 | 25 (19 to 31 ) | 10 (2 to 25 ) | -4.71 (-7.07 to -2.29 ) | <.001 |
| Xishuangbanna Dai | 34 (21 to 46 ) | 17 (4 to 34 ) | -3.61 (-6.12 to -1.05 ) | 0.006 | 33 (21 to 44 ) | 16 (3 to 33 ) | -3.69 (-6.22 to -1.08 ) | 0.006 | 34 (21 to 45 ) | 17 (3 to 34 ) | -3.65 (-6.16 to -1.07 ) | 0.006 |
| Yuxi | 23 (14 to 33 ) | 10 (2 to 26 ) | -4.12 (-4.91 to -3.33 ) | <.001 | 22 (14 to 32 ) | 10 (2 to 25 ) | -4.19 (-4.97 to -3.39 ) | <.001 | 23 (14 to 33 ) | 10 (2 to 26 ) | -4.15 (-4.94 to -3.36 ) | <.001 |
| Zhaotong | 28 (22 to 34 ) | 11 (2 to 26 ) | -4.84 (-7.31 to -2.30 ) | <.001 | 27 (21 to 33 ) | 10 (2 to 25 ) | -4.90 (-7.37 to -2.36 ) | <.001 | 28 (22 to 33 ) | 11 (2 to 26 ) | -4.87 (-7.34 to -2.33 ) | <.001 |
| Zhejiang |  |  |  |  |  |  |  |  |  |  |  |  |
| Hangzhou | 7 (4 to 10 ) | 3 (0 to 11 ) | -3.61 (-4.85 to -2.36 ) | <.001 | 6 (4 to 9 ) | 3 (0 to 10 ) | -3.69 (-4.93 to -2.44 ) | <.001 | 7 (4 to 10 ) | 3 (0 to 10 ) | -3.65 (-4.88 to -2.40 ) | <.001 |
| Huzhou | 6 (4 to 9 ) | 4 (0 to 11 ) | -3.53 (-5.12 to -1.91 ) | <.001 | 6 (4 to 9 ) | 3 (0 to 11 ) | -3.57 (-5.12 to -1.98 ) | <.001 | 6 (4 to 9 ) | 3 (0 to 11 ) | -3.55 (-5.12 to -1.94 ) | <.001 |
| Jiaxing | 7 (5 to 11 ) | 4 (0 to 11 ) | -4.01 (-6.11 to -1.86 ) | <.001 | 7 (5 to 10 ) | 3 (0 to 11 ) | -4.05 (-6.12 to -1.93 ) | <.001 | 7 (5 to 10 ) | 4 (0 to 11 ) | -4.03 (-6.12 to -1.89 ) | <.001 |
| Jinhua | 10 (6 to 15 ) | 5 (1 to 14 ) | -3.95 (-4.90 to -2.99 ) | <.001 | 10 (6 to 14 ) | 4 (0 to 13 ) | -4.23 (-4.88 to -3.58 ) | <.001 | 10 (6 to 15 ) | 5 (0 to 13 ) | -3.99 (-4.95 to -3.02 ) | <.001 |
| Lishui | 11 (7 to 16 ) | 5 (1 to 14 ) | -3.66 (-5.12 to -2.17 ) | <.001 | 10 (6 to 16 ) | 5 (1 to 13 ) | -3.82 (-5.49 to -2.13 ) | <.001 | 11 (6 to 16 ) | 5 (1 to 14 ) | -3.68 (-5.14 to -2.19 ) | <.001 |
| Ningbo | 8 (5 to 12 ) | 4 (0 to 12 ) | -4.53 (-6.12 to -2.91 ) | <.001 | 8 (5 to 12 ) | 3 (0 to 11 ) | -4.57 (-6.14 to -2.98 ) | <.001 | 8 (5 to 12 ) | 4 (0 to 11 ) | -4.55 (-6.13 to -2.95 ) | <.001 |
| Quzhou | 11 (7 to 16 ) | 6 (1 to 15 ) | -3.42 (-4.88 to -1.94 ) | <.001 | 11 (7 to 15 ) | 5 (1 to 14 ) | -3.46 (-4.92 to -1.97 ) | <.001 | 11 (7 to 16 ) | 6 (1 to 15 ) | -3.44 (-4.90 to -1.96 ) | <.001 |
| Shaoxing | 9 (5 to 13 ) | 4 (0 to 12 ) | -4.06 (-4.97 to -3.14 ) | <.001 | 8 (5 to 12 ) | 4 (0 to 12 ) | -4.11 (-4.98 to -3.22 ) | <.001 | 8 (5 to 12 ) | 4 (0 to 12 ) | -4.08 (-4.97 to -3.18 ) | <.001 |
| Taizhou | 9 (5 to 14 ) | 4 (0 to 12 ) | -4.47 (-4.88 to -4.06 ) | <.001 | 8 (5 to 13 ) | 3 (0 to 11 ) | -4.53 (-4.94 to -4.12 ) | <.001 | 9 (5 to 14 ) | 4 (0 to 11 ) | -4.50 (-4.91 to -4.09 ) | <.001 |
| Wenzhou | 8 (4 to 14 ) | 4 (0 to 11 ) | -4.13 (-5.00 to -3.25 ) | <.001 | 8 (4 to 13 ) | 3 (0 to 11 ) | -4.56 (-5.40 to -3.70 ) | <.001 | 8 (4 to 14 ) | 3 (0 to 11 ) | -4.51 (-5.36 to -3.66 ) | <.001 |
| Zhoushan | 6 (3 to 9 ) | 3 (0 to 9 ) | -3.83 (-5.88 to -1.74 ) | <.001 | 5 (3 to 9 ) | 2 (0 to 9 ) | -4.22 (-5.89 to -2.52 ) | <.001 | 5 (3 to 9 ) | 3 (0 to 9 ) | -3.87 (-5.92 to -1.78 ) | <.001 |
| Hong Kong |  |  |  |  |  |  |  |  |  |  |  |  |
| Central and Western | 4 (2 to 7 ) | 4 (0 to 15 ) | 0.26 (-2.76 to 3.37 ) | 0.870 | 4 (2 to 7 ) | 4 (0 to 14 ) | 0.18 (-2.92 to 3.39 ) | 0.909 | 4 (2 to 7 ) | 4 (0 to 14 ) | 0.22 (-2.83 to 3.37 ) | 0.888 |
| Eastern | 4 (2 to 8 ) | 3 (0 to 12 ) | -1.72 (-4.19 to 0.81 ) | 0.180 | 4 (2 to 8 ) | 3 (0 to 11 ) | -1.80 (-4.19 to 0.66 ) | 0.150 | 4 (2 to 8 ) | 3 (0 to 12 ) | -1.76 (-4.19 to 0.74 ) | 0.166 |
| Islands | 6 (3 to 9 ) | 4 (0 to 15 ) | -1.44 (-2.11 to -0.77 ) | <.001 | 5 (3 to 9 ) | 4 (0 to 14 ) | -1.49 (-2.13 to -0.84 ) | <.001 | 6 (3 to 9 ) | 4 (0 to 14 ) | -1.46 (-2.12 to -0.80 ) | <.001 |
| Kowloon City | 4 (2 to 6 ) | 3 (0 to 11 ) | -1.05 (-2.20 to 0.12 ) | 0.079 | 3 (2 to 6 ) | 3 (0 to 11 ) | -1.11 (-2.18 to -0.02 ) | 0.045 | 3 (2 to 6 ) | 3 (0 to 11 ) | -1.07 (-2.19 to 0.05 ) | 0.062 |
| Kwai Tsing | 3 (2 to 5 ) | 3 (0 to 12 ) | -0.21 (-1.71 to 1.31 ) | 0.783 | 3 (2 to 5 ) | 3 (0 to 11 ) | -0.28 (-1.65 to 1.10 ) | 0.688 | 3 (2 to 5 ) | 3 (0 to 12 ) | -0.24 (-1.68 to 1.21 ) | 0.740 |
| Kwun Tong | 4 (2 to 7 ) | 4 (0 to 13 ) | -0.76 (-1.81 to 0.30 ) | 0.160 | 4 (2 to 7 ) | 3 (0 to 12 ) | -0.85 (-1.90 to 0.22 ) | 0.119 | 4 (2 to 7 ) | 3 (0 to 13 ) | -0.80 (-1.85 to 0.26 ) | 0.140 |
| North | 6 (3 to 10 ) | 4 (0 to 14 ) | -2.13 (-2.70 to -1.55 ) | <.001 | 6 (3 to 9 ) | 4 (0 to 13 ) | -2.16 (-2.73 to -1.59 ) | <.001 | 6 (3 to 10 ) | 4 (0 to 13 ) | -2.15 (-2.71 to -1.57 ) | <.001 |
| Sai Kung | 4 (2 to 8 ) | 3 (0 to 12 ) | -1.63 (-2.43 to -0.83 ) | <.001 | 4 (2 to 7 ) | 3 (0 to 11 ) | -1.72 (-2.53 to -0.90 ) | <.001 | 4 (2 to 8 ) | 3 (0 to 12 ) | -1.67 (-2.48 to -0.86 ) | <.001 |
| Sha Tin | 5 (2 to 8 ) | 4 (0 to 14 ) | -0.72 (-1.43 to -0.02 ) | 0.044 | 4 (2 to 8 ) | 4 (0 to 13 ) | -0.81 (-1.53 to -0.09 ) | 0.029 | 5 (2 to 8 ) | 4 (0 to 14 ) | -0.76 (-1.47 to -0.05 ) | 0.036 |
| Sham Shui Po | 3 (2 to 6 ) | 3 (0 to 12 ) | -0.04 (-1.48 to 1.43 ) | 0.962 | 3 (2 to 5 ) | 3 (0 to 12 ) | -0.11 (-1.47 to 1.28 ) | 0.880 | 3 (2 to 5 ) | 3 (0 to 12 ) | -0.07 (-1.47 to 1.35 ) | 0.925 |
| Southern | 4 (2 to 7 ) | 4 (0 to 13 ) | -0.89 (-2.94 to 1.19 ) | 0.399 | 4 (2 to 7 ) | 3 (0 to 12 ) | -0.96 (-3.02 to 1.14 ) | 0.366 | 4 (2 to 7 ) | 4 (0 to 13 ) | -0.93 (-2.97 to 1.16 ) | 0.382 |
| Tai Po | 6 (3 to 10 ) | 4 (0 to 14 ) | -1.89 (-3.74 to -0.01 ) | 0.048 | 6 (3 to 10 ) | 4 (0 to 13 ) | -1.99 (-3.83 to -0.11 ) | 0.038 | 6 (3 to 10 ) | 4 (0 to 14 ) | -1.94 (-3.78 to -0.06 ) | 0.043 |
| Tsuen Wan | 5 (3 to 9 ) | 5 (1 to 14 ) | -0.90 (-1.86 to 0.08 ) | 0.072 | 5 (3 to 8 ) | 4 (0 to 14 ) | -0.95 (-1.89 to 0.00 ) | 0.050 | 5 (3 to 9 ) | 4 (1 to 14 ) | -0.92 (-1.87 to 0.04 ) | 0.061 |
| Tuen Mun | 4 (2 to 6 ) | 3 (0 to 10 ) | -1.61 (-2.40 to -0.82 ) | <.001 | 4 (2 to 6 ) | 3 (0 to 10 ) | -1.73 (-2.34 to -1.11 ) | <.001 | 4 (2 to 6 ) | 3 (0 to 10 ) | -1.67 (-2.30 to -1.03 ) | <.001 |
| Wan Chai | 4 (2 to 7 ) | 2 (0 to 10 ) | -2.32 (-4.50 to -0.08 ) | 0.042 | 4 (2 to 7 ) | 2 (0 to 9 ) | -2.39 (-4.48 to -0.25 ) | 0.029 | 4 (2 to 7 ) | 2 (0 to 9 ) | -2.35 (-4.49 to -0.16 ) | 0.036 |
| Wong Tai Sin | 4 (2 to 8 ) | 5 (0 to 15 ) | -0.06 (-1.20 to 1.08 ) | 0.912 | 4 (2 to 7 ) | 4 (0 to 14 ) | -0.14 (-1.13 to 0.87 ) | 0.791 | 4 (2 to 8 ) | 4 (0 to 15 ) | -0.10 (-1.17 to 0.98 ) | 0.859 |
| Yau Tsim Mong | 3 (2 to 6 ) | 3 (0 to 11 ) | -0.29 (-1.01 to 0.44 ) | 0.432 | 3 (2 to 5 ) | 3 (0 to 11 ) | -0.36 (-1.08 to 0.36 ) | 0.324 | 3 (2 to 6 ) | 3 (0 to 11 ) | -0.32 (-1.05 to 0.40 ) | 0.380 |
| Yuen Long | 4 (2 to 7 ) | 4 (0 to 12 ) | -1.11 (-1.68 to -0.54 ) | <.001 | 4 (2 to 7 ) | 3 (0 to 12 ) | -1.19 (-1.68 to -0.69 ) | <.001 | 4 (2 to 7 ) | 3 (0 to 12 ) | -1.15 (-1.68 to -0.61 ) | <.001 |
| Macao |  |  |  |  |  |  |  |  |  |  |  |  |
| Ilhas | 14 (8 to 23 ) | 11 (1 to 42 ) | -1.72 (-3.14 to -0.27 ) | 0.020 | 14 (7 to 22 ) | 11 (1 to 40 ) | -1.78 (-3.21 to -0.33 ) | 0.016 | 14 (8 to 23 ) | 11 (1 to 41 ) | -1.75 (-3.17 to -0.30 ) | 0.018 |
| Macau | 11 (6 to 18 ) | 9 (1 to 36 ) | -0.72 (-1.61 to 0.18 ) | 0.115 | 11 (6 to 17 ) | 9 (1 to 34 ) | -0.79 (-1.67 to 0.11 ) | 0.084 | 11 (6 to 18 ) | 9 (1 to 35 ) | -0.75 (-1.64 to 0.15 ) | 0.100 |
|  | **Wasting** | | | | | | | | | | | |
| Anhui |  |  |  |  |  |  |  |  |  |  |  |  |
| Anqing | 4 (2 to 7 ) | 3 (1 to 8 ) | -0.50 (-1.33 to 0.34 ) | 0.241 | 4 (2 to 7 ) | 3 (1 to 9 ) | -0.45 (-1.28 to 0.40 ) | 0.298 | 4 (2 to 7 ) | 3 (1 to 9 ) | -0.47 (-1.31 to 0.37 ) | 0.268 |
| Bengbu | 3 (2 to 5 ) | 3 (1 to 8 ) | -0.33 (-0.93 to 0.29 ) | 0.297 | 3 (2 to 5 ) | 3 (1 to 8 ) | -0.27 (-0.81 to 0.28 ) | 0.336 | 3 (2 to 5 ) | 3 (1 to 8 ) | -0.29 (-0.83 to 0.26 ) | 0.302 |
| Bozhou | 3 (2 to 6 ) | 3 (1 to 8 ) | -0.73 (-1.06 to -0.39 ) | <.001 | 3 (2 to 6 ) | 3 (1 to 9 ) | -0.67 (-1.01 to -0.33 ) | <.001 | 3 (2 to 6 ) | 3 (1 to 9 ) | -0.70 (-1.03 to -0.36 ) | <.001 |
| Chaohu | 3 (2 to 6 ) | 3 (1 to 8 ) | -0.23 (-0.62 to 0.16 ) | 0.245 | 4 (2 to 6 ) | 3 (1 to 9 ) | -0.18 (-0.58 to 0.22 ) | 0.379 | 4 (2 to 6 ) | 3 (1 to 8 ) | -0.21 (-0.60 to 0.19 ) | 0.304 |
| Chizhou | 4 (2 to 7 ) | 3 (1 to 9 ) | -0.41 (-1.21 to 0.39 ) | 0.312 | 4 (2 to 7 ) | 4 (1 to 9 ) | -0.36 (-1.18 to 0.47 ) | 0.394 | 4 (2 to 7 ) | 3 (1 to 9 ) | -0.39 (-1.20 to 0.43 ) | 0.350 |
| Chuzhou | 3 (2 to 6 ) | 3 (1 to 8 ) | -0.12 (-0.50 to 0.27 ) | 0.547 | 3 (2 to 6 ) | 3 (1 to 9 ) | -0.07 (-0.44 to 0.29 ) | 0.691 | 3 (2 to 6 ) | 3 (1 to 8 ) | -0.10 (-0.48 to 0.29 ) | 0.620 |
| Fuyang | 4 (2 to 6 ) | 3 (1 to 9 ) | -0.83 (-1.52 to -0.14 ) | 0.019 | 4 (2 to 6 ) | 3 (1 to 9 ) | -0.78 (-1.48 to -0.06 ) | 0.033 | 4 (2 to 6 ) | 3 (1 to 9 ) | -0.80 (-1.50 to -0.10 ) | 0.024 |
| Hefei | 3 (2 to 5 ) | 3 (1 to 7 ) | -0.53 (-1.03 to -0.03 ) | 0.037 | 3 (2 to 6 ) | 3 (1 to 8 ) | -0.49 (-1.01 to 0.03 ) | 0.067 | 3 (2 to 5 ) | 3 (1 to 7 ) | -0.52 (-1.06 to 0.03 ) | 0.063 |
| Huaibei | 3 (2 to 5 ) | 3 (1 to 8 ) | -0.27 (-1.20 to 0.67 ) | 0.577 | 3 (2 to 5 ) | 3 (1 to 8 ) | -0.23 (-1.16 to 0.71 ) | 0.632 | 3 (2 to 5 ) | 3 (1 to 8 ) | -0.25 (-1.18 to 0.69 ) | 0.602 |
| Huainan | 3 (2 to 5 ) | 3 (1 to 8 ) | -0.46 (-0.81 to -0.11 ) | 0.010 | 3 (2 to 6 ) | 3 (1 to 9 ) | -0.41 (-0.74 to -0.07 ) | 0.018 | 3 (2 to 6 ) | 3 (1 to 8 ) | -0.43 (-0.77 to -0.09 ) | 0.013 |
| Huangshan | 4 (2 to 7 ) | 4 (1 to 10 ) | -0.26 (-0.89 to 0.38 ) | 0.432 | 4 (2 to 7 ) | 4 (1 to 10 ) | -0.20 (-0.83 to 0.43 ) | 0.532 | 4 (2 to 7 ) | 4 (1 to 10 ) | -0.23 (-0.86 to 0.41 ) | 0.478 |
| Lu'an | 4 (2 to 6 ) | 3 (1 to 9 ) | -0.41 (-1.50 to 0.70 ) | 0.469 | 4 (2 to 6 ) | 3 (1 to 9 ) | -0.35 (-1.48 to 0.79 ) | 0.544 | 4 (2 to 6 ) | 3 (1 to 9 ) | -0.38 (-1.49 to 0.74 ) | 0.505 |
| Ma'anshan | 3 (2 to 6 ) | 3 (1 to 8 ) | -0.24 (-1.06 to 0.58 ) | 0.558 | 3 (2 to 6 ) | 3 (1 to 9 ) | -0.19 (-0.97 to 0.59 ) | 0.631 | 3 (2 to 6 ) | 3 (1 to 8 ) | -0.22 (-1.01 to 0.58 ) | 0.592 |
| Suzhou | 3 (2 to 5 ) | 3 (1 to 8 ) | -0.28 (-1.02 to 0.46 ) | 0.459 | 3 (2 to 5 ) | 3 (1 to 8 ) | -0.24 (-0.98 to 0.50 ) | 0.521 | 3 (2 to 5 ) | 3 (1 to 8 ) | -0.26 (-1.0 to 0.48 ) | 0.487 |
| Tongling | 4 (2 to 6 ) | 3 (1 to 8 ) | -0.34 (-0.99 to 0.31 ) | 0.301 | 4 (2 to 6 ) | 3 (1 to 9 ) | -0.29 (-0.95 to 0.37 ) | 0.388 | 4 (2 to 6 ) | 3 (1 to 8 ) | -0.32 (-0.97 to 0.34 ) | 0.341 |
| Wuhu | 3 (2 to 6 ) | 3 (1 to 8 ) | -0.30 (-0.83 to 0.23 ) | 0.271 | 3 (2 to 6 ) | 3 (1 to 8 ) | -0.25 (-0.75 to 0.26 ) | 0.340 | 3 (2 to 6 ) | 3 (1 to 8 ) | -0.27 (-0.79 to 0.25 ) | 0.302 |
| Xuancheng | 4 (2 to 6 ) | 3 (1 to 9 ) | -0.15 (-0.75 to 0.45 ) | 0.622 | 4 (2 to 7 ) | 4 (1 to 10 ) | -0.10 (-0.67 to 0.48 ) | 0.739 | 4 (2 to 7 ) | 3 (1 to 9 ) | -0.13 (-0.71 to 0.47 ) | 0.676 |
| Beijing |  |  |  |  |  |  |  |  |  |  |  |  |
| Beijing | 3 (2 to 5 ) | 3 (1 to 8 ) | -0.31 (-0.34 to -0.27 ) | <.001 | 3 (2 to 5 ) | 3 (1 to 8 ) | -0.27 (-0.28 to -0.25 ) | <.001 | 3 (2 to 5 ) | 3 (1 to 8 ) | -0.29 (-0.31 to -0.27 ) | <.001 |
| Chongqing |  |  |  |  |  |  |  |  |  |  |  |  |
| Chongqing | 3 (2 to 5 ) | 3 (1 to 8 ) | -0.60 (-0.63 to -0.58 ) | <.001 | 4 (2 to 6 ) | 3 (1 to 9 ) | -0.56 (-0.60 to -0.53 ) | <.001 | 3 (2 to 5 ) | 3 (1 to 8 ) | -0.60 (-0.65 to -0.54 ) | <.001 |
| Fujian |  |  |  |  |  |  |  |  |  |  |  |  |
| Fuzhou | 3 (1 to 6 ) | 3 (1 to 8 ) | -0.61 (-2.00 to 0.81 ) | 0.398 | 3 (1 to 6 ) | 3 (1 to 8 ) | -0.54 (-1.94 to 0.89 ) | 0.456 | 3 (1 to 6 ) | 3 (1 to 8 ) | -0.57 (-1.97 to 0.84 ) | 0.425 |
| Longyan | 4 (2 to 7 ) | 3 (1 to 9 ) | -0.47 (-1.40 to 0.46 ) | 0.319 | 4 (2 to 8 ) | 4 (1 to 10 ) | -0.41 (-1.38 to 0.57 ) | 0.415 | 4 (2 to 8 ) | 4 (1 to 10 ) | -0.44 (-1.39 to 0.51 ) | 0.364 |
| Nanping | 3 (1 to 7 ) | 3 (1 to 10 ) | -0.29 (-1.02 to 0.45 ) | 0.446 | 4 (1 to 7 ) | 4 (1 to 10 ) | -0.23 (-0.92 to 0.46 ) | 0.512 | 4 (1 to 7 ) | 4 (1 to 10 ) | -0.26 (-0.98 to 0.46 ) | 0.476 |
| Ningde | 3 (1 to 6 ) | 3 (1 to 9 ) | -0.29 (-0.64 to 0.07 ) | 0.115 | 3 (1 to 6 ) | 3 (1 to 10 ) | -0.24 (-0.58 to 0.11 ) | 0.183 | 3 (1 to 6 ) | 3 (1 to 10 ) | -0.26 (-0.61 to 0.09 ) | 0.144 |
| Putian | 3 (1 to 7 ) | 3 (1 to 8 ) | -0.55 (-1.08 to -0.03 ) | 0.040 | 3 (1 to 7 ) | 3 (1 to 9 ) | -0.50 (-0.99 to 0.00 ) | 0.049 | 3 (1 to 7 ) | 3 (1 to 8 ) | -0.53 (-1.04 to -0.01 ) | 0.044 |
| Quanzhou | 3 (1 to 7 ) | 3 (1 to 8 ) | -0.26 (-0.82 to 0.30 ) | 0.359 | 3 (1 to 7 ) | 3 (1 to 9 ) | -0.21 (-0.78 to 0.35 ) | 0.460 | 3 (1 to 7 ) | 3 (1 to 9 ) | -0.24 (-0.80 to 0.33 ) | 0.406 |
| Sanming | 4 (1 to 7 ) | 4 (1 to 10 ) | -0.33 (-0.67 to 0.01 ) | 0.060 | 4 (1 to 7 ) | 4 (1 to 10 ) | -0.27 (-0.59 to 0.05 ) | 0.099 | 4 (1 to 7 ) | 4 (1 to 10 ) | -0.30 (-0.63 to 0.03 ) | 0.075 |
| Xiamen | 3 (1 to 6 ) | 2 (0 to 6 ) | -0.87 (-1.77 to 0.04 ) | 0.062 | 3 (1 to 6 ) | 2 (0 to 7 ) | -0.81 (-1.75 to 0.14 ) | 0.095 | 3 (1 to 6 ) | 2 (0 to 7 ) | -0.84 (-1.76 to 0.09 ) | 0.076 |
| Zhangzhou | 3 (1 to 7 ) | 3 (1 to 8 ) | -0.43 (-0.80 to -0.07 ) | 0.020 | 4 (1 to 8 ) | 3 (1 to 9 ) | -0.38 (-0.75 to -0.01 ) | 0.041 | 4 (1 to 7 ) | 3 (1 to 9 ) | -0.41 (-0.78 to -0.04 ) | 0.029 |
| Gansu |  |  |  |  |  |  |  |  |  |  |  |  |
| Baiyin | 4 (2 to 7 ) | 3 (1 to 8 ) | -0.94 (-1.43 to -0.46 ) | <.001 | 4 (2 to 7 ) | 3 (1 to 9 ) | -0.95 (-1.34 to -0.55 ) | <.001 | 4 (2 to 7 ) | 3 (1 to 9 ) | -0.98 (-1.39 to -0.57 ) | <.001 |
| Dingxi | 3 (2 to 6 ) | 3 (1 to 9 ) | -0.22 (-0.68 to 0.25 ) | 0.353 | 3 (2 to 6 ) | 3 (1 to 9 ) | -0.17 (-0.67 to 0.33 ) | 0.499 | 3 (2 to 6 ) | 3 (1 to 9 ) | -0.19 (-0.67 to 0.28 ) | 0.422 |
| Gannan Tibetan | 4 (2 to 6 ) | 4 (1 to 11 ) | 0.23 (-0.41 to 0.86 ) | 0.482 | 4 (2 to 7 ) | 4 (1 to 11 ) | 0.29 (-0.36 to 0.94 ) | 0.384 | 4 (2 to 7 ) | 4 (1 to 11 ) | 0.26 (-0.38 to 0.90 ) | 0.432 |
| Jiayuguan | 4 (2 to 8 ) | 3 (1 to 9 ) | -1.47 (-2.86 to -0.06 ) | 0.041 | 4 (2 to 8 ) | 3 (1 to 9 ) | -1.42 (-2.83 to 0.01 ) | 0.051 | 4 (2 to 8 ) | 3 (1 to 9 ) | -1.45 (-2.85 to -0.03 ) | 0.045 |
| Jinchang | 4 (2 to 8 ) | 3 (1 to 8 ) | -1.54 (-2.80 to -0.28 ) | 0.017 | 5 (2 to 8 ) | 3 (1 to 8 ) | -1.50 (-2.75 to -0.24 ) | 0.020 | 4 (2 to 8 ) | 3 (1 to 8 ) | -1.52 (-2.78 to -0.25 ) | 0.019 |
| Jiuquan | 4 (2 to 8 ) | 3 (1 to 9 ) | -1.21 (-2.73 to 0.32 ) | 0.122 | 4 (2 to 8 ) | 4 (1 to 9 ) | -1.16 (-2.69 to 0.38 ) | 0.139 | 4 (2 to 8 ) | 4 (1 to 9 ) | -1.19 (-2.71 to 0.35 ) | 0.130 |
| Lanzhou | 4 (2 to 6 ) | 3 (1 to 7 ) | -1.18 (-1.63 to -0.72 ) | <.001 | 4 (2 to 7 ) | 3 (1 to 8 ) | -1.12 (-1.56 to -0.68 ) | <.001 | 4 (2 to 7 ) | 3 (1 to 7 ) | -1.15 (-1.60 to -0.70 ) | <.001 |
| Linxia Hui | 4 (2 to 6 ) | 3 (1 to 9 ) | -0.48 (-1.00 to 0.04 ) | 0.073 | 4 (2 to 7 ) | 3 (1 to 9 ) | -0.42 (-0.93 to 0.09 ) | 0.106 | 4 (2 to 7 ) | 3 (1 to 9 ) | -0.45 (-0.96 to 0.07 ) | 0.087 |
| Longnan | 3 (2 to 6 ) | 3 (1 to 9 ) | 0.18 (-0.26 to 0.61 ) | 0.429 | 3 (2 to 6 ) | 4 (1 to 10 ) | 0.22 (-0.22 to 0.66 ) | 0.324 | 3 (2 to 6 ) | 4 (1 to 10 ) | 0.20 (-0.24 to 0.63 ) | 0.376 |
| Pingliang | 3 (2 to 6 ) | 3 (1 to 9 ) | -0.25 (-1.15 to 0.66 ) | 0.586 | 3 (2 to 6 ) | 3 (1 to 9 ) | -0.21 (-1.11 to 0.71 ) | 0.657 | 3 (2 to 6 ) | 3 (1 to 9 ) | -0.23 (-1.13 to 0.68 ) | 0.620 |
| Qingyang | 3 (2 to 6 ) | 3 (1 to 9 ) | -0.18 (-1.71 to 1.37 ) | 0.815 | 3 (2 to 6 ) | 3 (1 to 9 ) | -0.14 (-1.67 to 1.42 ) | 0.860 | 3 (2 to 6 ) | 3 (1 to 9 ) | -0.16 (-1.69 to 1.39 ) | 0.837 |
| Tianshui | 3 (2 to 6 ) | 3 (1 to 9 ) | -0.23 (-1.05 to 0.59 ) | 0.580 | 3 (2 to 6 ) | 3 (1 to 9 ) | -0.19 (-1.00 to 0.64 ) | 0.658 | 3 (2 to 6 ) | 3 (1 to 9 ) | -0.21 (-1.03 to 0.61 ) | 0.617 |
| Wuwei | 4 (2 to 8 ) | 3 (1 to 8 ) | -1.41 (-2.35 to -0.47 ) | 0.003 | 4 (2 to 8 ) | 3 (1 to 8 ) | -1.37 (-2.30 to -0.43 ) | 0.004 | 4 (2 to 8 ) | 3 (1 to 8 ) | -1.39 (-2.33 to -0.45 ) | 0.004 |
| Zhangye | 4 (2 to 8 ) | 3 (1 to 9 ) | -1.41 (-2.33 to -0.49 ) | 0.003 | 5 (2 to 9 ) | 4 (1 to 10 ) | -1.37 (-2.28 to -0.44 ) | 0.004 | 5 (2 to 9 ) | 3 (1 to 9 ) | -1.39 (-2.31 to -0.47 ) | 0.003 |
| Guangdong |  |  |  |  |  |  |  |  |  |  |  |  |
| Chaozhou | 3 (1 to 6 ) | 3 (1 to 8 ) | -0.87 (-5.82 to 4.35 ) | 0.739 | 3 (1 to 7 ) | 3 (1 to 9 ) | -0.80 (-5.73 to 4.39 ) | 0.758 | 3 (1 to 6 ) | 3 (1 to 8 ) | -0.83 (-5.78 to 4.37 ) | 0.748 |
| Dongguan | 3 (1 to 5 ) | 3 (1 to 8 ) | -0.27 (-0.54 to 0.00 ) | 0.047 | 3 (2 to 5 ) | 3 (1 to 9 ) | -0.21 (-0.49 to 0.06 ) | 0.126 | 3 (1 to 5 ) | 3 (1 to 8 ) | -0.24 (-0.51 to 0.03 ) | 0.076 |
| Foshan | 3 (2 to 5 ) | 3 (1 to 8 ) | -0.62 (-1.08 to -0.17 ) | 0.007 | 3 (2 to 5 ) | 3 (1 to 8 ) | -0.57 (-1.02 to -0.12 ) | 0.013 | 3 (2 to 5 ) | 3 (1 to 8 ) | -0.60 (-1.05 to -0.15 ) | 0.010 |
| Guangzhou | 3 (1 to 5 ) | 3 (1 to 8 ) | -0.62 (-0.82 to -0.42 ) | <.001 | 3 (2 to 5 ) | 3 (1 to 8 ) | -0.57 (-0.78 to -0.37 ) | <.001 | 3 (2 to 5 ) | 3 (1 to 8 ) | -0.60 (-0.80 to -0.40 ) | <.001 |
| Heyuan | 3 (2 to 6 ) | 3 (1 to 9 ) | -0.50 (-3.37 to 2.46 ) | 0.737 | 3 (2 to 6 ) | 3 (1 to 9 ) | -0.43 (-3.28 to 2.50 ) | 0.769 | 3 (2 to 6 ) | 3 (1 to 9 ) | -0.47 (-3.33 to 2.47 ) | 0.752 |
| Huizhou | 3 (2 to 6 ) | 3 (1 to 8 ) | -0.50 (-2.08 to 1.10 ) | 0.538 | 3 (2 to 6 ) | 3 (1 to 9 ) | -0.43 (-2.00 to 1.16 ) | 0.591 | 3 (2 to 6 ) | 3 (1 to 9 ) | -0.47 (-2.04 to 1.13 ) | 0.563 |
| Jiangmen | 3 (2 to 5 ) | 3 (1 to 9 ) | -0.07 (-1.27 to 1.14 ) | 0.908 | 3 (2 to 5 ) | 3 (1 to 10 ) | -0.01 (-1.24 to 1.24 ) | 0.993 | 3 (2 to 5 ) | 3 (1 to 9 ) | -0.04 (-1.25 to 1.19 ) | 0.950 |
| Jieyang | 3 (1 to 6 ) | 3 (1 to 8 ) | -0.82 (-5.28 to 3.85 ) | 0.726 | 3 (1 to 7 ) | 3 (1 to 9 ) | -0.75 (-5.19 to 3.90 ) | 0.747 | 3 (1 to 6 ) | 3 (1 to 8 ) | -0.79 (-5.23 to 3.87 ) | 0.736 |
| Maoming | 3 (2 to 5 ) | 3 (1 to 10 ) | -0.24 (-3.42 to 3.05 ) | 0.885 | 4 (2 to 5 ) | 3 (1 to 11 ) | -0.17 (-3.37 to 3.13 ) | 0.917 | 4 (2 to 5 ) | 3 (1 to 11 ) | -0.21 (-3.40 to 3.09 ) | 0.900 |
| Meizhou | 3 (1 to 6 ) | 3 (1 to 9 ) | -0.70 (-5.20 to 4.01 ) | 0.767 | 3 (2 to 6 ) | 3 (1 to 9 ) | -0.63 (-5.10 to 4.05 ) | 0.787 | 3 (1 to 6 ) | 3 (1 to 9 ) | -0.67 (-5.15 to 4.03 ) | 0.777 |
| Qingyuan | 4 (2 to 6 ) | 3 (1 to 9 ) | -0.45 (-1.02 to 0.12 ) | 0.123 | 4 (2 to 6 ) | 3 (1 to 10 ) | -0.40 (-0.92 to 0.13 ) | 0.139 | 4 (2 to 6 ) | 3 (1 to 9 ) | -0.43 (-0.97 to 0.13 ) | 0.130 |
| Shantou | 3 (1 to 6 ) | 3 (1 to 9 ) | -1.00 (-5.88 to 4.12 ) | 0.696 | 3 (1 to 7 ) | 3 (1 to 9 ) | -0.93 (-5.79 to 4.17 ) | 0.714 | 3 (1 to 6 ) | 3 (1 to 9 ) | -0.97 (-5.83 to 4.14 ) | 0.704 |
| Shanwei | 3 (1 to 6 ) | 3 (1 to 8 ) | -0.59 (-4.14 to 3.09 ) | 0.748 | 3 (1 to 6 ) | 3 (1 to 9 ) | -0.53 (-4.07 to 3.15 ) | 0.776 | 3 (1 to 6 ) | 3 (1 to 9 ) | -0.56 (-4.11 to 3.12 ) | 0.761 |
| Shaoguan | 3 (2 to 6 ) | 3 (1 to 9 ) | -0.06 (-0.31 to 0.18 ) | 0.621 | 4 (2 to 6 ) | 4 (1 to 10 ) | -0.01 (-0.25 to 0.23 ) | 0.947 | 3 (2 to 6 ) | 3 (1 to 9 ) | -0.04 (-0.28 to 0.21 ) | 0.770 |
| Shenzhen | 3 (1 to 5 ) | 2 (0 to 7 ) | -0.52 (-1.22 to 0.18 ) | 0.148 | 3 (1 to 5 ) | 3 (1 to 8 ) | -0.46 (-1.14 to 0.21 ) | 0.177 | 3 (1 to 5 ) | 3 (1 to 7 ) | -0.49 (-1.18 to 0.20 ) | 0.161 |
| Yangjiang | 4 (2 to 5 ) | 3 (1 to 10 ) | 0.00 (-2.41 to 2.46 ) | 0.998 | 4 (2 to 6 ) | 4 (1 to 11 ) | 0.06 (-2.38 to 2.56 ) | 0.960 | 4 (2 to 6 ) | 3 (1 to 11 ) | 0.03 (-2.39 to 2.51 ) | 0.982 |
| Yunfu | 3 (2 to 5 ) | 3 (1 to 9 ) | -0.21 (-2.75 to 2.39 ) | 0.872 | 4 (2 to 5 ) | 3 (1 to 10 ) | -0.15 (-2.71 to 2.49 ) | 0.913 | 4 (2 to 5 ) | 3 (1 to 10 ) | -0.18 (-2.73 to 2.44 ) | 0.892 |
| Zhanjiang | 3 (2 to 5 ) | 3 (1 to 10 ) | -0.19 (-3.80 to 3.56 ) | 0.921 | 4 (2 to 5 ) | 3 (1 to 11 ) | -0.12 (-3.74 to 3.64 ) | 0.949 | 4 (2 to 5 ) | 3 (1 to 10 ) | -0.15 (-3.77 to 3.60 ) | 0.935 |
| Zhaoqing | 4 (2 to 6 ) | 3 (1 to 9 ) | -0.46 (-1.34 to 0.43 ) | 0.313 | 4 (2 to 6 ) | 3 (1 to 10 ) | -0.41 (-1.32 to 0.52 ) | 0.387 | 4 (2 to 6 ) | 3 (1 to 9 ) | -0.43 (-1.33 to 0.47 ) | 0.347 |
| Zhongshan | 3 (2 to 5 ) | 3 (1 to 9 ) | -0.34 (-0.79 to 0.10 ) | 0.126 | 3 (2 to 5 ) | 3 (1 to 9 ) | -0.39 (-0.64 to -0.13 ) | 0.003 | 3 (2 to 5 ) | 3 (1 to 9 ) | -0.31 (-0.77 to 0.14 ) | 0.180 |
| Zhuhai | 3 (2 to 5 ) | 3 (1 to 9 ) | -0.11 (-0.77 to 0.56 ) | 0.755 | 3 (2 to 6 ) | 3 (1 to 9 ) | -0.04 (-0.74 to 0.67 ) | 0.914 | 3 (2 to 5 ) | 3 (1 to 9 ) | -0.07 (-0.75 to 0.61 ) | 0.833 |
| Guangxi |  |  |  |  |  |  |  |  |  |  |  |  |
| Baise | 6 (4 to 9 ) | 5 (1 to 14 ) | -0.76 (-4.14 to 2.74 ) | 0.666 | 6 (4 to 10 ) | 6 (1 to 15 ) | -0.70 (-4.04 to 2.76 ) | 0.689 | 6 (4 to 9 ) | 6 (1 to 15 ) | -0.73 (-4.09 to 2.75 ) | 0.677 |
| Beihai | 5 (4 to 8 ) | 5 (1 to 15 ) | -0.32 (-1.35 to 0.72 ) | 0.540 | 6 (4 to 8 ) | 5 (1 to 16 ) | -0.27 (-1.31 to 0.77 ) | 0.606 | 5 (4 to 8 ) | 5 (1 to 15 ) | -0.30 (-1.33 to 0.74 ) | 0.572 |
| Chongzuo | 6 (4 to 9 ) | 5 (1 to 15 ) | -0.79 (-2.87 to 1.33 ) | 0.463 | 6 (4 to 9 ) | 6 (1 to 15 ) | -0.72 (-2.77 to 1.38 ) | 0.500 | 6 (4 to 9 ) | 5 (1 to 15 ) | -0.75 (-2.82 to 1.35 ) | 0.480 |
| Fangchenggang | 6 (4 to 9 ) | 5 (1 to 16 ) | -0.39 (-1.35 to 0.57 ) | 0.420 | 6 (4 to 9 ) | 6 (1 to 17 ) | -0.34 (-1.28 to 0.62 ) | 0.491 | 6 (4 to 9 ) | 6 (1 to 17 ) | -0.37 (-1.32 to 0.59 ) | 0.453 |
| Guigang | 6 (4 to 8 ) | 5 (1 to 15 ) | -0.74 (-1.34 to -0.14 ) | 0.016 | 6 (4 to 9 ) | 5 (1 to 15 ) | -0.70 (-1.29 to -0.09 ) | 0.023 | 6 (4 to 8 ) | 5 (1 to 15 ) | -0.72 (-1.32 to -0.12 ) | 0.019 |
| Guilin | 6 (4 to 9 ) | 5 (1 to 14 ) | -0.60 (-0.91 to -0.29 ) | <.001 | 6 (4 to 9 ) | 5 (1 to 14 ) | -0.54 (-0.86 to -0.21 ) | 0.001 | 6 (4 to 9 ) | 5 (1 to 14 ) | -0.57 (-0.88 to -0.26 ) | <.001 |
| Hechi | 6 (4 to 9 ) | 5 (1 to 15 ) | -0.54 (-1.73 to 0.68 ) | 0.386 | 6 (4 to 9 ) | 6 (1 to 16 ) | -0.49 (-1.74 to 0.77 ) | 0.441 | 6 (4 to 9 ) | 5 (1 to 15 ) | -0.52 (-1.73 to 0.72 ) | 0.412 |
| Hezhou | 6 (4 to 9 ) | 5 (1 to 14 ) | -0.66 (-3.34 to 2.09 ) | 0.634 | 6 (4 to 9 ) | 5 (1 to 15 ) | -0.60 (-3.28 to 2.16 ) | 0.668 | 6 (4 to 9 ) | 5 (1 to 14 ) | -0.63 (-3.31 to 2.12 ) | 0.650 |
| Laibin | 6 (4 to 8 ) | 5 (1 to 15 ) | -0.61 (-1.12 to -0.10 ) | 0.019 | 6 (4 to 9 ) | 5 (1 to 16 ) | -0.56 (-1.02 to -0.09 ) | 0.019 | 6 (4 to 9 ) | 5 (1 to 15 ) | -0.58 (-1.07 to -0.10 ) | 0.019 |
| Liuzhou | 6 (4 to 9 ) | 5 (1 to 15 ) | -0.77 (-1.25 to -0.29 ) | 0.002 | 6 (4 to 9 ) | 5 (1 to 15 ) | -0.71 (-1.15 to -0.27 ) | 0.002 | 6 (4 to 9 ) | 5 (1 to 15 ) | -0.74 (-1.20 to -0.28 ) | 0.002 |
| Nanning | 6 (4 to 8 ) | 5 (1 to 14 ) | -0.78 (-1.41 to -0.15 ) | 0.015 | 6 (4 to 9 ) | 5 (1 to 14 ) | -0.79 (-1.29 to -0.28 ) | 0.002 | 6 (4 to 8 ) | 5 (1 to 14 ) | -0.75 (-1.37 to -0.13 ) | 0.019 |
| Qinzhou | 5 (4 to 8 ) | 5 (1 to 15 ) | -0.47 (-1.13 to 0.19 ) | 0.160 | 6 (4 to 8 ) | 5 (1 to 15 ) | -0.44 (-1.23 to 0.37 ) | 0.286 | 6 (4 to 8 ) | 5 (1 to 15 ) | -0.44 (-1.11 to 0.22 ) | 0.191 |
| Wuzhou | 6 (4 to 9 ) | 5 (1 to 14 ) | -0.74 (-3.47 to 2.07 ) | 0.602 | 6 (4 to 9 ) | 5 (1 to 15 ) | -0.67 (-3.41 to 2.14 ) | 0.635 | 6 (4 to 9 ) | 5 (1 to 14 ) | -0.71 (-3.44 to 2.10 ) | 0.618 |
| Yulin | 5 (4 to 8 ) | 5 (1 to 15 ) | -0.55 (-1.26 to 0.17 ) | 0.135 | 6 (4 to 8 ) | 5 (1 to 15 ) | -0.50 (-1.21 to 0.22 ) | 0.174 | 6 (4 to 8 ) | 5 (1 to 15 ) | -0.52 (-1.23 to 0.19 ) | 0.153 |
| Guizhou |  |  |  |  |  |  |  |  |  |  |  |  |
| Anshun | 5 (3 to 7 ) | 4 (1 to 11 ) | -0.79 (-1.25 to -0.33 ) | 0.001 | 5 (3 to 7 ) | 4 (1 to 11 ) | -0.76 (-1.21 to -0.31 ) | 0.001 | 5 (3 to 7 ) | 4 (1 to 11 ) | -0.77 (-1.22 to -0.32 ) | 0.001 |
| Bijie | 4 (2 to 7 ) | 4 (1 to 11 ) | -0.55 (-1.00 to -0.11 ) | 0.016 | 4 (3 to 7 ) | 4 (1 to 11 ) | -0.51 (-0.96 to -0.06 ) | 0.026 | 4 (3 to 7 ) | 4 (1 to 11 ) | -0.53 (-0.98 to -0.08 ) | 0.020 |
| Guiyang | 4 (3 to 7 ) | 4 (1 to 11 ) | -0.89 (-1.14 to -0.63 ) | <.001 | 5 (3 to 7 ) | 4 (1 to 11 ) | -0.84 (-1.05 to -0.62 ) | <.001 | 5 (3 to 7 ) | 4 (1 to 11 ) | -0.86 (-1.10 to -0.63 ) | <.001 |
| Liupanshui | 5 (3 to 7 ) | 4 (1 to 11 ) | -0.66 (-1.94 to 0.64 ) | 0.319 | 5 (3 to 8 ) | 4 (1 to 12 ) | -0.61 (-1.89 to 0.68 ) | 0.350 | 5 (3 to 7 ) | 4 (1 to 11 ) | -0.64 (-1.92 to 0.66 ) | 0.334 |
| Qiandongnan Miao and Dong | 5 (3 to 7 ) | 4 (1 to 12 ) | -0.66 (-1.49 to 0.19 ) | 0.128 | 5 (3 to 7 ) | 4 (1 to 12 ) | -0.61 (-1.45 to 0.23 ) | 0.156 | 5 (3 to 7 ) | 4 (1 to 12 ) | -0.63 (-1.47 to 0.21 ) | 0.141 |
| Qiannan Buyei and Miao | 5 (3 to 7 ) | 4 (1 to 12 ) | -0.67 (-1.25 to -0.10 ) | 0.022 | 5 (3 to 7 ) | 4 (1 to 12 ) | -0.63 (-1.20 to -0.06 ) | 0.030 | 5 (3 to 7 ) | 4 (1 to 12 ) | -0.65 (-1.22 to -0.08 ) | 0.026 |
| Qianxinan Buyei and Miao | 5 (3 to 7 ) | 4 (1 to 11 ) | -0.81 (-1.53 to -0.09 ) | 0.027 | 5 (3 to 8 ) | 4 (1 to 12 ) | -0.75 (-1.41 to -0.08 ) | 0.028 | 5 (3 to 7 ) | 4 (1 to 11 ) | -0.78 (-1.47 to -0.09 ) | 0.027 |
| Tongren | 4 (3 to 7 ) | 4 (1 to 11 ) | -0.54 (-1.69 to 0.63 ) | 0.364 | 4 (3 to 7 ) | 4 (1 to 11 ) | -0.49 (-1.65 to 0.68 ) | 0.408 | 4 (3 to 7 ) | 4 (1 to 11 ) | -0.52 (-1.67 to 0.65 ) | 0.385 |
| Zunyi | 4 (3 to 7 ) | 4 (1 to 12 ) | -0.67 (-1.34 to -0.01 ) | 0.047 | 5 (3 to 7 ) | 4 (1 to 13 ) | -0.62 (-1.26 to 0.03 ) | 0.061 | 5 (3 to 7 ) | 4 (1 to 12 ) | -0.65 (-1.30 to 0.01 ) | 0.053 |
| Hainan |  |  |  |  |  |  |  |  |  |  |  |  |
| Haikou | 3 (2 to 5 ) | 3 (0 to 9 ) | -0.79 (-1.80 to 0.24 ) | 0.134 | 3 (2 to 5 ) | 3 (0 to 9 ) | -0.72 (-1.72 to 0.28 ) | 0.159 | 3 (2 to 5 ) | 3 (0 to 9 ) | -0.75 (-1.76 to 0.26 ) | 0.145 |
| Hainan | 3 (2 to 5 ) | 3 (0 to 10 ) | -0.36 (-0.60 to -0.13 ) | 0.003 | 4 (2 to 6 ) | 3 (1 to 10 ) | -0.33 (-0.42 to -0.24 ) | <.001 | 3 (2 to 5 ) | 3 (1 to 10 ) | -0.35 (-0.45 to -0.26 ) | <.001 |
| Sanya | 3 (2 to 6 ) | 3 (1 to 10 ) | -0.26 (-0.67 to 0.15 ) | 0.212 | 4 (2 to 6 ) | 3 (1 to 11 ) | -0.22 (-0.63 to 0.19 ) | 0.298 | 4 (2 to 6 ) | 3 (1 to 11 ) | -0.24 (-0.65 to 0.17 ) | 0.251 |
| Hebei |  |  |  |  |  |  |  |  |  |  |  |  |
| Baoding | 3 (2 to 5 ) | 3 (1 to 8 ) | -0.64 (-1.04 to -0.24 ) | 0.002 | 4 (2 to 5 ) | 3 (1 to 9 ) | -0.59 (-0.95 to -0.23 ) | 0.001 | 3 (2 to 5 ) | 3 (1 to 8 ) | -0.62 (-1.01 to -0.22 ) | 0.002 |
| Cangzhou | 3 (2 to 5 ) | 3 (1 to 8 ) | -0.04 (-0.38 to 0.30 ) | 0.801 | 3 (2 to 5 ) | 3 (1 to 8 ) | -0.04 (-0.71 to 0.64 ) | 0.909 | 3 (2 to 5 ) | 3 (1 to 8 ) | -0.02 (-0.37 to 0.32 ) | 0.895 |
| Chengde | 5 (3 to 8 ) | 3 (1 to 10 ) | -2.20 (-3.86 to -0.51 ) | 0.011 | 5 (3 to 8 ) | 3 (1 to 10 ) | -2.16 (-3.80 to -0.48 ) | 0.012 | 5 (3 to 8 ) | 3 (1 to 10 ) | -2.18 (-3.83 to -0.50 ) | 0.011 |
| Handan | 3 (2 to 5 ) | 3 (1 to 8 ) | 0.01 (-0.68 to 0.70 ) | 0.986 | 3 (2 to 5 ) | 3 (1 to 9 ) | 0.05 (-0.64 to 0.74 ) | 0.887 | 3 (2 to 5 ) | 3 (1 to 9 ) | 0.03 (-0.66 to 0.72 ) | 0.939 |
| Hengshui | 3 (2 to 5 ) | 3 (1 to 8 ) | 0.25 (-0.65 to 1.15 ) | 0.591 | 3 (2 to 5 ) | 3 (1 to 9 ) | 0.32 (-0.61 to 1.25 ) | 0.501 | 3 (2 to 5 ) | 3 (1 to 9 ) | 0.28 (-0.63 to 1.20 ) | 0.545 |
| Langfang | 3 (2 to 5 ) | 3 (1 to 8 ) | -0.45 (-0.81 to -0.08 ) | 0.018 | 3 (2 to 5 ) | 3 (1 to 9 ) | -0.40 (-0.77 to -0.03 ) | 0.033 | 3 (2 to 5 ) | 3 (1 to 8 ) | -0.42 (-0.79 to -0.05 ) | 0.025 |
| Qinhuangdao | 4 (2 to 7 ) | 3 (1 to 9 ) | -1.26 (-2.53 to 0.02 ) | 0.054 | 4 (2 to 7 ) | 3 (1 to 9 ) | -1.46 (-3.00 to 0.11 ) | 0.068 | 4 (2 to 7 ) | 3 (1 to 9 ) | -1.49 (-3.06 to 0.10 ) | 0.066 |
| Shijiazhuang | 3 (2 to 5 ) | 3 (1 to 8 ) | -0.39 (-0.78 to 0.00 ) | 0.047 | 3 (2 to 5 ) | 3 (1 to 8 ) | -0.32 (-0.73 to 0.08 ) | 0.120 | 3 (2 to 5 ) | 3 (1 to 8 ) | -0.36 (-0.75 to 0.04 ) | 0.075 |
| Tangshan | 4 (2 to 6 ) | 3 (1 to 9 ) | -1.00 (-2.04 to 0.04 ) | 0.059 | 4 (2 to 6 ) | 3 (1 to 9 ) | -0.96 (-2.01 to 0.09 ) | 0.074 | 4 (2 to 6 ) | 3 (1 to 9 ) | -0.98 (-2.02 to 0.06 ) | 0.066 |
| Xingtai | 3 (2 to 5 ) | 3 (1 to 8 ) | 0.09 (-0.41 to 0.59 ) | 0.731 | 3 (2 to 5 ) | 3 (1 to 9 ) | 0.13 (-0.36 to 0.63 ) | 0.604 | 3 (2 to 5 ) | 3 (1 to 9 ) | 0.11 (-0.39 to 0.61 ) | 0.669 |
| Zhangjiakou | 5 (3 to 7 ) | 3 (1 to 9 ) | -2.13 (-4.25 to 0.04 ) | 0.055 | 5 (3 to 8 ) | 3 (1 to 9 ) | -2.09 (-4.19 to 0.06 ) | 0.057 | 5 (3 to 8 ) | 3 (1 to 9 ) | -2.11 (-4.23 to 0.06 ) | 0.056 |
| Heilongjiang |  |  |  |  |  |  |  |  |  |  |  |  |
| Daqing | 3 (2 to 6 ) | 3 (1 to 8 ) | -1.24 (-1.61 to -0.87 ) | <.001 | 3 (2 to 6 ) | 3 (1 to 9 ) | -1.18 (-1.53 to -0.83 ) | <.001 | 3 (2 to 6 ) | 3 (1 to 9 ) | -1.21 (-1.57 to -0.85 ) | <.001 |
| Daxing'anling | 4 (1 to 9 ) | 3 (1 to 10 ) | -1.58 (-3.94 to 0.85 ) | 0.200 | 4 (1 to 10 ) | 3 (1 to 11 ) | -1.51 (-3.84 to 0.87 ) | 0.211 | 4 (1 to 9 ) | 3 (1 to 10 ) | -1.55 (-3.90 to 0.86 ) | 0.205 |
| Harbin | 3 (1 to 5 ) | 3 (1 to 9 ) | -0.19 (-0.60 to 0.23 ) | 0.373 | 3 (1 to 6 ) | 3 (1 to 9 ) | -0.17 (-0.56 to 0.23 ) | 0.405 | 3 (1 to 6 ) | 3 (1 to 9 ) | -0.16 (-0.58 to 0.26 ) | 0.460 |
| Hegang | 3 (1 to 6 ) | 3 (0 to 9 ) | 0.54 (-0.03 to 1.10 ) | 0.063 | 3 (1 to 6 ) | 3 (1 to 10 ) | 0.60 (0.00 to 1.20 ) | 0.051 | 3 (1 to 6 ) | 3 (1 to 10 ) | 0.57 (-0.01 to 1.15 ) | 0.056 |
| Heihe | 3 (1 to 7 ) | 3 (1 to 9 ) | -0.79 (-1.94 to 0.37 ) | 0.182 | 3 (1 to 7 ) | 3 (1 to 10 ) | -0.72 (-1.82 to 0.38 ) | 0.198 | 3 (1 to 7 ) | 3 (1 to 10 ) | -0.76 (-1.88 to 0.38 ) | 0.189 |
| Jiamusi | 3 (1 to 6 ) | 3 (1 to 10 ) | 0.70 (0.04 to 1.36 ) | 0.036 | 3 (1 to 6 ) | 3 (1 to 10 ) | 0.70 (0.15 to 1.26 ) | 0.013 | 3 (1 to 6 ) | 3 (1 to 10 ) | 0.67 (0.13 to 1.22 ) | 0.014 |
| Jixi | 3 (1 to 6 ) | 3 (0 to 10 ) | 0.92 (-0.09 to 1.94 ) | 0.073 | 3 (1 to 6 ) | 3 (1 to 10 ) | 0.98 (-0.06 to 2.03 ) | 0.065 | 3 (1 to 6 ) | 3 (1 to 10 ) | 0.95 (-0.07 to 1.98 ) | 0.069 |
| Mudanjiang | 3 (1 to 6 ) | 3 (1 to 10 ) | 0.69 (-0.18 to 1.57 ) | 0.122 | 3 (1 to 6 ) | 3 (1 to 10 ) | 0.73 (-0.14 to 1.61 ) | 0.102 | 3 (1 to 6 ) | 3 (1 to 10 ) | 0.71 (-0.16 to 1.59 ) | 0.111 |
| Qiqihar | 3 (2 to 6 ) | 3 (0 to 8 ) | -1.50 (-2.37 to -0.62 ) | 0.001 | 4 (2 to 6 ) | 3 (1 to 9 ) | -1.44 (-2.29 to -0.59 ) | 0.001 | 4 (2 to 6 ) | 3 (1 to 9 ) | -1.47 (-2.33 to -0.60 ) | 0.001 |
| Qitaihe | 3 (1 to 6 ) | 3 (0 to 10 ) | 0.68 (-0.12 to 1.48 ) | 0.094 | 3 (1 to 6 ) | 3 (1 to 10 ) | 0.73 (-0.09 to 1.57 ) | 0.081 | 3 (1 to 6 ) | 3 (0 to 10 ) | 0.71 (-0.10 to 1.52 ) | 0.087 |
| Shuangyashan | 3 (1 to 6 ) | 3 (0 to 10 ) | 0.83 (0.06 to 1.61 ) | 0.034 | 3 (1 to 7 ) | 3 (1 to 11 ) | 0.89 (0.09 to 1.69 ) | 0.028 | 3 (1 to 6 ) | 3 (0 to 10 ) | 0.86 (0.08 to 1.65 ) | 0.031 |
| Suihua | 3 (1 to 6 ) | 3 (1 to 9 ) | -0.59 (-0.94 to -0.25 ) | 0.001 | 3 (1 to 6 ) | 3 (1 to 9 ) | -0.55 (-0.89 to -0.21 ) | 0.002 | 3 (1 to 6 ) | 3 (1 to 9 ) | -0.57 (-0.92 to -0.23 ) | 0.001 |
| Yichun | 3 (1 to 6 ) | 3 (1 to 10 ) | 0.18 (-0.21 to 0.56 ) | 0.374 | 3 (1 to 7 ) | 3 (1 to 10 ) | 0.23 (-0.17 to 0.63 ) | 0.253 | 3 (1 to 6 ) | 3 (1 to 10 ) | 0.20 (-0.19 to 0.59 ) | 0.310 |
| Henan |  |  |  |  |  |  |  |  |  |  |  |  |
| Anyang | 3 (2 to 5 ) | 2 (1 to 7 ) | -0.80 (-1.49 to -0.10 ) | 0.026 | 3 (2 to 5 ) | 3 (1 to 7 ) | -0.75 (-1.44 to -0.06 ) | 0.034 | 3 (2 to 5 ) | 2 (1 to 7 ) | -0.78 (-1.47 to -0.08 ) | 0.029 |
| Hebi | 3 (2 to 5 ) | 2 (1 to 7 ) | -0.76 (-1.28 to -0.23 ) | 0.005 | 3 (2 to 5 ) | 3 (1 to 7 ) | -0.71 (-1.23 to -0.19 ) | 0.008 | 3 (2 to 5 ) | 3 (1 to 7 ) | -0.74 (-1.26 to -0.21 ) | 0.006 |
| Jiaozuo | 3 (2 to 5 ) | 3 (1 to 7 ) | -0.69 (-1.75 to 0.38 ) | 0.204 | 3 (2 to 6 ) | 3 (1 to 8 ) | -0.65 (-1.70 to 0.42 ) | 0.231 | 3 (2 to 5 ) | 3 (1 to 7 ) | -0.67 (-1.73 to 0.40 ) | 0.217 |
| Jiyuan shi | 3 (2 to 5 ) | 3 (1 to 7 ) | -0.92 (-2.81 to 1.02 ) | 0.350 | 3 (2 to 6 ) | 3 (1 to 8 ) | -0.85 (-2.74 to 1.07 ) | 0.383 | 3 (2 to 6 ) | 3 (1 to 7 ) | -0.89 (-2.78 to 1.04 ) | 0.365 |
| Kaifeng | 3 (2 to 5 ) | 3 (1 to 7 ) | -0.50 (-0.73 to -0.27 ) | <.001 | 3 (2 to 5 ) | 3 (1 to 7 ) | -0.46 (-0.68 to -0.23 ) | <.001 | 3 (2 to 5 ) | 3 (1 to 7 ) | -0.48 (-0.71 to -0.25 ) | <.001 |
| Luohe | 3 (2 to 5 ) | 3 (1 to 7 ) | -0.47 (-0.66 to -0.28 ) | <.001 | 3 (2 to 5 ) | 3 (1 to 8 ) | -0.43 (-0.78 to -0.09 ) | 0.014 | 3 (2 to 5 ) | 3 (1 to 7 ) | -0.46 (-0.82 to -0.10 ) | 0.012 |
| Luoyang | 3 (2 to 5 ) | 3 (1 to 7 ) | -0.78 (-2.39 to 0.85 ) | 0.344 | 3 (2 to 5 ) | 3 (1 to 8 ) | -0.72 (-2.35 to 0.94 ) | 0.394 | 3 (2 to 5 ) | 3 (1 to 8 ) | -0.75 (-2.37 to 0.89 ) | 0.368 |
| Nanyang | 3 (2 to 6 ) | 3 (1 to 8 ) | -0.86 (-2.03 to 0.32 ) | 0.151 | 3 (2 to 6 ) | 3 (1 to 8 ) | -0.82 (-2.00 to 0.38 ) | 0.178 | 3 (2 to 6 ) | 3 (1 to 8 ) | -0.84 (-2.02 to 0.35 ) | 0.164 |
| Pingdingshan | 3 (2 to 5 ) | 3 (1 to 7 ) | -0.59 (-1.16 to 0.00 ) | 0.048 | 3 (2 to 5 ) | 3 (1 to 8 ) | -0.54 (-1.13 to 0.05 ) | 0.073 | 3 (2 to 5 ) | 3 (1 to 7 ) | -0.56 (-1.15 to 0.02 ) | 0.060 |
| Puyang | 3 (2 to 4 ) | 2 (1 to 7 ) | -0.63 (-1.44 to 0.18 ) | 0.126 | 3 (2 to 5 ) | 3 (1 to 7 ) | -0.59 (-1.38 to 0.21 ) | 0.148 | 3 (2 to 5 ) | 2 (1 to 7 ) | -0.61 (-1.41 to 0.19 ) | 0.135 |
| Sanmenxia | 4 (2 to 6 ) | 3 (1 to 8 ) | -1.16 (-2.82 to 0.52 ) | 0.175 | 4 (2 to 6 ) | 3 (1 to 9 ) | -1.24 (-3.03 to 0.58 ) | 0.181 | 4 (2 to 6 ) | 3 (1 to 9 ) | -1.28 (-3.07 to 0.55 ) | 0.168 |
| Shangqiu | 3 (2 to 5 ) | 3 (1 to 8 ) | -0.33 (-1.56 to 0.91 ) | 0.601 | 3 (2 to 5 ) | 3 (1 to 8 ) | -0.26 (-1.42 to 0.92 ) | 0.667 | 3 (2 to 5 ) | 3 (1 to 8 ) | -0.30 (-1.49 to 0.91 ) | 0.631 |
| Xinxiang | 3 (2 to 5 ) | 3 (1 to 7 ) | -0.62 (-1.26 to 0.03 ) | 0.060 | 3 (2 to 5 ) | 3 (1 to 8 ) | -0.57 (-1.21 to 0.07 ) | 0.080 | 3 (2 to 5 ) | 3 (1 to 7 ) | -0.60 (-1.23 to 0.05 ) | 0.068 |
| Xinyang | 3 (2 to 5 ) | 3 (1 to 8 ) | -0.27 (-3.91 to 3.51 ) | 0.886 | 3 (2 to 5 ) | 3 (1 to 8 ) | -0.36 (-2.88 to 2.23 ) | 0.784 | 3 (2 to 5 ) | 3 (1 to 8 ) | -0.24 (-3.88 to 3.55 ) | 0.900 |
| Xuchang | 3 (2 to 5 ) | 3 (1 to 8 ) | -0.50 (-0.80 to -0.20 ) | 0.001 | 3 (2 to 5 ) | 3 (1 to 8 ) | -0.45 (-0.75 to -0.16 ) | 0.003 | 3 (2 to 5 ) | 3 (1 to 8 ) | -0.48 (-0.78 to -0.18 ) | 0.002 |
| Zhengzhou | 3 (1 to 5 ) | 2 (1 to 7 ) | -0.65 (-1.22 to -0.07 ) | 0.028 | 3 (2 to 5 ) | 3 (1 to 7 ) | -0.61 (-1.17 to -0.04 ) | 0.037 | 3 (2 to 5 ) | 2 (1 to 7 ) | -0.63 (-1.20 to -0.05 ) | 0.032 |
| Zhoukou | 3 (1 to 5 ) | 3 (1 to 7 ) | -0.39 (-1.04 to 0.26 ) | 0.239 | 3 (2 to 5 ) | 3 (1 to 8 ) | -0.33 (-0.98 to 0.32 ) | 0.315 | 3 (2 to 5 ) | 3 (1 to 7 ) | -0.36 (-1.01 to 0.29 ) | 0.273 |
| Zhumadian | 3 (2 to 5 ) | 3 (1 to 8 ) | -0.42 (-1.41 to 0.57 ) | 0.402 | 3 (2 to 5 ) | 3 (1 to 8 ) | -0.38 (-1.38 to 0.63 ) | 0.458 | 3 (2 to 5 ) | 3 (1 to 8 ) | -0.40 (-1.39 to 0.60 ) | 0.428 |
| Hubei |  |  |  |  |  |  |  |  |  |  |  |  |
| Enshi Tujia and Miao | 4 (2 to 6 ) | 3 (1 to 8 ) | -1.04 (-2.98 to 0.95 ) | 0.304 | 4 (2 to 6 ) | 3 (1 to 8 ) | -0.97 (-2.92 to 1.02 ) | 0.335 | 4 (2 to 6 ) | 3 (1 to 8 ) | -1.01 (-2.95 to 0.98 ) | 0.318 |
| Ezhou | 3 (1 to 5 ) | 3 (1 to 8 ) | -0.61 (-2.75 to 1.58 ) | 0.580 | 3 (1 to 5 ) | 3 (1 to 8 ) | -0.55 (-2.65 to 1.61 ) | 0.617 | 3 (1 to 5 ) | 3 (1 to 8 ) | -0.58 (-2.71 to 1.59 ) | 0.597 |
| Huanggang | 3 (1 to 5 ) | 3 (1 to 8 ) | -0.47 (-2.82 to 1.95 ) | 0.702 | 3 (1 to 5 ) | 3 (1 to 8 ) | -0.40 (-2.72 to 1.97 ) | 0.739 | 3 (1 to 5 ) | 3 (1 to 8 ) | -0.43 (-2.77 to 1.96 ) | 0.719 |
| Huangshi | 3 (1 to 5 ) | 3 (1 to 8 ) | -0.67 (-3.55 to 2.30 ) | 0.655 | 3 (1 to 5 ) | 3 (1 to 8 ) | -0.60 (-3.46 to 2.34 ) | 0.685 | 3 (1 to 5 ) | 3 (1 to 8 ) | -0.64 (-3.51 to 2.32 ) | 0.669 |
| Jingmen | 3 (2 to 5 ) | 3 (1 to 8 ) | -0.76 (-1.28 to -0.23 ) | 0.005 | 3 (2 to 6 ) | 3 (1 to 8 ) | -0.71 (-1.23 to -0.18 ) | 0.009 | 3 (2 to 6 ) | 3 (1 to 8 ) | -0.73 (-1.26 to -0.21 ) | 0.007 |
| Jingzhou | 3 (2 to 5 ) | 3 (1 to 8 ) | -0.80 (-1.23 to -0.37 ) | <.001 | 3 (2 to 6 ) | 3 (1 to 8 ) | -0.75 (-1.18 to -0.32 ) | 0.001 | 3 (2 to 6 ) | 3 (1 to 8 ) | -0.78 (-1.21 to -0.34 ) | <.001 |
| Qianjiang | 3 (2 to 6 ) | 3 (1 to 8 ) | -0.73 (-1.34 to -0.11 ) | 0.020 | 3 (2 to 6 ) | 3 (1 to 9 ) | -0.67 (-1.30 to -0.04 ) | 0.038 | 3 (2 to 6 ) | 3 (1 to 8 ) | -0.70 (-1.32 to -0.08 ) | 0.028 |
| Shennongjia | 4 (2 to 6 ) | 3 (1 to 9 ) | -0.86 (-2.31 to 0.60 ) | 0.247 | 4 (2 to 6 ) | 3 (1 to 9 ) | -0.81 (-2.27 to 0.66 ) | 0.277 | 4 (2 to 6 ) | 3 (1 to 9 ) | -0.84 (-2.29 to 0.63 ) | 0.261 |
| Shiyan | 4 (2 to 6 ) | 3 (1 to 9 ) | -1.15 (-2.19 to -0.09 ) | 0.034 | 4 (2 to 6 ) | 3 (1 to 9 ) | -1.10 (-2.15 to -0.04 ) | 0.043 | 4 (2 to 6 ) | 3 (1 to 9 ) | -1.12 (-2.17 to -0.07 ) | 0.038 |
| Suizhou Shi | 3 (2 to 5 ) | 3 (1 to 8 ) | -0.47 (-0.82 to -0.13 ) | 0.007 | 3 (2 to 5 ) | 3 (1 to 9 ) | -0.42 (-0.76 to -0.08 ) | 0.015 | 3 (2 to 5 ) | 3 (1 to 8 ) | -0.45 (-0.79 to -0.11 ) | 0.010 |
| Tianmen | 3 (2 to 5 ) | 3 (1 to 8 ) | -0.61 (-1.17 to -0.05 ) | 0.033 | 3 (2 to 6 ) | 3 (1 to 9 ) | -0.55 (-1.13 to 0.02 ) | 0.059 | 3 (2 to 5 ) | 3 (1 to 8 ) | -0.58 (-1.15 to -0.02 ) | 0.044 |
| Wuhan | 3 (1 to 4 ) | 2 (1 to 7 ) | -0.68 (-1.87 to 0.53 ) | 0.268 | 3 (1 to 5 ) | 2 (1 to 7 ) | -0.61 (-1.79 to 0.57 ) | 0.310 | 3 (1 to 4 ) | 2 (1 to 7 ) | -0.65 (-1.83 to 0.55 ) | 0.288 |
| Xiangfan | 3 (2 to 5 ) | 3 (1 to 8 ) | -0.75 (-1.33 to -0.16 ) | 0.012 | 3 (2 to 6 ) | 3 (1 to 8 ) | -0.69 (-1.31 to -0.08 ) | 0.027 | 3 (2 to 5 ) | 3 (1 to 8 ) | -0.72 (-1.32 to -0.12 ) | 0.018 |
| Xianning | 3 (2 to 5 ) | 3 (1 to 8 ) | -0.46 (-1.13 to 0.21 ) | 0.176 | 3 (2 to 5 ) | 3 (1 to 9 ) | -0.41 (-1.03 to 0.22 ) | 0.201 | 3 (2 to 5 ) | 3 (1 to 9 ) | -0.44 (-1.08 to 0.21 ) | 0.187 |
| Xiantao | 3 (2 to 5 ) | 3 (1 to 8 ) | -0.60 (-1.04 to -0.15 ) | 0.008 | 3 (2 to 5 ) | 3 (1 to 8 ) | -0.55 (-0.87 to -0.22 ) | 0.001 | 3 (2 to 5 ) | 3 (1 to 8 ) | -0.58 (-0.91 to -0.24 ) | 0.001 |
| Xiaogan | 3 (2 to 5 ) | 3 (1 to 8 ) | -0.51 (-0.91 to -0.11 ) | 0.013 | 3 (2 to 5 ) | 3 (1 to 8 ) | -0.44 (-0.82 to -0.05 ) | 0.027 | 3 (2 to 5 ) | 3 (1 to 8 ) | -0.47 (-0.86 to -0.09 ) | 0.017 |
| Yichang | 4 (2 to 6 ) | 3 (1 to 8 ) | -0.88 (-2.33 to 0.59 ) | 0.240 | 4 (2 to 6 ) | 3 (1 to 8 ) | -0.81 (-2.29 to 0.69 ) | 0.286 | 4 (2 to 6 ) | 3 (1 to 8 ) | -0.85 (-2.31 to 0.64 ) | 0.261 |
| Hunan |  |  |  |  |  |  |  |  |  |  |  |  |
| Changde | 4 (3 to 7 ) | 4 (1 to 11 ) | -0.48 (-0.99 to 0.05 ) | 0.073 | 5 (3 to 7 ) | 4 (1 to 12 ) | -0.45 (-1.25 to 0.37 ) | 0.279 | 4 (3 to 7 ) | 4 (1 to 12 ) | -0.45 (-0.97 to 0.07 ) | 0.091 |
| Changsha | 4 (2 to 6 ) | 4 (1 to 11 ) | -0.48 (-1.02 to 0.08 ) | 0.091 | 4 (2 to 7 ) | 4 (1 to 12 ) | -0.41 (-0.94 to 0.13 ) | 0.134 | 4 (2 to 7 ) | 4 (1 to 11 ) | -0.44 (-0.98 to 0.10 ) | 0.109 |
| Chenzhou | 5 (3 to 7 ) | 4 (1 to 12 ) | -0.40 (-0.96 to 0.17 ) | 0.173 | 5 (3 to 8 ) | 4 (1 to 12 ) | -0.34 (-0.87 to 0.19 ) | 0.205 | 5 (3 to 8 ) | 4 (1 to 12 ) | -0.37 (-0.92 to 0.18 ) | 0.187 |
| Hengyang | 5 (3 to 7 ) | 4 (1 to 12 ) | -0.56 (-0.93 to -0.19 ) | 0.003 | 5 (3 to 8 ) | 4 (1 to 12 ) | -0.51 (-0.83 to -0.19 ) | 0.002 | 5 (3 to 7 ) | 4 (1 to 12 ) | -0.53 (-0.88 to -0.19 ) | 0.002 |
| Huaihua | 5 (3 to 8 ) | 5 (1 to 13 ) | -0.64 (-2.55 to 1.31 ) | 0.520 | 5 (3 to 8 ) | 5 (1 to 14 ) | -0.57 (-2.48 to 1.38 ) | 0.563 | 5 (3 to 8 ) | 5 (1 to 13 ) | -0.61 (-2.52 to 1.34 ) | 0.540 |
| Loudi | 5 (3 to 7 ) | 4 (1 to 11 ) | -0.64 (-0.79 to -0.50 ) | <.001 | 5 (3 to 7 ) | 4 (1 to 12 ) | -0.60 (-0.74 to -0.45 ) | <.001 | 5 (3 to 7 ) | 4 (1 to 11 ) | -0.62 (-0.77 to -0.47 ) | <.001 |
| Shaoyang | 5 (3 to 7 ) | 4 (1 to 12 ) | -0.84 (-1.68 to 0.01 ) | 0.052 | 5 (3 to 7 ) | 4 (1 to 12 ) | -0.77 (-1.62 to 0.07 ) | 0.073 | 5 (3 to 7 ) | 4 (1 to 12 ) | -0.81 (-1.65 to 0.04 ) | 0.061 |
| Xiangtan | 4 (3 to 7 ) | 4 (1 to 12 ) | -0.46 (-0.81 to -0.11 ) | 0.011 | 5 (3 to 7 ) | 4 (1 to 12 ) | -0.39 (-0.77 to -0.02 ) | 0.040 | 5 (3 to 7 ) | 4 (1 to 12 ) | -0.43 (-0.79 to -0.07 ) | 0.021 |
| Xiangxi Tujia and Miao | 5 (3 to 8 ) | 4 (1 to 12 ) | -0.57 (-3.17 to 2.09 ) | 0.669 | 5 (3 to 8 ) | 5 (1 to 13 ) | -0.51 (-3.09 to 2.14 ) | 0.703 | 5 (3 to 8 ) | 5 (1 to 13 ) | -0.54 (-3.13 to 2.11 ) | 0.685 |
| Yiyang | 4 (3 to 7 ) | 4 (1 to 11 ) | -0.46 (-0.93 to 0.01 ) | 0.055 | 5 (3 to 7 ) | 4 (1 to 12 ) | -0.41 (-0.89 to 0.07 ) | 0.091 | 4 (3 to 7 ) | 4 (1 to 12 ) | -0.44 (-0.91 to 0.04 ) | 0.071 |
| Yongzhou | 5 (3 to 7 ) | 4 (1 to 12 ) | -0.74 (-1.54 to 0.07 ) | 0.073 | 5 (3 to 8 ) | 4 (1 to 13 ) | -0.68 (-1.51 to 0.15 ) | 0.110 | 5 (3 to 8 ) | 4 (1 to 12 ) | -0.71 (-1.53 to 0.11 ) | 0.090 |
| Yueyang | 4 (2 to 7 ) | 4 (1 to 11 ) | -0.26 (-1.30 to 0.78 ) | 0.619 | 4 (2 to 7 ) | 4 (1 to 12 ) | -0.20 (-1.18 to 0.80 ) | 0.698 | 4 (2 to 7 ) | 4 (1 to 12 ) | -0.23 (-1.24 to 0.79 ) | 0.656 |
| Zhangjiajie | 5 (3 to 7 ) | 4 (1 to 12 ) | -0.63 (-1.51 to 0.26 ) | 0.165 | 5 (3 to 8 ) | 4 (1 to 13 ) | -0.57 (-1.49 to 0.35 ) | 0.224 | 5 (3 to 8 ) | 4 (1 to 12 ) | -0.60 (-1.50 to 0.30 ) | 0.192 |
| Zhuzhou | 4 (2 to 7 ) | 4 (1 to 12 ) | -0.43 (-3.22 to 2.45 ) | 0.769 | 5 (3 to 7 ) | 4 (1 to 13 ) | -0.36 (-3.14 to 2.51 ) | 0.805 | 5 (2 to 7 ) | 4 (1 to 13 ) | -0.39 (-3.18 to 2.48 ) | 0.786 |
| Jiangsu |  |  |  |  |  |  |  |  |  |  |  |  |
| Changzhou | 3 (1 to 5 ) | 3 (1 to 7 ) | -0.49 (-0.97 to -0.01 ) | 0.047 | 3 (2 to 6 ) | 3 (1 to 8 ) | -0.44 (-0.90 to 0.03 ) | 0.068 | 3 (1 to 6 ) | 3 (1 to 8 ) | -0.46 (-0.94 to 0.01 ) | 0.056 |
| Huai'an | 3 (2 to 5 ) | 3 (1 to 7 ) | -0.53 (-0.74 to -0.31 ) | <.001 | 3 (2 to 5 ) | 3 (1 to 7 ) | -0.48 (-0.69 to -0.27 ) | <.001 | 3 (2 to 5 ) | 3 (1 to 7 ) | -0.50 (-0.72 to -0.29 ) | <.001 |
| Lianyungang | 3 (2 to 5 ) | 2 (1 to 7 ) | -0.64 (-2.39 to 1.14 ) | 0.476 | 3 (2 to 5 ) | 3 (1 to 7 ) | -0.60 (-2.35 to 1.18 ) | 0.505 | 3 (2 to 5 ) | 3 (1 to 7 ) | -0.62 (-2.37 to 1.16 ) | 0.490 |
| Nanjing | 3 (1 to 5 ) | 2 (1 to 6 ) | -0.66 (-1.33 to 0.01 ) | 0.054 | 3 (2 to 5 ) | 3 (1 to 7 ) | -0.61 (-1.27 to 0.05 ) | 0.072 | 3 (2 to 5 ) | 2 (1 to 6 ) | -0.64 (-1.30 to 0.03 ) | 0.062 |
| Nantong | 3 (2 to 6 ) | 3 (1 to 7 ) | -0.70 (-1.61 to 0.21 ) | 0.132 | 3 (2 to 6 ) | 3 (1 to 8 ) | -0.64 (-1.56 to 0.29 ) | 0.175 | 3 (2 to 6 ) | 3 (1 to 7 ) | -0.67 (-1.58 to 0.25 ) | 0.151 |
| Suqian | 3 (2 to 5 ) | 3 (1 to 7 ) | -0.55 (-1.72 to 0.64 ) | 0.362 | 3 (2 to 5 ) | 3 (1 to 7 ) | -0.56 (-0.96 to -0.16 ) | 0.006 | 3 (2 to 5 ) | 3 (1 to 7 ) | -0.52 (-1.68 to 0.65 ) | 0.383 |
| Suzhou | 3 (1 to 5 ) | 2 (1 to 7 ) | -0.62 (-1.35 to 0.13 ) | 0.104 | 3 (1 to 6 ) | 3 (1 to 7 ) | -0.56 (-1.34 to 0.22 ) | 0.158 | 3 (1 to 5 ) | 3 (1 to 7 ) | -0.59 (-1.35 to 0.17 ) | 0.128 |
| Taizhou | 3 (2 to 5 ) | 3 (1 to 8 ) | -0.59 (-1.16 to -0.01 ) | 0.046 | 3 (2 to 6 ) | 3 (1 to 8 ) | -0.53 (-1.10 to 0.03 ) | 0.064 | 3 (2 to 6 ) | 3 (1 to 8 ) | -0.56 (-1.13 to 0.01 ) | 0.053 |
| Wuxi | 3 (1 to 5 ) | 3 (1 to 7 ) | -0.59 (-1.09 to -0.09 ) | 0.020 | 3 (1 to 6 ) | 3 (1 to 8 ) | -0.54 (-1.05 to -0.03 ) | 0.038 | 3 (1 to 5 ) | 3 (1 to 7 ) | -0.57 (-1.07 to -0.06 ) | 0.027 |
| Xuzhou | 3 (2 to 5 ) | 2 (1 to 6 ) | -0.63 (-2.50 to 1.28 ) | 0.517 | 3 (2 to 5 ) | 3 (1 to 7 ) | -0.59 (-2.47 to 1.33 ) | 0.547 | 3 (2 to 5 ) | 2 (1 to 6 ) | -0.61 (-2.48 to 1.31 ) | 0.531 |
| Yancheng | 3 (2 to 5 ) | 3 (1 to 7 ) | -0.66 (-1.26 to -0.06 ) | 0.030 | 3 (2 to 5 ) | 3 (1 to 8 ) | -0.62 (-1.25 to 0.02 ) | 0.060 | 3 (2 to 5 ) | 3 (1 to 7 ) | -0.64 (-1.26 to -0.02 ) | 0.043 |
| Yangzhou | 3 (2 to 5 ) | 3 (1 to 8 ) | -0.46 (-0.88 to -0.03 ) | 0.036 | 3 (2 to 5 ) | 3 (1 to 8 ) | -0.41 (-0.85 to 0.02 ) | 0.065 | 3 (2 to 5 ) | 3 (1 to 8 ) | -0.44 (-0.86 to 0.0 ) | 0.047 |
| Zhenjiang | 3 (2 to 5 ) | 3 (1 to 7 ) | -0.42 (-0.86 to 0.03 ) | 0.066 | 3 (2 to 6 ) | 3 (1 to 8 ) | -0.37 (-0.79 to 0.06 ) | 0.095 | 3 (2 to 5 ) | 3 (1 to 8 ) | -0.39 (-0.83 to 0.04 ) | 0.078 |
| Jiangxi |  |  |  |  |  |  |  |  |  |  |  |  |
| Fuzhou | 4 (2 to 6 ) | 3 (1 to 9 ) | -0.67 (-1.29 to -0.05 ) | 0.034 | 4 (2 to 6 ) | 3 (1 to 9 ) | -0.71 (-1.57 to 0.17 ) | 0.113 | 4 (2 to 6 ) | 3 (1 to 9 ) | -0.74 (-1.63 to 0.15 ) | 0.105 |
| Ganzhou | 4 (2 to 7 ) | 3 (1 to 9 ) | -0.97 (-1.51 to -0.43 ) | <.001 | 4 (2 to 7 ) | 3 (1 to 9 ) | -0.92 (-1.45 to -0.39 ) | 0.001 | 4 (2 to 7 ) | 3 (1 to 9 ) | -0.95 (-1.48 to -0.41 ) | 0.001 |
| Ji'an | 4 (2 to 6 ) | 3 (1 to 9 ) | -0.93 (-1.53 to -0.32 ) | 0.003 | 4 (2 to 7 ) | 3 (1 to 9 ) | -0.89 (-1.75 to -0.03 ) | 0.043 | 4 (2 to 6 ) | 3 (1 to 9 ) | -0.90 (-1.51 to -0.28 ) | 0.005 |
| Jingdezhen | 3 (1 to 6 ) | 3 (1 to 9 ) | -0.47 (-0.92 to -0.01 ) | 0.047 | 3 (1 to 6 ) | 3 (1 to 9 ) | -0.42 (-0.87 to 0.04 ) | 0.076 | 3 (1 to 6 ) | 3 (1 to 9 ) | -0.44 (-0.90 to 0.02 ) | 0.060 |
| Jiujiang | 3 (1 to 5 ) | 3 (1 to 8 ) | -0.59 (-1.07 to -0.12 ) | 0.015 | 3 (2 to 6 ) | 3 (1 to 9 ) | -0.54 (-0.98 to -0.10 ) | 0.017 | 3 (1 to 6 ) | 3 (1 to 9 ) | -0.57 (-1.02 to -0.11 ) | 0.016 |
| Nanchang | 3 (1 to 5 ) | 3 (1 to 8 ) | -0.77 (-1.58 to 0.04 ) | 0.063 | 3 (2 to 5 ) | 3 (1 to 8 ) | -0.75 (-0.99 to -0.52 ) | <.001 | 3 (1 to 5 ) | 3 (1 to 8 ) | -0.74 (-1.53 to 0.06 ) | 0.071 |
| Pingxiang | 4 (2 to 6 ) | 3 (1 to 9 ) | -0.82 (-2.13 to 0.51 ) | 0.225 | 4 (2 to 6 ) | 3 (1 to 9 ) | -0.75 (-2.10 to 0.61 ) | 0.279 | 4 (2 to 6 ) | 3 (1 to 9 ) | -0.79 (-2.11 to 0.56 ) | 0.250 |
| Shangrao | 3 (1 to 6 ) | 3 (1 to 8 ) | -0.40 (-1.50 to 0.72 ) | 0.484 | 3 (1 to 6 ) | 3 (1 to 9 ) | -0.33 (-1.40 to 0.75 ) | 0.544 | 3 (1 to 6 ) | 3 (1 to 9 ) | -0.37 (-1.45 to 0.73 ) | 0.512 |
| Xinyu | 4 (2 to 6 ) | 3 (1 to 9 ) | -0.98 (-1.40 to -0.55 ) | <.001 | 4 (2 to 6 ) | 3 (1 to 9 ) | -0.93 (-1.35 to -0.50 ) | <.001 | 4 (2 to 6 ) | 3 (1 to 9 ) | -0.95 (-1.37 to -0.53 ) | <.001 |
| Yichun | 4 (2 to 6 ) | 3 (1 to 9 ) | -0.82 (-1.25 to -0.39 ) | <.001 | 4 (2 to 6 ) | 3 (1 to 10 ) | -0.77 (-1.20 to -0.33 ) | 0.001 | 4 (2 to 6 ) | 3 (1 to 9 ) | -0.80 (-1.23 to -0.36 ) | <.001 |
| Yingtan | 3 (1 to 6 ) | 3 (1 to 9 ) | -0.44 (-1.18 to 0.30 ) | 0.244 | 3 (2 to 6 ) | 3 (1 to 9 ) | -0.38 (-1.07 to 0.32 ) | 0.285 | 3 (1 to 6 ) | 3 (1 to 9 ) | -0.41 (-1.13 to 0.31 ) | 0.262 |
| Jilin |  |  |  |  |  |  |  |  |  |  |  |  |
| Baicheng | 4 (2 to 7 ) | 3 (1 to 10 ) | -1.92 (-2.92 to -0.90 ) | <.001 | 4 (2 to 7 ) | 3 (1 to 10 ) | -1.86 (-2.84 to -0.88 ) | <.001 | 4 (2 to 7 ) | 3 (1 to 10 ) | -1.89 (-2.88 to -0.89 ) | <.001 |
| Baishan | 3 (1 to 6 ) | 3 (1 to 11 ) | 0.29 (-0.74 to 1.32 ) | 0.584 | 3 (1 to 7 ) | 3 (1 to 11 ) | 0.21 (-0.63 to 1.06 ) | 0.619 | 3 (1 to 6 ) | 3 (1 to 11 ) | 0.19 (-0.65 to 1.04 ) | 0.657 |
| Changchun | 3 (2 to 6 ) | 3 (1 to 9 ) | -0.55 (-0.82 to -0.27 ) | <.001 | 3 (2 to 6 ) | 3 (1 to 9 ) | -0.52 (-0.81 to -0.24 ) | <.001 | 3 (2 to 6 ) | 3 (1 to 9 ) | -0.53 (-0.80 to -0.26 ) | <.001 |
| Jilin | 3 (1 to 6 ) | 3 (1 to 10 ) | -0.03 (-0.28 to 0.22 ) | 0.817 | 3 (1 to 6 ) | 3 (1 to 11 ) | 0.03 (-0.22 to 0.28 ) | 0.830 | 3 (1 to 6 ) | 3 (1 to 10 ) | 0.0 (-0.25 to 0.25 ) | 0.987 |
| Liaoyuan | 3 (2 to 6 ) | 3 (1 to 11 ) | -0.54 (-0.96 to -0.12 ) | 0.013 | 3 (2 to 6 ) | 3 (1 to 11 ) | -0.47 (-0.93 to -0.01 ) | 0.046 | 3 (2 to 6 ) | 3 (1 to 11 ) | -0.51 (-0.94 to -0.07 ) | 0.024 |
| Siping | 3 (2 to 6 ) | 3 (1 to 10 ) | -1.04 (-1.32 to -0.76 ) | <.001 | 4 (2 to 7 ) | 3 (1 to 10 ) | -0.98 (-1.26 to -0.69 ) | <.001 | 4 (2 to 6 ) | 3 (1 to 10 ) | -1.01 (-1.29 to -0.73 ) | <.001 |
| Songyuan | 4 (2 to 6 ) | 3 (1 to 9 ) | -1.09 (-1.70 to -0.47 ) | 0.001 | 4 (2 to 7 ) | 3 (1 to 10 ) | -1.03 (-1.62 to -0.45 ) | 0.001 | 4 (2 to 6 ) | 3 (1 to 10 ) | -1.06 (-1.66 to -0.46 ) | 0.001 |
| Tonghua | 3 (2 to 6 ) | 3 (1 to 10 ) | -0.27 (-1.05 to 0.51 ) | 0.494 | 3 (2 to 6 ) | 3 (1 to 11 ) | -0.23 (-1.01 to 0.56 ) | 0.571 | 3 (2 to 6 ) | 3 (1 to 10 ) | -0.25 (-1.03 to 0.53 ) | 0.530 |
| Yanbian Korean | 3 (1 to 6 ) | 3 (1 to 10 ) | 0.87 (0.15 to 1.60 ) | 0.018 | 3 (1 to 6 ) | 3 (1 to 11 ) | 0.92 (0.17 to 1.68 ) | 0.016 | 3 (1 to 6 ) | 3 (1 to 11 ) | 0.90 (0.16 to 1.64 ) | 0.017 |
| Liaoning |  |  |  |  |  |  |  |  |  |  |  |  |
| Anshan | 4 (2 to 7 ) | 4 (1 to 12 ) | -0.31 (-0.44 to -0.18 ) | <.001 | 4 (2 to 7 ) | 4 (1 to 13 ) | -0.27 (-0.44 to -0.11 ) | 0.001 | 4 (2 to 7 ) | 4 (1 to 12 ) | -0.30 (-0.44 to -0.15 ) | <.001 |
| Benxi | 4 (2 to 8 ) | 4 (1 to 14 ) | -0.06 (-0.36 to 0.24 ) | 0.696 | 5 (2 to 8 ) | 5 (1 to 14 ) | -0.01 (-0.30 to 0.27 ) | 0.931 | 5 (2 to 8 ) | 5 (1 to 14 ) | -0.04 (-0.33 to 0.26 ) | 0.806 |
| Chaoyang | 5 (3 to 8 ) | 4 (1 to 12 ) | -0.85 (-1.48 to -0.21 ) | 0.009 | 5 (3 to 8 ) | 4 (1 to 13 ) | -0.81 (-1.45 to -0.16 ) | 0.015 | 5 (3 to 8 ) | 4 (1 to 13 ) | -0.83 (-1.47 to -0.19 ) | 0.012 |
| Dalian | 3 (2 to 6 ) | 4 (1 to 11 ) | 0.72 (-1.03 to 2.50 ) | 0.423 | 4 (2 to 6 ) | 4 (1 to 11 ) | 0.62 (-1.01 to 2.28 ) | 0.457 | 3 (2 to 6 ) | 4 (1 to 11 ) | 0.67 (-0.75 to 2.12 ) | 0.356 |
| Dandong | 4 (2 to 8 ) | 4 (1 to 13 ) | 0.22 (-0.32 to 0.77 ) | 0.421 | 4 (2 to 8 ) | 5 (1 to 14 ) | 0.28 (-0.29 to 0.85 ) | 0.336 | 4 (2 to 8 ) | 5 (1 to 13 ) | 0.25 (-0.31 to 0.81 ) | 0.377 |
| Fushun | 5 (2 to 8 ) | 5 (1 to 14 ) | -0.20 (-0.46 to 0.06 ) | 0.131 | 5 (3 to 9 ) | 5 (1 to 15 ) | -0.16 (-0.41 to 0.10 ) | 0.238 | 5 (2 to 8 ) | 5 (1 to 15 ) | -0.18 (-0.44 to 0.08 ) | 0.175 |
| Fuxin | 5 (3 to 8 ) | 4 (1 to 13 ) | -1.18 (-1.85 to -0.50 ) | 0.001 | 5 (3 to 9 ) | 4 (1 to 13 ) | -1.23 (-2.02 to -0.44 ) | 0.002 | 5 (3 to 9 ) | 4 (1 to 13 ) | -1.26 (-2.06 to -0.46 ) | 0.002 |
| Huludao | 4 (2 to 7 ) | 4 (1 to 12 ) | -0.34 (-0.58 to -0.11 ) | 0.004 | 5 (3 to 7 ) | 4 (1 to 13 ) | -0.29 (-0.52 to -0.06 ) | 0.013 | 5 (2 to 7 ) | 4 (1 to 13 ) | -0.32 (-0.55 to -0.09 ) | 0.007 |
| Jinzhou | 5 (3 to 7 ) | 4 (1 to 12 ) | -0.94 (-1.25 to -0.63 ) | <.001 | 5 (3 to 8 ) | 4 (1 to 12 ) | -0.89 (-1.17 to -0.60 ) | <.001 | 5 (3 to 8 ) | 4 (1 to 12 ) | -0.92 (-1.22 to -0.62 ) | <.001 |
| Liaoyang | 4 (2 to 7 ) | 4 (1 to 12 ) | -0.36 (-0.52 to -0.20 ) | <.001 | 5 (2 to 8 ) | 4 (1 to 13 ) | -0.31 (-0.48 to -0.13 ) | 0.001 | 4 (2 to 8 ) | 4 (1 to 12 ) | -0.33 (-0.50 to -0.17 ) | <.001 |
| Panjin | 4 (2 to 7 ) | 4 (1 to 11 ) | -0.62 (-0.85 to -0.39 ) | <.001 | 4 (3 to 7 ) | 4 (1 to 12 ) | -0.56 (-0.78 to -0.35 ) | <.001 | 4 (2 to 7 ) | 4 (1 to 12 ) | -0.59 (-0.82 to -0.37 ) | <.001 |
| Shenyang | 4 (2 to 7 ) | 4 (1 to 11 ) | -0.67 (-0.94 to -0.40 ) | <.001 | 4 (2 to 7 ) | 4 (1 to 12 ) | -0.62 (-0.90 to -0.34 ) | <.001 | 4 (2 to 7 ) | 4 (1 to 11 ) | -0.65 (-0.92 to -0.37 ) | <.001 |
| Tieling | 5 (3 to 8 ) | 4 (1 to 13 ) | -0.43 (-0.89 to 0.02 ) | 0.062 | 5 (3 to 8 ) | 4 (1 to 13 ) | -0.40 (-0.79 to 0.00 ) | 0.052 | 5 (3 to 8 ) | 4 (1 to 13 ) | -0.40 (-0.85 to 0.04 ) | 0.073 |
| Inner Mongolia |  |  |  |  |  |  |  |  |  |  |  |  |
| Alxa | 3 (1 to 4 ) | 2 (1 to 6 ) | -0.35 (-1.77 to 1.09 ) | 0.628 | 3 (1 to 4 ) | 2 (1 to 6 ) | -0.31 (-1.73 to 1.13 ) | 0.671 | 3 (1 to 4 ) | 2 (1 to 6 ) | -0.33 (-1.75 to 1.11 ) | 0.649 |
| Baotou | 2 (1 to 4 ) | 2 (1 to 5 ) | -0.97 (-2.45 to 0.53 ) | 0.204 | 3 (1 to 4 ) | 2 (1 to 6 ) | -0.91 (-2.39 to 0.61 ) | 0.239 | 2 (1 to 4 ) | 2 (1 to 5 ) | -0.94 (-2.42 to 0.57 ) | 0.220 |
| Baynnur | 3 (2 to 4 ) | 2 (1 to 6 ) | -1.17 (-2.79 to 0.47 ) | 0.159 | 3 (2 to 5 ) | 2 (1 to 6 ) | -1.11 (-2.73 to 0.53 ) | 0.183 | 3 (2 to 5 ) | 2 (1 to 6 ) | -1.14 (-2.76 to 0.50 ) | 0.171 |
| Chifeng | 2 (1 to 4 ) | 2 (0 to 7 ) | -0.51 (-1.19 to 0.18 ) | 0.147 | 2 (1 to 4 ) | 2 (1 to 7 ) | -0.45 (-1.12 to 0.23 ) | 0.191 | 2 (1 to 4 ) | 2 (1 to 7 ) | -0.48 (-1.16 to 0.20 ) | 0.167 |
| Hohhot | 2 (1 to 4 ) | 2 (1 to 6 ) | -0.51 (-1.45 to 0.43 ) | 0.287 | 2 (1 to 4 ) | 2 (1 to 6 ) | -0.44 (-1.40 to 0.52 ) | 0.365 | 2 (1 to 4 ) | 2 (1 to 6 ) | -0.48 (-1.43 to 0.47 ) | 0.323 |
| Hulunbuir | 3 (1 to 5 ) | 2 (1 to 8 ) | -0.85 (-1.61 to -0.08 ) | 0.030 | 3 (1 to 5 ) | 3 (1 to 8 ) | -0.80 (-1.57 to -0.03 ) | 0.041 | 3 (1 to 5 ) | 3 (1 to 8 ) | -0.83 (-1.59 to -0.06 ) | 0.035 |
| Ordos | 2 (1 to 4 ) | 2 (1 to 6 ) | -0.21 (-1.04 to 0.64 ) | 0.632 | 2 (1 to 4 ) | 2 (1 to 6 ) | -0.16 (-1.00 to 0.68 ) | 0.707 | 2 (1 to 4 ) | 2 (1 to 6 ) | -0.18 (-1.02 to 0.66 ) | 0.668 |
| Tongliao | 2 (1 to 4 ) | 2 (0 to 7 ) | -0.43 (-1.36 to 0.50 ) | 0.362 | 2 (1 to 4 ) | 2 (0 to 7 ) | -0.38 (-1.30 to 0.56 ) | 0.429 | 2 (1 to 4 ) | 2 (0 to 7 ) | -0.41 (-1.33 to 0.53 ) | 0.394 |
| Ulaan Chab | 2 (1 to 3 ) | 2 (1 to 6 ) | -0.25 (-0.63 to 0.13 ) | 0.190 | 2 (1 to 4 ) | 2 (1 to 7 ) | -0.20 (-0.59 to 0.18 ) | 0.294 | 2 (1 to 3 ) | 2 (1 to 6 ) | -0.23 (-0.61 to 0.15 ) | 0.236 |
| Wuhai | 3 (1 to 4 ) | 2 (1 to 6 ) | -0.65 (-2.20 to 0.93 ) | 0.420 | 3 (1 to 4 ) | 2 (1 to 7 ) | -0.60 (-2.15 to 0.97 ) | 0.452 | 3 (1 to 4 ) | 2 (1 to 6 ) | -0.62 (-2.17 to 0.95 ) | 0.435 |
| Xilin Gol | 3 (2 to 5 ) | 2 (1 to 7 ) | -1.80 (-2.63 to -0.97 ) | <.001 | 3 (2 to 5 ) | 2 (1 to 7 ) | -1.76 (-2.58 to -0.93 ) | <.001 | 3 (2 to 5 ) | 2 (1 to 7 ) | -1.78 (-2.61 to -0.95 ) | <.001 |
| Xing'an | 3 (1 to 5 ) | 2 (1 to 8 ) | -0.68 (-2.20 to 0.86 ) | 0.384 | 3 (1 to 5 ) | 3 (1 to 8 ) | -0.62 (-2.12 to 0.91 ) | 0.426 | 3 (1 to 5 ) | 2 (1 to 8 ) | -0.65 (-2.16 to 0.88 ) | 0.404 |
| Ningxia |  |  |  |  |  |  |  |  |  |  |  |  |
| Guyuan | 2 (1 to 4 ) | 3 (1 to 7 ) | 0.24 (-0.37 to 0.86 ) | 0.433 | 2 (1 to 4 ) | 3 (1 to 7 ) | 0.24 (-0.40 to 0.90 ) | 0.463 | 2 (1 to 4 ) | 3 (1 to 7 ) | 0.27 (-0.35 to 0.89 ) | 0.398 |
| Shizuishan | 3 (2 to 6 ) | 3 (1 to 7 ) | -1.30 (-1.81 to -0.78 ) | <.001 | 3 (2 to 6 ) | 3 (1 to 7 ) | -1.25 (-1.77 to -0.74 ) | <.001 | 3 (2 to 6 ) | 3 (1 to 7 ) | -1.28 (-1.79 to -0.76 ) | <.001 |
| Wuzhong | 3 (1 to 5 ) | 3 (1 to 7 ) | -0.69 (-0.76 to -0.61 ) | <.001 | 3 (1 to 5 ) | 3 (1 to 7 ) | -0.65 (-0.74 to -0.55 ) | <.001 | 3 (1 to 5 ) | 3 (1 to 7 ) | -0.67 (-0.74 to -0.59 ) | <.001 |
| Yinchuan | 3 (1 to 6 ) | 2 (1 to 7 ) | -1.26 (-1.66 to -0.87 ) | <.001 | 3 (2 to 6 ) | 3 (1 to 7 ) | -1.22 (-1.61 to -0.83 ) | <.001 | 3 (1 to 6 ) | 3 (1 to 7 ) | -1.24 (-1.63 to -0.85 ) | <.001 |
| Zhongwei | 3 (1 to 5 ) | 3 (1 to 7 ) | -0.48 (-0.83 to -0.14 ) | 0.006 | 3 (1 to 5 ) | 3 (1 to 7 ) | -0.45 (-0.73 to -0.18 ) | 0.001 | 3 (1 to 5 ) | 3 (1 to 7 ) | -0.45 (-0.80 to -0.10 ) | 0.012 |
| Qinghai |  |  |  |  |  |  |  |  |  |  |  |  |
| Golog Tibetan | 4 (1 to 7 ) | 4 (1 to 10 ) | 0.60 (-0.33 to 1.53 ) | 0.205 | 4 (1 to 7 ) | 4 (1 to 11 ) | 0.67 (-0.32 to 1.66 ) | 0.186 | 4 (1 to 7 ) | 4 (1 to 11 ) | 0.63 (-0.32 to 1.59 ) | 0.195 |
| Gyêgu Tibetan | 3 (1 to 6 ) | 4 (1 to 9 ) | 0.54 (-0.66 to 1.75 ) | 0.381 | 3 (1 to 6 ) | 4 (1 to 10 ) | 0.59 (-0.58 to 1.78 ) | 0.322 | 3 (1 to 6 ) | 4 (1 to 9 ) | 0.56 (-0.62 to 1.76 ) | 0.352 |
| Haibei Tibetan | 4 (2 to 7 ) | 3 (1 to 8 ) | -0.86 (-1.53 to -0.18 ) | 0.013 | 4 (2 to 8 ) | 3 (1 to 9 ) | -0.80 (-1.46 to -0.13 ) | 0.019 | 4 (2 to 8 ) | 3 (1 to 9 ) | -0.83 (-1.49 to -0.16 ) | 0.016 |
| Haidong | 4 (2 to 7 ) | 3 (1 to 7 ) | -0.88 (-1.40 to -0.36 ) | 0.001 | 4 (2 to 7 ) | 3 (1 to 8 ) | -0.82 (-1.36 to -0.28 ) | 0.003 | 4 (2 to 7 ) | 3 (1 to 8 ) | -0.85 (-1.38 to -0.32 ) | 0.002 |
| Hainan Tibetan | 4 (2 to 7 ) | 3 (1 to 9 ) | -0.52 (-0.79 to -0.24 ) | <.001 | 4 (2 to 8 ) | 4 (1 to 10 ) | -0.45 (-0.73 to -0.16 ) | 0.002 | 4 (2 to 7 ) | 4 (1 to 9 ) | -0.48 (-0.76 to -0.21 ) | 0.001 |
| Haixi Mongol and Tibetan | 4 (1 to 8 ) | 4 (1 to 9 ) | -0.60 (-2.06 to 0.88 ) | 0.423 | 4 (2 to 8 ) | 4 (1 to 10 ) | -0.54 (-1.97 to 0.90 ) | 0.459 | 4 (2 to 8 ) | 4 (1 to 9 ) | -0.57 (-2.02 to 0.89 ) | 0.440 |
| Huangnan Tibetan | 4 (2 to 7 ) | 4 (1 to 9 ) | -0.28 (-0.84 to 0.29 ) | 0.337 | 4 (2 to 7 ) | 4 (1 to 10 ) | -0.24 (-0.81 to 0.33 ) | 0.409 | 4 (2 to 7 ) | 4 (1 to 10 ) | -0.26 (-0.83 to 0.31 ) | 0.371 |
| Xining | 4 (2 to 7 ) | 3 (1 to 7 ) | -1.16 (-1.86 to -0.46 ) | 0.001 | 4 (2 to 7 ) | 3 (1 to 7 ) | -1.15 (-1.54 to -0.76 ) | <.001 | 4 (2 to 7 ) | 3 (1 to 7 ) | -1.13 (-1.84 to -0.42 ) | 0.002 |
| Shaanxi |  |  |  |  |  |  |  |  |  |  |  |  |
| Ankang | 3 (2 to 6 ) | 3 (1 to 9 ) | 0.08 (-1.64 to 1.82 ) | 0.931 | 4 (2 to 6 ) | 4 (1 to 10 ) | 0.12 (-1.61 to 1.88 ) | 0.893 | 3 (2 to 6 ) | 4 (1 to 10 ) | 0.10 (-1.62 to 1.84 ) | 0.913 |
| Baoji | 4 (2 to 7 ) | 3 (1 to 9 ) | -0.83 (-1.53 to -0.12 ) | 0.022 | 4 (2 to 7 ) | 3 (1 to 9 ) | -0.77 (-1.56 to 0.02 ) | 0.055 | 4 (2 to 7 ) | 3 (1 to 9 ) | -0.81 (-1.60 to -0.01 ) | 0.046 |
| Hanzhong | 4 (2 to 7 ) | 3 (1 to 10 ) | -0.33 (-1.05 to 0.39 ) | 0.370 | 4 (2 to 7 ) | 4 (1 to 10 ) | -0.26 (-1.01 to 0.49 ) | 0.498 | 4 (2 to 7 ) | 4 (1 to 10 ) | -0.30 (-1.03 to 0.44 ) | 0.428 |
| Shangluo | 3 (2 to 6 ) | 3 (1 to 9 ) | 0.41 (-0.49 to 1.32 ) | 0.372 | 3 (2 to 6 ) | 4 (1 to 10 ) | 0.46 (-0.45 to 1.37 ) | 0.324 | 3 (2 to 6 ) | 4 (1 to 10 ) | 0.43 (-0.47 to 1.35 ) | 0.348 |
| Tongchuan | 3 (2 to 6 ) | 3 (1 to 8 ) | -0.47 (-0.85 to -0.10 ) | 0.013 | 3 (2 to 6 ) | 3 (1 to 9 ) | -0.43 (-0.80 to -0.06 ) | 0.023 | 3 (2 to 6 ) | 3 (1 to 9 ) | -0.45 (-0.82 to -0.08 ) | 0.017 |
| Weinan | 3 (2 to 6 ) | 3 (1 to 9 ) | -0.25 (-0.48 to -0.02 ) | 0.031 | 3 (2 to 6 ) | 3 (1 to 9 ) | -0.23 (-0.44 to -0.02 ) | 0.033 | 3 (2 to 6 ) | 3 (1 to 9 ) | -0.21 (-0.45 to 0.02 ) | 0.068 |
| Xi'an | 3 (2 to 5 ) | 3 (1 to 7 ) | -0.67 (-1.06 to -0.27 ) | 0.001 | 3 (2 to 5 ) | 3 (1 to 8 ) | -0.62 (-1.00 to -0.24 ) | 0.001 | 3 (2 to 5 ) | 3 (1 to 8 ) | -0.64 (-1.03 to -0.26 ) | 0.001 |
| Xianyang | 3 (2 to 6 ) | 3 (1 to 8 ) | -0.62 (-1.19 to -0.04 ) | 0.035 | 4 (2 to 6 ) | 3 (1 to 9 ) | -0.56 (-1.13 to 0.01 ) | 0.054 | 3 (2 to 6 ) | 3 (1 to 8 ) | -0.59 (-1.16 to -0.02 ) | 0.043 |
| Yan'an | 3 (2 to 6 ) | 3 (1 to 8 ) | -0.63 (-1.73 to 0.47 ) | 0.258 | 3 (2 to 6 ) | 3 (1 to 8 ) | -0.59 (-1.68 to 0.51 ) | 0.292 | 3 (2 to 6 ) | 3 (1 to 8 ) | -0.61 (-1.70 to 0.49 ) | 0.274 |
| Yulin | 4 (2 to 6 ) | 3 (1 to 7 ) | -1.29 (-2.38 to -0.19 ) | 0.022 | 4 (2 to 7 ) | 3 (1 to 8 ) | -1.25 (-2.34 to -0.14 ) | 0.027 | 4 (2 to 6 ) | 3 (1 to 8 ) | -1.27 (-2.36 to -0.16 ) | 0.024 |
| Shandong |  |  |  |  |  |  |  |  |  |  |  |  |
| Binzhou | 3 (2 to 4 ) | 2 (1 to 6 ) | -0.92 (-1.70 to -0.13 ) | 0.022 | 3 (2 to 5 ) | 2 (1 to 6 ) | -0.87 (-1.65 to -0.09 ) | 0.029 | 3 (2 to 4 ) | 2 (1 to 6 ) | -0.90 (-1.67 to -0.11 ) | 0.025 |
| Dezhou | 2 (1 to 4 ) | 2 (1 to 6 ) | -0.78 (-1.60 to 0.06 ) | 0.068 | 3 (2 to 4 ) | 2 (1 to 6 ) | -0.73 (-1.55 to 0.10 ) | 0.084 | 3 (1 to 4 ) | 2 (1 to 6 ) | -0.75 (-1.58 to 0.08 ) | 0.075 |
| Dongying | 3 (2 to 4 ) | 2 (0 to 6 ) | -1.11 (-1.89 to -0.31 ) | 0.006 | 3 (2 to 4 ) | 2 (0 to 6 ) | -1.06 (-1.84 to -0.27 ) | 0.008 | 3 (2 to 4 ) | 2 (0 to 6 ) | -1.08 (-1.87 to -0.29 ) | 0.007 |
| Heze | 2 (1 to 4 ) | 2 (1 to 6 ) | -0.62 (-1.27 to 0.03 ) | 0.061 | 2 (1 to 4 ) | 2 (1 to 6 ) | -0.58 (-1.22 to 0.07 ) | 0.081 | 2 (1 to 4 ) | 2 (1 to 6 ) | -0.60 (-1.25 to 0.05 ) | 0.070 |
| Jinan | 2 (1 to 4 ) | 2 (0 to 5 ) | -0.71 (-1.29 to -0.13 ) | 0.017 | 2 (1 to 4 ) | 2 (0 to 6 ) | -0.67 (-1.23 to -0.10 ) | 0.021 | 2 (1 to 4 ) | 2 (0 to 6 ) | -0.69 (-1.26 to -0.11 ) | 0.019 |
| Jining | 2 (1 to 4 ) | 2 (1 to 6 ) | -0.55 (-0.80 to -0.30 ) | <.001 | 2 (1 to 4 ) | 2 (1 to 6 ) | -0.51 (-0.75 to -0.26 ) | <.001 | 2 (1 to 4 ) | 2 (1 to 6 ) | -0.53 (-0.78 to -0.28 ) | <.001 |
| Laiwu | 2 (1 to 4 ) | 2 (0 to 5 ) | -0.53 (-0.78 to -0.27 ) | <.001 | 2 (1 to 4 ) | 2 (0 to 6 ) | -0.48 (-0.67 to -0.28 ) | <.001 | 2 (1 to 4 ) | 2 (0 to 6 ) | -0.51 (-0.76 to -0.25 ) | <.001 |
| Liaocheng | 2 (1 to 4 ) | 2 (0 to 6 ) | -0.81 (-2.05 to 0.44 ) | 0.201 | 3 (1 to 4 ) | 2 (0 to 6 ) | -0.86 (-1.92 to 0.21 ) | 0.113 | 2 (1 to 4 ) | 2 (0 to 6 ) | -0.79 (-2.04 to 0.46 ) | 0.214 |
| Linyi | 2 (1 to 4 ) | 2 (1 to 6 ) | -0.09 (-0.78 to 0.61 ) | 0.807 | 2 (1 to 4 ) | 2 (1 to 6 ) | -0.04 (-0.75 to 0.66 ) | 0.902 | 2 (1 to 4 ) | 2 (1 to 6 ) | -0.07 (-0.76 to 0.64 ) | 0.853 |
| Qingdao | 2 (1 to 4 ) | 2 (0 to 5 ) | -0.78 (-1.30 to -0.27 ) | 0.003 | 2 (1 to 4 ) | 2 (0 to 5 ) | -0.74 (-1.25 to -0.22 ) | 0.005 | 2 (1 to 4 ) | 2 (0 to 5 ) | -0.76 (-1.28 to -0.25 ) | 0.004 |
| Rizhao | 2 (1 to 4 ) | 2 (1 to 6 ) | -0.27 (-1.15 to 0.63 ) | 0.558 | 2 (1 to 4 ) | 2 (1 to 6 ) | -0.23 (-1.11 to 0.66 ) | 0.614 | 2 (1 to 4 ) | 2 (1 to 6 ) | -0.25 (-1.13 to 0.64 ) | 0.585 |
| Tai'an | 2 (1 to 4 ) | 2 (0 to 6 ) | -0.41 (-0.68 to -0.14 ) | 0.003 | 2 (1 to 4 ) | 2 (1 to 6 ) | -0.40 (-0.68 to -0.12 ) | 0.006 | 2 (1 to 4 ) | 2 (1 to 6 ) | -0.39 (-0.66 to -0.12 ) | 0.005 |
| Weifang | 2 (1 to 4 ) | 2 (0 to 5 ) | -0.82 (-1.24 to -0.41 ) | <.001 | 2 (1 to 4 ) | 2 (0 to 6 ) | -0.78 (-1.19 to -0.36 ) | <.001 | 2 (1 to 4 ) | 2 (0 to 5 ) | -0.80 (-1.22 to -0.39 ) | <.001 |
| Weihai | 2 (1 to 4 ) | 2 (0 to 5 ) | -1.36 (-2.70 to -0.01 ) | 0.049 | 3 (1 to 5 ) | 2 (0 to 6 ) | -1.29 (-2.59 to 0.02 ) | 0.054 | 3 (1 to 5 ) | 2 (0 to 5 ) | -1.33 (-2.65 to 0.01 ) | 0.051 |
| Yantai | 3 (1 to 4 ) | 2 (0 to 6 ) | -1.37 (-2.78 to 0.06 ) | 0.061 | 3 (2 to 5 ) | 2 (0 to 6 ) | -1.30 (-2.67 to 0.10 ) | 0.068 | 3 (1 to 4 ) | 2 (0 to 6 ) | -1.33 (-2.73 to 0.08 ) | 0.064 |
| Zaozhuang | 2 (1 to 4 ) | 2 (0 to 6 ) | -0.14 (-0.80 to 0.52 ) | 0.674 | 2 (1 to 4 ) | 2 (1 to 6 ) | -0.10 (-0.77 to 0.57 ) | 0.772 | 2 (1 to 4 ) | 2 (1 to 6 ) | -0.12 (-0.79 to 0.55 ) | 0.721 |
| Zibo | 2 (1 to 4 ) | 2 (0 to 5 ) | -0.86 (-1.06 to -0.65 ) | <.001 | 2 (2 to 4 ) | 2 (1 to 6 ) | -0.81 (-1.23 to -0.38 ) | <.001 | 2 (1 to 4 ) | 2 (0 to 6 ) | -0.83 (-1.26 to -0.40 ) | <.001 |
| Shanghai |  |  |  |  |  |  |  |  |  |  |  |  |
| Shanghai | 3 (1 to 6 ) | 3 (1 to 8 ) | -0.34 (-0.35 to -0.32 ) | <.001 | 3 (1 to 6 ) | 3 (1 to 8 ) | -0.30 (-0.33 to -0.26 ) | <.001 | 3 (1 to 6 ) | 3 (1 to 8 ) | -0.32 (-0.34 to -0.30 ) | <.001 |
| Shanxi |  |  |  |  |  |  |  |  |  |  |  |  |
| Changzhi | 3 (2 to 5 ) | 3 (1 to 8 ) | 0.40 (-0.36 to 1.17 ) | 0.303 | 3 (2 to 5 ) | 3 (1 to 9 ) | 0.47 (-0.37 to 1.32 ) | 0.274 | 3 (2 to 5 ) | 3 (1 to 8 ) | 0.45 (-0.39 to 1.30 ) | 0.296 |
| Datong | 4 (2 to 6 ) | 3 (1 to 9 ) | -1.46 (-2.58 to -0.32 ) | 0.012 | 4 (2 to 7 ) | 3 (1 to 9 ) | -1.41 (-2.53 to -0.27 ) | 0.015 | 4 (2 to 6 ) | 3 (1 to 9 ) | -1.43 (-2.55 to -0.30 ) | 0.013 |
| Jincheng | 3 (2 to 5 ) | 3 (1 to 8 ) | 0.53 (-0.45 to 1.52 ) | 0.290 | 3 (2 to 5 ) | 3 (1 to 9 ) | 0.57 (-0.41 to 1.57 ) | 0.255 | 3 (2 to 5 ) | 3 (1 to 9 ) | 0.55 (-0.43 to 1.54 ) | 0.272 |
| Jinzhong | 3 (2 to 5 ) | 3 (1 to 8 ) | -0.19 (-0.41 to 0.03 ) | 0.094 | 3 (2 to 5 ) | 3 (1 to 9 ) | -0.19 (-0.54 to 0.16 ) | 0.291 | 3 (2 to 5 ) | 3 (1 to 8 ) | -0.23 (-0.61 to 0.16 ) | 0.248 |
| Linfen | 3 (2 to 6 ) | 3 (1 to 9 ) | -0.17 (-0.52 to 0.17 ) | 0.323 | 3 (2 to 6 ) | 3 (1 to 9 ) | -0.13 (-0.48 to 0.22 ) | 0.465 | 3 (2 to 6 ) | 3 (1 to 9 ) | -0.15 (-0.50 to 0.19 ) | 0.387 |
| Luliang | 4 (2 to 6 ) | 3 (1 to 8 ) | -0.76 (-1.06 to -0.45 ) | <.001 | 4 (2 to 6 ) | 3 (1 to 8 ) | -0.77 (-1.07 to -0.47 ) | <.001 | 4 (2 to 6 ) | 3 (1 to 8 ) | -0.74 (-1.04 to -0.43 ) | <.001 |
| Shuozhou | 4 (2 to 7 ) | 3 (1 to 9 ) | -1.44 (-3.33 to 0.48 ) | 0.139 | 4 (2 to 7 ) | 3 (1 to 9 ) | -1.40 (-3.26 to 0.50 ) | 0.147 | 4 (2 to 7 ) | 3 (1 to 9 ) | -1.42 (-3.30 to 0.49 ) | 0.143 |
| Taiyuan | 3 (2 to 5 ) | 3 (1 to 7 ) | -0.66 (-1.13 to -0.19 ) | 0.006 | 3 (2 to 6 ) | 3 (1 to 8 ) | -0.61 (-1.10 to -0.13 ) | 0.013 | 3 (2 to 5 ) | 3 (1 to 7 ) | -0.64 (-1.11 to -0.16 ) | 0.009 |
| Xinzhou | 4 (2 to 6 ) | 3 (1 to 8 ) | -0.98 (-1.35 to -0.61 ) | <.001 | 4 (2 to 7 ) | 3 (1 to 9 ) | -0.94 (-1.31 to -0.57 ) | <.001 | 4 (2 to 6 ) | 3 (1 to 8 ) | -0.96 (-1.33 to -0.59 ) | <.001 |
| Yangquan | 3 (2 to 5 ) | 3 (1 to 8 ) | 0.08 (-0.28 to 0.45 ) | 0.655 | 3 (2 to 5 ) | 3 (1 to 9 ) | 0.13 (-0.23 to 0.49 ) | 0.493 | 3 (2 to 5 ) | 3 (1 to 8 ) | 0.10 (-0.26 to 0.46 ) | 0.574 |
| Yuncheng | 3 (2 to 6 ) | 3 (1 to 9 ) | -0.06 (-1.16 to 1.05 ) | 0.914 | 3 (2 to 6 ) | 3 (1 to 10 ) | -0.01 (-1.10 to 1.09 ) | 0.985 | 3 (2 to 6 ) | 3 (1 to 10 ) | -0.04 (-1.13 to 1.07 ) | 0.948 |
| Sichuan* |  |  |  |  |  |  |  |  |  |  |  |  |
| Bazhong | 3 (2 to 6 ) | 3 (1 to 8 ) | -0.46 (-1.10 to 0.18 ) | 0.160 | 4 (2 to 6 ) | 3 (1 to 9 ) | -0.42 (-1.05 to 0.22 ) | 0.202 | 3 (2 to 6 ) | 3 (1 to 8 ) | -0.44 (-1.08 to 0.20 ) | 0.179 |
| Chengdu | 3 (2 to 6 ) | 3 (1 to 7 ) | -1.21 (-1.74 to -0.67 ) | <.001 | 3 (2 to 6 ) | 3 (1 to 8 ) | -1.26 (-1.94 to -0.57 ) | <.001 | 3 (2 to 6 ) | 3 (1 to 8 ) | -1.29 (-1.98 to -0.59 ) | <.001 |
| Dazhou | 3 (2 to 5 ) | 3 (1 to 8 ) | -0.40 (-1.69 to 0.90 ) | 0.544 | 3 (2 to 6 ) | 3 (1 to 9 ) | -0.36 (-1.65 to 0.95 ) | 0.591 | 3 (2 to 5 ) | 3 (1 to 8 ) | -0.38 (-1.67 to 0.93 ) | 0.567 |
| Deyang | 3 (2 to 6 ) | 3 (1 to 8 ) | -1.00 (-1.94 to -0.05 ) | 0.040 | 4 (2 to 7 ) | 3 (1 to 8 ) | -0.97 (-1.78 to -0.15 ) | 0.021 | 3 (2 to 6 ) | 3 (1 to 8 ) | -1.0 (-1.83 to -0.17 ) | 0.018 |
| Garzê Tibetan | 4 (2 to 7 ) | 4 (1 to 9 ) | -0.23 (-2.39 to 1.98 ) | 0.836 | 4 (2 to 8 ) | 4 (1 to 10 ) | -0.19 (-2.34 to 2.01 ) | 0.867 | 4 (2 to 7 ) | 4 (1 to 9 ) | -0.21 (-2.37 to 2.0 ) | 0.851 |
| Guang'an | 3 (2 to 6 ) | 3 (1 to 9 ) | -0.28 (-1.14 to 0.59 ) | 0.530 | 3 (2 to 6 ) | 3 (1 to 10 ) | -0.23 (-1.10 to 0.64 ) | 0.601 | 3 (2 to 6 ) | 3 (1 to 9 ) | -0.26 (-1.12 to 0.62 ) | 0.564 |
| Guangyuan | 4 (2 to 6 ) | 3 (1 to 9 ) | -0.48 (-1.17 to 0.21 ) | 0.173 | 4 (2 to 6 ) | 3 (1 to 9 ) | -0.43 (-1.12 to 0.26 ) | 0.219 | 4 (2 to 6 ) | 3 (1 to 9 ) | -0.46 (-1.15 to 0.23 ) | 0.194 |
| Leshan | 4 (2 to 6 ) | 3 (1 to 9 ) | -0.76 (-1.56 to 0.03 ) | 0.061 | 4 (2 to 7 ) | 3 (1 to 9 ) | -0.72 (-1.51 to 0.07 ) | 0.074 | 4 (2 to 6 ) | 3 (1 to 9 ) | -0.74 (-1.53 to 0.05 ) | 0.067 |
| Liangshan Yi | 3 (2 to 6 ) | 3 (1 to 9 ) | -0.52 (-1.33 to 0.29 ) | 0.208 | 4 (2 to 7 ) | 3 (1 to 9 ) | -0.48 (-1.28 to 0.33 ) | 0.249 | 4 (2 to 7 ) | 3 (1 to 9 ) | -0.50 (-1.30 to 0.31 ) | 0.227 |
| Luzhou | 3 (2 to 6 ) | 3 (1 to 9 ) | -0.52 (-1.22 to 0.19 ) | 0.153 | 3 (2 to 6 ) | 3 (1 to 9 ) | -0.47 (-1.18 to 0.24 ) | 0.193 | 3 (2 to 6 ) | 3 (1 to 9 ) | -0.50 (-1.20 to 0.22 ) | 0.171 |
| Meishan | 3 (2 to 6 ) | 3 (1 to 8 ) | -0.70 (-1.19 to -0.20 ) | 0.006 | 4 (2 to 7 ) | 3 (1 to 9 ) | -0.64 (-1.22 to -0.06 ) | 0.030 | 4 (2 to 7 ) | 3 (1 to 9 ) | -0.68 (-1.17 to -0.19 ) | 0.007 |
| Mianyang | 3 (2 to 6 ) | 3 (1 to 8 ) | -0.74 (-1.47 to -0.01 ) | 0.046 | 4 (2 to 7 ) | 3 (1 to 9 ) | -0.68 (-1.40 to 0.06 ) | 0.071 | 3 (2 to 6 ) | 3 (1 to 9 ) | -0.71 (-1.44 to 0.02 ) | 0.057 |
| Nanchong | 3 (2 to 6 ) | 3 (1 to 8 ) | -0.39 (-1.37 to 0.59 ) | 0.435 | 3 (2 to 6 ) | 3 (1 to 9 ) | -0.35 (-1.33 to 0.65 ) | 0.492 | 3 (2 to 6 ) | 3 (1 to 9 ) | -0.37 (-1.35 to 0.62 ) | 0.462 |
| Ngawa Tibetan and Qiang | 4 (2 to 7 ) | 3 (1 to 9 ) | -0.76 (-3.10 to 1.64 ) | 0.533 | 4 (2 to 8 ) | 4 (1 to 10 ) | -0.70 (-3.01 to 1.67 ) | 0.561 | 4 (2 to 7 ) | 3 (1 to 9 ) | -0.73 (-3.06 to 1.66 ) | 0.546 |
| Panzhihua | 4 (2 to 7 ) | 3 (1 to 9 ) | -0.95 (-2.64 to 0.77 ) | 0.277 | 4 (2 to 8 ) | 3 (1 to 9 ) | -0.90 (-2.58 to 0.81 ) | 0.298 | 4 (2 to 7 ) | 3 (1 to 9 ) | -0.93 (-2.61 to 0.79 ) | 0.287 |
| Suining | 3 (2 to 6 ) | 3 (1 to 9 ) | -0.48 (-1.12 to 0.17 ) | 0.145 | 4 (2 to 6 ) | 3 (1 to 9 ) | -0.42 (-1.05 to 0.21 ) | 0.193 | 3 (2 to 6 ) | 3 (1 to 9 ) | -0.45 (-1.09 to 0.19 ) | 0.166 |
| Ya'an | 4 (2 to 7 ) | 3 (1 to 9 ) | -0.93 (-2.46 to 0.62 ) | 0.236 | 4 (2 to 8 ) | 3 (1 to 9 ) | -0.89 (-2.41 to 0.65 ) | 0.257 | 4 (2 to 8 ) | 3 (1 to 9 ) | -0.91 (-2.44 to 0.63 ) | 0.246 |
| Yibin | 3 (2 to 6 ) | 3 (1 to 9 ) | -0.59 (-1.17 to 0.01 ) | 0.053 | 4 (2 to 6 ) | 3 (1 to 9 ) | -0.54 (-1.11 to 0.05 ) | 0.071 | 3 (2 to 6 ) | 3 (1 to 9 ) | -0.56 (-1.14 to 0.03 ) | 0.061 |
| Zigong | 3 (2 to 6 ) | 3 (1 to 9 ) | -0.58 (-0.99 to -0.17 ) | 0.005 | 4 (2 to 6 ) | 3 (1 to 9 ) | -0.53 (-0.93 to -0.14 ) | 0.009 | 3 (2 to 6 ) | 3 (1 to 9 ) | -0.56 (-0.96 to -0.16 ) | 0.007 |
| Ziyang | 3 (2 to 6 ) | 3 (1 to 9 ) | -0.46 (-1.05 to 0.13 ) | 0.128 | 3 (2 to 6 ) | 3 (1 to 9 ) | -0.41 (-1.01 to 0.19 ) | 0.177 | 3 (2 to 6 ) | 3 (1 to 9 ) | -0.44 (-1.03 to 0.16 ) | 0.150 |
| Tianjin |  |  |  |  |  |  |  |  |  |  |  |  |
| Tianjin | 3 (2 to 5 ) | 3 (1 to 8 ) | -0.44 (-0.46 to -0.42 ) | <.001 | 3 (2 to 5 ) | 3 (1 to 8 ) | -0.40 (-0.42 to -0.38 ) | <.001 | 3 (2 to 5 ) | 3 (1 to 8 ) | -0.43 (-0.47 to -0.38 ) | <.001 |
| Xinjiang |  |  |  |  |  |  |  |  |  |  |  |  |
| Aksu | 3 (1 to 9 ) | 3 (1 to 11 ) | -0.36 (-0.76 to 0.04 ) | 0.075 | 4 (1 to 10 ) | 3 (1 to 11 ) | -0.30 (-0.74 to 0.13 ) | 0.175 | 4 (1 to 9 ) | 3 (1 to 11 ) | -0.33 (-0.75 to 0.08 ) | 0.116 |
| Altay | 4 (2 to 7 ) | 3 (1 to 9 ) | -0.98 (-6.96 to 5.39 ) | 0.757 | 4 (2 to 8 ) | 4 (1 to 10 ) | -0.91 (-6.85 to 5.40 ) | 0.770 | 4 (2 to 8 ) | 3 (1 to 9 ) | -0.95 (-6.91 to 5.39 ) | 0.763 |
| Bayin'gholin Mongol | 3 (1 to 8 ) | 3 (1 to 10 ) | 0.00 (-1.09 to 1.10 ) | 0.999 | 4 (1 to 8 ) | 4 (1 to 10 ) | 0.06 (-1.05 to 1.17 ) | 0.921 | 3 (1 to 8 ) | 3 (1 to 10 ) | 0.03 (-1.07 to 1.13 ) | 0.961 |
| Bortala Mongol | 4 (1 to 10 ) | 3 (1 to 10 ) | -0.51 (-0.96 to -0.05 ) | 0.030 | 4 (1 to 10 ) | 4 (1 to 11 ) | -0.46 (-0.91 to 0.00 ) | 0.048 | 4 (1 to 10 ) | 4 (1 to 10 ) | -0.48 (-0.94 to -0.03 ) | 0.038 |
| Changji Hui | 3 (1 to 7 ) | 3 (1 to 8 ) | -0.28 (-3.94 to 3.51 ) | 0.881 | 3 (1 to 7 ) | 3 (1 to 9 ) | -0.22 (-3.84 to 3.54 ) | 0.907 | 3 (1 to 7 ) | 3 (1 to 9 ) | -0.25 (-3.89 to 3.52 ) | 0.893 |
| Hami | 4 (2 to 6 ) | 3 (1 to 9 ) | -0.40 (-3.17 to 2.45 ) | 0.780 | 4 (2 to 7 ) | 4 (1 to 10 ) | -0.34 (-3.11 to 2.50 ) | 0.810 | 4 (2 to 6 ) | 3 (1 to 10 ) | -0.37 (-3.14 to 2.47 ) | 0.794 |
| Ili Kazakh | 4 (1 to 10 ) | 3 (1 to 10 ) | -0.71 (-1.17 to -0.25 ) | 0.003 | 4 (1 to 10 ) | 3 (1 to 10 ) | -0.67 (-1.12 to -0.21 ) | 0.004 | 4 (1 to 10 ) | 3 (1 to 10 ) | -0.69 (-1.15 to -0.23 ) | 0.003 |
| Karamay | 3 (1 to 8 ) | 3 (1 to 10 ) | -0.52 (-2.81 to 1.82 ) | 0.661 | 4 (1 to 8 ) | 3 (1 to 10 ) | -0.45 (-2.72 to 1.86 ) | 0.697 | 4 (1 to 8 ) | 3 (1 to 10 ) | -0.49 (-2.76 to 1.84 ) | 0.678 |
| Kashgar | 4 (1 to 11 ) | 3 (0 to 9 ) | -0.89 (-2.20 to 0.43 ) | 0.185 | 4 (1 to 11 ) | 3 (0 to 9 ) | -0.87 (-2.40 to 0.68 ) | 0.271 | 4 (1 to 11 ) | 3 (0 to 9 ) | -0.89 (-2.42 to 0.66 ) | 0.259 |
| Khotan | 3 (1 to 10 ) | 3 (1 to 10 ) | -0.13 (-1.86 to 1.62 ) | 0.883 | 4 (1 to 10 ) | 3 (1 to 10 ) | -0.07 (-1.83 to 1.73 ) | 0.940 | 3 (1 to 10 ) | 3 (1 to 10 ) | -0.10 (-1.84 to 1.67 ) | 0.911 |
| Kizilsu Kirghiz | 3 (1 to 11 ) | 3 (0 to 10 ) | -0.95 (-2.59 to 0.73 ) | 0.265 | 4 (1 to 11 ) | 3 (0 to 10 ) | -0.90 (-2.53 to 0.76 ) | 0.287 | 3 (1 to 11 ) | 3 (0 to 10 ) | -0.92 (-2.56 to 0.74 ) | 0.276 |
| Shihezi | 3 (1 to 7 ) | 3 (1 to 8 ) | -0.51 (-3.09 to 2.13 ) | 0.701 | 3 (1 to 7 ) | 3 (1 to 9 ) | -0.45 (-2.99 to 2.16 ) | 0.733 | 3 (1 to 7 ) | 3 (1 to 9 ) | -0.48 (-3.04 to 2.14 ) | 0.716 |
| Tacheng | 4 (1 to 8 ) | 3 (1 to 10 ) | -0.52 (-2.44 to 1.44 ) | 0.599 | 4 (1 to 9 ) | 3 (1 to 10 ) | -0.46 (-2.35 to 1.47 ) | 0.639 | 4 (1 to 8 ) | 3 (1 to 10 ) | -0.49 (-2.40 to 1.45 ) | 0.618 |
| Turfan | 3 (2 to 7 ) | 3 (1 to 9 ) | -0.05 (-2.16 to 2.10 ) | 0.963 | 4 (2 to 7 ) | 4 (1 to 10 ) | 0.00 (-2.10 to 2.16 ) | 0.997 | 3 (2 to 7 ) | 3 (1 to 9 ) | -0.02 (-2.13 to 2.13 ) | 0.982 |
| rümqi | 3 (1 to 7 ) | 3 (1 to 8 ) | -0.41 (-3.54 to 2.83 ) | 0.804 | 3 (1 to 7 ) | 3 (1 to 8 ) | -0.34 (-3.45 to 2.87 ) | 0.832 | 3 (1 to 7 ) | 3 (1 to 8 ) | -0.37 (-3.50 to 2.85 ) | 0.817 |
| Xizang |  |  |  |  |  |  |  |  |  |  |  |  |
| Chamdo | 4 (1 to 8 ) | 3 (1 to 9 ) | -0.20 (-1.56 to 1.19 ) | 0.780 | 4 (1 to 8 ) | 4 (1 to 10 ) | -0.14 (-1.55 to 1.29 ) | 0.847 | 4 (1 to 8 ) | 4 (1 to 10 ) | -0.17 (-1.55 to 1.24 ) | 0.813 |
| Lhasa | 4 (1 to 10 ) | 4 (1 to 10 ) | -0.46 (-1.00 to 0.07 ) | 0.092 | 4 (1 to 10 ) | 4 (1 to 11 ) | -0.42 (-0.92 to 0.09 ) | 0.108 | 4 (1 to 10 ) | 4 (1 to 11 ) | -0.44 (-0.96 to 0.08 ) | 0.099 |
| Nagchu | 4 (1 to 8 ) | 3 (1 to 9 ) | -0.43 (-0.79 to -0.07 ) | 0.018 | 4 (1 to 8 ) | 3 (1 to 10 ) | -0.38 (-0.72 to -0.05 ) | 0.026 | 4 (1 to 8 ) | 3 (1 to 9 ) | -0.41 (-0.76 to -0.06 ) | 0.021 |
| Ngari | 4 (1 to 9 ) | 3 (1 to 9 ) | -0.71 (-2.05 to 0.65 ) | 0.307 | 4 (1 to 10 ) | 3 (1 to 9 ) | -0.66 (-2.12 to 0.82 ) | 0.378 | 4 (1 to 10 ) | 3 (1 to 9 ) | -0.69 (-2.17 to 0.81 ) | 0.366 |
| Nyingtri | 4 (1 to 9 ) | 4 (1 to 11 ) | -0.16 (-0.90 to 0.59 ) | 0.678 | 4 (1 to 9 ) | 4 (1 to 11 ) | -0.11 (-0.88 to 0.67 ) | 0.787 | 4 (1 to 9 ) | 4 (1 to 11 ) | -0.13 (-0.89 to 0.63 ) | 0.731 |
| Shannan | 4 (1 to 10 ) | 4 (1 to 11 ) | -0.28 (-0.78 to 0.23 ) | 0.276 | 4 (1 to 11 ) | 4 (1 to 12 ) | -0.23 (-0.74 to 0.28 ) | 0.373 | 4 (1 to 11 ) | 4 (1 to 12 ) | -0.26 (-0.76 to 0.25 ) | 0.320 |
| Shigatse | 4 (1 to 10 ) | 3 (1 to 11 ) | -0.52 (-1.42 to 0.39 ) | 0.264 | 4 (1 to 11 ) | 4 (1 to 11 ) | -0.46 (-1.32 to 0.41 ) | 0.296 | 4 (1 to 10 ) | 4 (1 to 11 ) | -0.49 (-1.37 to 0.40 ) | 0.279 |
| Yunnan |  |  |  |  |  |  |  |  |  |  |  |  |
| Baoshan | 5 (2 to 11 ) | 4 (1 to 13 ) | -0.49 (-1.77 to 0.81 ) | 0.460 | 5 (2 to 11 ) | 5 (1 to 13 ) | -0.45 (-1.72 to 0.85 ) | 0.497 | 5 (2 to 11 ) | 5 (1 to 13 ) | -0.47 (-1.75 to 0.83 ) | 0.477 |
| Chuxiong Yi | 5 (2 to 9 ) | 4 (1 to 12 ) | -0.81 (-1.11 to -0.50 ) | <.001 | 5 (2 to 10 ) | 4 (1 to 12 ) | -0.76 (-1.08 to -0.44 ) | <.001 | 5 (2 to 9 ) | 4 (1 to 12 ) | -0.78 (-1.10 to -0.47 ) | <.001 |
| Dali Bai | 5 (2 to 10 ) | 4 (1 to 12 ) | -0.73 (-1.34 to -0.12 ) | 0.019 | 5 (2 to 10 ) | 4 (1 to 12 ) | -0.69 (-1.29 to -0.08 ) | 0.027 | 5 (2 to 10 ) | 4 (1 to 12 ) | -0.71 (-1.31 to -0.10 ) | 0.022 |
| Dehong Dai and Jingpo | 5 (2 to 12 ) | 5 (1 to 13 ) | -0.42 (-2.12 to 1.32 ) | 0.636 | 5 (2 to 12 ) | 5 (1 to 14 ) | -0.37 (-2.06 to 1.35 ) | 0.669 | 5 (2 to 12 ) | 5 (1 to 13 ) | -0.40 (-2.09 to 1.33 ) | 0.652 |
| Dêqên Tibetan | 5 (2 to 10 ) | 5 (1 to 13 ) | -0.24 (-1.34 to 0.87 ) | 0.671 | 5 (2 to 10 ) | 5 (1 to 13 ) | -0.19 (-1.27 to 0.89 ) | 0.728 | 5 (2 to 10 ) | 5 (1 to 13 ) | -0.22 (-1.30 to 0.88 ) | 0.698 |
| Honghe Hani and Yi | 5 (2 to 8 ) | 4 (1 to 12 ) | -0.58 (-1.43 to 0.28 ) | 0.183 | 5 (2 to 9 ) | 4 (1 to 13 ) | -0.55 (-0.93 to -0.17 ) | 0.005 | 5 (2 to 9 ) | 4 (1 to 13 ) | -0.55 (-1.30 to 0.19 ) | 0.145 |
| Kunming | 4 (2 to 8 ) | 3 (1 to 10 ) | -1.07 (-1.77 to -0.37 ) | 0.003 | 4 (2 to 8 ) | 4 (1 to 11 ) | -1.05 (-1.51 to -0.59 ) | <.001 | 4 (2 to 8 ) | 4 (1 to 10 ) | -1.04 (-1.74 to -0.33 ) | 0.004 |
| Lijiang | 5 (2 to 10 ) | 4 (1 to 12 ) | -0.72 (-1.08 to -0.35 ) | <.001 | 5 (2 to 10 ) | 4 (1 to 12 ) | -0.67 (-1.04 to -0.31 ) | <.001 | 5 (2 to 10 ) | 4 (1 to 12 ) | -0.70 (-1.06 to -0.33 ) | <.001 |
| Lincang | 5 (2 to 10 ) | 5 (1 to 14 ) | -0.26 (-1.62 to 1.12 ) | 0.713 | 5 (2 to 11 ) | 5 (1 to 15 ) | -0.21 (-1.56 to 1.16 ) | 0.761 | 5 (2 to 11 ) | 5 (1 to 15 ) | -0.23 (-1.59 to 1.14 ) | 0.736 |
| Nujiang Lisu | 5 (2 to 11 ) | 5 (1 to 13 ) | -0.37 (-1.50 to 0.78 ) | 0.529 | 5 (2 to 12 ) | 5 (1 to 14 ) | -0.32 (-1.44 to 0.81 ) | 0.579 | 5 (2 to 11 ) | 5 (1 to 14 ) | -0.34 (-1.47 to 0.80 ) | 0.553 |
| Pu'er | 5 (2 to 10 ) | 5 (1 to 16 ) | -0.17 (-1.36 to 1.03 ) | 0.776 | 5 (2 to 11 ) | 5 (1 to 16 ) | -0.13 (-1.31 to 1.07 ) | 0.836 | 5 (2 to 10 ) | 5 (1 to 16 ) | -0.15 (-1.33 to 1.05 ) | 0.805 |
| Qujing | 4 (2 to 7 ) | 4 (1 to 11 ) | -0.77 (-1.50 to -0.04 ) | 0.040 | 4 (2 to 8 ) | 4 (1 to 11 ) | -0.73 (-1.45 to 0.01 ) | 0.053 | 4 (2 to 7 ) | 4 (1 to 11 ) | -0.75 (-1.48 to -0.01 ) | 0.046 |
| Wenshan Zhuang and Miao | 5 (2 to 8 ) | 4 (1 to 11 ) | -0.90 (-2.10 to 0.31 ) | 0.143 | 5 (2 to 8 ) | 4 (1 to 11 ) | -0.86 (-2.05 to 0.35 ) | 0.162 | 5 (2 to 8 ) | 4 (1 to 11 ) | -0.88 (-2.07 to 0.33 ) | 0.152 |
| Xishuangbanna Dai | 5 (2 to 11 ) | 5 (1 to 16 ) | -0.19 (-0.93 to 0.56 ) | 0.619 | 5 (2 to 12 ) | 5 (1 to 16 ) | -0.13 (-0.87 to 0.61 ) | 0.726 | 5 (2 to 12 ) | 5 (1 to 16 ) | -0.16 (-0.90 to 0.58 ) | 0.670 |
| Yuxi | 5 (2 to 9 ) | 4 (1 to 12 ) | -0.89 (-1.36 to -0.41 ) | <.001 | 5 (2 to 9 ) | 4 (1 to 13 ) | -0.84 (-1.32 to -0.36 ) | 0.001 | 5 (2 to 9 ) | 4 (1 to 12 ) | -0.87 (-1.34 to -0.39 ) | <.001 |
| Zhaotong | 4 (2 to 7 ) | 4 (1 to 10 ) | -0.64 (-1.11 to -0.16 ) | 0.009 | 4 (2 to 7 ) | 4 (1 to 11 ) | -0.57 (-1.06 to -0.07 ) | 0.026 | 4 (2 to 7 ) | 4 (1 to 11 ) | -0.60 (-1.08 to -0.12 ) | 0.015 |
| Zhejiang |  |  |  |  |  |  |  |  |  |  |  |  |
| Hangzhou | 3 (1 to 5 ) | 3 (1 to 7 ) | -0.36 (-0.69 to -0.03 ) | 0.032 | 3 (1 to 5 ) | 3 (1 to 7 ) | -0.31 (-0.61 to -0.01 ) | 0.045 | 3 (1 to 5 ) | 3 (1 to 7 ) | -0.34 (-0.65 to -0.02 ) | 0.037 |
| Huzhou | 3 (1 to 5 ) | 3 (1 to 7 ) | -0.15 (-0.41 to 0.10 ) | 0.235 | 3 (1 to 5 ) | 3 (1 to 8 ) | -0.10 (-0.34 to 0.14 ) | 0.425 | 3 (1 to 5 ) | 3 (1 to 7 ) | -0.13 (-0.37 to 0.12 ) | 0.312 |
| Jiaxing | 3 (1 to 5 ) | 3 (1 to 7 ) | -0.44 (-0.95 to 0.06 ) | 0.085 | 3 (1 to 5 ) | 3 (1 to 8 ) | -0.39 (-0.90 to 0.12 ) | 0.130 | 3 (1 to 5 ) | 3 (1 to 7 ) | -0.42 (-0.92 to 0.09 ) | 0.104 |
| Jinhua | 3 (1 to 5 ) | 3 (1 to 8 ) | -0.41 (-0.82 to -0.01 ) | 0.046 | 3 (1 to 6 ) | 3 (1 to 8 ) | -0.37 (-0.76 to 0.03 ) | 0.071 | 3 (1 to 6 ) | 3 (1 to 8 ) | -0.39 (-0.79 to 0.01 ) | 0.058 |
| Lishui | 3 (1 to 6 ) | 3 (1 to 8 ) | -0.42 (-0.87 to 0.03 ) | 0.069 | 3 (1 to 6 ) | 3 (1 to 9 ) | -0.36 (-0.80 to 0.07 ) | 0.102 | 3 (1 to 6 ) | 3 (1 to 8 ) | -0.39 (-0.83 to 0.05 ) | 0.083 |
| Ningbo | 3 (1 to 6 ) | 3 (1 to 8 ) | -0.50 (-0.71 to -0.28 ) | <.001 | 3 (1 to 6 ) | 3 (1 to 8 ) | -0.45 (-0.66 to -0.24 ) | <.001 | 3 (1 to 6 ) | 3 (1 to 8 ) | -0.47 (-0.69 to -0.26 ) | <.001 |
| Quzhou | 3 (1 to 6 ) | 3 (1 to 8 ) | -0.27 (-0.50 to -0.05 ) | 0.018 | 3 (1 to 6 ) | 3 (1 to 9 ) | -0.23 (-0.45 to 0.00 ) | 0.052 | 3 (1 to 6 ) | 3 (1 to 8 ) | -0.25 (-0.48 to -0.02 ) | 0.030 |
| Shaoxing | 3 (1 to 5 ) | 3 (1 to 8 ) | -0.33 (-0.71 to 0.05 ) | 0.092 | 3 (1 to 5 ) | 3 (1 to 8 ) | -0.30 (-0.65 to 0.05 ) | 0.096 | 3 (1 to 5 ) | 3 (1 to 8 ) | -0.31 (-0.70 to 0.09 ) | 0.127 |
| Taizhou | 3 (1 to 6 ) | 3 (1 to 8 ) | -0.43 (-0.83 to -0.03 ) | 0.034 | 3 (1 to 6 ) | 3 (1 to 8 ) | -0.38 (-0.78 to 0.03 ) | 0.069 | 3 (1 to 6 ) | 3 (1 to 8 ) | -0.40 (-0.80 to 0.0 ) | 0.048 |
| Wenzhou | 3 (1 to 6 ) | 3 (1 to 7 ) | -0.52 (-1.20 to 0.16 ) | 0.133 | 3 (1 to 6 ) | 3 (1 to 8 ) | -0.47 (-1.12 to 0.19 ) | 0.166 | 3 (1 to 6 ) | 3 (1 to 8 ) | -0.49 (-1.16 to 0.18 ) | 0.148 |
| Zhoushan | 3 (1 to 6 ) | 3 (1 to 8 ) | -0.43 (-1.10 to 0.24 ) | 0.210 | 3 (1 to 6 ) | 3 (1 to 9 ) | -0.38 (-1.06 to 0.30 ) | 0.269 | 3 (1 to 6 ) | 3 (1 to 8 ) | -0.41 (-1.08 to 0.27 ) | 0.237 |
| Hong Kong |  |  |  |  |  |  |  |  |  |  |  |  |
| Central and Western | 2 (1 to 4 ) | 2 (0 to 6 ) | 0.35 (-0.74 to 1.46 ) | 0.527 | 2 (1 to 4 ) | 2 (0 to 6 ) | 0.32 (-0.28 to 0.91 ) | 0.296 | 2 (1 to 4 ) | 2 (0 to 6 ) | 0.29 (-0.31 to 0.90 ) | 0.337 |
| Eastern | 2 (1 to 3 ) | 2 (0 to 5 ) | -0.26 (-0.94 to 0.43 ) | 0.457 | 2 (1 to 3 ) | 2 (0 to 6 ) | -0.23 (-0.94 to 0.48 ) | 0.526 | 2 (1 to 3 ) | 2 (0 to 6 ) | -0.24 (-0.92 to 0.45 ) | 0.496 |
| Islands | 2 (1 to 4 ) | 2 (0 to 6 ) | 0.25 (-0.23 to 0.72 ) | 0.307 | 2 (1 to 4 ) | 2 (0 to 7 ) | 0.25 (-0.10 to 0.60 ) | 0.165 | 2 (1 to 4 ) | 2 (0 to 7 ) | 0.27 (-0.18 to 0.73 ) | 0.236 |
| Kowloon City | 1 (1 to 2 ) | 1 (0 to 4 ) | -0.33 (-0.59 to -0.08 ) | 0.011 | 1 (1 to 2 ) | 1 (0 to 4 ) | -0.29 (-0.55 to -0.03 ) | 0.028 | 1 (1 to 2 ) | 1 (0 to 4 ) | -0.31 (-0.57 to -0.05 ) | 0.017 |
| Kwai Tsing | 2 (1 to 3 ) | 2 (0 to 5 ) | 0.78 (0.33 to 1.23 ) | 0.001 | 2 (1 to 3 ) | 2 (0 to 6 ) | 0.83 (0.40 to 1.27 ) | <.001 | 2 (1 to 3 ) | 2 (0 to 5 ) | 0.81 (0.37 to 1.25 ) | <.001 |
| Kwun Tong | 1 (1 to 2 ) | 1 (0 to 4 ) | -0.78 (-1.51 to -0.05 ) | 0.036 | 1 (1 to 2 ) | 1 (0 to 4 ) | -0.74 (-1.47 to 0.00 ) | 0.049 | 1 (1 to 2 ) | 1 (0 to 4 ) | -0.76 (-1.49 to -0.03 ) | 0.042 |
| North | 2 (1 to 4 ) | 2 (0 to 6 ) | -0.46 (-1.05 to 0.14 ) | 0.130 | 2 (1 to 4 ) | 2 (0 to 6 ) | -0.39 (-0.96 to 0.19 ) | 0.189 | 2 (1 to 4 ) | 2 (0 to 6 ) | -0.43 (-1.01 to 0.16 ) | 0.155 |
| Sai Kung | 2 (1 to 3 ) | 2 (0 to 5 ) | -0.47 (-0.86 to -0.08 ) | 0.019 | 2 (1 to 3 ) | 2 (0 to 5 ) | -0.45 (-0.89 to -0.01 ) | 0.046 | 2 (1 to 3 ) | 2 (0 to 5 ) | -0.45 (-0.85 to -0.05 ) | 0.026 |
| Sha Tin | 2 (1 to 3 ) | 2 (0 to 6 ) | -0.06 (-0.64 to 0.53 ) | 0.843 | 2 (1 to 4 ) | 2 (0 to 6 ) | 0.01 (-0.52 to 0.54 ) | 0.973 | 2 (1 to 3 ) | 2 (0 to 6 ) | -0.03 (-0.58 to 0.54 ) | 0.927 |
| Sham Shui Po | 2 (1 to 3 ) | 2 (0 to 5 ) | 0.42 (-0.56 to 1.42 ) | 0.404 | 2 (1 to 3 ) | 2 (0 to 5 ) | 0.47 (-0.45 to 1.39 ) | 0.321 | 2 (1 to 3 ) | 2 (0 to 5 ) | 0.44 (-0.51 to 1.40 ) | 0.364 |
| Southern | 2 (1 to 3 ) | 2 (0 to 6 ) | 0.21 (-0.41 to 0.83 ) | 0.507 | 2 (1 to 4 ) | 2 (0 to 6 ) | 0.24 (-0.50 to 0.99 ) | 0.525 | 2 (1 to 3 ) | 2 (0 to 6 ) | 0.23 (-0.38 to 0.85 ) | 0.457 |
| Tai Po | 2 (1 to 4 ) | 2 (0 to 6 ) | -0.20 (-0.76 to 0.37 ) | 0.491 | 2 (1 to 4 ) | 2 (0 to 7 ) | -0.15 (-0.72 to 0.41 ) | 0.594 | 2 (1 to 4 ) | 2 (0 to 7 ) | -0.18 (-0.74 to 0.39 ) | 0.539 |
| Tsuen Wan | 2 (1 to 3 ) | 2 (0 to 6 ) | 0.47 (-0.18 to 1.12 ) | 0.154 | 2 (1 to 3 ) | 2 (0 to 7 ) | 0.53 (-0.11 to 1.19 ) | 0.106 | 2 (1 to 3 ) | 2 (0 to 6 ) | 0.50 (-0.14 to 1.15 ) | 0.129 |
| Tuen Mun | 2 (1 to 3 ) | 2 (0 to 5 ) | 0.03 (-0.23 to 0.30 ) | 0.805 | 2 (1 to 3 ) | 2 (0 to 6 ) | 0.08 (-0.20 to 0.36 ) | 0.589 | 2 (1 to 3 ) | 2 (0 to 6 ) | 0.05 (-0.22 to 0.33 ) | 0.694 |
| Wan Chai | 2 (1 to 3 ) | 2 (0 to 5 ) | -0.36 (-1.01 to 0.30 ) | 0.286 | 2 (1 to 4 ) | 2 (0 to 6 ) | -0.31 (-0.93 to 0.31 ) | 0.326 | 2 (1 to 3 ) | 2 (0 to 6 ) | -0.33 (-0.97 to 0.30 ) | 0.304 |
| Wong Tai Sin | 2 (1 to 3 ) | 2 (0 to 5 ) | -0.04 (-1.08 to 1.02 ) | 0.947 | 2 (1 to 3 ) | 2 (0 to 5 ) | 0.03 (-0.97 to 1.05 ) | 0.950 | 2 (1 to 3 ) | 2 (0 to 5 ) | 0.0 (-1.03 to 1.03 ) | 0.996 |
| Yau Tsim Mong | 1 (1 to 2 ) | 1 (0 to 4 ) | 0.51 (0.19 to 0.84 ) | 0.002 | 1 (1 to 3 ) | 1 (0 to 4 ) | 0.56 (0.25 to 0.88 ) | <.001 | 1 (1 to 2 ) | 1 (0 to 4 ) | 0.54 (0.22 to 0.86 ) | 0.001 |
| Yuen Long | 2 (1 to 3 ) | 2 (0 to 5 ) | -0.44 (-0.92 to 0.04 ) | 0.072 | 2 (1 to 4 ) | 2 (0 to 6 ) | -0.40 (-0.86 to 0.07 ) | 0.095 | 2 (1 to 3 ) | 2 (0 to 5 ) | -0.42 (-0.89 to 0.05 ) | 0.082 |
| Macao |  |  |  |  |  |  |  |  |  |  |  |  |
| Ilhas | 3 (2 to 6 ) | 3 (1 to 10 ) | -0.35 (-0.96 to 0.26 ) | 0.260 | 4 (2 to 7 ) | 3 (1 to 10 ) | -0.31 (-0.93 to 0.32 ) | 0.337 | 4 (2 to 6 ) | 3 (1 to 10 ) | -0.33 (-0.94 to 0.29 ) | 0.296 |
| Macau | 3 (1 to 6 ) | 3 (1 to 9 ) | -0.11 (-0.36 to 0.14 ) | 0.405 | 3 (2 to 6 ) | 3 (1 to 10 ) | -0.18 (-0.53 to 0.17 ) | 0.316 | 3 (1 to 6 ) | 3 (1 to 9 ) | -0.20 (-0.55 to 0.15 ) | 0.267 |
|  |  |  |  |  |  |  |  |  |  |  |  |  |
|  | **Underweight** | | | | | | | | | | | |
| Anhui |  |  |  |  |  |  |  |  |  |  |  |  |
| Anqing | 11 (6 to 18 ) | 6 (2 to 16 ) | -3.01 (-4.09 to -1.92 ) | <.001 | 10 (5 to 16 ) | 5 (1 to 14 ) | -3.08 (-4.16 to -1.98 ) | <.001 | 10 (5 to 17 ) | 6 (2 to 15 ) | -3.06 (-4.22 to -1.89 ) | <.001 |
| Bengbu | 6 (4 to 11 ) | 4 (1 to 13 ) | -2.32 (-3.53 to -1.09 ) | <.001 | 6 (3 to 10 ) | 4 (1 to 12 ) | -2.31 (-3.15 to -1.45 ) | <.001 | 6 (4 to 10 ) | 4 (1 to 12 ) | -2.25 (-3.13 to -1.37 ) | <.001 |
| Bozhou | 6 (3 to 10 ) | 3 (1 to 9 ) | -3.19 (-5.10 to -1.25 ) | 0.001 | 5 (3 to 9 ) | 3 (1 to 8 ) | -3.28 (-5.37 to -1.15 ) | 0.003 | 6 (3 to 9 ) | 3 (1 to 9 ) | -3.22 (-5.0 to -1.41 ) | 0.001 |
| Chaohu | 9 (5 to 14 ) | 6 (2 to 18 ) | -1.92 (-2.66 to -1.18 ) | <.001 | 8 (4 to 13 ) | 5 (1 to 17 ) | -2.05 (-2.85 to -1.23 ) | <.001 | 8 (5 to 14 ) | 6 (1 to 18 ) | -1.95 (-2.84 to -1.05 ) | <.001 |
| Chizhou | 12 (6 to 20 ) | 7 (2 to 18 ) | -2.68 (-3.80 to -1.55 ) | <.001 | 11 (6 to 18 ) | 6 (2 to 17 ) | -2.76 (-4.31 to -1.19 ) | 0.001 | 11 (6 to 19 ) | 7 (2 to 18 ) | -2.65 (-4.24 to -1.04 ) | 0.001 |
| Chuzhou | 7 (4 to 12 ) | 6 (2 to 18 ) | -0.89 (-1.93 to 0.17 ) | 0.099 | 7 (4 to 11 ) | 5 (2 to 16 ) | -1.00 (-1.97 to -0.02 ) | 0.045 | 7 (4 to 11 ) | 6 (2 to 17 ) | -0.96 (-1.90 to 0.0 ) | 0.049 |
| Fuyang | 6 (3 to 11 ) | 3 (1 to 9 ) | -3.66 (-4.81 to -2.50 ) | <.001 | 6 (3 to 10 ) | 3 (1 to 8 ) | -3.76 (-4.85 to -2.65 ) | <.001 | 6 (3 to 11 ) | 3 (1 to 9 ) | -3.70 (-4.82 to -2.57 ) | <.001 |
| Hefei | 6 (4 to 11 ) | 4 (1 to 12 ) | -2.59 (-3.68 to -1.49 ) | <.001 | 6 (3 to 10 ) | 4 (1 to 10 ) | -2.66 (-3.97 to -1.33 ) | <.001 | 6 (4 to 10 ) | 4 (1 to 11 ) | -2.63 (-3.43 to -1.83 ) | <.001 |
| Huaibei | 5 (3 to 9 ) | 4 (1 to 10 ) | -2.25 (-4.26 to -0.20 ) | 0.031 | 5 (3 to 8 ) | 3 (1 to 9 ) | -2.49 (-4.43 to -0.50 ) | 0.014 | 5 (3 to 9 ) | 3 (1 to 10 ) | -2.31 (-4.30 to -0.29 ) | 0.025 |
| Huainan | 7 (4 to 11 ) | 4 (1 to 13 ) | -2.58 (-3.62 to -1.52 ) | <.001 | 6 (4 to 10 ) | 4 (1 to 11 ) | -2.77 (-3.38 to -2.16 ) | <.001 | 7 (4 to 11 ) | 4 (1 to 12 ) | -2.73 (-3.35 to -2.10 ) | <.001 |
| Huangshan | 11 (6 to 19 ) | 8 (2 to 22 ) | -1.92 (-3.09 to -0.74 ) | 0.001 | 10 (5 to 18 ) | 7 (2 to 20 ) | -1.89 (-3.45 to -0.30 ) | 0.020 | 11 (6 to 19 ) | 7 (2 to 21 ) | -1.93 (-3.03 to -0.82 ) | 0.001 |
| Lu'an | 9 (5 to 16 ) | 6 (2 to 17 ) | -2.47 (-3.25 to -1.69 ) | <.001 | 9 (5 to 15 ) | 5 (1 to 15 ) | -2.53 (-3.54 to -1.51 ) | <.001 | 9 (5 to 16 ) | 6 (2 to 16 ) | -2.50 (-3.37 to -1.62 ) | <.001 |
| Ma'anshan | 7 (4 to 11 ) | 5 (1 to 16 ) | -1.27 (-2.00 to -0.53 ) | 0.001 | 6 (3 to 10 ) | 5 (1 to 15 ) | -1.40 (-2.09 to -0.70 ) | <.001 | 6 (3 to 11 ) | 5 (1 to 16 ) | -1.30 (-2.04 to -0.55 ) | 0.001 |
| Suzhou | 6 (4 to 9 ) | 4 (1 to 12 ) | -2.19 (-4.04 to -0.31 ) | 0.023 | 6 (3 to 9 ) | 4 (1 to 11 ) | -2.28 (-3.90 to -0.63 ) | 0.007 | 6 (4 to 9 ) | 4 (1 to 11 ) | -2.08 (-3.94 to -0.18 ) | 0.032 |
| Tongling | 9 (5 to 15 ) | 5 (1 to 16 ) | -2.48 (-3.89 to -1.05 ) | 0.001 | 8 (4 to 14 ) | 5 (1 to 14 ) | -2.95 (-4.41 to -1.46 ) | <.001 | 9 (5 to 14 ) | 5 (1 to 15 ) | -2.46 (-4.14 to -0.75 ) | 0.005 |
| Wuhu | 7 (4 to 13 ) | 5 (1 to 16 ) | -1.84 (-3.14 to -0.53 ) | 0.006 | 7 (4 to 12 ) | 5 (1 to 14 ) | -2.12 (-2.97 to -1.26 ) | <.001 | 7 (4 to 12 ) | 5 (1 to 15 ) | -1.82 (-3.08 to -0.53 ) | 0.006 |
| Xuancheng | 9 (5 to 15 ) | 7 (2 to 22 ) | -1.06 (-1.91 to -0.19 ) | 0.017 | 8 (5 to 14 ) | 7 (2 to 20 ) | -1.32 (-2.44 to -0.19 ) | 0.023 | 9 (5 to 15 ) | 7 (2 to 21 ) | -1.03 (-2.14 to 0.09 ) | 0.070 |
| Beijing |  |  |  |  |  |  |  |  |  |  |  |  |
| Beijing | 4 (2 to 6 ) | 3 (1 to 8 ) | -1.24 (-1.30 to -1.17 ) | <.001 | 3 (2 to 6 ) | 3 (1 to 7 ) | -1.36 (-1.45 to -1.28 ) | <.001 | 3 (02 to 6 ) | 3 (1 to 8 ) | -1.30 (-1.40 to -1.20 ) | <.001 |
| Chongqing |  |  |  |  |  |  |  |  |  |  |  |  |
| Chongqing | 7 (4 to 11 ) | 4 (1 to 11 ) | -2.82 (-2.88 to -2.77 ) | <.001 | 7 (4 to 10 ) | 4 (1 to 10 ) | -2.93 (-3.04 to -2.83 ) | <.001 | 7 (4 to 10 ) | 4 (1 to 11 ) | -2.87 (-3.0 to -2.74 ) | <.001 |
| Fujian |  |  |  |  |  |  |  |  |  |  |  |  |
| Fuzhou | 5 (2 to 10 ) | 3 (1 to 8 ) | -2.74 (-3.74 to -1.73 ) | <.001 | 4 (2 to 9 ) | 2 (1 to 7 ) | -2.68 (-3.45 to -1.91 ) | <.001 | 4 (02 to 10 ) | 3 (1 to 8 ) | -2.76 (-3.38 to -2.14 ) | <.001 |
| Longyan | 9 (4 to 17 ) | 6 (1 to 17 ) | -1.95 (-2.84 to -1.06 ) | <.001 | 8 (4 to 16 ) | 5 (1 to 15 ) | -2.02 (-2.91 to -1.12 ) | <.001 | 9 (4 to 16 ) | 6 (1 to 16 ) | -1.98 (-2.87 to -1.09 ) | <.001 |
| Nanping | 8 (4 to 16 ) | 7 (2 to 20 ) | -1.35 (-2.36 to -0.32 ) | 0.010 | 8 (3 to 14 ) | 6 (1 to 18 ) | -1.42 (-2.47 to -0.36 ) | 0.009 | 8 (4 to 15 ) | 6 (2 to 19 ) | -1.39 (-2.40 to -0.36 ) | 0.008 |
| Ningde | 7 (3 to 14 ) | 5 (1 to 15 ) | -2.08 (-2.60 to -1.56 ) | <.001 | 6 (2 to 13 ) | 4 (1 to 14 ) | -2.17 (-2.70 to -1.64 ) | <.001 | 7 (3 to 14 ) | 5 (1 to 14 ) | -2.12 (-2.65 to -1.59 ) | <.001 |
| Putian | 5 (2 to 10 ) | 3 (1 to 8 ) | -2.88 (-4.36 to -1.38 ) | <.001 | 4 (2 to 10 ) | 2 (0 to 7 ) | -2.73 (-4.36 to -1.07 ) | 0.001 | 5 (02 to 10 ) | 3 (1 to 7 ) | -2.82 (-4.27 to -1.34 ) | <.001 |
| Quanzhou | 5 (2 to 10 ) | 3 (1 to 8 ) | -2.44 (-3.03 to -1.84 ) | <.001 | 4 (2 to 9 ) | 3 (1 to 7 ) | -2.27 (-3.39 to -1.14 ) | <.001 | 4 (02 to 10 ) | 3 (1 to 8 ) | -2.37 (-3.05 to -1.67 ) | <.001 |
| Sanming | 9 (4 to 16 ) | 7 (1 to 18 ) | -1.31 (-2.32 to -0.28 ) | 0.013 | 8 (4 to 15 ) | 6 (1 to 16 ) | -1.37 (-2.32 to -0.41 ) | 0.005 | 8 (4 to 16 ) | 6 (1 to 17 ) | -1.33 (-2.36 to -0.30 ) | 0.012 |
| Xiamen | 3 (1 to 7 ) | 2 (0 to 4 ) | -3.37 (-4.41 to -2.31 ) | <.001 | 3 (1 to 6 ) | 1 (0 to 4 ) | -3.29 (-5.10 to -1.45 ) | 0.001 | 3 (01 to 7 ) | 1 (0 to 4 ) | -3.39 (-4.67 to -2.08 ) | <.001 |
| Zhangzhou | 6 (2 to 13 ) | 4 (1 to 12 ) | -2.05 (-2.82 to -1.27 ) | <.001 | 6 (2 to 12 ) | 4 (1 to 11 ) | -2.10 (-2.87 to -1.32 ) | <.001 | 6 (02 to 13 ) | 4 (1 to 12 ) | -2.07 (-2.84 to -1.29 ) | <.001 |
| Gansu |  |  |  |  |  |  |  |  |  |  |  |  |
| Baiyin | 8 (3 to 18 ) | 5 (1 to 13 ) | -3.02 (-3.67 to -2.36 ) | <.001 | 8 (3 to 17 ) | 4 (1 to 11 ) | -3.06 (-3.70 to -2.40 ) | <.001 | 8 (3 to 17 ) | 5 (1 to 12 ) | -3.05 (-3.66 to -2.44 ) | <.001 |
| Dingxi | 10 (4 to 19 ) | 5 (1 to 14 ) | -3.33 (-4.17 to -2.47 ) | <.001 | 9 (4 to 18 ) | 5 (1 to 13 ) | -3.43 (-3.92 to -2.95 ) | <.001 | 10 (4 to 18 ) | 5 (1 to 14 ) | -3.38 (-4.28 to -2.47 ) | <.001 |
| Gannan Tibetan | 15 (7 to 26 ) | 11 (3 to 27 ) | -1.98 (-3.23 to -0.72 ) | 0.002 | 14 (6 to 24 ) | 10 (2 to 25 ) | -2.06 (-3.27 to -0.84 ) | 0.001 | 14 (6 to 25 ) | 10 (3 to 26 ) | -2.02 (-3.25 to -0.78 ) | 0.002 |
| Jiayuguan | 11 (5 to 20 ) | 6 (1 to 16 ) | -3.21 (-5.96 to -0.39 ) | 0.026 | 10 (4 to 19 ) | 6 (1 to 15 ) | -3.30 (-6.00 to -0.52 ) | 0.020 | 10 (4 to 20 ) | 6 (1 to 16 ) | -3.25 (-5.98 to -0.45 ) | 0.023 |
| Jinchang | 12 (5 to 22 ) | 7 (2 to 18 ) | -3.03 (-4.84 to -1.20 ) | 0.001 | 11 (5 to 20 ) | 6 (1 to 16 ) | -3.02 (-5.07 to -0.93 ) | 0.005 | 11 (5 to 21 ) | 7 (2 to 17 ) | -2.95 (-5.0 to -0.86 ) | 0.006 |
| Jiuquan | 12 (5 to 20 ) | 9 (2 to 22 ) | -1.86 (-4.13 to 0.46 ) | 0.115 | 11 (5 to 18 ) | 8 (2 to 20 ) | -1.95 (-4.18 to 0.32 ) | 0.092 | 11 (5 to 19 ) | 8 (2 to 21 ) | -1.90 (-4.15 to 0.40 ) | 0.104 |
| Lanzhou | 7 (3 to 15 ) | 3 (1 to 9 ) | -3.86 (-4.44 to -3.27 ) | <.001 | 7 (3 to 14 ) | 3 (1 to 8 ) | -4.13 (-5.14 to -3.11 ) | <.001 | 7 (3 to 15 ) | 3 (1 to 9 ) | -4.09 (-5.13 to -3.04 ) | <.001 |
| Linxia Hui | 11 (4 to 22 ) | 6 (1 to 15 ) | -3.77 (-4.92 to -2.61 ) | <.001 | 10 (4 to 20 ) | 5 (1 to 13 ) | -3.81 (-4.67 to -2.94 ) | <.001 | 11 (4 to 21 ) | 5 (1 to 14 ) | -3.77 (-4.81 to -2.73 ) | <.001 |
| Longnan | 9 (4 to 17 ) | 6 (2 to 16 ) | -2.17 (-3.82 to -0.49 ) | 0.011 | 9 (4 to 15 ) | 5 (1 to 14 ) | -2.23 (-4.60 to 0.20 ) | 0.072 | 9 (4 to 16 ) | 6 (1 to 15 ) | -2.22 (-3.90 to -0.50 ) | 0.011 |
| Pingliang | 7 (3 to 15 ) | 4 (1 to 12 ) | -2.76 (-3.62 to -1.90 ) | <.001 | 7 (3 to 13 ) | 4 (1 to 11 ) | -2.89 (-4.19 to -1.58 ) | <.001 | 7 (3 to 14 ) | 4 (1 to 12 ) | -2.80 (-3.67 to -1.91 ) | <.001 |
| Qingyang | 6 (3 to 12 ) | 4 (1 to 12 ) | -1.72 (-3.58 to 0.18 ) | 0.076 | 6 (2 to 11 ) | 4 (1 to 11 ) | -1.96 (-3.58 to -0.31 ) | 0.020 | 6 (3 to 11 ) | 4 (1 to 11 ) | -1.75 (-3.63 to 0.16 ) | 0.073 |
| Tianshui | 9 (4 to 17 ) | 5 (1 to 12 ) | -3.21 (-4.50 to -1.90 ) | <.001 | 8 (3 to 15 ) | 4 (1 to 11 ) | -3.32 (-4.68 to -1.94 ) | <.001 | 9 (4 to 16 ) | 5 (1 to 11 ) | -3.26 (-4.58 to -1.92 ) | <.001 |
| Wuwei | 11 (5 to 21 ) | 6 (2 to 17 ) | -2.98 (-4.71 to -1.21 ) | 0.001 | 10 (4 to 19 ) | 6 (1 to 16 ) | -3.06 (-4.74 to -1.35 ) | 0.001 | 10 (4 to 20 ) | 6 (2 to 16 ) | -3.01 (-4.72 to -1.27 ) | 0.001 |
| Zhangye | 14 (6 to 23 ) | 9 (2 to 23 ) | -2.78 (-4.99 to -0.52 ) | 0.016 | 13 (5 to 21 ) | 8 (2 to 21 ) | -2.86 (-5.00 to -0.67 ) | 0.011 | 13 (6 to 22 ) | 8 (2 to 22 ) | -2.82 (-5.0 to -0.59 ) | 0.014 |
| Guangdong |  |  |  |  |  |  |  |  |  |  |  |  |
| Chaozhou | 5 (2 to 10 ) | 2 (0 to 7 ) | -4.87 (-8.37 to -1.23 ) | 0.009 | 5 (2 to 10 ) | 2 (0 to 6 ) | -4.73 (-7.25 to -2.14 ) | <.001 | 5 (02 to 10 ) | 2 (0 to 6 ) | -4.81 (-7.89 to -1.63 ) | 0.003 |
| Dongguan | 3 (2 to 6 ) | 2 (0 to 6 ) | -2.14 (-3.40 to -0.86 ) | 0.001 | 3 (2 to 6 ) | 2 (0 to 5 ) | -2.24 (-3.30 to -1.17 ) | <.001 | 3 (02 to 6 ) | 2 (0 to 6 ) | -2.17 (-3.23 to -1.11 ) | <.001 |
| Foshan | 4 (2 to 7 ) | 2 (0 to 6 ) | -2.55 (-3.50 to -1.60 ) | <.001 | 4 (2 to 6 ) | 2 (0 to 6 ) | -2.66 (-3.60 to -1.72 ) | <.001 | 4 (02 to 6 ) | 2 (0 to 6 ) | -2.59 (-3.53 to -1.65 ) | <.001 |
| Guangzhou | 3 (2 to 6 ) | 2 (0 to 5 ) | -2.47 (-3.16 to -1.77 ) | <.001 | 3 (2 to 6 ) | 2 (0 to 5 ) | -2.55 (-3.21 to -1.88 ) | <.001 | 3 (02 to 6 ) | 2 (0 to 5 ) | -2.52 (-3.20 to -1.84 ) | <.001 |
| Heyuan | 8 (4 to 14 ) | 4 (1 to 11 ) | -3.46 (-4.50 to -2.41 ) | <.001 | 7 (4 to 13 ) | 4 (1 to 10 ) | -3.67 (-5.53 to -1.78 ) | <.001 | 7 (4 to 14 ) | 4 (1 to 10 ) | -3.74 (-6.05 to -1.37 ) | 0.002 |
| Huizhou | 5 (3 to 10 ) | 3 (1 to 8 ) | -2.58 (-3.48 to -1.67 ) | <.001 | 5 (2 to 9 ) | 3 (1 to 8 ) | -2.62 (-3.37 to -1.87 ) | <.001 | 5 (02 to 9 ) | 3 (1 to 8 ) | -2.63 (-3.28 to -1.96 ) | <.001 |
| Jiangmen | 5 (3 to 8 ) | 3 (1 to 10 ) | -1.58 (-5.61 to 2.62 ) | 0.455 | 4 (3 to 7 ) | 3 (1 to 9 ) | -1.82 (-5.99 to 2.54 ) | 0.408 | 5 (3 to 8 ) | 3 (1 to 10 ) | -1.75 (-5.96 to 2.64 ) | 0.429 |
| Jieyang | 5 (2 to 11 ) | 2 (1 to 7 ) | -4.53 (-6.99 to -2.00 ) | 0.001 | 5 (2 to 11 ) | 2 (0 to 6 ) | -4.40 (-6.05 to -2.72 ) | <.001 | 5 (02 to 11 ) | 2 (0 to 7 ) | -4.47 (-6.58 to -2.32 ) | <.001 |
| Maoming | 8 (5 to 11 ) | 7 (2 to 19 ) | -0.93 (-1.78 to -0.06 ) | 0.036 | 7 (5 to 10 ) | 6 (1 to 17 ) | -1.03 (-1.85 to -0.21 ) | 0.014 | 8 (5 to 10 ) | 6 (2 to 18 ) | -0.97 (-1.81 to -0.13 ) | 0.024 |
| Meizhou | 7 (3 to 14 ) | 3 (1 to 10 ) | -4.57 (-7.76 to -1.28 ) | 0.007 | 7 (3 to 13 ) | 3 (1 to 9 ) | -4.44 (-6.65 to -2.18 ) | <.001 | 7 (3 to 14 ) | 3 (1 to 9 ) | -4.52 (-7.28 to -1.68 ) | 0.002 |
| Qingyuan | 8 (5 to 13 ) | 5 (1 to 12 ) | -2.91 (-4.16 to -1.64 ) | <.001 | 8 (5 to 12 ) | 4 (1 to 11 ) | -2.97 (-4.20 to -1.73 ) | <.001 | 8 (5 to 12 ) | 5 (1 to 12 ) | -2.94 (-4.17 to -1.69 ) | <.001 |
| Shantou | 4 (1 to 8 ) | 1 (0 to 4 ) | -5.13 (-8.10 to -2.06 ) | 0.001 | 3 (1 to 7 ) | 1 (0 to 4 ) | -5.00 (-7.12 to -2.82 ) | <.001 | 3 (01 to 8 ) | 1 (0 to 4 ) | -5.07 (-7.68 to -2.40 ) | <.001 |
| Shanwei | 5 (2 to 10 ) | 2 (1 to 7 ) | -3.31 (-5.45 to -1.12 ) | 0.003 | 4 (2 to 9 ) | 2 (0 to 6 ) | -3.58 (-5.02 to -2.12 ) | <.001 | 5 (02 to 9 ) | 2 (0 to 6 ) | -3.36 (-5.50 to -1.16 ) | 0.003 |
| Shaoguan | 8 (5 to 13 ) | 5 (1 to 12 ) | -2.94 (-5.77 to -0.02 ) | 0.049 | 8 (4 to 12 ) | 4 (1 to 11 ) | -3.11 (-5.28 to -0.90 ) | 0.006 | 8 (5 to 13 ) | 4 (1 to 11 ) | -2.96 (-6.19 to 0.38 ) | 0.082 |
| Shenzhen | 3 (1 to 6 ) | 2 (0 to 5 ) | -2.32 (-3.36 to -1.25 ) | <.001 | 3 (1 to 5 ) | 2 (0 to 4 ) | -2.26 (-3.99 to -0.50 ) | 0.012 | 3 (01 to 6 ) | 2 (0 to 5 ) | -2.27 (-3.56 to -0.96 ) | 0.001 |
| Yangjiang | 8 (5 to 12 ) | 7 (1 to 18 ) | -0.48 (-2.81 to 1.91 ) | 0.691 | 7 (4 to 11 ) | 6 (1 to 16 ) | -0.67 (-2.75 to 1.45 ) | 0.532 | 7 (5 to 11 ) | 6 (1 to 17 ) | -0.62 (-2.69 to 1.50 ) | 0.566 |
| Yunfu | 8 (5 to 12 ) | 6 (1 to 15 ) | -1.86 (-4.33 to 0.68 ) | 0.149 | 7 (5 to 11 ) | 5 (1 to 13 ) | -1.96 (-4.37 to 0.52 ) | 0.120 | 8 (5 to 11 ) | 5 (1 to 14 ) | -1.90 (-4.35 to 0.61 ) | 0.136 |
| Zhanjiang | 6 (5 to 9 ) | 6 (1 to 18 ) | -0.35 (-2.17 to 1.50 ) | 0.706 | 6 (4 to 8 ) | 6 (1 to 16 ) | -0.47 (-2.21 to 1.30 ) | 0.601 | 6 (4 to 8 ) | 6 (1 to 17 ) | -0.40 (-2.19 to 1.41 ) | 0.660 |
| Zhaoqing | 8 (5 to 12 ) | 5 (1 to 13 ) | -2.49 (-3.39 to -1.59 ) | <.001 | 7 (5 to 11 ) | 4 (1 to 11 ) | -2.57 (-3.33 to -1.81 ) | <.001 | 8 (5 to 11 ) | 5 (1 to 12 ) | -2.51 (-3.38 to -1.64 ) | <.001 |
| Zhongshan | 4 (2 to 6 ) | 2 (0 to 7 ) | -1.88 (-5.28 to 1.65 ) | 0.292 | 3 (2 to 6 ) | 2 (0 to 6 ) | -1.99 (-4.29 to 0.37 ) | 0.097 | 3 (02 to 6 ) | 2 (0 to 7 ) | -1.95 (-3.80 to -0.06 ) | 0.043 |
| Zhuhai | 4 (2 to 7 ) | 3 (1 to 8 ) | -1.05 (-2.08 to -0.01 ) | 0.047 | 3 (2 to 6 ) | 3 (0 to 7 ) | -1.15 (-2.20 to -0.08 ) | 0.035 | 4 (02 to 6 ) | 3 (1 to 8 ) | -1.09 (-2.13 to -0.05 ) | 0.041 |
| Guangxi |  |  |  |  |  |  |  |  |  |  |  |  |
| Baise | 14 (10 to 18 ) | 7 (2 to 20 ) | -3.31 (-4.77 to -1.82 ) | <.001 | 13 (9 to 17 ) | 7 (2 to 18 ) | -3.39 (-4.92 to -1.83 ) | <.001 | 13 (10 to 17 ) | 7 (2 to 19 ) | -3.34 (-4.83 to -1.82 ) | <.001 |
| Beihai | 9 (7 to 12 ) | 9 (2 to 25 ) | 0.10 (-1.96 to 2.20 ) | 0.926 | 9 (6 to 11 ) | 8 (2 to 22 ) | 0.00 (-2.02 to 2.06 ) | 0.999 | 9 (6 to 12 ) | 9 (2 to 24 ) | 0.05 (-1.99 to 2.14 ) | 0.959 |
| Chongzuo | 11 (8 to 15 ) | 10 (2 to 26 ) | -1.02 (-1.93 to -0.10 ) | 0.030 | 10 (7 to 14 ) | 9 (2 to 23 ) | -1.13 (-2.04 to -0.22 ) | 0.015 | 11 (8 to 15 ) | 9 (2 to 25 ) | -1.07 (-1.97 to -0.16 ) | 0.022 |
| Fangchenggang | 11 (8 to 16 ) | 13 (3 to 33 ) | 0.48 (-2.51 to 3.56 ) | 0.755 | 10 (7 to 15 ) | 11 (3 to 30 ) | 0.36 (-2.68 to 3.49 ) | 0.820 | 11 (8 to 15 ) | 12 (3 to 32 ) | 0.43 (-2.58 to 3.53 ) | 0.784 |
| Guigang | 12 (9 to 15 ) | 8 (2 to 21 ) | -2.36 (-3.78 to -0.91 ) | 0.001 | 11 (8 to 14 ) | 7 (2 to 19 ) | -2.46 (-3.81 to -1.10 ) | <.001 | 12 (9 to 15 ) | 8 (2 to 20 ) | -2.18 (-4.14 to -0.18 ) | 0.033 |
| Guilin | 11 (8 to 14 ) | 6 (2 to 15 ) | -2.89 (-5.88 to 0.20 ) | 0.067 | 10 (7 to 13 ) | 5 (1 to 13 ) | -2.99 (-6.04 to 0.16 ) | 0.062 | 10 (8 to 14 ) | 6 (2 to 14 ) | -2.93 (-5.94 to 0.18 ) | 0.064 |
| Hechi | 14 (11 to 17 ) | 8 (2 to 20 ) | -3.11 (-5.37 to -0.78 ) | 0.009 | 13 (10 to 16 ) | 7 (2 to 18 ) | -3.37 (-5.52 to -1.18 ) | 0.003 | 13 (10 to 16 ) | 7 (2 to 19 ) | -3.13 (-5.08 to -1.13 ) | 0.002 |
| Hezhou | 12 (8 to 16 ) | 7 (2 to 17 ) | -3.44 (-5.49 to -1.34 ) | 0.001 | 11 (8 to 15 ) | 6 (2 to 15 ) | -3.51 (-5.70 to -1.26 ) | 0.002 | 11 (8 to 15 ) | 7 (2 to 16 ) | -3.45 (-5.65 to -1.19 ) | 0.003 |
| Laibin | 12 (9 to 15 ) | 8 (2 to 21 ) | -2.11 (-3.50 to -0.70 ) | 0.003 | 11 (8 to 14 ) | 7 (2 to 19 ) | -2.21 (-3.55 to -0.86 ) | 0.001 | 11 (9 to 14 ) | 7 (2 to 20 ) | -2.15 (-3.49 to -0.79 ) | 0.002 |
| Liuzhou | 11 (9 to 15 ) | 6 (2 to 18 ) | -3.01 (-4.58 to -1.42 ) | <.001 | 11 (8 to 13 ) | 6 (1 to 16 ) | -3.13 (-4.76 to -1.47 ) | <.001 | 11 (8 to 14 ) | 6 (2 to 17 ) | -3.07 (-4.66 to -1.45 ) | <.001 |
| Nanning | 10 (8 to 13 ) | 8 (2 to 21 ) | -1.67 (-3.38 to 0.06 ) | 0.059 | 9 (7 to 12 ) | 7 (2 to 19 ) | -1.81 (-3.53 to -0.05 ) | 0.044 | 10 (8 to 13 ) | 7 (2 to 20 ) | -1.73 (-3.44 to 0.01 ) | 0.051 |
| Qinzhou | 11 (8 to 14 ) | 9 (2 to 23 ) | -1.09 (-3.96 to 1.86 ) | 0.465 | 10 (7 to 13 ) | 8 (2 to 21 ) | -1.22 (-4.18 to 1.83 ) | 0.430 | 10 (8 to 14 ) | 9 (2 to 22 ) | -1.15 (-4.05 to 1.85 ) | 0.448 |
| Wuzhou | 11 (8 to 16 ) | 8 (2 to 20 ) | -2.55 (-3.92 to -1.15 ) | <.001 | 11 (8 to 15 ) | 7 (2 to 18 ) | -2.26 (-4.49 to 0.02 ) | 0.052 | 11 (8 to 15 ) | 7 (2 to 19 ) | -2.59 (-3.97 to -1.19 ) | <.001 |
| Yulin | 11 (8 to 15 ) | 9 (2 to 23 ) | -1.17 (-2.79 to 0.49 ) | 0.165 | 10 (8 to 14 ) | 8 (2 to 21 ) | -1.26 (-2.41 to -0.09 ) | 0.035 | 11 (8 to 14 ) | 9 (2 to 22 ) | -1.12 (-3.36 to 1.17 ) | 0.335 |
| Guizhou |  |  |  |  |  |  |  |  |  |  |  |  |
| Anshun | 20 (15 to 26 ) | 10 (3 to 29 ) | -3.62 (-4.45 to -2.78 ) | <.001 | 19 (14 to 24 ) | 9 (2 to 26 ) | -3.74 (-4.38 to -3.09 ) | <.001 | 20 (15 to 25 ) | 10 (2 to 27 ) | -3.63 (-4.66 to -2.58 ) | <.001 |
| Bijie | 18 (13 to 23 ) | 8 (2 to 24 ) | -4.13 (-5.08 to -3.16 ) | <.001 | 16 (12 to 21 ) | 7 (2 to 21 ) | -4.24 (-5.22 to -3.25 ) | <.001 | 17 (13 to 22 ) | 8 (2 to 23 ) | -4.17 (-5.12 to -3.22 ) | <.001 |
| Guiyang | 16 (12 to 20 ) | 8 (2 to 23 ) | -3.71 (-4.68 to -2.74 ) | <.001 | 14 (11 to 18 ) | 7 (2 to 21 ) | -3.83 (-4.49 to -3.16 ) | <.001 | 15 (11 to 19 ) | 8 (2 to 22 ) | -3.77 (-4.64 to -2.89 ) | <.001 |
| Liupanshui | 20 (14 to 26 ) | 9 (2 to 25 ) | -4.18 (-6.48 to -1.82 ) | 0.001 | 18 (13 to 24 ) | 8 (2 to 23 ) | -4.04 (-5.45 to -2.61 ) | <.001 | 19 (14 to 25 ) | 9 (2 to 24 ) | -4.22 (-5.49 to -2.93 ) | <.001 |
| Qiandongnan Miao and Dong | 19 (14 to 24 ) | 13 (3 to 36 ) | -2.10 (-3.16 to -1.03 ) | <.001 | 18 (13 to 22 ) | 12 (3 to 33 ) | -2.18 (-3.13 to -1.22 ) | <.001 | 19 (14 to 23 ) | 13 (3 to 35 ) | -2.14 (-3.18 to -1.09 ) | <.001 |
| Qiannan Buyei and Miao | 20 (15 to 25 ) | 13 (3 to 36 ) | -2.35 (-2.87 to -1.82 ) | <.001 | 18 (14 to 23 ) | 12 (3 to 32 ) | -2.44 (-2.77 to -2.10 ) | <.001 | 19 (15 to 24 ) | 12 (3 to 34 ) | -2.39 (-2.83 to -1.94 ) | <.001 |
| Qianxinan Buyei and Miao | 20 (15 to 27 ) | 11 (3 to 29 ) | -3.45 (-5.31 to -1.56 ) | <.001 | 19 (13 to 25 ) | 10 (3 to 26 ) | -3.25 (-4.62 to -1.85 ) | <.001 | 19 (14 to 26 ) | 10 (3 to 28 ) | -3.19 (-4.55 to -1.82 ) | <.001 |
| Tongren | 15 (11 to 20 ) | 10 (3 to 31 ) | -2.60 (-4.57 to -0.59 ) | 0.011 | 14 (10 to 19 ) | 9 (2 to 28 ) | -2.70 (-4.60 to -0.76 ) | 0.007 | 15 (11 to 20 ) | 10 (3 to 29 ) | -2.64 (-4.58 to -0.67 ) | 0.009 |
| Zunyi | 15 (11 to 19 ) | 9 (2 to 26 ) | -2.92 (-4.41 to -1.40 ) | <.001 | 13 (10 to 18 ) | 8 (2 to 23 ) | -3.01 (-4.70 to -1.29 ) | 0.001 | 14 (10 to 18 ) | 9 (2 to 25 ) | -2.95 (-4.41 to -1.47 ) | <.001 |
| Hainan |  |  |  |  |  |  |  |  |  |  |  |  |
| Haikou | 6 (4 to 8 ) | 3 (1 to 8 ) | -3.61 (-4.54 to -2.67 ) | <.001 | 5 (4 to 7 ) | 3 (1 to 7 ) | -3.71 (-4.69 to -2.72 ) | <.001 | 6 (4 to 8 ) | 3 (1 to 8 ) | -3.64 (-4.58 to -2.70 ) | <.001 |
| Hainan | 8 (5 to 10 ) | 5 (1 to 14 ) | -2.16 (-2.56 to -1.76 ) | <.001 | 7 (5 to 10 ) | 5 (1 to 13 ) | -2.34 (-2.85 to -1.84 ) | <.001 | 7 (5 to 10 ) | 5 (1 to 14 ) | -2.21 (-2.65 to -1.78 ) | <.001 |
| Sanya | 6 (4 to 10 ) | 5 (1 to 15 ) | -1.40 (-3.00 to 0.23 ) | 0.092 | 6 (4 to 9 ) | 5 (1 to 14 ) | -1.41 (-2.54 to -0.28 ) | 0.015 | 6 (4 to 10 ) | 5 (1 to 15 ) | -1.43 (-3.0 to 0.15 ) | 0.076 |
| Hebei |  |  |  |  |  |  |  |  |  |  |  |  |
| Baoding | 6 (3 to 9 ) | 4 (1 to 10 ) | -2.09 (-2.47 to -1.70 ) | <.001 | 5 (3 to 8 ) | 3 (1 to 9 ) | -2.33 (-3.09 to -1.56 ) | <.001 | 5 (3 to 8 ) | 4 (1 to 10 ) | -2.14 (-2.53 to -1.74 ) | <.001 |
| Cangzhou | 6 (4 to 10 ) | 4 (1 to 13 ) | -1.88 (-2.53 to -1.22 ) | <.001 | 6 (4 to 9 ) | 4 (1 to 11 ) | -2.05 (-2.77 to -1.33 ) | <.001 | 6 (4 to 9 ) | 4 (1 to 12 ) | -1.90 (-2.64 to -1.16 ) | <.001 |
| Chengde | 12 (7 to 19 ) | 8 (2 to 22 ) | -2.48 (-3.69 to -1.26 ) | <.001 | 11 (7 to 18 ) | 7 (2 to 20 ) | -2.58 (-3.75 to -1.39 ) | <.001 | 12 (7 to 19 ) | 7 (2 to 21 ) | -2.53 (-3.72 to -1.32 ) | <.001 |
| Handan | 6 (4 to 10 ) | 3 (1 to 9 ) | -3.62 (-4.68 to -2.55 ) | <.001 | 6 (3 to 10 ) | 3 (1 to 8 ) | -3.73 (-4.80 to -2.64 ) | <.001 | 6 (4 to 10 ) | 3 (1 to 8 ) | -3.67 (-4.73 to -2.59 ) | <.001 |
| Hengshui | 6 (4 to 9 ) | 4 (1 to 12 ) | -2.11 (-2.46 to -1.75 ) | <.001 | 6 (3 to 9 ) | 4 (1 to 11 ) | -2.20 (-2.54 to -1.85 ) | <.001 | 6 (4 to 9 ) | 4 (1 to 11 ) | -2.16 (-2.53 to -1.80 ) | <.001 |
| Langfang | 4 (3 to 7 ) | 4 (1 to 12 ) | -0.08 (-1.06 to 0.91 ) | 0.870 | 4 (2 to 7 ) | 4 (1 to 11 ) | -0.20 (-1.33 to 0.93 ) | 0.724 | 4 (3 to 7 ) | 4 (1 to 12 ) | -0.13 (-1.14 to 0.90 ) | 0.808 |
| Qinhuangdao | 7 (4 to 12 ) | 6 (1 to 18 ) | -1.09 (-2.70 to 0.55 ) | 0.193 | 7 (4 to 11 ) | 5 (1 to 16 ) | -1.18 (-2.76 to 0.41 ) | 0.145 | 7 (4 to 11 ) | 6 (1 to 17 ) | -1.13 (-2.72 to 0.49 ) | 0.171 |
| Shijiazhuang | 5 (3 to 8 ) | 3 (1 to 9 ) | -2.38 (-3.04 to -1.71 ) | <.001 | 5 (3 to 7 ) | 3 (1 to 8 ) | -2.49 (-2.94 to -2.03 ) | <.001 | 5 (3 to 7 ) | 3 (1 to 9 ) | -2.44 (-2.89 to -1.98 ) | <.001 |
| Tangshan | 6 (3 to 10 ) | 5 (1 to 15 ) | -0.72 (-2.32 to 0.90 ) | 0.380 | 6 (3 to 9 ) | 5 (1 to 13 ) | -0.85 (-2.03 to 0.35 ) | 0.164 | 6 (3 to 9 ) | 5 (1 to 14 ) | -0.78 (-1.97 to 0.41 ) | 0.198 |
| Xingtai | 6 (4 to 10 ) | 4 (1 to 11 ) | -2.59 (-3.16 to -2.02 ) | <.001 | 6 (4 to 9 ) | 3 (1 to 10 ) | -2.64 (-3.66 to -1.61 ) | <.001 | 6 (4 to 9 ) | 4 (1 to 10 ) | -2.58 (-3.57 to -1.57 ) | <.001 |
| Zhangjiakou | 10 (6 to 15 ) | 6 (2 to 17 ) | -2.51 (-3.13 to -1.88 ) | <.001 | 9 (5 to 14 ) | 6 (1 to 15 ) | -2.62 (-3.23 to -2.01 ) | <.001 | 10 (6 to 15 ) | 6 (2 to 16 ) | -2.56 (-3.17 to -1.94 ) | <.001 |
| Heilongjiang |  |  |  |  |  |  |  |  |  |  |  |  |
| Daqing | 6 (3 to 11 ) | 3 (1 to 8 ) | -4.25 (-5.63 to -2.85 ) | <.001 | 6 (2 to 10 ) | 2 (0 to 7 ) | -4.33 (-5.20 to -3.45 ) | <.001 | 6 (02 to 11 ) | 3 (1 to 8 ) | -4.26 (-5.63 to -2.87 ) | <.001 |
| Daxing'anling | 9 (3 to 20 ) | 6 (1 to 21 ) | -1.66 (-3.40 to 0.12 ) | 0.068 | 8 (2 to 19 ) | 6 (1 to 19 ) | -1.74 (-3.51 to 0.07 ) | 0.059 | 8 (02 to 20 ) | 6 (1 to 20 ) | -1.69 (-3.44 to 0.09 ) | 0.063 |
| Harbin | 4 (2 to 8 ) | 3 (1 to 11 ) | -1.68 (-2.13 to -1.22 ) | <.001 | 4 (2 to 7 ) | 3 (0 to 10 ) | -1.82 (-2.15 to -1.50 ) | <.001 | 4 (02 to 8 ) | 3 (1 to 11 ) | -1.72 (-2.09 to -1.35 ) | <.001 |
| Hegang | 4 (1 to 9 ) | 3 (0 to 14 ) | -0.60 (-1.69 to 0.50 ) | 0.284 | 4 (1 to 9 ) | 3 (0 to 13 ) | -0.65 (-1.62 to 0.33 ) | 0.190 | 4 (01 to 9 ) | 3 (0 to 14 ) | -0.64 (-1.73 to 0.46 ) | 0.251 |
| Heihe | 6 (2 to 12 ) | 4 (1 to 13 ) | -2.19 (-2.98 to -1.40 ) | <.001 | 5 (2 to 11 ) | 3 (1 to 12 ) | -2.22 (-2.97 to -1.47 ) | <.001 | 6 (02 to 11 ) | 4 (1 to 13 ) | -2.17 (-2.92 to -1.41 ) | <.001 |
| Jiamusi | 4 (1 to 10 ) | 4 (0 to 16 ) | -0.37 (-1.68 to 0.94 ) | 0.577 | 4 (1 to 9 ) | 3 (0 to 14 ) | -0.48 (-1.79 to 0.84 ) | 0.475 | 4 (01 to 10 ) | 4 (0 to 15 ) | -0.42 (-1.72 to 0.89 ) | 0.528 |
| Jixi | 4 (1 to 10 ) | 4 (0 to 16 ) | 0.10 (-1.76 to 1.99 ) | 0.919 | 4 (1 to 9 ) | 4 (0 to 14 ) | 0.11 (-2.10 to 2.37 ) | 0.924 | 4 (01 to 10 ) | 4 (0 to 15 ) | 0.10 (-1.89 to 2.13 ) | 0.922 |
| Mudanjiang | 4 (1 to 8 ) | 4 (1 to 14 ) | 0.40 (-1.13 to 1.94 ) | 0.613 | 3 (1 to 8 ) | 4 (1 to 13 ) | 0.24 (-1.05 to 1.55 ) | 0.716 | 4 (01 to 8 ) | 4 (1 to 14 ) | 0.38 (-1.24 to 2.03 ) | 0.649 |
| Qiqihar | 6 (3 to 12 ) | 3 (1 to 9 ) | -4.07 (-4.83 to -3.30 ) | <.001 | 6 (2 to 11 ) | 3 (0 to 8 ) | -4.14 (-4.79 to -3.49 ) | <.001 | 6 (02 to 11 ) | 3 (0 to 8 ) | -4.10 (-4.81 to -3.39 ) | <.001 |
| Qitaihe | 4 (1 to 9 ) | 4 (0 to 13 ) | -0.43 (-2.03 to 1.19 ) | 0.600 | 4 (1 to 8 ) | 3 (0 to 12 ) | -0.71 (-1.88 to 0.48 ) | 0.243 | 4 (01 to 9 ) | 3 (0 to 13 ) | -0.46 (-2.07 to 1.17 ) | 0.577 |
| Shuangyashan | 4 (1 to 11 ) | 4 (0 to 15 ) | -0.15 (-1.77 to 1.50 ) | 0.860 | 4 (1 to 10 ) | 4 (0 to 14 ) | -0.14 (-2.13 to 1.89 ) | 0.891 | 4 (01 to 11 ) | 4 (0 to 15 ) | -0.15 (-1.91 to 1.65 ) | 0.872 |
| Suihua | 5 (2 to 10 ) | 3 (1 to 10 ) | -3.13 (-3.61 to -2.65 ) | <.001 | 5 (2 to 9 ) | 3 (0 to 9 ) | -3.22 (-3.74 to -2.70 ) | <.001 | 5 (02 to 9 ) | 3 (0 to 9 ) | -3.15 (-3.56 to -2.74 ) | <.001 |
| Yichun | 5 (2 to 11 ) | 4 (1 to 16 ) | -1.07 (-1.83 to -0.30 ) | 0.006 | 5 (1 to 10 ) | 4 (1 to 14 ) | -1.17 (-1.85 to -0.49 ) | 0.001 | 5 (02 to 10 ) | 4 (1 to 15 ) | -1.12 (-1.83 to -0.39 ) | 0.003 |
| Henan |  |  |  |  |  |  |  |  |  |  |  |  |
| Anyang | 8 (5 to 14 ) | 4 (1 to 12 ) | -3.39 (-4.48 to -2.29 ) | <.001 | 8 (4 to 13 ) | 4 (1 to 11 ) | -3.54 (-5.34 to -1.72 ) | <.001 | 8 (4 to 13 ) | 4 (1 to 12 ) | -3.33 (-4.89 to -1.75 ) | <.001 |
| Hebi | 9 (5 to 14 ) | 5 (1 to 13 ) | -3.22 (-4.34 to -2.09 ) | <.001 | 8 (4 to 13 ) | 4 (1 to 12 ) | -3.17 (-4.31 to -2.02 ) | <.001 | 8 (5 to 14 ) | 5 (1 to 13 ) | -3.12 (-4.37 to -1.85 ) | <.001 |
| Jiaozuo | 6 (3 to 12 ) | 5 (1 to 14 ) | -1.49 (-2.73 to -0.25 ) | 0.019 | 6 (3 to 11 ) | 4 (1 to 13 ) | -1.53 (-2.58 to -0.47 ) | 0.005 | 6 (3 to 11 ) | 5 (1 to 13 ) | -1.51 (-2.66 to -0.36 ) | 0.011 |
| Jiyuan shi | 7 (3 to 12 ) | 5 (1 to 15 ) | -1.42 (-2.37 to -0.46 ) | 0.004 | 6 (3 to 11 ) | 5 (1 to 14 ) | -1.46 (-2.34 to -0.58 ) | 0.001 | 6 (3 to 12 ) | 5 (1 to 14 ) | -1.44 (-2.35 to -0.53 ) | 0.002 |
| Kaifeng | 8 (4 to 14 ) | 5 (1 to 13 ) | -2.88 (-3.56 to -2.20 ) | <.001 | 8 (4 to 13 ) | 4 (1 to 12 ) | -3.07 (-3.77 to -2.38 ) | <.001 | 8 (4 to 14 ) | 4 (1 to 12 ) | -2.94 (-3.56 to -2.33 ) | <.001 |
| Luohe | 6 (3 to 12 ) | 4 (1 to 11 ) | -2.37 (-3.12 to -1.61 ) | <.001 | 6 (3 to 11 ) | 4 (1 to 10 ) | -2.46 (-3.40 to -1.50 ) | <.001 | 6 (3 to 11 ) | 4 (1 to 11 ) | -2.41 (-3.31 to -1.50 ) | <.001 |
| Luoyang | 6 (3 to 11 ) | 5 (1 to 14 ) | -1.59 (-3.01 to -0.15 ) | 0.030 | 6 (3 to 10 ) | 4 (1 to 12 ) | -1.69 (-2.58 to -0.78 ) | <.001 | 6 (3 to 11 ) | 5 (1 to 13 ) | -1.66 (-2.66 to -0.65 ) | 0.001 |
| Nanyang | 9 (4 to 15 ) | 6 (2 to 16 ) | -2.09 (-3.22 to -0.95 ) | <.001 | 8 (4 to 14 ) | 5 (1 to 14 ) | -2.24 (-3.32 to -1.15 ) | <.001 | 8 (4 to 15 ) | 5 (1 to 15 ) | -2.18 (-3.27 to -1.08 ) | <.001 |
| Pingdingshan | 6 (3 to 12 ) | 4 (1 to 12 ) | -1.91 (-2.50 to -1.30 ) | <.001 | 6 (3 to 11 ) | 4 (1 to 11 ) | -2.00 (-2.48 to -1.51 ) | <.001 | 6 (3 to 11 ) | 4 (1 to 11 ) | -1.93 (-2.40 to -1.45 ) | <.001 |
| Puyang | 9 (6 to 15 ) | 5 (1 to 13 ) | -3.75 (-4.90 to -2.59 ) | <.001 | 9 (5 to 14 ) | 4 (1 to 11 ) | -3.83 (-4.98 to -2.66 ) | <.001 | 9 (5 to 14 ) | 4 (1 to 12 ) | -3.80 (-4.95 to -2.63 ) | <.001 |
| Sanmenxia | 9 (4 to 16 ) | 6 (2 to 18 ) | -1.69 (-2.76 to -0.60 ) | 0.002 | 8 (4 to 14 ) | 6 (1 to 16 ) | -1.87 (-3.37 to -0.35 ) | 0.016 | 8 (4 to 15 ) | 6 (2 to 17 ) | -1.71 (-2.80 to -0.62 ) | 0.002 |
| Shangqiu | 10 (6 to 16 ) | 6 (2 to 16 ) | -2.77 (-3.88 to -1.64 ) | <.001 | 9 (6 to 15 ) | 6 (2 to 15 ) | -2.70 (-4.08 to -1.30 ) | <.001 | 10 (6 to 16 ) | 6 (2 to 15 ) | -2.80 (-3.90 to -1.68 ) | <.001 |
| Xinxiang | 8 (4 to 14 ) | 5 (1 to 13 ) | -2.60 (-3.78 to -1.41 ) | <.001 | 7 (4 to 13 ) | 4 (1 to 12 ) | -2.66 (-3.70 to -1.60 ) | <.001 | 8 (4 to 13 ) | 5 (1 to 13 ) | -2.63 (-3.74 to -1.50 ) | <.001 |
| Xinyang | 12 (6 to 21 ) | 7 (2 to 20 ) | -2.60 (-4.24 to -0.93 ) | 0.002 | 11 (6 to 20 ) | 7 (2 to 18 ) | -2.62 (-4.39 to -0.81 ) | 0.005 | 12 (6 to 21 ) | 7 (2 to 19 ) | -2.62 (-4.32 to -0.88 ) | 0.003 |
| Xuchang | 5 (2 to 10 ) | 3 (1 to 9 ) | -2.04 (-2.83 to -1.25 ) | <.001 | 5 (2 to 9 ) | 3 (1 to 8 ) | -2.11 (-3.00 to -1.21 ) | <.001 | 5 (02 to 9 ) | 3 (1 to 9 ) | -2.11 (-3.09 to -1.12 ) | <.001 |
| Zhengzhou | 5 (3 to 10 ) | 4 (1 to 11 ) | -2.02 (-3.51 to -0.50 ) | 0.009 | 5 (2 to 9 ) | 3 (1 to 10 ) | -2.09 (-3.47 to -0.69 ) | 0.004 | 5 (3 to 9 ) | 4 (1 to 10 ) | -2.05 (-3.50 to -0.58 ) | 0.006 |
| Zhoukou | 10 (5 to 17 ) | 6 (2 to 16 ) | -2.87 (-4.27 to -1.46 ) | <.001 | 9 (5 to 16 ) | 5 (2 to 14 ) | -2.92 (-4.28 to -1.55 ) | <.001 | 9 (5 to 16 ) | 5 (2 to 15 ) | -2.90 (-4.26 to -1.51 ) | <.001 |
| Zhumadian | 10 (5 to 19 ) | 6 (2 to 17 ) | -2.46 (-3.18 to -1.74 ) | <.001 | 9 (5 to 17 ) | 6 (2 to 15 ) | -2.67 (-3.50 to -1.84 ) | <.001 | 10 (5 to 18 ) | 6 (2 to 16 ) | -2.63 (-3.47 to -1.78 ) | <.001 |
| Hubei |  |  |  |  |  |  |  |  |  |  |  |  |
| Enshi Tujia and Miao | 9 (5 to 14 ) | 5 (1 to 14 ) | -2.79 (-5.21 to -0.31 ) | 0.027 | 9 (5 to 13 ) | 5 (1 to 12 ) | -2.87 (-5.31 to -0.37 ) | 0.025 | 9 (5 to 13 ) | 5 (1 to 13 ) | -2.83 (-5.25 to -0.34 ) | 0.026 |
| Ezhou | 6 (3 to 11 ) | 4 (1 to 10 ) | -2.39 (-3.28 to -1.49 ) | <.001 | 6 (3 to 10 ) | 4 (1 to 9 ) | -2.46 (-3.32 to -1.59 ) | <.001 | 6 (3 to 11 ) | 4 (1 to 10 ) | -2.36 (-3.26 to -1.45 ) | <.001 |
| Huanggang | 9 (5 to 15 ) | 5 (1 to 13 ) | -3.02 (-3.86 to -2.16 ) | <.001 | 8 (4 to 14 ) | 4 (1 to 12 ) | -3.14 (-3.75 to -2.52 ) | <.001 | 8 (4 to 14 ) | 5 (1 to 12 ) | -2.97 (-3.91 to -2.03 ) | <.001 |
| Huangshi | 8 (4 to 14 ) | 5 (1 to 12 ) | -2.59 (-3.69 to -1.48 ) | <.001 | 7 (4 to 13 ) | 4 (1 to 11 ) | -2.74 (-4.02 to -1.44 ) | <.001 | 8 (4 to 13 ) | 5 (1 to 12 ) | -2.70 (-4.07 to -1.32 ) | <.001 |
| Jingmen | 7 (4 to 11 ) | 4 (1 to 11 ) | -2.63 (-3.34 to -1.91 ) | <.001 | 6 (3 to 11 ) | 4 (1 to 10 ) | -3.00 (-3.68 to -2.32 ) | <.001 | 7 (4 to 11 ) | 4 (1 to 10 ) | -2.67 (-3.36 to -1.97 ) | <.001 |
| Jingzhou | 7 (4 to 12 ) | 4 (1 to 12 ) | -2.86 (-3.21 to -2.52 ) | <.001 | 7 (4 to 11 ) | 4 (1 to 11 ) | -2.94 (-3.32 to -2.56 ) | <.001 | 7 (4 to 12 ) | 4 (1 to 11 ) | -2.90 (-3.25 to -2.54 ) | <.001 |
| Qianjiang | 7 (4 to 12 ) | 4 (1 to 12 ) | -2.67 (-3.27 to -2.08 ) | <.001 | 7 (4 to 11 ) | 4 (1 to 11 ) | -2.74 (-3.42 to -2.06 ) | <.001 | 7 (4 to 12 ) | 4 (1 to 12 ) | -2.72 (-3.29 to -2.14 ) | <.001 |
| Shennongjia | 9 (4 to 15 ) | 6 (2 to 16 ) | -2.14 (-4.46 to 0.24 ) | 0.078 | 8 (4 to 14 ) | 5 (1 to 14 ) | -2.29 (-3.57 to -0.99 ) | 0.001 | 8 (4 to 14 ) | 6 (2 to 15 ) | -2.08 (-4.75 to 0.68 ) | 0.138 |
| Shiyan | 8 (4 to 15 ) | 5 (1 to 13 ) | -3.09 (-4.82 to -1.34 ) | 0.001 | 8 (4 to 14 ) | 4 (1 to 12 ) | -3.43 (-4.05 to -2.81 ) | <.001 | 8 (4 to 14 ) | 4 (1 to 13 ) | -3.39 (-4.01 to -2.77 ) | <.001 |
| Suizhou Shi | 7 (4 to 13 ) | 4 (1 to 11 ) | -2.69 (-3.40 to -1.98 ) | <.001 | 7 (3 to 12 ) | 4 (1 to 10 ) | -2.78 (-3.50 to -2.06 ) | <.001 | 7 (3 to 12 ) | 4 (1 to 11 ) | -2.80 (-3.55 to -2.04 ) | <.001 |
| Tianmen | 7 (4 to 12 ) | 4 (1 to 12 ) | -2.44 (-3.32 to -1.55 ) | <.001 | 7 (4 to 11 ) | 4 (1 to 11 ) | -2.50 (-3.49 to -1.51 ) | <.001 | 7 (4 to 12 ) | 4 (1 to 12 ) | -2.45 (-3.48 to -1.40 ) | <.001 |
| Wuhan | 4 (2 to 8 ) | 3 (1 to 7 ) | -2.37 (-3.07 to -1.66 ) | <.001 | 4 (2 to 7 ) | 2 (1 to 6 ) | -2.37 (-3.50 to -1.23 ) | <.001 | 4 (02 to 8 ) | 3 (1 to 7 ) | -2.37 (-3.24 to -1.50 ) | <.001 |
| Xiangfan | 7 (3 to 12 ) | 4 (1 to 11 ) | -2.90 (-4.52 to -1.25 ) | 0.001 | 6 (3 to 11 ) | 4 (1 to 10 ) | -2.98 (-4.38 to -1.55 ) | <.001 | 7 (3 to 12 ) | 4 (1 to 10 ) | -2.91 (-4.52 to -1.28 ) | 0.001 |
| Xianning | 8 (4 to 13 ) | 5 (1 to 13 ) | -2.16 (-2.73 to -1.59 ) | <.001 | 7 (4 to 12 ) | 5 (1 to 12 ) | -2.23 (-2.69 to -1.76 ) | <.001 | 7 (4 to 13 ) | 5 (1 to 13 ) | -2.19 (-2.71 to -1.67 ) | <.001 |
| Xiantao | 7 (4 to 11 ) | 4 (1 to 11 ) | -2.51 (-3.27 to -1.74 ) | <.001 | 6 (3 to 10 ) | 4 (1 to 10 ) | -2.62 (-3.28 to -1.96 ) | <.001 | 6 (3 to 11 ) | 4 (1 to 11 ) | -2.55 (-3.34 to -1.75 ) | <.001 |
| Xiaogan | 6 (3 to 11 ) | 4 (1 to 11 ) | -2.58 (-3.28 to -1.88 ) | <.001 | 6 (3 to 10 ) | 4 (1 to 10 ) | -2.80 (-3.39 to -2.21 ) | <.001 | 6 (3 to 11 ) | 4 (1 to 10 ) | -2.59 (-3.49 to -1.67 ) | <.001 |
| Yichang | 8 (4 to 13 ) | 4 (1 to 12 ) | -3.02 (-3.90 to -2.13 ) | <.001 | 7 (4 to 12 ) | 4 (1 to 11 ) | -3.08 (-4.64 to -1.48 ) | <.001 | 8 (4 to 12 ) | 4 (1 to 12 ) | -3.05 (-3.94 to -2.15 ) | <.001 |
| Hunan |  |  |  |  |  |  |  |  |  |  |  |  |
| Changde | 8 (5 to 11 ) | 5 (1 to 15 ) | -1.86 (-2.85 to -0.85 ) | <.001 | 7 (4 to 11 ) | 5 (1 to 14 ) | -1.95 (-3.20 to -0.69 ) | 0.003 | 7 (5 to 11 ) | 5 (1 to 14 ) | -1.85 (-3.20 to -0.49 ) | 0.008 |
| Changsha | 6 (4 to 10 ) | 4 (1 to 12 ) | -2.03 (-3.46 to -0.57 ) | 0.006 | 6 (4 to 9 ) | 4 (1 to 11 ) | -1.85 (-2.94 to -0.75 ) | 0.001 | 6 (4 to 9 ) | 4 (1 to 11 ) | -1.82 (-3.09 to -0.54 ) | 0.005 |
| Chenzhou | 11 (7 to 16 ) | 7 (2 to 18 ) | -2.32 (-2.98 to -1.66 ) | <.001 | 10 (6 to 15 ) | 7 (2 to 17 ) | -2.43 (-3.24 to -1.62 ) | <.001 | 11 (7 to 15 ) | 7 (2 to 18 ) | -2.29 (-3.30 to -1.27 ) | <.001 |
| Hengyang | 9 (6 to 13 ) | 6 (2 to 14 ) | -2.36 (-2.99 to -1.73 ) | <.001 | 9 (5 to 12 ) | 5 (1 to 13 ) | -2.51 (-3.08 to -1.95 ) | <.001 | 9 (6 to 12 ) | 6 (1 to 13 ) | -2.47 (-3.28 to -1.66 ) | <.001 |
| Huaihua | 13 (9 to 17 ) | 8 (2 to 22 ) | -2.41 (-3.36 to -1.45 ) | <.001 | 12 (9 to 16 ) | 7 (2 to 20 ) | -2.49 (-3.49 to -1.48 ) | <.001 | 12 (9 to 16 ) | 8 (2 to 21 ) | -2.45 (-3.41 to -1.47 ) | <.001 |
| Loudi | 8 (6 to 12 ) | 6 (1 to 14 ) | -2.24 (-2.55 to -1.93 ) | <.001 | 8 (5 to 11 ) | 5 (1 to 13 ) | -2.35 (-2.69 to -2.00 ) | <.001 | 8 (5 to 11 ) | 5 (1 to 13 ) | -2.28 (-2.58 to -1.98 ) | <.001 |
| Shaoyang | 10 (7 to 13 ) | 6 (2 to 15 ) | -2.81 (-3.43 to -2.18 ) | <.001 | 9 (7 to 12 ) | 5 (1 to 13 ) | -2.78 (-3.59 to -1.96 ) | <.001 | 10 (7 to 13 ) | 6 (1 to 14 ) | -2.74 (-3.53 to -1.94 ) | <.001 |
| Xiangtan | 7 (5 to 11 ) | 5 (1 to 14 ) | -1.72 (-2.84 to -0.59 ) | 0.003 | 7 (4 to 10 ) | 5 (1 to 13 ) | -1.65 (-2.44 to -0.86 ) | <.001 | 7 (4 to 11 ) | 5 (1 to 13 ) | -1.61 (-2.69 to -0.52 ) | 0.004 |
| Xiangxi Tujia and Miao | 11 (8 to 16 ) | 7 (2 to 19 ) | -2.55 (-3.68 to -1.39 ) | <.001 | 10 (7 to 14 ) | 6 (2 to 17 ) | -2.66 (-3.79 to -1.51 ) | <.001 | 11 (7 to 15 ) | 7 (2 to 18 ) | -2.59 (-3.73 to -1.45 ) | <.001 |
| Yiyang | 8 (5 to 11 ) | 6 (1 to 15 ) | -1.59 (-2.62 to -0.55 ) | 0.003 | 7 (5 to 10 ) | 5 (1 to 14 ) | -1.69 (-2.56 to -0.80 ) | <.001 | 7 (5 to 11 ) | 5 (1 to 14 ) | -1.65 (-2.68 to -0.60 ) | 0.002 |
| Yongzhou | 11 (7 to 15 ) | 7 (2 to 18 ) | -2.22 (-2.98 to -1.46 ) | <.001 | 10 (7 to 14 ) | 7 (2 to 16 ) | -2.33 (-3.10 to -1.55 ) | <.001 | 11 (7 to 14 ) | 7 (2 to 17 ) | -2.27 (-3.10 to -1.43 ) | <.001 |
| Yueyang | 7 (4 to 12 ) | 6 (2 to 14 ) | -1.29 (-2.62 to 0.05 ) | 0.058 | 7 (4 to 11 ) | 5 (1 to 13 ) | -1.36 (-2.33 to -0.37 ) | 0.007 | 7 (4 to 11 ) | 5 (2 to 14 ) | -1.34 (-2.25 to -0.43 ) | 0.004 |
| Zhangjiajie | 9 (6 to 13 ) | 6 (2 to 18 ) | -2.23 (-3.70 to -0.74 ) | 0.004 | 8 (5 to 12 ) | 6 (1 to 16 ) | -2.02 (-4.16 to 0.16 ) | 0.070 | 9 (5 to 13 ) | 6 (2 to 17 ) | -2.05 (-3.32 to -0.77 ) | 0.002 |
| Zhuzhou | 9 (5 to 13 ) | 6 (2 to 15 ) | -1.83 (-2.50 to -1.16 ) | <.001 | 8 (5 to 12 ) | 6 (1 to 14 ) | -1.75 (-2.50 to -1.00 ) | <.001 | 8 (5 to 12 ) | 6 (2 to 14 ) | -1.88 (-2.51 to -1.23 ) | <.001 |
| Jiangsu |  |  |  |  |  |  |  |  |  |  |  |  |
| Changzhou | 5 (3 to 9 ) | 4 (1 to 12 ) | -2.01 (-3.18 to -0.83 ) | 0.001 | 5 (3 to 8 ) | 3 (1 to 10 ) | -2.22 (-3.37 to -1.05 ) | <.001 | 5 (3 to 9 ) | 3 (1 to 11 ) | -1.99 (-3.31 to -0.66 ) | 0.003 |
| Huai'an | 6 (4 to 10 ) | 4 (1 to 13 ) | -1.79 (-2.24 to -1.35 ) | <.001 | 6 (4 to 9 ) | 4 (1 to 12 ) | -1.88 (-2.40 to -1.35 ) | <.001 | 6 (4 to 10 ) | 4 (1 to 13 ) | -1.77 (-2.44 to -1.08 ) | <.001 |
| Lianyungang | 6 (4 to 9 ) | 4 (1 to 12 ) | -2.00 (-2.83 to -1.16 ) | <.001 | 5 (3 to 8 ) | 4 (1 to 11 ) | -2.19 (-3.21 to -1.17 ) | <.001 | 6 (3 to 9 ) | 4 (1 to 11 ) | -2.03 (-2.90 to -1.16 ) | <.001 |
| Nanjing | 5 (3 to 8 ) | 3 (1 to 10 ) | -2.36 (-3.62 to -1.07 ) | <.001 | 5 (3 to 8 ) | 3 (1 to 9 ) | -2.42 (-3.58 to -1.25 ) | <.001 | 5 (3 to 8 ) | 3 (1 to 9 ) | -2.43 (-4.06 to -0.76 ) | 0.004 |
| Nantong | 5 (3 to 9 ) | 4 (1 to 12 ) | -1.64 (-3.74 to 0.50 ) | 0.133 | 5 (2 to 9 ) | 3 (1 to 11 ) | -1.74 (-3.76 to 0.33 ) | 0.100 | 5 (3 to 9 ) | 4 (1 to 12 ) | -1.68 (-3.75 to 0.43 ) | 0.118 |
| Suqian | 7 (4 to 10 ) | 4 (1 to 13 ) | -2.05 (-2.85 to -1.25 ) | <.001 | 6 (4 to 9 ) | 4 (1 to 12 ) | -2.25 (-2.74 to -1.76 ) | <.001 | 6 (4 to 10 ) | 4 (1 to 12 ) | -2.20 (-2.72 to -1.67 ) | <.001 |
| Suzhou | 4 (2 to 8 ) | 3 (1 to 9 ) | -2.43 (-4.50 to -0.30 ) | 0.025 | 4 (2 to 7 ) | 2 (1 to 8 ) | -2.52 (-4.51 to -0.49 ) | 0.015 | 4 (02 to 8 ) | 3 (1 to 9 ) | -2.47 (-4.51 to -0.38 ) | 0.021 |
| Taizhou | 6 (3 to 10 ) | 4 (1 to 13 ) | -2.01 (-3.60 to -0.40 ) | 0.015 | 5 (3 to 9 ) | 4 (1 to 12 ) | -2.06 (-3.60 to -0.49 ) | 0.010 | 6 (3 to 10 ) | 4 (1 to 12 ) | -2.03 (-3.59 to -0.45 ) | 0.012 |
| Wuxi | 5 (3 to 8 ) | 3 (1 to 10 ) | -2.39 (-3.50 to -1.27 ) | <.001 | 4 (2 to 8 ) | 3 (1 to 9 ) | -2.46 (-3.56 to -1.36 ) | <.001 | 5 (02 to 8 ) | 3 (1 to 10 ) | -2.42 (-3.52 to -1.31 ) | <.001 |
| Xuzhou | 6 (4 to 10 ) | 4 (1 to 10 ) | -3.27 (-4.47 to -2.06 ) | <.001 | 6 (4 to 9 ) | 3 (1 to 9 ) | -3.11 (-4.38 to -1.82 ) | <.001 | 6 (4 to 9 ) | 3 (1 to 10 ) | -3.08 (-4.14 to -2.02 ) | <.001 |
| Yancheng | 7 (4 to 11 ) | 5 (1 to 14 ) | -2.06 (-2.79 to -1.33 ) | <.001 | 6 (4 to 10 ) | 4 (1 to 13 ) | -2.15 (-2.74 to -1.56 ) | <.001 | 6 (4 to 10 ) | 4 (1 to 14 ) | -2.09 (-2.77 to -1.40 ) | <.001 |
| Yangzhou | 6 (4 to 10 ) | 5 (1 to 15 ) | -1.55 (-2.04 to -1.06 ) | <.001 | 6 (3 to 9 ) | 4 (1 to 13 ) | -1.63 (-2.02 to -1.25 ) | <.001 | 6 (4 to 9 ) | 4 (1 to 14 ) | -1.59 (-2.08 to -1.09 ) | <.001 |
| Zhenjiang | 6 (3 to 9 ) | 4 (1 to 14 ) | -1.70 (-3.05 to -0.33 ) | 0.015 | 5 (3 to 8 ) | 4 (1 to 12 ) | -1.69 (-2.62 to -0.75 ) | <.001 | 5 (3 to 9 ) | 4 (1 to 13 ) | -1.68 (-2.75 to -0.60 ) | 0.002 |
| Jiangxi |  |  |  |  |  |  |  |  |  |  |  |  |
| Fuzhou | 9 (4 to 16 ) | 5 (1 to 13 ) | -3.33 (-3.97 to -2.69 ) | <.001 | 8 (4 to 14 ) | 4 (1 to 12 ) | -3.21 (-3.68 to -2.74 ) | <.001 | 9 (4 to 15 ) | 5 (1 to 13 ) | -3.36 (-3.95 to -2.77 ) | <.001 |
| Ganzhou | 10 (6 to 18 ) | 5 (1 to 14 ) | -3.67 (-4.60 to -2.73 ) | <.001 | 10 (5 to 16 ) | 5 (1 to 13 ) | -3.77 (-4.73 to -2.81 ) | <.001 | 10 (5 to 17 ) | 5 (1 to 13 ) | -3.74 (-4.49 to -2.98 ) | <.001 |
| Ji'an | 9 (5 to 15 ) | 5 (1 to 14 ) | -3.22 (-4.16 to -2.27 ) | <.001 | 9 (5 to 14 ) | 5 (1 to 12 ) | -3.29 (-3.99 to -2.59 ) | <.001 | 9 (5 to 14 ) | 5 (1 to 13 ) | -3.24 (-4.13 to -2.34 ) | <.001 |
| Jingdezhen | 9 (5 to 16 ) | 5 (1 to 14 ) | -3.38 (-4.40 to -2.35 ) | <.001 | 9 (4 to 15 ) | 5 (1 to 12 ) | -3.43 (-4.59 to -2.27 ) | <.001 | 9 (5 to 16 ) | 5 (1 to 13 ) | -3.41 (-4.48 to -2.32 ) | <.001 |
| Jiujiang | 8 (4 to 13 ) | 5 (1 to 13 ) | -2.36 (-3.59 to -1.11 ) | <.001 | 7 (4 to 12 ) | 5 (1 to 12 ) | -2.46 (-3.82 to -1.08 ) | 0.001 | 8 (4 to 12 ) | 5 (1 to 12 ) | -2.39 (-3.64 to -1.13 ) | <.001 |
| Nanchang | 6 (3 to 9 ) | 3 (1 to 8 ) | -3.39 (-4.57 to -2.20 ) | <.001 | 5 (3 to 9 ) | 3 (1 to 7 ) | -3.47 (-4.42 to -2.50 ) | <.001 | 6 (3 to 9 ) | 3 (1 to 7 ) | -3.63 (-4.55 to -2.71 ) | <.001 |
| Pingxiang | 7 (4 to 12 ) | 4 (1 to 11 ) | -2.78 (-3.29 to -2.27 ) | <.001 | 7 (4 to 11 ) | 4 (1 to 10 ) | -2.86 (-3.39 to -2.33 ) | <.001 | 7 (4 to 11 ) | 4 (1 to 11 ) | -2.80 (-3.20 to -2.40 ) | <.001 |
| Shangrao | 9 (4 to 16 ) | 5 (1 to 14 ) | -3.24 (-4.15 to -2.33 ) | <.001 | 8 (4 to 14 ) | 5 (1 to 12 ) | -3.30 (-4.21 to -2.38 ) | <.001 | 9 (4 to 15 ) | 5 (1 to 13 ) | -3.27 (-4.17 to -2.36 ) | <.001 |
| Xinyu | 7 (4 to 13 ) | 4 (1 to 11 ) | -2.99 (-3.43 to -2.54 ) | <.001 | 7 (4 to 12 ) | 4 (1 to 10 ) | -3.15 (-3.64 to -2.66 ) | <.001 | 7 (4 to 12 ) | 4 (1 to 10 ) | -2.94 (-3.61 to -2.27 ) | <.001 |
| Yichun | 7 (4 to 12 ) | 5 (1 to 12 ) | -2.70 (-3.40 to -1.99 ) | <.001 | 7 (4 to 11 ) | 4 (1 to 11 ) | -2.73 (-3.52 to -1.93 ) | <.001 | 7 (4 to 12 ) | 4 (1 to 11 ) | -2.70 (-3.46 to -1.93 ) | <.001 |
| Yingtan | 9 (5 to 16 ) | 5 (1 to 15 ) | -3.06 (-3.96 to -2.15 ) | <.001 | 9 (4 to 15 ) | 5 (1 to 13 ) | -3.15 (-3.73 to -2.57 ) | <.001 | 9 (4 to 16 ) | 5 (1 to 14 ) | -3.08 (-3.98 to -2.16 ) | <.001 |
| Jilin |  |  |  |  |  |  |  |  |  |  |  |  |
| Baicheng | 10 (5 to 18 ) | 4 (1 to 13 ) | -5.06 (-7.19 to -2.88 ) | <.001 | 9 (4 to 17 ) | 4 (1 to 12 ) | -5.13 (-6.64 to -3.61 ) | <.001 | 10 (5 to 17 ) | 4 (1 to 12 ) | -5.11 (-6.76 to -3.43 ) | <.001 |
| Baishan | 5 (2 to 10 ) | 5 (1 to 16 ) | -0.45 (-2.44 to 1.58 ) | 0.660 | 5 (2 to 9 ) | 4 (1 to 14 ) | -0.48 (-3.01 to 2.12 ) | 0.717 | 5 (02 to 10 ) | 5 (1 to 15 ) | -0.46 (-2.68 to 1.80 ) | 0.685 |
| Changchun | 5 (2 to 10 ) | 4 (1 to 11 ) | -2.09 (-2.83 to -1.34 ) | <.001 | 5 (2 to 9 ) | 3 (1 to 10 ) | -2.14 (-3.00 to -1.28 ) | <.001 | 5 (02 to 9 ) | 3 (1 to 11 ) | -2.14 (-2.88 to -1.39 ) | <.001 |
| Jilin | 5 (2 to 10 ) | 4 (1 to 14 ) | -0.84 (-1.30 to -0.37 ) | <.001 | 5 (2 to 9 ) | 4 (1 to 12 ) | -0.94 (-1.47 to -0.41 ) | <.001 | 5 (02 to 9 ) | 4 (1 to 13 ) | -0.88 (-1.33 to -0.42 ) | <.001 |
| Liaoyuan | 6 (3 to 10 ) | 3 (1 to 11 ) | -2.76 (-3.63 to -1.88 ) | <.001 | 5 (3 to 10 ) | 3 (1 to 10 ) | -2.78 (-4.19 to -1.35 ) | <.001 | 6 (3 to 10 ) | 3 (1 to 11 ) | -2.77 (-3.83 to -1.69 ) | <.001 |
| Siping | 7 (3 to 12 ) | 3 (1 to 10 ) | -3.82 (-4.34 to -3.30 ) | <.001 | 6 (3 to 11 ) | 3 (1 to 9 ) | -3.91 (-4.31 to -3.51 ) | <.001 | 7 (3 to 11 ) | 3 (1 to 9 ) | -3.83 (-4.32 to -3.34 ) | <.001 |
| Songyuan | 7 (4 to 13 ) | 4 (1 to 12 ) | -3.47 (-4.68 to -2.25 ) | <.001 | 7 (3 to 12 ) | 4 (1 to 11 ) | -3.49 (-4.44 to -2.53 ) | <.001 | 7 (3 to 13 ) | 4 (1 to 11 ) | -3.48 (-4.53 to -2.43 ) | <.001 |
| Tonghua | 6 (3 to 10 ) | 4 (1 to 13 ) | -1.86 (-3.54 to -0.16 ) | 0.032 | 5 (2 to 9 ) | 4 (1 to 12 ) | -1.89 (-4.09 to 0.36 ) | 0.099 | 6 (3 to 10 ) | 4 (1 to 12 ) | -1.88 (-3.76 to 0.04 ) | 0.055 |
| Yanbian Korean | 4 (1 to 9 ) | 5 (1 to 19 ) | 1.38 (-0.81 to 3.62 ) | 0.219 | 4 (1 to 8 ) | 5 (1 to 17 ) | 1.21 (0.43 to 1.99 ) | 0.002 | 4 (01 to 8 ) | 5 (1 to 18 ) | 1.28 (0.42 to 2.14 ) | 0.003 |
| Liaoning |  |  |  |  |  |  |  |  |  |  |  |  |
| Anshan | 4 (2 to 6 ) | 3 (1 to 9 ) | -1.50 (-2.89 to -0.08 ) | 0.038 | 3 (2 to 6 ) | 2 (1 to 8 ) | -1.70 (-2.56 to -0.82 ) | <.001 | 4 (02 to 6 ) | 3 (1 to 8 ) | -1.40 (-2.57 to -0.21 ) | 0.021 |
| Benxi | 4 (2 to 7 ) | 4 (1 to 12 ) | -0.52 (-1.71 to 0.69 ) | 0.401 | 4 (2 to 6 ) | 3 (1 to 11 ) | -0.65 (-1.73 to 0.44 ) | 0.239 | 4 (02 to 7 ) | 3 (1 to 12 ) | -0.53 (-1.93 to 0.89 ) | 0.463 |
| Chaoyang | 9 (5 to 13 ) | 4 (1 to 12 ) | -4.46 (-6.96 to -1.90 ) | 0.001 | 8 (4 to 12 ) | 4 (1 to 11 ) | -4.49 (-6.44 to -2.51 ) | <.001 | 8 (5 to 13 ) | 4 (1 to 11 ) | -4.48 (-6.72 to -2.18 ) | <.001 |
| Dalian | 3 (2 to 5 ) | 3 (1 to 9 ) | -0.58 (-1.56 to 0.41 ) | 0.253 | 3 (1 to 5 ) | 2 (1 to 8 ) | -0.70 (-1.51 to 0.11 ) | 0.092 | 3 (01 to 5 ) | 2 (1 to 8 ) | -0.64 (-1.59 to 0.32 ) | 0.190 |
| Dandong | 4 (2 to 8 ) | 4 (1 to 13 ) | -0.54 (-2.98 to 1.97 ) | 0.671 | 4 (2 to 7 ) | 3 (1 to 12 ) | -0.84 (-2.44 to 0.78 ) | 0.307 | 4 (02 to 7 ) | 4 (1 to 13 ) | -0.55 (-3.30 to 2.27 ) | 0.699 |
| Fushun | 4 (2 to 7 ) | 4 (1 to 13 ) | -0.56 (-1.47 to 0.36 ) | 0.231 | 4 (2 to 7 ) | 3 (1 to 12 ) | -0.68 (-1.35 to -0.01 ) | 0.047 | 4 (02 to 7 ) | 4 (1 to 12 ) | -0.63 (-1.48 to 0.22 ) | 0.146 |
| Fuxin | 6 (3 to 9 ) | 3 (1 to 8 ) | -3.87 (-4.17 to -3.57 ) | <.001 | 5 (3 to 8 ) | 2 (1 to 8 ) | -3.97 (-4.63 to -3.30 ) | <.001 | 5 (3 to 9 ) | 3 (1 to 8 ) | -3.90 (-4.18 to -3.63 ) | <.001 |
| Huludao | 6 (4 to 10 ) | 4 (1 to 11 ) | -3.17 (-4.70 to -1.62 ) | <.001 | 6 (3 to 9 ) | 3 (1 to 10 ) | -3.21 (-4.31 to -2.09 ) | <.001 | 6 (4 to 9 ) | 3 (1 to 11 ) | -3.19 (-4.51 to -1.85 ) | <.001 |
| Jinzhou | 5 (3 to 8 ) | 2 (1 to 7 ) | -3.76 (-4.13 to -3.38 ) | <.001 | 4 (2 to 7 ) | 2 (0 to 7 ) | -3.90 (-4.39 to -3.41 ) | <.001 | 5 (3 to 8 ) | 2 (0 to 7 ) | -3.79 (-4.18 to -3.39 ) | <.001 |
| Liaoyang | 4 (2 to 6 ) | 3 (1 to 9 ) | -1.22 (-2.67 to 0.25 ) | 0.103 | 3 (2 to 6 ) | 3 (1 to 8 ) | -1.38 (-2.07 to -0.69 ) | <.001 | 4 (02 to 6 ) | 3 (1 to 9 ) | -1.23 (-2.95 to 0.51 ) | 0.165 |
| Panjin | 3 (2 to 6 ) | 2 (0 to 6 ) | -2.78 (-3.34 to -2.20 ) | <.001 | 3 (2 to 5 ) | 2 (0 to 6 ) | -2.85 (-3.43 to -2.27 ) | <.001 | 3 (02 to 5 ) | 2 (0 to 6 ) | -2.81 (-3.38 to -2.24 ) | <.001 |
| Shenyang | 3 (2 to 5 ) | 2 (0 to 7 ) | -1.57 (-2.49 to -0.65 ) | 0.001 | 3 (1 to 5 ) | 2 (0 to 6 ) | -1.81 (-2.82 to -0.78 ) | 0.001 | 3 (02 to 5 ) | 2 (0 to 6 ) | -1.81 (-2.66 to -0.96 ) | <.001 |
| Tieling | 5 (3 to 8 ) | 3 (1 to 11 ) | -1.85 (-2.61 to -1.08 ) | <.001 | 4 (3 to 7 ) | 3 (1 to 10 ) | -1.99 (-2.47 to -1.51 ) | <.001 | 5 (3 to 7 ) | 3 (1 to 10 ) | -1.95 (-2.38 to -1.51 ) | <.001 |
| Inner Mongolia |  |  |  |  |  |  |  |  |  |  |  |  |
| Alxa | 6 (3 to 11 ) | 2 (1 to 5 ) | -6.20 (-9.58 to -2.70 ) | 0.001 | 6 (3 to 10 ) | 2 (1 to 5 ) | -5.86 (-8.96 to -2.65 ) | <.001 | 6 (3 to 10 ) | 2 (1 to 5 ) | -6.24 (-9.59 to -2.76 ) | 0.001 |
| Baotou | 3 (2 to 6 ) | 1 (0 to 3 ) | -4.99 (-6.70 to -3.25 ) | <.001 | 3 (2 to 5 ) | 1 (0 to 3 ) | -5.08 (-6.72 to -3.41 ) | <.001 | 3 (02 to 5 ) | 1 (0 to 3 ) | -5.03 (-6.71 to -3.32 ) | <.001 |
| Baynnur | 5 (3 to 9 ) | 2 (0 to 5 ) | -5.74 (-7.79 to -3.65 ) | <.001 | 5 (3 to 8 ) | 2 (0 to 4 ) | -6.10 (-8.06 to -4.10 ) | <.001 | 5 (3 to 9 ) | 2 (0 to 4 ) | -6.06 (-8.06 to -4.02 ) | <.001 |
| Chifeng | 6 (3 to 9 ) | 2 (1 to 7 ) | -4.36 (-5.00 to -3.71 ) | <.001 | 5 (3 to 8 ) | 2 (0 to 6 ) | -4.56 (-5.19 to -3.92 ) | <.001 | 5 (3 to 9 ) | 2 (1 to 6 ) | -4.52 (-5.38 to -3.66 ) | <.001 |
| Hohhot | 3 (2 to 5 ) | 1 (0 to 4 ) | -4.39 (-5.54 to -3.22 ) | <.001 | 3 (1 to 5 ) | 1 (0 to 3 ) | -4.48 (-6.03 to -2.91 ) | <.001 | 3 (02 to 5 ) | 1 (0 to 3 ) | -4.46 (-6.11 to -2.79 ) | <.001 |
| Hulunbuir | 5 (2 to 8 ) | 4 (1 to 10 ) | -1.61 (-3.66 to 0.48 ) | 0.130 | 4 (2 to 8 ) | 3 (1 to 9 ) | -1.57 (-3.36 to 0.24 ) | 0.089 | 5 (02 to 8 ) | 3 (1 to 10 ) | -1.52 (-3.36 to 0.36 ) | 0.112 |
| Ordos | 4 (2 to 6 ) | 1 (0 to 4 ) | -4.98 (-6.75 to -3.17 ) | <.001 | 3 (2 to 6 ) | 1 (0 to 3 ) | -5.09 (-6.94 to -3.20 ) | <.001 | 4 (02 to 6 ) | 1 (0 to 3 ) | -5.03 (-6.83 to -3.18 ) | <.001 |
| Tongliao | 4 (2 to 6 ) | 2 (0 to 6 ) | -3.68 (-4.82 to -2.52 ) | <.001 | 4 (2 to 6 ) | 2 (0 to 5 ) | -3.85 (-4.87 to -2.82 ) | <.001 | 4 (02 to 6 ) | 2 (0 to 6 ) | -3.79 (-4.88 to -2.68 ) | <.001 |
| Ulaan Chab | 4 (2 to 6 ) | 2 (0 to 5 ) | -4.04 (-5.39 to -2.67 ) | <.001 | 4 (2 to 6 ) | 2 (0 to 5 ) | -4.08 (-5.80 to -2.33 ) | <.001 | 4 (02 to 6 ) | 2 (0 to 5 ) | -4.07 (-5.67 to -2.44 ) | <.001 |
| Wuhai | 5 (3 to 10 ) | 2 (0 to 4 ) | -6.37 (-8.98 to -3.67 ) | <.001 | 5 (2 to 9 ) | 1 (0 to 4 ) | -6.80 (-9.79 to -3.71 ) | <.001 | 5 (02 to 9 ) | 2 (0 to 4 ) | -6.40 (-9.0 to -3.73 ) | <.001 |
| Xilin Gol | 8 (6 to 11 ) | 3 (1 to 7 ) | -5.39 (-6.12 to -4.65 ) | <.001 | 7 (5 to 10 ) | 3 (1 to 6 ) | -5.51 (-6.34 to -4.67 ) | <.001 | 8 (5 to 10 ) | 3 (1 to 7 ) | -5.44 (-6.21 to -4.67 ) | <.001 |
| Xing'an | 5 (2 to 8 ) | 3 (1 to 10 ) | -2.57 (-4.44 to -0.66 ) | 0.008 | 5 (2 to 8 ) | 3 (1 to 9 ) | -2.94 (-4.67 to -1.17 ) | 0.001 | 5 (02 to 8 ) | 3 (1 to 9 ) | -2.58 (-4.72 to -0.39 ) | 0.021 |
| Ningxia |  |  |  |  |  |  |  |  |  |  |  |  |
| Guyuan | 5 (2 to 9 ) | 2 (0 to 5 ) | -5.05 (-6.05 to -4.04 ) | <.001 | 4 (2 to 9 ) | 2 (0 to 5 ) | -5.17 (-6.28 to -4.05 ) | <.001 | 5 (02 to 9 ) | 2 (0 to 5 ) | -5.11 (-6.23 to -3.97 ) | <.001 |
| Shizuishan | 5 (2 to 9 ) | 2 (1 to 6 ) | -4.73 (-6.17 to -3.27 ) | <.001 | 5 (2 to 8 ) | 2 (0 to 6 ) | -4.81 (-6.15 to -3.45 ) | <.001 | 5 (02 to 9 ) | 2 (0 to 6 ) | -4.76 (-6.16 to -3.35 ) | <.001 |
| Wuzhong | 5 (2 to 10 ) | 2 (0 to 5 ) | -5.08 (-5.28 to -4.88 ) | <.001 | 5 (2 to 9 ) | 2 (0 to 5 ) | -5.19 (-5.45 to -4.93 ) | <.001 | 5 (02 to 10 ) | 2 (0 to 5 ) | -5.13 (-5.31 to -4.94 ) | <.001 |
| Yinchuan | 5 (2 to 9 ) | 2 (0 to 5 ) | -5.26 (-6.19 to -4.32 ) | <.001 | 4 (2 to 9 ) | 2 (0 to 5 ) | -5.45 (-6.42 to -4.46 ) | <.001 | 5 (02 to 9 ) | 2 (0 to 5 ) | -5.29 (-6.23 to -4.34 ) | <.001 |
| Zhongwei | 5 (2 to 11 ) | 2 (0 to 5 ) | -4.89 (-5.47 to -4.29 ) | <.001 | 5 (2 to 10 ) | 2 (0 to 5 ) | -4.97 (-5.74 to -4.20 ) | <.001 | 5 (02 to 10 ) | 2 (0 to 5 ) | -4.95 (-5.58 to -4.30 ) | <.001 |
| Qinghai |  |  |  |  |  |  |  |  |  |  |  |  |
| Golog Tibetan | 15 (6 to 25 ) | 13 (3 to 34 ) | -0.71 (-1.42 to 0.01 ) | 0.052 | 14 (6 to 23 ) | 12 (3 to 30 ) | -0.77 (-1.69 to 0.16 ) | 0.106 | 15 (6 to 24 ) | 12 (3 to 32 ) | -0.75 (-1.48 to -0.01 ) | 0.047 |
| Gyêgu Tibetan | 14 (5 to 23 ) | 12 (3 to 34 ) | -0.89 (-1.90 to 0.13 ) | 0.087 | 13 (5 to 22 ) | 11 (2 to 30 ) | -0.89 (-1.82 to 0.04 ) | 0.062 | 13 (5 to 23 ) | 11 (3 to 32 ) | -0.89 (-2.18 to 0.42 ) | 0.181 |
| Haibei Tibetan | 11 (5 to 19 ) | 7 (2 to 20 ) | -2.62 (-3.41 to -1.83 ) | <.001 | 10 (5 to 18 ) | 6 (2 to 18 ) | -2.72 (-3.26 to -2.17 ) | <.001 | 11 (5 to 19 ) | 7 (2 to 19 ) | -2.54 (-3.55 to -1.53 ) | <.001 |
| Haidong | 9 (3 to 16 ) | 3 (1 to 9 ) | -4.72 (-5.38 to -4.05 ) | <.001 | 8 (3 to 14 ) | 3 (1 to 8 ) | -4.78 (-5.50 to -4.05 ) | <.001 | 8 (3 to 15 ) | 3 (1 to 8 ) | -4.77 (-5.43 to -4.10 ) | <.001 |
| Hainan Tibetan | 12 (5 to 21 ) | 6 (1 to 15 ) | -3.56 (-4.74 to -2.38 ) | <.001 | 12 (5 to 19 ) | 6 (1 to 14 ) | -3.73 (-4.29 to -3.17 ) | <.001 | 12 (5 to 20 ) | 6 (1 to 14 ) | -3.62 (-4.74 to -2.50 ) | <.001 |
| Haixi Mongol and Tibetan | 12 (5 to 21 ) | 7 (2 to 18 ) | -2.55 (-3.85 to -1.24 ) | <.001 | 11 (4 to 19 ) | 7 (2 to 16 ) | -2.63 (-3.85 to -1.40 ) | <.001 | 11 (5 to 20 ) | 7 (2 to 17 ) | -2.59 (-3.85 to -1.31 ) | <.001 |
| Huangnan Tibetan | 13 (5 to 22 ) | 7 (2 to 18 ) | -3.22 (-4.20 to -2.24 ) | <.001 | 12 (5 to 20 ) | 6 (2 to 16 ) | -3.30 (-4.19 to -2.41 ) | <.001 | 12 (5 to 21 ) | 7 (2 to 17 ) | -3.26 (-4.19 to -2.32 ) | <.001 |
| Xining | 8 (3 to 15 ) | 3 (1 to 7 ) | -5.07 (-6.08 to -4.05 ) | <.001 | 7 (3 to 13 ) | 3 (1 to 7 ) | -5.42 (-6.66 to -4.17 ) | <.001 | 8 (3 to 14 ) | 3 (1 to 7 ) | -5.38 (-6.67 to -4.08 ) | <.001 |
| Shaanxi |  |  |  |  |  |  |  |  |  |  |  |  |
| Ankang | 9 (4 to 15 ) | 6 (2 to 17 ) | -1.49 (-2.82 to -0.14 ) | 0.031 | 8 (4 to 14 ) | 6 (1 to 16 ) | -1.58 (-3.16 to 0.02 ) | 0.053 | 8 (4 to 15 ) | 6 (2 to 16 ) | -1.50 (-2.86 to -0.12 ) | 0.033 |
| Baoji | 8 (4 to 17 ) | 4 (1 to 11 ) | -3.91 (-4.80 to -3.01 ) | <.001 | 8 (3 to 15 ) | 4 (1 to 10 ) | -4.09 (-5.00 to -3.18 ) | <.001 | 8 (3 to 16 ) | 4 (1 to 10 ) | -4.04 (-4.97 to -3.11 ) | <.001 |
| Hanzhong | 10 (5 to 19 ) | 6 (2 to 16 ) | -2.61 (-4.06 to -1.14 ) | 0.001 | 9 (4 to 17 ) | 5 (1 to 14 ) | -2.61 (-4.52 to -0.65 ) | 0.009 | 10 (4 to 18 ) | 6 (1 to 15 ) | -2.56 (-4.47 to -0.62 ) | 0.010 |
| Shangluo | 8 (3 to 14 ) | 7 (2 to 18 ) | -0.29 (-1.25 to 0.68 ) | 0.558 | 7 (3 to 13 ) | 6 (2 to 16 ) | -0.35 (-1.50 to 0.80 ) | 0.547 | 7 (3 to 13 ) | 7 (2 to 17 ) | -0.35 (-1.42 to 0.73 ) | 0.526 |
| Tongchuan | 6 (2 to 11 ) | 3 (1 to 10 ) | -2.74 (-3.47 to -2.00 ) | <.001 | 5 (2 to 11 ) | 3 (1 to 9 ) | -2.86 (-3.70 to -2.00 ) | <.001 | 6 (02 to 11 ) | 3 (1 to 9 ) | -2.79 (-3.56 to -2.01 ) | <.001 |
| Weinan | 6 (2 to 11 ) | 4 (1 to 10 ) | -2.41 (-3.41 to -1.40 ) | <.001 | 5 (2 to 10 ) | 3 (1 to 9 ) | -2.41 (-3.28 to -1.54 ) | <.001 | 5 (02 to 10 ) | 3 (1 to 10 ) | -2.41 (-3.30 to -1.52 ) | <.001 |
| Xi'an | 4 (2 to 8 ) | 2 (1 to 7 ) | -2.98 (-3.83 to -2.13 ) | <.001 | 4 (2 to 8 ) | 2 (1 to 6 ) | -3.10 (-4.06 to -2.13 ) | <.001 | 4 (02 to 8 ) | 2 (1 to 6 ) | -3.03 (-3.89 to -2.17 ) | <.001 |
| Xianyang | 6 (2 to 12 ) | 3 (1 to 9 ) | -3.12 (-4.24 to -1.98 ) | <.001 | 5 (2 to 11 ) | 3 (1 to 8 ) | -3.29 (-3.88 to -2.70 ) | <.001 | 6 (02 to 12 ) | 3 (1 to 8 ) | -3.34 (-4.20 to -2.47 ) | <.001 |
| Yan'an | 8 (3 to 16 ) | 4 (1 to 11 ) | -3.39 (-4.25 to -2.52 ) | <.001 | 7 (3 to 14 ) | 4 (1 to 10 ) | -3.49 (-4.36 to -2.60 ) | <.001 | 8 (3 to 15 ) | 4 (1 to 11 ) | -3.43 (-4.30 to -2.56 ) | <.001 |
| Yulin | 9 (4 to 16 ) | 4 (1 to 10 ) | -4.11 (-6.11 to -2.06 ) | <.001 | 8 (4 to 15 ) | 4 (1 to 9 ) | -4.16 (-6.01 to -2.27 ) | <.001 | 9 (4 to 15 ) | 4 (1 to 10 ) | -4.16 (-6.03 to -2.24 ) | <.001 |
| Shandong |  |  |  |  |  |  |  |  |  |  |  |  |
| Binzhou | 4 (2 to 6 ) | 3 (1 to 7 ) | -2.11 (-2.85 to -1.36 ) | <.001 | 4 (2 to 6 ) | 2 (1 to 6 ) | -2.19 (-2.97 to -1.41 ) | <.001 | 4 (02 to 6 ) | 2 (1 to 7 ) | -2.15 (-2.90 to -1.38 ) | <.001 |
| Dezhou | 4 (2 to 5 ) | 2 (1 to 6 ) | -2.55 (-3.51 to -1.58 ) | <.001 | 3 (2 to 5 ) | 2 (1 to 6 ) | -2.63 (-3.55 to -1.71 ) | <.001 | 3 (02 to 5 ) | 2 (1 to 6 ) | -2.59 (-3.53 to -1.64 ) | <.001 |
| Dongying | 4 (2 to 6 ) | 2 (1 to 7 ) | -2.31 (-3.12 to -1.49 ) | <.001 | 3 (2 to 5 ) | 2 (1 to 6 ) | -2.40 (-3.14 to -1.65 ) | <.001 | 3 (02 to 5 ) | 2 (1 to 7 ) | -2.35 (-3.12 to -1.57 ) | <.001 |
| Heze | 4 (3 to 7 ) | 2 (1 to 5 ) | -3.96 (-5.16 to -2.74 ) | <.001 | 4 (2 to 6 ) | 2 (0 to 5 ) | -4.12 (-4.92 to -3.31 ) | <.001 | 4 (02 to 6 ) | 2 (1 to 5 ) | -4.06 (-4.90 to -3.22 ) | <.001 |
| Jinan | 3 (2 to 4 ) | 2 (0 to 5 ) | -2.75 (-3.72 to -1.78 ) | <.001 | 2 (1 to 4 ) | 2 (0 to 4 ) | -2.69 (-3.56 to -1.83 ) | <.001 | 3 (02 to 4 ) | 2 (0 to 4 ) | -2.66 (-3.66 to -1.65 ) | <.001 |
| Jining | 4 (2 to 6 ) | 2 (1 to 6 ) | -3.55 (-4.12 to -2.97 ) | <.001 | 4 (2 to 6 ) | 2 (0 to 5 ) | -3.68 (-4.12 to -3.23 ) | <.001 | 4 (02 to 6 ) | 2 (1 to 5 ) | -3.62 (-4.11 to -3.13 ) | <.001 |
| Laiwu | 3 (2 to 4 ) | 2 (0 to 5 ) | -2.21 (-2.99 to -1.43 ) | <.001 | 3 (2 to 4 ) | 2 (0 to 4 ) | -2.33 (-3.27 to -1.39 ) | <.001 | 3 (02 to 4 ) | 2 (0 to 5 ) | -2.32 (-3.23 to -1.40 ) | <.001 |
| Liaocheng | 4 (2 to 6 ) | 2 (0 to 5 ) | -3.84 (-5.42 to -2.24 ) | <.001 | 4 (2 to 6 ) | 2 (0 to 5 ) | -3.91 (-5.24 to -2.57 ) | <.001 | 4 (02 to 6 ) | 2 (0 to 5 ) | -3.87 (-5.33 to -2.40 ) | <.001 |
| Linyi | 3 (2 to 5 ) | 2 (1 to 6 ) | -1.47 (-2.44 to -0.49 ) | 0.003 | 3 (2 to 5 ) | 2 (1 to 6 ) | -1.56 (-2.78 to -0.34 ) | 0.013 | 3 (02 to 5 ) | 2 (1 to 6 ) | -1.54 (-2.68 to -0.38 ) | 0.009 |
| Qingdao | 2 (1 to 4 ) | 2 (1 to 7 ) | -0.50 (-1.80 to 0.81 ) | 0.454 | 2 (1 to 4 ) | 2 (0 to 6 ) | -0.61 (-1.84 to 0.63 ) | 0.334 | 02 (01 to 4 ) | 2 (0 to 6 ) | -0.55 (-1.82 to 0.74 ) | 0.401 |
| Rizhao | 3 (2 to 4 ) | 2 (1 to 7 ) | -1.07 (-1.99 to -0.14 ) | 0.024 | 2 (1 to 4 ) | 2 (0 to 6 ) | -1.27 (-1.97 to -0.56 ) | <.001 | 3 (01 to 4 ) | 2 (1 to 6 ) | -1.19 (-1.99 to -0.38 ) | 0.004 |
| Tai'an | 3 (2 to 5 ) | 2 (0 to 5 ) | -2.57 (-3.37 to -1.76 ) | <.001 | 3 (2 to 4 ) | 2 (0 to 4 ) | -2.61 (-3.46 to -1.74 ) | <.001 | 3 (02 to 4 ) | 2 (0 to 5 ) | -2.59 (-3.44 to -1.73 ) | <.001 |
| Weifang | 3 (2 to 5 ) | 2 (1 to 7 ) | -1.80 (-2.38 to -1.21 ) | <.001 | 3 (2 to 5 ) | 2 (0 to 6 ) | -1.89 (-2.53 to -1.25 ) | <.001 | 3 (02 to 5 ) | 2 (1 to 6 ) | -1.84 (-2.45 to -1.23 ) | <.001 |
| Weihai | 2 (1 to 4 ) | 2 (0 to 7 ) | 0.62 (-2.42 to 3.75 ) | 0.694 | 2 (1 to 3 ) | 2 (0 to 6 ) | 0.48 (-2.37 to 3.42 ) | 0.743 | 02 (01 to 3 ) | 2 (0 to 6 ) | 0.56 (-2.40 to 3.61 ) | 0.715 |
| Yantai | 2 (1 to 4 ) | 2 (1 to 8 ) | 0.31 (-2.06 to 2.74 ) | 0.801 | 2 (1 to 4 ) | 2 (1 to 7 ) | 0.21 (-2.07 to 2.54 ) | 0.859 | 02 (01 to 4 ) | 2 (1 to 7 ) | 0.26 (-2.07 to 2.65 ) | 0.826 |
| Zaozhuang | 3 (2 to 5 ) | 2 (1 to 7 ) | -2.37 (-3.63 to -1.10 ) | <.001 | 3 (2 to 5 ) | 2 (1 to 6 ) | -2.30 (-3.35 to -1.25 ) | <.001 | 3 (02 to 5 ) | 2 (1 to 6 ) | -2.41 (-3.81 to -1.0 ) | 0.001 |
| Zibo | 3 (2 to 5 ) | 2 (0 to 5 ) | -2.59 (-3.57 to -1.59 ) | <.001 | 3 (2 to 5 ) | 2 (0 to 5 ) | -2.64 (-3.22 to -2.06 ) | <.001 | 3 (02 to 5 ) | 2 (0 to 5 ) | -2.60 (-3.15 to -2.04 ) | <.001 |
| Shanghai |  |  |  |  |  |  |  |  |  |  |  |  |
| Shanghai | 4 (2 to 8 ) | 3 (1 to 12 ) | -1.09 (-1.24 to -0.93 ) | <.001 | 4 (2 to 7 ) | 3 (1 to 10 ) | -1.18 (-1.39 to -0.96 ) | <.001 | 4 (02 to 8 ) | 3 (1 to 11 ) | -1.13 (-1.32 to -0.95 ) | <.001 |
| Shanxi |  |  |  |  |  |  |  |  |  |  |  |  |
| Changzhi | 6 (3 to 11 ) | 4 (1 to 13 ) | -1.77 (-2.70 to -0.84 ) | <.001 | 6 (3 to 10 ) | 4 (1 to 12 ) | -1.87 (-2.93 to -0.80 ) | 0.001 | 6 (3 to 10 ) | 4 (1 to 12 ) | -1.81 (-2.80 to -0.82 ) | <.001 |
| Datong | 9 (5 to 15 ) | 5 (1 to 15 ) | -2.76 (-4.85 to -0.63 ) | 0.011 | 8 (5 to 13 ) | 5 (1 to 13 ) | -3.13 (-4.71 to -1.52 ) | <.001 | 9 (5 to 14 ) | 5 (1 to 14 ) | -2.80 (-4.85 to -0.70 ) | 0.009 |
| Jincheng | 6 (3 to 11 ) | 4 (1 to 13 ) | -1.49 (-2.43 to -0.55 ) | 0.002 | 5 (3 to 10 ) | 4 (1 to 12 ) | -1.56 (-2.62 to -0.49 ) | 0.004 | 6 (3 to 10 ) | 4 (1 to 12 ) | -1.52 (-2.51 to -0.53 ) | 0.003 |
| Jinzhong | 6 (3 to 11 ) | 4 (1 to 12 ) | -2.17 (-2.37 to -1.97 ) | <.001 | 6 (3 to 10 ) | 4 (1 to 11 ) | -2.29 (-2.50 to -2.07 ) | <.001 | 6 (3 to 10 ) | 4 (1 to 11 ) | -2.23 (-2.53 to -1.92 ) | <.001 |
| Linfen | 7 (3 to 12 ) | 4 (1 to 11 ) | -2.80 (-3.42 to -2.18 ) | <.001 | 6 (3 to 12 ) | 4 (1 to 10 ) | -2.91 (-3.50 to -2.31 ) | <.001 | 7 (3 to 12 ) | 4 (1 to 11 ) | -2.85 (-3.45 to -2.24 ) | <.001 |
| Luliang | 8 (4 to 14 ) | 4 (1 to 12 ) | -3.33 (-3.75 to -2.91 ) | <.001 | 8 (3 to 13 ) | 4 (1 to 11 ) | -3.35 (-3.96 to -2.72 ) | <.001 | 8 (4 to 14 ) | 4 (1 to 11 ) | -3.37 (-3.78 to -2.97 ) | <.001 |
| Shuozhou | 9 (5 to 14 ) | 5 (1 to 13 ) | -3.35 (-5.22 to -1.44 ) | 0.001 | 8 (5 to 13 ) | 4 (1 to 12 ) | -3.43 (-5.26 to -1.56 ) | <.001 | 9 (5 to 14 ) | 5 (1 to 13 ) | -3.38 (-5.23 to -1.50 ) | <.001 |
| Taiyuan | 5 (2 to 8 ) | 3 (1 to 8 ) | -2.62 (-3.14 to -2.10 ) | <.001 | 4 (2 to 8 ) | 3 (1 to 7 ) | -2.72 (-3.23 to -2.20 ) | <.001 | 5 (02 to 8 ) | 3 (1 to 8 ) | -2.66 (-3.18 to -2.15 ) | <.001 |
| Xinzhou | 9 (5 to 14 ) | 5 (1 to 13 ) | -2.73 (-3.59 to -1.86 ) | <.001 | 8 (4 to 13 ) | 5 (1 to 12 ) | -2.84 (-3.89 to -1.78 ) | <.001 | 8 (4 to 13 ) | 5 (1 to 13 ) | -2.80 (-3.82 to -1.77 ) | <.001 |
| Yangquan | 6 (3 to 9 ) | 4 (1 to 12 ) | -1.23 (-1.62 to -0.83 ) | <.001 | 5 (3 to 9 ) | 4 (1 to 11 ) | -1.33 (-1.78 to -0.87 ) | <.001 | 5 (3 to 9 ) | 4 (1 to 11 ) | -1.27 (-1.69 to -0.86 ) | <.001 |
| Yuncheng | 7 (3 to 13 ) | 4 (1 to 12 ) | -2.41 (-3.24 to -1.58 ) | <.001 | 6 (3 to 12 ) | 4 (1 to 11 ) | -2.48 (-4.10 to -0.84 ) | 0.003 | 6 (3 to 12 ) | 4 (1 to 12 ) | -2.45 (-3.31 to -1.58 ) | <.001 |
| Sichuan* |  |  |  |  |  |  |  |  |  |  |  |  |
| Bazhong | 10 (5 to 17 ) | 4 (1 to 12 ) | -4.38 (-5.72 to -3.02 ) | <.001 | 9 (4 to 16 ) | 4 (1 to 11 ) | -4.47 (-5.77 to -3.16 ) | <.001 | 9 (5 to 16 ) | 4 (1 to 11 ) | -4.42 (-5.74 to -3.08 ) | <.001 |
| Chengdu | 5 (2 to 10 ) | 2 (0 to 4 ) | -6.37 (-7.27 to -5.46 ) | <.001 | 5 (2 to 9 ) | 1 (0 to 4 ) | -6.45 (-7.21 to -5.68 ) | <.001 | 5 (02 to 10 ) | 1 (0 to 4 ) | -6.41 (-7.29 to -5.53 ) | <.001 |
| Dazhou | 9 (4 to 14 ) | 4 (1 to 10 ) | -4.41 (-5.86 to -2.95 ) | <.001 | 8 (4 to 13 ) | 4 (1 to 9 ) | -4.30 (-6.44 to -2.12 ) | <.001 | 8 (4 to 14 ) | 4 (1 to 10 ) | -4.46 (-5.87 to -3.02 ) | <.001 |
| Deyang | 6 (3 to 12 ) | 2 (1 to 6 ) | -5.85 (-6.92 to -4.77 ) | <.001 | 6 (3 to 11 ) | 2 (0 to 5 ) | -5.93 (-6.90 to -4.95 ) | <.001 | 6 (3 to 11 ) | 2 (1 to 6 ) | -5.88 (-6.85 to -4.90 ) | <.001 |
| Garzê Tibetan | 23 (10 to 38 ) | 10 (2 to 29 ) | -4.66 (-7.61 to -1.61 ) | 0.003 | 21 (9 to 36 ) | 9 (2 to 26 ) | -4.73 (-6.98 to -2.42 ) | <.001 | 22 (10 to 37 ) | 9 (2 to 28 ) | -4.73 (-7.28 to -2.10 ) | <.001 |
| Guang'an | 8 (4 to 13 ) | 3 (1 to 9 ) | -5.19 (-6.97 to -3.37 ) | <.001 | 7 (4 to 12 ) | 3 (1 to 8 ) | -4.96 (-6.20 to -3.71 ) | <.001 | 8 (4 to 13 ) | 3 (1 to 9 ) | -5.21 (-6.94 to -3.44 ) | <.001 |
| Guangyuan | 10 (5 to 19 ) | 4 (1 to 12 ) | -4.81 (-6.18 to -3.41 ) | <.001 | 10 (4 to 17 ) | 4 (1 to 11 ) | -4.90 (-6.31 to -3.47 ) | <.001 | 10 (4 to 18 ) | 4 (1 to 11 ) | -4.85 (-6.24 to -3.44 ) | <.001 |
| Leshan | 9 (5 to 16 ) | 3 (1 to 8 ) | -5.94 (-7.00 to -4.87 ) | <.001 | 8 (4 to 14 ) | 3 (1 to 7 ) | -5.90 (-7.23 to -4.56 ) | <.001 | 9 (5 to 15 ) | 3 (1 to 8 ) | -5.96 (-7.0 to -4.90 ) | <.001 |
| Liangshan Yi | 15 (8 to 24 ) | 4 (1 to 12 ) | -6.57 (-7.67 to -5.45 ) | <.001 | 13 (7 to 22 ) | 4 (1 to 11 ) | -6.52 (-7.43 to -5.59 ) | <.001 | 14 (8 to 23 ) | 4 (1 to 11 ) | -6.49 (-7.43 to -5.55 ) | <.001 |
| Luzhou | 11 (7 to 15 ) | 3 (1 to 10 ) | -5.67 (-6.86 to -4.47 ) | <.001 | 10 (6 to 14 ) | 3 (1 to 9 ) | -5.75 (-6.76 to -4.74 ) | <.001 | 10 (6 to 15 ) | 3 (1 to 10 ) | -5.69 (-6.90 to -4.46 ) | <.001 |
| Meishan | 7 (4 to 13 ) | 2 (1 to 7 ) | -5.52 (-6.51 to -4.52 ) | <.001 | 7 (3 to 12 ) | 2 (1 to 6 ) | -5.73 (-6.82 to -4.63 ) | <.001 | 7 (3 to 12 ) | 2 (1 to 6 ) | -5.70 (-6.70 to -4.69 ) | <.001 |
| Mianyang | 8 (4 to 15 ) | 3 (1 to 8 ) | -5.41 (-6.62 to -4.20 ) | <.001 | 7 (3 to 14 ) | 2 (1 to 7 ) | -5.50 (-6.40 to -4.59 ) | <.001 | 8 (3 to 14 ) | 3 (1 to 7 ) | -5.45 (-6.66 to -4.22 ) | <.001 |
| Nanchong | 8 (4 to 14 ) | 3 (1 to 9 ) | -4.93 (-5.69 to -4.16 ) | <.001 | 7 (4 to 13 ) | 3 (1 to 8 ) | -5.02 (-5.87 to -4.16 ) | <.001 | 8 (4 to 13 ) | 3 (1 to 8 ) | -4.96 (-5.70 to -4.21 ) | <.001 |
| Ngawa Tibetan and Qiang | 16 (7 to 26 ) | 6 (2 to 15 ) | -5.33 (-6.47 to -4.17 ) | <.001 | 14 (6 to 24 ) | 5 (1 to 14 ) | -5.35 (-7.25 to -3.41 ) | <.001 | 15 (6 to 26 ) | 6 (1 to 14 ) | -5.37 (-6.51 to -4.22 ) | <.001 |
| Panzhihua | 10 (5 to 19 ) | 3 (1 to 9 ) | -6.25 (-7.95 to -4.51 ) | <.001 | 10 (4 to 18 ) | 3 (1 to 8 ) | -6.34 (-8.04 to -4.61 ) | <.001 | 10 (5 to 19 ) | 3 (1 to 8 ) | -6.29 (-7.99 to -4.56 ) | <.001 |
| Suining | 6 (3 to 10 ) | 2 (1 to 6 ) | -5.62 (-6.39 to -4.84 ) | <.001 | 5 (3 to 10 ) | 2 (0 to 5 ) | -5.70 (-6.52 to -4.87 ) | <.001 | 6 (3 to 10 ) | 2 (1 to 5 ) | -5.65 (-6.40 to -4.89 ) | <.001 |
| Ya'an | 11 (5 to 20 ) | 4 (1 to 10 ) | -6.10 (-7.05 to -5.13 ) | <.001 | 11 (5 to 18 ) | 3 (1 to 9 ) | -6.10 (-7.26 to -4.92 ) | <.001 | 11 (5 to 19 ) | 3 (1 to 9 ) | -6.13 (-6.75 to -5.51 ) | <.001 |
| Yibin | 10 (6 to 16 ) | 3 (1 to 8 ) | -6.34 (-7.61 to -5.05 ) | <.001 | 10 (6 to 15 ) | 3 (1 to 8 ) | -6.49 (-7.70 to -5.26 ) | <.001 | 10 (6 to 16 ) | 3 (1 to 8 ) | -6.45 (-7.69 to -5.19 ) | <.001 |
| Zigong | 7 (4 to 11 ) | 2 (1 to 6 ) | -5.86 (-7.26 to -4.45 ) | <.001 | 6 (4 to 10 ) | 2 (0 to 6 ) | -6.13 (-7.35 to -4.90 ) | <.001 | 7 (4 to 11 ) | 2 (0 to 6 ) | -6.08 (-7.34 to -4.81 ) | <.001 |
| Ziyang | 6 (3 to 10 ) | 2 (1 to 6 ) | -5.14 (-5.77 to -4.50 ) | <.001 | 5 (3 to 10 ) | 2 (0 to 5 ) | -5.37 (-6.12 to -4.61 ) | <.001 | 6 (3 to 10 ) | 2 (1 to 6 ) | -5.16 (-5.88 to -4.44 ) | <.001 |
| Tianjin |  |  |  |  |  |  |  |  |  |  |  |  |
| Tianjin | 4 (3 to 7 ) | 3 (1 to 9 ) | -2.01 (-2.06 to -1.95 ) | <.001 | 4 (2 to 7 ) | 3 (1 to 8 ) | -2.16 (-2.28 to -2.03 ) | <.001 | 4 (3 to 7 ) | 3 (1 to 8 ) | -2.05 (-2.15 to -1.96 ) | <.001 |
| Xinjiang |  |  |  |  |  |  |  |  |  |  |  |  |
| Aksu | 9 (2 to 22 ) | 4 (1 to 14 ) | -3.61 (-4.58 to -2.64 ) | <.001 | 8 (2 to 20 ) | 4 (1 to 13 ) | -3.99 (-5.78 to -2.18 ) | <.001 | 9 (02 to 21 ) | 4 (1 to 14 ) | -3.67 (-4.61 to -2.72 ) | <.001 |
| Altay | 9 (5 to 15 ) | 9 (2 to 28 ) | -0.22 (-2.36 to 1.97 ) | 0.844 | 9 (4 to 14 ) | 8 (2 to 25 ) | -0.32 (-2.57 to 1.99 ) | 0.786 | 9 (5 to 15 ) | 9 (2 to 27 ) | -0.26 (-2.45 to 1.98 ) | 0.817 |
| Bayin'gholin Mongol | 7 (2 to 18 ) | 7 (1 to 23 ) | -0.22 (-2.44 to 2.05 ) | 0.848 | 7 (2 to 16 ) | 6 (1 to 21 ) | -0.31 (-2.53 to 1.96 ) | 0.787 | 7 (02 to 17 ) | 7 (1 to 22 ) | -0.25 (-2.45 to 1.99 ) | 0.824 |
| Bortala Mongol | 9 (2 to 21 ) | 6 (1 to 23 ) | -1.89 (-2.92 to -0.84 ) | <.001 | 8 (2 to 20 ) | 6 (1 to 21 ) | -1.98 (-3.04 to -0.92 ) | <.001 | 9 (02 to 21 ) | 6 (1 to 22 ) | -1.93 (-2.97 to -0.88 ) | <.001 |
| Changji Hui | 5 (2 to 10 ) | 6 (1 to 19 ) | 0.91 (-2.45 to 4.39 ) | 0.599 | 4 (2 to 9 ) | 5 (1 to 17 ) | 0.87 (-2.30 to 4.14 ) | 0.596 | 5 (02 to 10 ) | 5 (1 to 18 ) | 0.93 (-2.21 to 4.18 ) | 0.566 |
| Hami | 8 (4 to 14 ) | 7 (2 to 20 ) | -0.19 (-4.89 to 4.74 ) | 0.938 | 7 (4 to 13 ) | 6 (2 to 18 ) | -0.29 (-5.03 to 4.68 ) | 0.906 | 7 (4 to 13 ) | 7 (2 to 19 ) | -0.24 (-4.95 to 4.71 ) | 0.924 |
| Ili Kazakh | 9 (2 to 21 ) | 5 (1 to 17 ) | -3.37 (-4.35 to -2.38 ) | <.001 | 8 (2 to 19 ) | 4 (1 to 15 ) | -3.49 (-4.46 to -2.52 ) | <.001 | 9 (02 to 20 ) | 4 (1 to 16 ) | -3.42 (-4.44 to -2.39 ) | <.001 |
| Karamay | 6 (2 to 14 ) | 5 (1 to 17 ) | -0.92 (-3.29 to 1.50 ) | 0.452 | 5 (2 to 13 ) | 4 (1 to 16 ) | -1.04 (-3.39 to 1.38 ) | 0.397 | 6 (02 to 13 ) | 5 (1 to 17 ) | -0.97 (-3.33 to 1.44 ) | 0.426 |
| Kashgar | 10 (2 to 27 ) | 4 (0 to 14 ) | -5.08 (-7.47 to -2.63 ) | <.001 | 9 (2 to 25 ) | 3 (0 to 13 ) | -5.15 (-7.38 to -2.87 ) | <.001 | 10 (02 to 26 ) | 4 (0 to 14 ) | -5.11 (-7.43 to -2.74 ) | <.001 |
| Khotan | 11 (2 to 28 ) | 8 (1 to 26 ) | -1.87 (-4.41 to 0.74 ) | 0.159 | 10 (2 to 26 ) | 7 (1 to 24 ) | -2.22 (-4.56 to 0.17 ) | 0.069 | 11 (02 to 27 ) | 7 (1 to 25 ) | -2.16 (-4.50 to 0.24 ) | 0.077 |
| Kizilsu Kirghiz | 9 (1 to 26 ) | 3 (0 to 11 ) | -5.71 (-9.54 to -1.72 ) | 0.005 | 9 (1 to 24 ) | 3 (0 to 10 ) | -5.94 (-8.57 to -3.24 ) | <.001 | 9 (01 to 25 ) | 3 (0 to 11 ) | -5.90 (-8.58 to -3.15 ) | <.001 |
| Shihezi | 4 (1 to 10 ) | 3 (1 to 13 ) | -0.75 (-3.20 to 1.76 ) | 0.555 | 4 (1 to 9 ) | 3 (0 to 12 ) | -0.80 (-2.77 to 1.21 ) | 0.432 | 4 (01 to 9 ) | 3 (1 to 12 ) | -0.73 (-2.70 to 1.28 ) | 0.472 |
| Tacheng | 7 (2 to 16 ) | 5 (1 to 19 ) | -1.00 (-3.04 to 1.08 ) | 0.342 | 6 (2 to 14 ) | 5 (1 to 17 ) | -1.12 (-3.16 to 0.97 ) | 0.293 | 6 (02 to 15 ) | 5 (1 to 18 ) | -1.05 (-3.09 to 1.03 ) | 0.319 |
| Turfan | 7 (3 to 14 ) | 8 (2 to 25 ) | 1.03 (-2.89 to 5.11 ) | 0.611 | 6 (2 to 12 ) | 7 (2 to 22 ) | 0.93 (-3.03 to 5.05 ) | 0.651 | 6 (02 to 13 ) | 7 (2 to 23 ) | 0.98 (-2.95 to 5.08 ) | 0.629 |
| rümqi | 4 (1 to 8 ) | 4 (1 to 14 ) | 0.51 (-1.97 to 3.05 ) | 0.688 | 3 (1 to 8 ) | 4 (1 to 13 ) | 0.58 (-2.39 to 3.65 ) | 0.704 | 3 (01 to 8 ) | 4 (1 to 14 ) | 0.50 (-2.05 to 3.12 ) | 0.703 |
| Xizang |  |  |  |  |  |  |  |  |  |  |  |  |
| Chamdo | 12 (5 to 22 ) | 7 (1 to 22 ) | -3.02 (-3.67 to -2.37 ) | <.001 | 11 (4 to 20 ) | 6 (1 to 20 ) | -3.10 (-3.80 to -2.39 ) | <.001 | 12 (4 to 21 ) | 7 (1 to 21 ) | -3.06 (-3.73 to -2.38 ) | <.001 |
| Lhasa | 13 (4 to 25 ) | 9 (2 to 32 ) | -1.52 (-1.89 to -1.15 ) | <.001 | 12 (3 to 23 ) | 9 (1 to 29 ) | -1.75 (-2.32 to -1.18 ) | <.001 | 12 (4 to 24 ) | 9 (1 to 31 ) | -1.56 (-1.92 to -1.19 ) | <.001 |
| Nagchu | 14 (5 to 26 ) | 11 (2 to 32 ) | -1.65 (-2.37 to -0.93 ) | <.001 | 13 (4 to 24 ) | 10 (2 to 29 ) | -1.68 (-2.29 to -1.06 ) | <.001 | 14 (5 to 25 ) | 10 (2 to 31 ) | -1.64 (-2.38 to -0.91 ) | <.001 |
| Ngari | 11 (2 to 25 ) | 8 (1 to 30 ) | -1.39 (-3.48 to 0.74 ) | 0.198 | 10 (2 to 23 ) | 8 (1 to 27 ) | -1.66 (-3.32 to 0.04 ) | 0.055 | 10 (02 to 24 ) | 8 (1 to 29 ) | -1.44 (-3.52 to 0.68 ) | 0.180 |
| Nyingtri | 11 (4 to 22 ) | 8 (2 to 25 ) | -1.54 (-1.90 to -1.19 ) | <.001 | 10 (3 to 20 ) | 8 (2 to 23 ) | -1.73 (-2.18 to -1.28 ) | <.001 | 11 (3 to 21 ) | 8 (2 to 24 ) | -1.67 (-2.13 to -1.21 ) | <.001 |
| Shannan | 12 (4 to 24 ) | 10 (2 to 35 ) | -0.88 (-1.15 to -0.61 ) | <.001 | 11 (3 to 22 ) | 9 (1 to 31 ) | -0.98 (-1.35 to -0.60 ) | <.001 | 11 (3 to 23 ) | 10 (2 to 33 ) | -0.91 (-1.25 to -0.58 ) | <.001 |
| Shigatse | 13 (4 to 27 ) | 10 (2 to 33 ) | -1.70 (-2.44 to -0.96 ) | <.001 | 12 (3 to 25 ) | 9 (1 to 30 ) | -1.76 (-2.28 to -1.22 ) | <.001 | 13 (4 to 26 ) | 9 (1 to 31 ) | -1.74 (-2.45 to -1.02 ) | <.001 |
| Yunnan |  |  |  |  |  |  |  |  |  |  |  |  |
| Baoshan | 12 (4 to 27 ) | 6 (1 to 19 ) | -3.23 (-4.49 to -1.96 ) | <.001 | 11 (4 to 25 ) | 6 (1 to 17 ) | -3.26 (-4.66 to -1.85 ) | <.001 | 11 (4 to 26 ) | 6 (1 to 18 ) | -3.20 (-4.31 to -2.07 ) | <.001 |
| Chuxiong Yi | 9 (4 to 17 ) | 3 (1 to 8 ) | -5.55 (-6.90 to -4.18 ) | <.001 | 8 (4 to 15 ) | 3 (1 to 7 ) | -5.62 (-7.10 to -4.11 ) | <.001 | 9 (4 to 16 ) | 3 (1 to 8 ) | -5.60 (-6.97 to -4.21 ) | <.001 |
| Dali Bai | 11 (4 to 22 ) | 4 (1 to 11 ) | -5.14 (-6.22 to -4.06 ) | <.001 | 10 (4 to 20 ) | 4 (1 to 10 ) | -5.25 (-6.11 to -4.37 ) | <.001 | 10 (4 to 21 ) | 4 (1 to 10 ) | -5.17 (-6.22 to -4.12 ) | <.001 |
| Dehong Dai and Jingpo | 13 (4 to 28 ) | 8 (2 to 24 ) | -2.55 (-4.17 to -0.90 ) | 0.003 | 12 (4 to 26 ) | 7 (1 to 22 ) | -2.40 (-3.83 to -0.95 ) | 0.001 | 12 (4 to 27 ) | 8 (1 to 23 ) | -2.42 (-3.72 to -1.11 ) | <.001 |
| Dêqên Tibetan | 17 (7 to 33 ) | 6 (1 to 18 ) | -5.43 (-7.47 to -3.34 ) | <.001 | 15 (6 to 30 ) | 6 (1 to 16 ) | -5.47 (-7.58 to -3.31 ) | <.001 | 16 (6 to 32 ) | 6 (1 to 17 ) | -5.47 (-7.49 to -3.40 ) | <.001 |
| Honghe Hani and Yi | 13 (7 to 22 ) | 6 (1 to 16 ) | -4.25 (-5.02 to -3.48 ) | <.001 | 12 (6 to 21 ) | 5 (1 to 15 ) | -4.33 (-4.94 to -3.72 ) | <.001 | 13 (6 to 22 ) | 6 (1 to 16 ) | -4.28 (-4.91 to -3.64 ) | <.001 |
| Kunming | 8 (4 to 13 ) | 2 (0 to 5 ) | -6.85 (-7.97 to -5.71 ) | <.001 | 7 (4 to 12 ) | 2 (0 to 5 ) | -6.97 (-8.81 to -5.09 ) | <.001 | 7 (4 to 13 ) | 2 (0 to 5 ) | -6.87 (-8.0 to -5.71 ) | <.001 |
| Lijiang | 13 (6 to 24 ) | 4 (1 to 10 ) | -6.43 (-7.16 to -5.70 ) | <.001 | 12 (5 to 22 ) | 3 (1 to 9 ) | -6.54 (-7.25 to -5.83 ) | <.001 | 12 (5 to 23 ) | 3 (1 to 10 ) | -6.48 (-7.20 to -5.76 ) | <.001 |
| Lincang | 13 (5 to 26 ) | 8 (2 to 23 ) | -2.53 (-3.19 to -1.87 ) | <.001 | 12 (4 to 24 ) | 7 (2 to 21 ) | -2.64 (-3.26 to -2.01 ) | <.001 | 12 (5 to 25 ) | 8 (2 to 22 ) | -2.46 (-3.18 to -1.74 ) | <.001 |
| Nujiang Lisu | 16 (6 to 33 ) | 7 (2 to 21 ) | -4.27 (-6.12 to -2.39 ) | <.001 | 15 (6 to 31 ) | 7 (1 to 19 ) | -4.20 (-6.15 to -2.21 ) | <.001 | 15 (6 to 32 ) | 7 (1 to 20 ) | -4.32 (-6.18 to -2.43 ) | <.001 |
| Pu'er | 14 (6 to 27 ) | 10 (2 to 28 ) | -1.99 (-2.83 to -1.14 ) | <.001 | 13 (5 to 25 ) | 9 (2 to 25 ) | -2.07 (-2.82 to -1.32 ) | <.001 | 14 (5 to 26 ) | 9 (2 to 27 ) | -2.10 (-2.66 to -1.54 ) | <.001 |
| Qujing | 13 (9 to 20 ) | 3 (1 to 7 ) | -8.12 (-9.85 to -6.34 ) | <.001 | 12 (8 to 18 ) | 3 (1 to 7 ) | -7.97 (-8.93 to -7.00 ) | <.001 | 13 (8 to 19 ) | 3 (1 to 7 ) | -7.97 (-9.51 to -6.41 ) | <.001 |
| Wenshan Zhuang and Miao | 16 (10 to 23 ) | 5 (1 to 14 ) | -6.13 (-7.27 to -4.98 ) | <.001 | 14 (9 to 22 ) | 4 (1 to 13 ) | -6.22 (-7.34 to -5.08 ) | <.001 | 15 (10 to 23 ) | 5 (1 to 13 ) | -6.17 (-7.30 to -5.03 ) | <.001 |
| Xishuangbanna Dai | 17 (6 to 33 ) | 14 (3 to 41 ) | -1.07 (-2.42 to 0.31 ) | 0.127 | 16 (6 to 31 ) | 13 (2 to 37 ) | -1.26 (-2.33 to -0.19 ) | 0.022 | 17 (6 to 32 ) | 13 (3 to 39 ) | -1.29 (-2.18 to -0.39 ) | 0.005 |
| Yuxi | 10 (5 to 17 ) | 3 (1 to 10 ) | -5.33 (-6.89 to -3.74 ) | <.001 | 9 (4 to 16 ) | 3 (1 to 9 ) | -5.59 (-6.52 to -4.64 ) | <.001 | 9 (4 to 17 ) | 3 (1 to 9 ) | -5.36 (-6.77 to -3.93 ) | <.001 |
| Zhaotong | 14 (9 to 20 ) | 2 (1 to 7 ) | -8.59 (-9.68 to -7.49 ) | <.001 | 12 (8 to 19 ) | 2 (1 to 6 ) | -8.69 (-9.76 to -7.61 ) | <.001 | 13 (8 to 19 ) | 2 (1 to 6 ) | -8.63 (-9.71 to -7.54 ) | <.001 |
| Zhejiang |  |  |  |  |  |  |  |  |  |  |  |  |
| Hangzhou | 3 (2 to 6 ) | 2 (0 to 6 ) | -2.82 (-3.60 to -2.04 ) | <.001 | 3 (1 to 5 ) | 2 (0 to 5 ) | -2.88 (-3.68 to -2.07 ) | <.001 | 3 (01 to 5 ) | 2 (0 to 5 ) | -2.86 (-3.55 to -2.16 ) | <.001 |
| Huzhou | 3 (2 to 5 ) | 2 (1 to 6 ) | -1.86 (-3.26 to -0.43 ) | 0.011 | 3 (1 to 5 ) | 2 (0 to 6 ) | -1.92 (-3.40 to -0.41 ) | 0.013 | 3 (01 to 5 ) | 2 (0 to 6 ) | -1.90 (-3.24 to -0.53 ) | 0.007 |
| Jiaxing | 3 (1 to 5 ) | 2 (0 to 5 ) | -2.93 (-4.29 to -1.55 ) | <.001 | 3 (1 to 5 ) | 1 (0 to 5 ) | -3.03 (-4.23 to -1.81 ) | <.001 | 3 (01 to 5 ) | 2 (0 to 5 ) | -2.98 (-4.27 to -1.67 ) | <.001 |
| Jinhua | 4 (2 to 8 ) | 2 (1 to 7 ) | -3.64 (-4.14 to -3.14 ) | <.001 | 4 (2 to 7 ) | 2 (0 to 6 ) | -3.79 (-4.37 to -3.21 ) | <.001 | 4 (02 to 8 ) | 2 (0 to 6 ) | -3.69 (-4.16 to -3.22 ) | <.001 |
| Lishui | 6 (2 to 10 ) | 3 (1 to 9 ) | -3.92 (-4.71 to -3.13 ) | <.001 | 5 (2 to 10 ) | 2 (1 to 8 ) | -3.92 (-4.39 to -3.45 ) | <.001 | 5 (02 to 10 ) | 3 (1 to 8 ) | -3.91 (-4.33 to -3.49 ) | <.001 |
| Ningbo | 4 (1 to 7 ) | 2 (0 to 6 ) | -4.06 (-5.33 to -2.78 ) | <.001 | 3 (1 to 7 ) | 2 (0 to 5 ) | -4.16 (-5.34 to -2.97 ) | <.001 | 3 (01 to 7 ) | 2 (0 to 5 ) | -4.11 (-5.34 to -2.86 ) | <.001 |
| Quzhou | 5 (2 to 9 ) | 3 (1 to 9 ) | -3.21 (-4.01 to -2.41 ) | <.001 | 5 (2 to 8 ) | 3 (1 to 8 ) | -3.22 (-4.13 to -2.30 ) | <.001 | 5 (02 to 9 ) | 3 (1 to 9 ) | -3.22 (-4.02 to -2.40 ) | <.001 |
| Shaoxing | 4 (2 to 7 ) | 2 (0 to 6 ) | -3.27 (-3.65 to -2.89 ) | <.001 | 3 (1 to 6 ) | 2 (0 to 6 ) | -3.37 (-3.77 to -2.96 ) | <.001 | 3 (02 to 6 ) | 2 (0 to 6 ) | -3.33 (-3.73 to -2.92 ) | <.001 |
| Taizhou | 5 (2 to 10 ) | 2 (0 to 6 ) | -4.87 (-5.94 to -3.78 ) | <.001 | 5 (2 to 9 ) | 2 (0 to 6 ) | -5.01 (-5.51 to -4.52 ) | <.001 | 5 (02 to 10 ) | 2 (0 to 6 ) | -4.99 (-5.72 to -4.25 ) | <.001 |
| Wenzhou | 4 (2 to 9 ) | 2 (0 to 5 ) | -5.08 (-5.80 to -4.36 ) | <.001 | 4 (1 to 8 ) | 1 (0 to 5 ) | -5.10 (-5.86 to -4.32 ) | <.001 | 4 (01 to 8 ) | 1 (0 to 5 ) | -5.10 (-5.83 to -4.37 ) | <.001 |
| Zhoushan | 3 (1 to 7 ) | 2 (0 to 6 ) | -3.44 (-5.04 to -1.81 ) | <.001 | 3 (1 to 6 ) | 2 (0 to 5 ) | -3.54 (-5.07 to -2.00 ) | <.001 | 3 (01 to 7 ) | 2 (0 to 6 ) | -3.49 (-5.06 to -1.89 ) | <.001 |
| Hong Kong |  |  |  |  |  |  |  |  |  |  |  |  |
| Central and Western | 3 (1 to 6 ) | 3 (1 to 9 ) | 0.28 (-1.61 to 2.20 ) | 0.772 | 3 (1 to 6 ) | 3 (1 to 8 ) | 0.18 (-1.77 to 2.17 ) | 0.859 | 3 (01 to 6 ) | 3 (1 to 8 ) | 0.27 (-1.95 to 2.54 ) | 0.812 |
| Eastern | 3 (1 to 5 ) | 2 (0 to 5 ) | -1.34 (-2.44 to -0.22 ) | 0.019 | 2 (1 to 5 ) | 2 (0 to 5 ) | -1.37 (-2.71 to -0.01 ) | 0.048 | 02 (01 to 5 ) | 2 (0 to 5 ) | -1.30 (-2.38 to -0.20 ) | 0.020 |
| Islands | 4 (2 to 8 ) | 4 (1 to 11 ) | 0.03 (-0.47 to 0.54 ) | 0.903 | 4 (2 to 7 ) | 4 (1 to 10 ) | 0.06 (-0.65 to 0.77 ) | 0.876 | 4 (02 to 7 ) | 4 (1 to 10 ) | 0.09 (-0.60 to 0.78 ) | 0.803 |
| Kowloon City | 1 (0 to 2 ) | 1 (0 to 2 ) | -2.58 (-3.80 to -1.34 ) | <.001 | 1 (0 to 2 ) | 1 (0 to 2 ) | -2.73 (-4.50 to -0.92 ) | 0.003 | 01 (0 to 02 ) | 1 (0 to 2 ) | -2.67 (-4.49 to -0.82 ) | 0.005 |
| Kwai Tsing | 2 (1 to 4 ) | 2 (0 to 6 ) | 0.40 (-1.50 to 2.34 ) | 0.680 | 2 (1 to 4 ) | 2 (0 to 5 ) | 0.35 (-0.79 to 1.51 ) | 0.548 | 02 (01 to 4 ) | 2 (0 to 5 ) | 0.35 (-1.56 to 2.31 ) | 0.719 |
| Kwun Tong | 1 (1 to 2 ) | 1 (0 to 2 ) | -3.99 (-5.39 to -2.56 ) | <.001 | 1 (0 to 2 ) | 0 (0 to 1 ) | -4.02 (-5.65 to -2.36 ) | <.001 | 01 (0 to 02 ) | 1 (0 to 1 ) | -3.96 (-5.68 to -2.22 ) | <.001 |
| North | 4 (2 to 8 ) | 3 (1 to 8 ) | -1.83 (-2.58 to -1.06 ) | <.001 | 4 (2 to 8 ) | 3 (1 to 7 ) | -1.85 (-2.76 to -0.93 ) | <.001 | 4 (02 to 8 ) | 3 (1 to 8 ) | -1.86 (-2.82 to -0.89 ) | <.001 |
| Sai Kung | 3 (1 to 5 ) | 2 (0 to 5 ) | -2.27 (-3.86 to -0.65 ) | 0.006 | 3 (1 to 5 ) | 2 (0 to 4 ) | -2.21 (-3.18 to -1.23 ) | <.001 | 3 (01 to 5 ) | 2 (0 to 5 ) | -2.21 (-3.40 to -1.0 ) | <.001 |
| Sha Tin | 3 (1 to 6 ) | 3 (1 to 7 ) | -0.68 (-1.28 to -0.07 ) | 0.028 | 3 (1 to 5 ) | 2 (0 to 6 ) | -0.88 (-1.43 to -0.32 ) | 0.002 | 3 (01 to 6 ) | 2 (0 to 7 ) | -0.79 (-1.32 to -0.26 ) | 0.003 |
| Sham Shui Po | 2 (1 to 4 ) | 2 (0 to 5 ) | 0.31 (-1.09 to 1.74 ) | 0.665 | 2 (1 to 3 ) | 2 (0 to 5 ) | 0.30 (-0.61 to 1.23 ) | 0.517 | 02 (01 to 4 ) | 2 (0 to 5 ) | 0.24 (-0.79 to 1.28 ) | 0.650 |
| Southern | 3 (1 to 6 ) | 3 (1 to 8 ) | 0.02 (-1.20 to 1.25 ) | 0.980 | 3 (1 to 6 ) | 3 (1 to 7 ) | 0.02 (-1.21 to 1.27 ) | 0.971 | 3 (01 to 6 ) | 3 (1 to 8 ) | -0.06 (-1.24 to 1.14 ) | 0.921 |
| Tai Po | 4 (2 to 8 ) | 4 (1 to 9 ) | -1.09 (-1.90 to -0.28 ) | 0.008 | 4 (2 to 8 ) | 3 (1 to 8 ) | -1.24 (-2.61 to 0.15 ) | 0.081 | 4 (02 to 8 ) | 3 (1 to 9 ) | -1.14 (-2.03 to -0.24 ) | 0.013 |
| Tsuen Wan | 3 (2 to 7 ) | 4 (1 to 10 ) | 0.29 (-0.11 to 0.70 ) | 0.150 | 3 (1 to 6 ) | 3 (1 to 9 ) | 0.22 (-0.19 to 0.64 ) | 0.293 | 3 (02 to 6 ) | 3 (1 to 9 ) | 0.18 (-0.28 to 0.64 ) | 0.435 |
| Tuen Mun | 3 (1 to 5 ) | 2 (0 to 6 ) | -0.59 (-1.65 to 0.47 ) | 0.274 | 2 (1 to 4 ) | 2 (0 to 5 ) | -0.69 (-1.38 to 0.01 ) | 0.052 | 02 (01 to 5 ) | 2 (0 to 6 ) | -0.60 (-1.66 to 0.46 ) | 0.265 |
| Wan Chai | 3 (1 to 6 ) | 2 (0 to 6 ) | -1.44 (-3.40 to 0.56 ) | 0.158 | 3 (1 to 5 ) | 2 (0 to 5 ) | -1.52 (-3.45 to 0.44 ) | 0.127 | 3 (01 to 6 ) | 2 (0 to 5 ) | -1.50 (-3.23 to 0.25 ) | 0.093 |
| Wong Tai Sin | 2 (1 to 4 ) | 2 (0 to 5 ) | -0.71 (-2.55 to 1.16 ) | 0.453 | 2 (1 to 4 ) | 2 (0 to 4 ) | -0.79 (-2.60 to 1.05 ) | 0.399 | 02 (01 to 4 ) | 2 (0 to 4 ) | -0.75 (-2.57 to 1.11 ) | 0.428 |
| Yau Tsim Mong | 1 (1 to 3 ) | 1 (0 to 4 ) | -0.38 (-1.77 to 1.04 ) | 0.599 | 1 (1 to 3 ) | 1 (0 to 3 ) | -0.30 (-0.96 to 0.36 ) | 0.369 | 01 (01 to 3 ) | 1 (0 to 3 ) | -0.37 (-1.09 to 0.36 ) | 0.324 |
| Yuen Long | 3 (2 to 6 ) | 2 (0 to 6 ) | -1.68 (-2.00 to -1.36 ) | <.001 | 3 (1 to 6 ) | 2 (0 to 6 ) | -1.83 (-2.00 to -1.66 ) | <.001 | 3 (02 to 6 ) | 2 (0 to 6 ) | -1.71 (-2.18 to -1.25 ) | <.001 |
| Macao |  |  |  |  |  |  |  |  |  |  |  |  |
| Ilhas | 6 (3 to 11 ) | 4 (1 to 12 ) | -1.90 (-4.78 to 1.08 ) | 0.209 | 6 (3 to 11 ) | 4 (1 to 11 ) | -2.27 (-4.14 to -0.36 ) | 0.020 | 6 (3 to 11 ) | 4 (1 to 11 ) | -1.91 (-5.46 to 1.76 ) | 0.304 |
| Macau | 4 (2 to 8 ) | 3 (1 to 9 ) | -1.17 (-3.38 to 1.09 ) | 0.307 | 4 (2 to 8 ) | 3 (1 to 9 ) | -0.98 (-2.51 to 0.57 ) | 0.213 | 4 (02 to 8 ) | 3 (1 to 9 ) | -0.88 (-2.29 to 0.55 ) | 0.226 |
| *The data in Neijiang, Sichuan Province cannot be presented due to the issue of data. | | | | | | | | | | | | |
