# Supplementary material for: Temporal Trends in the Prevalence of Child Undernutrition in China From 2000 to 2019, With Projections of Prevalence in 2030: Cross-Sectional Analysis
Source: JMIR Public Health Surveill. 2024 Oct 9;10:e58564. doi: 10.2196/58564 (PMC11499720; doi:10.2196/58564)
Supplement: Multimedia Appendix 6 [file publichealth_v10i1e58564_app6.docx]

**Multimedia Appendix 6.** Prevalence and trends of child growth failure in 5 age groups by sex in China from 2000 to 2019.

|  |  | Mean percent | | | | | | | | | |
| --- | --- | --- | --- | --- | --- | --- | --- | --- | --- | --- | --- |
|  |  | Early Neonatal | | Late Neonatal | | Post Neonatal | | 1 to 4 | | under 5 | |
| **Location** |  | Boys | Girls | Boys | Girls | Boys | Girls | Boys | Girls | Boys | Girls |
|  |  | **Stunting** | | | | | | | | | |
| China* | 2000 | 14 | 15 | 14 | 15 | 15 | 15 | 20 | 19 | 19 | 18 |
|  | 2019 | 9 | 10 | 9 | 10 | 10 | 10 | 12 | 11 | 12 | 11 |
|  | AAPC | -2.48 (<.001 ) | -2.25 (<.001 ) | -2.49 (<.001 ) | -2.22 (<.001 ) | -2.14 (<.001 ) | -2.14 (<.001 ) | -2.50 (<.001 ) | -2.70 (<.001 ) | -2.44 (<.001 ) | -2.53 (<.001 ) |
| China |  |  |  |  |  |  |  |  |  |  |  |
| Anhui | 2000 | 13 | 15 | 13 | 14 | 15 | 15 | 19 | 18 | 18 | 17 |
|  | 2019 | 9 | 10 | 9 | 10 | 10 | 10 | 13 | 11 | 12 | 12 |
|  | AAPC | -2.08 (<.001 ) | -1.85 (<.001 ) | -2.10 (<.001 ) | -1.83 (<.001 ) | -1.74 (<.001 ) | -1.74 (<.001 ) | -2.10 (<.001 ) | -2.30 (<.001 ) | -2.04 (<.001 ) | -2.13 (<.001 ) |
| Beijing | 2000 | 7 | 7 | 7 | 7 | 7 | 7 | 10 | 9 | 9 | 9 |
|  | 2019 | 6 | 6 | 6 | 6 | 7 | 7 | 8 | 7 | 8 | 7 |
|  | AAPC | -0.99 (<.001 ) | -0.76 (<.001 ) | -1.01 (<.001 ) | -0.73 (<.001 ) | -0.65 (<.001 ) | -0.65 (<.001 ) | -0.98 (<.001 ) | -1.18 (<.001 ) | -0.95 (<.001 ) | -0.99 (<.001 ) |
| Chongqing | 2000 | 13 | 14 | 13 | 14 | 14 | 14 | 18 | 17 | 17 | 17 |
|  | 2019 | 8 | 9 | 8 | 9 | 9 | 9 | 11 | 10 | 11 | 11 |
|  | AAPC | -2.40 (<.001 ) | -2.17 (<.001 ) | -2.42 (<.001 ) | -2.15 (<.001 ) | -2.06 (<.001 ) | -2.06 (<.001 ) | -2.42 (<.001 ) | -2.63 (<.001 ) | -2.36 (<.001 ) | -2.45 (<.001 ) |
| Fujian | 2000 | 11 | 12 | 11 | 12 | 12 | 12 | 16 | 15 | 15 | 14 |
|  | 2019 | 7 | 8 | 7 | 8 | 8 | 8 | 10 | 9 | 10 | 9 |
|  | AAPC | -2.14 (<.001 ) | -1.91 (<.001 ) | -2.16 (<.001 ) | -1.89 (<.001 ) | -1.80 (<.001 ) | -1.80 (<.001 ) | -2.26 (<.001 ) | -2.38 (<.001 ) | -2.10 (<.001 ) | -2.26 (<.001 ) |
| Gansu | 2000 | 16 | 18 | 16 | 18 | 18 | 18 | 23 | 22 | 22 | 21 |
|  | 2019 | 11 | 12 | 11 | 12 | 12 | 12 | 15 | 14 | 14 | 14 |
|  | AAPC | -2.20 (<.001 ) | -1.97 (<.001 ) | -2.21 (<.001 ) | -1.94 (<.001 ) | -1.86 (<.001 ) | -1.86 (<.001 ) | -2.22 (<.001 ) | -2.42 (<.001 ) | -2.16 (<.001 ) | -2.25 (<.001 ) |
| Guangdong | 2000 | 11 | 11 | 10 | 11 | 11 | 11 | 15 | 14 | 14 | 14 |
|  | 2019 | 7 | 8 | 7 | 8 | 8 | 8 | 10 | 9 | 10 | 9 |
|  | AAPC | -2.08 (<.001 ) | -1.85 (<.001 ) | -2.10 (<.001 ) | -1.82 (<.001 ) | -1.74 (<.001 ) | -1.74 (<.001 ) | -2.10 (<.001 ) | -2.30 (<.001 ) | -2.04 (<.001 ) | -2.13 (<.001 ) |
| Guangxi | 2000 | 19 | 21 | 19 | 21 | 21 | 21 | 28 | 26 | 26 | 25 |
|  | 2019 | 13 | 14 | 12 | 14 | 15 | 15 | 18 | 16 | 17 | 16 |
|  | AAPC | -2.28 (<.001 ) | -2.05 (<.001 ) | -2.29 (<.001 ) | -2.02 (<.001 ) | -1.94 (<.001 ) | -1.94 (<.001 ) | -2.30 (<.001 ) | -2.50 (<.001 ) | -2.24 (<.001 ) | -2.33 (<.001 ) |
| Guizhou | 2000 | 29 | 31 | 29 | 31 | 31 | 31 | 41 | 39 | 39 | 38 |
|  | 2019 | 19 | 22 | 19 | 22 | 23 | 23 | 27 | 25 | 26 | 25 |
|  | AAPC | -2.10 (<.001 ) | -1.87 (<.001 ) | -2.12 (<.001 ) | -1.84 (<.001 ) | -1.76 (<.001 ) | -1.76 (<.001 ) | -2.12 (<.001 ) | -2.32 (<.001 ) | -2.06 (<.001 ) | -2.15 (<.001 ) |
| Hainan | 2000 | 13 | 14 | 13 | 14 | 14 | 14 | 19 | 17 | 18 | 17 |
|  | 2019 | 9 | 10 | 9 | 10 | 10 | 10 | 12 | 11 | 12 | 11 |
|  | AAPC | -2.03 (<.001 ) | -1.80 (<.001 ) | -2.05 (<.001 ) | -1.78 (<.001 ) | -1.69 (<.001 ) | -1.69 (<.001 ) | -2.06 (<.001 ) | -2.26 (<.001 ) | -1.99 (<.001 ) | -2.09 (<.001 ) |
| Hebei | 2000 | 12 | 13 | 12 | 13 | 13 | 13 | 17 | 16 | 16 | 15 |
|  | 2019 | 8 | 9 | 8 | 9 | 10 | 10 | 12 | 11 | 11 | 11 |
|  | AAPC | -1.79 (<.001 ) | -1.56 (<.001 ) | -1.80 (<.001 ) | -1.53 (<.001 ) | -1.45 (<.001 ) | -1.45 (<.001 ) | -1.81 (<.001 ) | -2.01 (<.001 ) | -1.75 (<.001 ) | -1.84 (<.001 ) |
| Heilongjiang | 2000 | 11 | 12 | 11 | 12 | 12 | 12 | 15 | 15 | 15 | 14 |
|  | 2019 | 5 | 6 | 5 | 6 | 6 | 6 | 7 | 6 | 7 | 7 |
|  | AAPC | -3.96 (<.001 ) | -3.77 (<.001 ) | -3.98 (<.001 ) | -3.77 (<.001 ) | -3.66 (<.001 ) | -3.66 (<.001 ) | -4.04 (<.001 ) | -4.23 (<.001 ) | -3.96 (<.001 ) | -4.06 (<.001 ) |
| Henan | 2000 | 17 | 19 | 17 | 19 | 19 | 19 | 24 | 23 | 23 | 23 |
|  | 2019 | 12 | 13 | 12 | 13 | 14 | 14 | 17 | 15 | 16 | 15 |
|  | AAPC | -2.01 (<.001 ) | -1.78 (<.001 ) | -2.03 (<.001 ) | -1.76 (<.001 ) | -1.67 (<.001 ) | -1.67 (<.001 ) | -2.04 (<.001 ) | -2.24 (<.001 ) | -1.97 (<.001 ) | -2.06 (<.001 ) |
| Hubei | 2000 | 16 | 17 | 15 | 17 | 17 | 17 | 22 | 21 | 21 | 20 |
|  | 2019 | 11 | 12 | 11 | 12 | 13 | 13 | 15 | 14 | 15 | 14 |
|  | AAPC | -1.89 (<.001 ) | -1.66 (<.001 ) | -1.90 (<.001 ) | -1.63 (<.001 ) | -1.55 (<.001 ) | -1.55 (<.001 ) | -2.01 (<.001 ) | -2.20 (<.001 ) | -1.85 (<.001 ) | -2.01 (<.001 ) |
| Hunan | 2000 | 20 | 22 | 20 | 22 | 22 | 22 | 29 | 27 | 28 | 27 |
|  | 2019 | 15 | 17 | 15 | 17 | 17 | 17 | 21 | 19 | 20 | 19 |
|  | AAPC | -1.64 (<.001 ) | -1.41 (<.001 ) | -1.66 (<.001 ) | -1.38 (<.001 ) | -1.30 (<.001 ) | -1.30 (<.001 ) | -1.66 (<.001 ) | -1.86 (<.001 ) | -1.60 (<.001 ) | -1.69 (<.001 ) |
| Jiangsu | 2000 | 10 | 11 | 10 | 11 | 11 | 11 | 14 | 13 | 13 | 13 |
|  | 2019 | 7 | 8 | 7 | 8 | 8 | 8 | 9 | 8 | 9 | 9 |
|  | AAPC | -2.12 (<.001 ) | -1.89 (<.001 ) | -2.14 (<.001 ) | -1.87 (<.001 ) | -1.78 (<.001 ) | -1.78 (<.001 ) | -2.15 (<.001 ) | -2.43 (<.001 ) | -2.08 (<.001 ) | -2.25 (<.001 ) |
| Jiangxi | 2000 | 15 | 17 | 15 | 17 | 17 | 17 | 22 | 20 | 21 | 20 |
|  | 2019 | 9 | 11 | 9 | 11 | 11 | 11 | 13 | 12 | 13 | 12 |
|  | AAPC | -2.51 (<.001 ) | -2.28 (<.001 ) | -2.53 (<.001 ) | -2.26 (<.001 ) | -2.17 (<.001 ) | -2.17 (<.001 ) | -2.53 (<.001 ) | -2.73 (<.001 ) | -2.47 (<.001 ) | -2.56 (<.001 ) |
| Jilin | 2000 | 11 | 12 | 11 | 12 | 12 | 12 | 16 | 15 | 15 | 14 |
|  | 2019 | 8 | 9 | 8 | 9 | 9 | 9 | 11 | 10 | 10 | 10 |
|  | AAPC | -1.90 (<.001 ) | -1.66 (<.001 ) | -1.91 (<.001 ) | -1.64 (<.001 ) | -1.60 (<.001 ) | -1.60 (<.001 ) | -1.92 (<.001 ) | -2.13 (<.001 ) | -1.85 (<.001 ) | -1.94 (<.001 ) |
| Liaoning | 2000 | 8 | 8 | 8 | 8 | 8 | 8 | 11 | 10 | 10 | 10 |
|  | 2019 | 5 | 6 | 5 | 6 | 6 | 6 | 7 | 7 | 7 | 7 |
|  | AAPC | -2.02 (<.001 ) | -1.76 (<.001 ) | -2.02 (<.001 ) | -1.74 (<.001 ) | -1.65 (<.001 ) | -1.65 (<.001 ) | -2.04 (<.001 ) | -2.23 (<.001 ) | -2.01 (<.001 ) | -2.04 (<.001 ) |
| Inner Mongolia | 2000 | 10 | 11 | 10 | 11 | 11 | 11 | 15 | 14 | 14 | 14 |
|  | 2019 | 4 | 5 | 4 | 5 | 5 | 5 | 6 | 5 | 6 | 5 |
|  | AAPC | -4.96 (<.001 ) | -4.66 (<.001 ) | -4.98 (<.001 ) | -4.64 (<.001 ) | -4.55 (<.001 ) | -4.55 (<.001 ) | -4.97 (<.001 ) | -5.17 (<.001 ) | -4.84 (<.001 ) | -4.99 (<.001 ) |
| Ningxia | 2000 | 10 | 11 | 10 | 11 | 11 | 11 | 14 | 13 | 13 | 13 |
|  | 2019 | 4 | 4 | 4 | 4 | 4 | 4 | 5 | 5 | 5 | 5 |
|  | AAPC | -5.16 (<.001 ) | -4.89 (<.001 ) | -5.18 (<.001 ) | -4.86 (<.001 ) | -4.76 (<.001 ) | -4.76 (<.001 ) | -5.10 (<.001 ) | -5.30 (<.001 ) | -5.04 (<.001 ) | -5.13 (<.001 ) |
| Qinghai | 2000 | 17 | 18 | 17 | 18 | 18 | 18 | 24 | 22 | 23 | 22 |
|  | 2019 | 11 | 13 | 11 | 13 | 13 | 13 | 16 | 14 | 15 | 15 |
|  | AAPC | -2.13 (<.001 ) | -1.90 (<.001 ) | -2.15 (<.001 ) | -1.88 (<.001 ) | -1.79 (<.001 ) | -1.79 (<.001 ) | -2.16 (<.001 ) | -2.35 (<.001 ) | -2.09 (<.001 ) | -2.19 (<.001 ) |
| Shaanxi | 2000 | 13 | 14 | 12 | 14 | 14 | 14 | 18 | 17 | 17 | 16 |
|  | 2019 | 8 | 9 | 8 | 9 | 9 | 9 | 11 | 10 | 11 | 10 |
|  | AAPC | -2.33 (<.001 ) | -2.10 (<.001 ) | -2.35 (<.001 ) | -2.08 (<.001 ) | -2.00 (<.001 ) | -2.00 (<.001 ) | -2.35 (<.001 ) | -2.56 (<.001 ) | -2.29 (<.001 ) | -2.38 (<.001 ) |
| Shandong | 2000 | 11 | 12 | 11 | 12 | 12 | 12 | 15 | 14 | 14 | 14 |
|  | 2019 | 6 | 7 | 6 | 7 | 7 | 7 | 8 | 8 | 8 | 8 |
|  | AAPC | -3.06 (<.001 ) | -2.83 (<.001 ) | -3.07 (<.001 ) | -2.80 (<.001 ) | -2.72 (<.001 ) | -2.72 (<.001 ) | -3.27 (<.001 ) | -3.28 (<.001 ) | -3.02 (<.001 ) | -3.28 (<.001 ) |
| Shanghai | 2000 | 8 | 8 | 8 | 8 | 8 | 8 | 11 | 10 | 10 | 10 |
|  | 2019 | 7 | 7 | 6 | 7 | 8 | 8 | 9 | 8 | 9 | 8 |
|  | AAPC | -0.80 (<.001 ) | -0.57 (<.001 ) | -0.82 (<.001 ) | -0.54 (<.001 ) | -0.46 (<.001 ) | -0.46 (<.001 ) | -0.73 (<.001 ) | -0.92 (<.001 ) | -0.70 (<.001 ) | -0.74 (<.001 ) |
| Shanxi | 2000 | 13 | 14 | 13 | 14 | 14 | 14 | 18 | 17 | 17 | 17 |
|  | 2019 | 8 | 9 | 8 | 10 | 10 | 10 | 12 | 11 | 11 | 11 |
|  | AAPC | -2.19 (<.001 ) | -1.96 (<.001 ) | -2.21 (<.001 ) | -1.94 (<.001 ) | -1.85 (<.001 ) | -1.85 (<.001 ) | -2.26 (<.001 ) | -2.46 (<.001 ) | -2.15 (<.001 ) | -2.24 (<.001 ) |
| Sichuan | 2000 | 16 | 17 | 16 | 17 | 18 | 18 | 23 | 21 | 22 | 21 |
|  | 2019 | 6 | 7 | 6 | 7 | 7 | 7 | 9 | 8 | 9 | 8 |
|  | AAPC | -4.90 (<.001 ) | -4.67 (<.001 ) | -4.91 (<.001 ) | -4.65 (<.001 ) | -4.57 (<.001 ) | -4.57 (<.001 ) | -4.85 (<.001 ) | -5.03 (<.001 ) | -4.87 (<.001 ) | -4.88 (<.001 ) |
| Tianjin | 2000 | 8 | 9 | 8 | 9 | 9 | 9 | 12 | 11 | 11 | 11 |
|  | 2019 | 6 | 7 | 6 | 7 | 7 | 7 | 9 | 8 | 8 | 8 |
|  | AAPC | -1.67 (<.001 ) | -1.46 (<.001 ) | -1.69 (<.001 ) | -1.43 (<.001 ) | -1.35 (<.001 ) | -1.35 (<.001 ) | -1.71 (<.001 ) | -1.91 (<.001 ) | -1.68 (<.001 ) | -1.73 (<.001 ) |
| Xinjiang | 2000 | 15 | 16 | 15 | 16 | 16 | 16 | 21 | 20 | 20 | 19 |
|  | 2019 | 10 | 11 | 10 | 11 | 12 | 12 | 14 | 13 | 14 | 13 |
|  | AAPC | -2.05 (<.001 ) | -1.82 (<.001 ) | -2.07 (<.001 ) | -1.80 (<.001 ) | -1.71 (<.001 ) | -1.71 (<.001 ) | -2.06 (<.001 ) | -2.27 (<.001 ) | -2.00 (<.001 ) | -2.10 (<.001 ) |
| Xizang | 2000 | 20 | 22 | 20 | 22 | 22 | 22 | 29 | 27 | 27 | 26 |
|  | 2019 | 16 | 18 | 16 | 19 | 19 | 19 | 23 | 21 | 22 | 21 |
|  | AAPC | -1.13 (<.001 ) | -0.90 (<.001 ) | -1.15 (<.001 ) | -0.88 (<.001 ) | -0.80 (<.001 ) | -0.80 (<.001 ) | -1.15 (<.001 ) | -1.35 (<.001 ) | -1.09 (<.001 ) | -1.18 (<.001 ) |
| Yunnan | 2000 | 20 | 21 | 19 | 21 | 21 | 21 | 28 | 26 | 26 | 26 |
|  | 2019 | 9 | 10 | 9 | 10 | 10 | 10 | 12 | 11 | 12 | 11 |
|  | AAPC | -4.18 (<.001 ) | -3.94 (<.001 ) | -4.20 (<.001 ) | -3.92 (<.001 ) | -3.85 (<.001 ) | -3.85 (<.001 ) | -4.19 (<.001 ) | -4.38 (<.001 ) | -4.13 (<.001 ) | -4.22 (<.001 ) |
| Zhejiang | 2000 | 6 | 7 | 6 | 7 | 7 | 7 | 9 | 8 | 8 | 8 |
|  | 2019 | 3 | 3 | 3 | 3 | 3 | 3 | 4 | 4 | 4 | 4 |
|  | AAPC | -4.11 (<.001 ) | -3.87 (<.001 ) | -4.13 (<.001 ) | -3.85 (<.001 ) | -3.77 (<.001 ) | -3.77 (<.001 ) | -4.20 (<.001 ) | -4.48 (<.001 ) | -4.17 (<.001 ) | -4.22 (<.001 ) |
| Hong Kong | 2000 | 3 | 3 | 3 | 3 | 3 | 3 | 5 | 4 | 4 | 4 |
|  | 2019 | 3 | 3 | 3 | 3 | 3 | 3 | 4 | 3 | 4 | 3 |
|  | AAPC | -1.10 (<.001 ) | -0.87 (<.001 ) | -1.12 (<.001 ) | -0.84 (<.001 ) | -0.76 (<.001 ) | -0.76 (<.001 ) | -1.12 (<.001 ) | -1.32 (<.001 ) | -1.06 (<.001 ) | -1.15 (<.001 ) |
| Macao | 2000 | 9 | 10 | 9 | 10 | 10 | 10 | 12 | 12 | 12 | 12 |
|  | 2019 | 7 | 8 | 7 | 8 | 8 | 8 | 10 | 9 | 10 | 9 |
|  | AAPC | -0.99 (<.001 ) | -0.75 (<.001 ) | -1.01 (<.001 ) | -0.75 (<.001 ) | -0.67 (<.001 ) | -0.67 (<.001 ) | -1.01 (<.001 ) | -1.29 (<.001 ) | -0.94 (<.001 ) | -1.04 (<.001 ) |
|  |  | **Wasting** | | | | | | | | | |
| Chinesse Mainland | 2000 | 15 | 17 | 7 | 7 | 6 | 6 | 3 | 3 | 3 | 4 |
|  | 2019 | 10 | 12 | 6 | 6 | 5 | 5 | 3 | 3 | 3 | 3 |
|  | AAPC | -2.14 (<.001 ) | -1.77 (<.001 ) | -0.45 (<.001 ) | -0.46 (<.001 ) | -0.48 (<.001 ) | -0.48 (<.001 ) | -0.59 (<.001 ) | -0.54 (<.001 ) | -0.59 (<.001 ) | -0.56 (<.001 ) |
| China |  |  |  |  |  |  |  |  |  |  |  |
| Anhui | 2000 | 15 | 16 | 6 | 7 | 6 | 6 | 3 | 3 | 3 | 4 |
|  | 2019 | 10 | 12 | 6 | 6 | 5 | 5 | 3 | 3 | 3 | 3 |
|  | AAPC | -1.74 (<.001 ) | -1.37 (<.001 ) | -0.39 (<.001 ) | -0.40 (<.001 ) | -0.42 (<.001 ) | -0.42 (<.001 ) | -0.52 (<.001 ) | -0.48 (<.001 ) | -0.53 (<.001 ) | -0.49 (<.001 ) |
| Beijing | 2000 | 7 | 8 | 6 | 6 | 5 | 5 | 2 | 2 | 3 | 3 |
|  | 2019 | 7 | 8 | 6 | 6 | 5 | 5 | 2 | 2 | 3 | 3 |
|  | AAPC | -0.65 (<.001 ) | -0.26 (<.001 ) | -0.14 (0.011 ) | -0.18 (0.015 ) | -0.17 (0.097 ) | -0.17 (0.097 ) | -0.30 (<.001 ) | -0.26 (<.001 ) | -0.31 (<.001 ) | -0.27 (<.001 ) |
| Chongqing | 2000 | 14 | 16 | 6 | 7 | 6 | 6 | 3 | 3 | 3 | 4 |
|  | 2019 | 9 | 11 | 6 | 6 | 5 | 5 | 2 | 3 | 3 | 3 |
|  | AAPC | -2.06 (<.001 ) | -1.69 (<.001 ) | -0.46 (<.001 ) | -0.47 (<.001 ) | -0.49 (<.001 ) | -0.49 (<.001 ) | -0.60 (<.001 ) | -0.55 (<.001 ) | -0.60 (<.001 ) | -0.56 (<.001 ) |
| Fujian | 2000 | 12 | 13 | 6 | 7 | 6 | 6 | 3 | 3 | 3 | 3 |
|  | 2019 | 8 | 10 | 6 | 6 | 5 | 5 | 2 | 2 | 3 | 3 |
|  | AAPC | -1.80 (<.001 ) | -1.43 (<.001 ) | -0.43 (<.001 ) | -0.44 (<.001 ) | -0.46 (<.001 ) | -0.46 (<.001 ) | -0.57 (<.001 ) | -0.52 (<.001 ) | -0.59 (<.001 ) | -0.55 (<.001 ) |
| Gansu | 2000 | 18 | 20 | 7 | 7 | 6 | 6 | 3 | 3 | 4 | 4 |
|  | 2019 | 12 | 15 | 6 | 7 | 6 | 6 | 3 | 3 | 3 | 3 |
|  | AAPC | -1.86 (<.001 ) | -1.49 (<.001 ) | -0.38 (<.001 ) | -0.40 (<.001 ) | -0.41 (<.001 ) | -0.41 (<.001 ) | -0.52 (<.001 ) | -0.47 (<.001 ) | -0.52 (<.001 ) | -0.49 (<.001 ) |
| Guangdong | 2000 | 11 | 13 | 6 | 7 | 5 | 5 | 3 | 3 | 3 | 3 |
|  | 2019 | 8 | 10 | 6 | 6 | 5 | 5 | 2 | 2 | 3 | 3 |
|  | AAPC | -1.74 (<.001 ) | -1.37 (<.001 ) | -0.26 (<.001 ) | -0.27 (<.001 ) | -0.29 (<.001 ) | -0.29 (<.001 ) | -0.40 (<.001 ) | -0.35 (<.001 ) | -0.41 (<.001 ) | -0.36 (<.001 ) |
| Guangxi | 2000 | 21 | 24 | 11 | 12 | 10 | 10 | 5 | 5 | 6 | 6 |
|  | 2019 | 15 | 18 | 10 | 11 | 9 | 9 | 4 | 4 | 5 | 5 |
|  | AAPC | -1.94 (<.001 ) | -1.56 (<.001 ) | -0.51 (<.001 ) | -0.52 (<.001 ) | -0.54 (<.001 ) | -0.54 (<.001 ) | -0.65 (<.001 ) | -0.60 (<.001 ) | -0.65 (<.001 ) | -0.61 (<.001 ) |
| Guizhou | 2000 | 31 | 35 | 8 | 9 | 8 | 8 | 4 | 4 | 4 | 5 |
|  | 2019 | 23 | 27 | 8 | 8 | 7 | 7 | 3 | 3 | 4 | 4 |
|  | AAPC | -1.76 (<.001 ) | -1.38 (<.001 ) | -0.53 (<.001 ) | -0.55 (<.001 ) | -0.57 (<.001 ) | -0.57 (<.001 ) | -0.67 (<.001 ) | -0.62 (<.001 ) | -0.67 (<.001 ) | -0.63 (<.001 ) |
| Hainan | 2000 | 14 | 16 | 6 | 7 | 6 | 6 | 3 | 3 | 3 | 3 |
|  | 2019 | 10 | 12 | 6 | 6 | 5 | 5 | 3 | 3 | 3 | 3 |
|  | AAPC | -1.69 (<.001 ) | -1.32 (<.001 ) | -0.30 (<.001 ) | -0.31 (<.001 ) | -0.33 (<.001 ) | -0.33 (<.001 ) | -0.43 (<.001 ) | -0.39 (<.001 ) | -0.46 (<.001 ) | -0.39 (<.001 ) |
| Hebei | 2000 | 13 | 14 | 6 | 7 | 6 | 6 | 3 | 3 | 3 | 3 |
|  | 2019 | 10 | 12 | 6 | 6 | 5 | 5 | 2 | 3 | 3 | 3 |
|  | AAPC | -1.45 (<.001 ) | -1.07 (<.001 ) | -0.43 (<.001 ) | -0.44 (<.001 ) | -0.46 (<.001 ) | -0.46 (<.001 ) | -0.57 (<.001 ) | -0.52 (<.001 ) | -0.59 (<.001 ) | -0.53 (<.001 ) |
| Heilongjiang | 2000 | 12 | 13 | 6 | 6 | 5 | 5 | 2 | 2 | 3 | 3 |
|  | 2019 | 6 | 7 | 5 | 6 | 5 | 5 | 2 | 2 | 3 | 3 |
|  | AAPC | -3.66 (<.001 ) | -3.32 (<.001 ) | -0.21 (<.001 ) | -0.23 (<.001 ) | -0.25 (<.001 ) | -0.25 (<.001 ) | -0.36 (<.001 ) | -0.31 (<.001 ) | -0.37 (<.001 ) | -0.32 (<.001 ) |
| Henan | 2000 | 19 | 21 | 6 | 6 | 5 | 5 | 2 | 2 | 3 | 3 |
|  | 2019 | 14 | 16 | 5 | 6 | 5 | 5 | 2 | 2 | 3 | 3 |
|  | AAPC | -1.67 (<.001 ) | -1.30 (<.001 ) | -0.46 (<.001 ) | -0.48 (<.001 ) | -0.49 (<.001 ) | -0.49 (<.001 ) | -0.60 (<.001 ) | -0.55 (<.001 ) | -0.60 (<.001 ) | -0.57 (<.001 ) |
| Hubei | 2000 | 17 | 19 | 6 | 6 | 5 | 5 | 3 | 3 | 3 | 3 |
|  | 2019 | 13 | 15 | 5 | 6 | 5 | 5 | 2 | 2 | 3 | 3 |
|  | AAPC | -1.55 (<.001 ) | -1.17 (<.001 ) | -0.55 (<.001 ) | -0.57 (<.001 ) | -0.59 (<.001 ) | -0.59 (<.001 ) | -0.69 (<.001 ) | -0.64 (<.001 ) | -0.69 (<.001 ) | -0.66 (<.001 ) |
| Hunan | 2000 | 22 | 25 | 9 | 9 | 8 | 8 | 4 | 4 | 5 | 5 |
|  | 2019 | 17 | 21 | 8 | 9 | 7 | 7 | 3 | 3 | 4 | 4 |
|  | AAPC | -1.30 (<.001 ) | -0.92 (<.001 ) | -0.43 (<.001 ) | -0.44 (<.001 ) | -0.46 (<.001 ) | -0.46 (<.001 ) | -0.56 (<.001 ) | -0.52 (<.001 ) | -0.57 (<.001 ) | -0.54 (<.001 ) |
| Jiangsu | 2000 | 11 | 12 | 5 | 6 | 5 | 5 | 2 | 2 | 3 | 3 |
|  | 2019 | 8 | 9 | 5 | 5 | 4 | 4 | 2 | 2 | 3 | 3 |
|  | AAPC | -1.78 (<.001 ) | -1.41 (<.001 ) | -0.52 (<.001 ) | -0.53 (<.001 ) | -0.55 (<.001 ) | -0.55 (<.001 ) | -0.66 (<.001 ) | -0.61 (<.001 ) | -0.66 (<.001 ) | -0.62 (<.001 ) |
| Jiangxi | 2000 | 17 | 18 | 7 | 7 | 6 | 6 | 3 | 3 | 4 | 4 |
|  | 2019 | 11 | 13 | 6 | 6 | 5 | 5 | 3 | 3 | 3 | 3 |
|  | AAPC | -2.17 (<.001 ) | -1.80 (<.001 ) | -0.60 (<.001 ) | -0.61 (<.001 ) | -0.63 (<.001 ) | -0.63 (<.001 ) | -0.73 (<.001 ) | -0.69 (<.001 ) | -0.73 (<.001 ) | -0.70 (<.001 ) |
| Jilin | 2000 | 12 | 13 | 6 | 7 | 6 | 6 | 3 | 3 | 3 | 3 |
|  | 2019 | 9 | 11 | 6 | 6 | 5 | 5 | 2 | 2 | 3 | 3 |
|  | AAPC | -1.60 (<.001 ) | -1.18 (<.001 ) | -0.42 (<.001 ) | -0.43 (<.001 ) | -0.45 (<.001 ) | -0.45 (<.001 ) | -0.56 (<.001 ) | -0.51 (<.001 ) | -0.57 (<.001 ) | -0.52 (<.001 ) |
| Liaoning | 2000 | 8 | 9 | 8 | 9 | 7 | 7 | 4 | 4 | 4 | 5 |
|  | 2019 | 6 | 7 | 8 | 8 | 7 | 7 | 3 | 3 | 4 | 4 |
|  | AAPC | -1.65 (<.001 ) | -1.27 (<.001 ) | -0.28 (<.001 ) | -0.29 (<.001 ) | -0.31 (<.001 ) | -0.31 (<.001 ) | -0.42 (<.001 ) | -0.37 (<.001 ) | -0.43 (<.001 ) | -0.38 (<.001 ) |
| Inner Mongolia | 2000 | 11 | 13 | 5 | 5 | 4 | 4 | 2 | 2 | 2 | 3 |
|  | 2019 | 5 | 6 | 4 | 5 | 4 | 4 | 2 | 2 | 2 | 2 |
|  | AAPC | -4.55 (<.001 ) | -4.20 (<.001 ) | -0.51 (<.001 ) | -0.53 (<.001 ) | -0.54 (<.001 ) | -0.54 (<.001 ) | -0.65 (<.001 ) | -0.60 (<.001 ) | -0.67 (<.001 ) | -0.62 (<.001 ) |
| Ningxia | 2000 | 11 | 12 | 5 | 6 | 5 | 5 | 2 | 2 | 3 | 3 |
|  | 2019 | 4 | 5 | 5 | 5 | 4 | 4 | 2 | 2 | 3 | 3 |
|  | AAPC | -4.76 (<.001 ) | -4.41 (<.001 ) | -0.46 (<.001 ) | -0.47 (<.001 ) | -0.49 (<.001 ) | -0.49 (<.001 ) | -0.59 (<.001 ) | -0.55 (<.001 ) | -0.61 (<.001 ) | -0.55 (<.001 ) |
| Qinghai | 2000 | 18 | 20 | 7 | 7 | 6 | 6 | 3 | 3 | 4 | 4 |
|  | 2019 | 13 | 16 | 6 | 7 | 6 | 6 | 3 | 3 | 3 | 3 |
|  | AAPC | -1.79 (<.001 ) | -1.42 (<.001 ) | -0.46 (<.001 ) | -0.48 (<.001 ) | -0.49 (<.001 ) | -0.49 (<.001 ) | -0.60 (<.001 ) | -0.55 (<.001 ) | -0.61 (<.001 ) | -0.57 (<.001 ) |
| Shaanxi | 2000 | 14 | 15 | 6 | 7 | 6 | 6 | 3 | 3 | 3 | 3 |
|  | 2019 | 9 | 11 | 6 | 6 | 5 | 5 | 2 | 3 | 3 | 3 |
|  | AAPC | -2.00 (<.001 ) | -1.63 (<.001 ) | -0.40 (<.001 ) | -0.42 (<.001 ) | -0.43 (<.001 ) | -0.43 (<.001 ) | -0.54 (<.001 ) | -0.49 (<.001 ) | -0.55 (<.001 ) | -0.51 (<.001 ) |
| Shandong | 2000 | 12 | 13 | 4 | 5 | 4 | 4 | 2 | 2 | 2 | 2 |
|  | 2019 | 7 | 8 | 4 | 4 | 4 | 4 | 2 | 2 | 2 | 2 |
|  | AAPC | -2.72 (<.001 ) | -2.35 (<.001 ) | -0.48 (<.001 ) | -0.49 (<.001 ) | -0.51 (<.001 ) | -0.51 (<.001 ) | -0.62 (<.001 ) | -0.57 (<.001 ) | -0.63 (<.001 ) | -0.58 (<.001 ) |
| Shanghai | 2000 | 8 | 9 | 6 | 6 | 5 | 5 | 3 | 3 | 3 | 3 |
|  | 2019 | 8 | 9 | 6 | 6 | 5 | 5 | 2 | 2 | 3 | 3 |
|  | AAPC | -0.46 (<.001 ) | -0.08 (0.247 ) | -0.18 (0.013 ) | -0.19 (0.197 ) | -0.21 (0.067 ) | -0.21 (0.067 ) | -0.31 (0.005 ) | -0.27 (0.020 ) | -0.34 (<.001 ) | -0.30 (<.001 ) |
| Shanxi | 2000 | 14 | 15 | 6 | 7 | 6 | 6 | 3 | 3 | 3 | 3 |
|  | 2019 | 10 | 12 | 6 | 6 | 5 | 5 | 3 | 3 | 3 | 3 |
|  | AAPC | -1.85 (<.001 ) | -1.48 (<.001 ) | -0.31 (<.001 ) | -0.32 (<.001 ) | -0.34 (<.001 ) | -0.34 (<.001 ) | -0.45 (<.001 ) | -0.40 (<.001 ) | -0.46 (<.001 ) | -0.41 (<.001 ) |
| Sichuan | 2000 | 18 | 19 | 6 | 7 | 6 | 6 | 3 | 3 | 3 | 4 |
|  | 2019 | 7 | 9 | 6 | 6 | 5 | 5 | 3 | 3 | 3 | 3 |
|  | AAPC | -4.57 (<.001 ) | -4.12 (<.001 ) | -0.48 (<.001 ) | -0.49 (<.001 ) | -0.51 (<.001 ) | -0.51 (<.001 ) | -0.61 (<.001 ) | -0.57 (<.001 ) | -0.62 (<.001 ) | -0.58 (<.001 ) |
| Tianjin | 2000 | 9 | 10 | 6 | 6 | 5 | 5 | 3 | 3 | 3 | 3 |
|  | 2019 | 7 | 8 | 6 | 6 | 5 | 5 | 2 | 2 | 3 | 3 |
|  | AAPC | -1.35 (<.001 ) | -0.97 (<.001 ) | -0.30 (<.001 ) | -0.31 (<.001 ) | -0.33 (<.001 ) | -0.33 (<.001 ) | -0.44 (<.001 ) | -0.39 (<.001 ) | -0.44 (<.001 ) | -0.40 (<.001 ) |
| Xinjiang | 2000 | 16 | 18 | 7 | 7 | 6 | 6 | 3 | 3 | 3 | 4 |
|  | 2019 | 12 | 14 | 6 | 7 | 5 | 5 | 3 | 3 | 3 | 3 |
|  | AAPC | -1.71 (<.001 ) | -1.34 (<.001 ) | -0.37 (<.001 ) | -0.39 (<.001 ) | -0.40 (<.001 ) | -0.40 (<.001 ) | -0.51 (<.001 ) | -0.46 (<.001 ) | -0.54 (<.001 ) | -0.49 (<.001 ) |
| Xizang | 2000 | 22 | 24 | 7 | 8 | 6 | 6 | 3 | 3 | 4 | 4 |
|  | 2019 | 19 | 23 | 7 | 7 | 6 | 6 | 3 | 3 | 4 | 4 |
|  | AAPC | -0.80 (<.001 ) | -0.41 (<.001 ) | -0.25 (<.001 ) | -0.26 (<.001 ) | -0.28 (<.001 ) | -0.28 (<.001 ) | -0.39 (<.001 ) | -0.34 (<.001 ) | -0.39 (<.001 ) | -0.35 (<.001 ) |
| Yunnan | 2000 | 21 | 24 | 9 | 9 | 8 | 8 | 4 | 4 | 5 | 5 |
|  | 2019 | 10 | 12 | 8 | 8 | 7 | 7 | 3 | 3 | 4 | 4 |
|  | AAPC | -3.85 (<.001 ) | -3.48 (<.001 ) | -0.52 (<.001 ) | -0.53 (<.001 ) | -0.55 (<.001 ) | -0.55 (<.001 ) | -0.65 (<.001 ) | -0.61 (<.001 ) | -0.66 (<.001 ) | -0.62 (<.001 ) |
| Zhejiang | 2000 | 7 | 8 | 5 | 6 | 5 | 5 | 2 | 2 | 3 | 3 |
|  | 2019 | 3 | 4 | 5 | 6 | 5 | 5 | 2 | 2 | 3 | 3 |
|  | AAPC | -3.77 (<.001 ) | -3.48 (<.001 ) | -0.31 (<.001 ) | -0.31 (<.001 ) | -0.33 (<.001 ) | -0.33 (<.001 ) | -0.44 (<.001 ) | -0.39 (<.001 ) | -0.45 (<.001 ) | -0.40 (<.001 ) |
| Hong Kong | 2000 | 3 | 4 | 3 | 4 | 3 | 3 | 1 | 1 | 2 | 2 |
|  | 2019 | 3 | 4 | 3 | 4 | 3 | 3 | 1 | 1 | 2 | 2 |
|  | AAPC | -0.76 (<.001 ) | -0.38 (<.001 ) | 0.09 (0.651 ) | 0.08 (0.697 ) | 0.06 (0.758 ) | 0.06 (0.758 ) | -0.04 (0.820 ) | 0.00 (0.994 ) | -0.07 (0.559 ) | -0.03 (0.806 ) |
| Macao | 2000 | 10 | 11 | 6 | 6 | 5 | 5 | 3 | 3 | 3 | 3 |
|  | 2019 | 8 | 10 | 6 | 6 | 5 | 5 | 3 | 3 | 3 | 3 |
|  | AAPC | -0.67 (<.001 ) | -0.32 (<.001 ) | -0.07 (0.103 ) | -0.08 (0.053 ) | -0.10 (0.016 ) | -0.10 (0.016 ) | -0.21 (<.001 ) | -0.16 (<.001 ) | -0.20 (<.001 ) | -0.16 (<.001 ) |
|  |  | **Underweight** | | | | | | | | | |
| Chinesse Mainland | 2000 | 14 | 15 | 8 | 8 | 8 | 8 | 8 | 7 | 8 | 7 |
|  | 2019 | 9 | 10 | 4 | 5 | 4 | 4 | 4 | 4 | 4 | 4 |
|  | AAPC | -2.49 (<.001 ) | -2.22 (<.001 ) | -2.99 (<.001 ) | -2.57 (<.001 ) | -2.80 (<.001 ) | -2.80 (<.001 ) | -2.91 (<.001 ) | -3.19 (<.001 ) | -2.90 (<.001 ) | -3.01 (<.001 ) |
| China |  |  |  |  |  |  |  |  |  |  |  |
| Anhui | 2000 | 13 | 14 | 8 | 8 | 8 | 8 | 7 | 7 | 7 | 7 |
|  | 2019 | 9 | 10 | 5 | 5 | 5 | 5 | 5 | 4 | 5 | 4 |
|  | AAPC | -2.10 (<.001 ) | -1.83 (<.001 ) | -2.56 (<.001 ) | -2.14 (<.001 ) | -2.37 (<.001 ) | -2.37 (<.001 ) | -2.48 (<.001 ) | -2.76 (<.001 ) | -2.47 (<.001 ) | -2.58 (<.001 ) |
| Beijing | 2000 | 7 | 7 | 4 | 4 | 4 | 4 | 4 | 3 | 4 | 3 |
|  | 2019 | 6 | 6 | 3 | 3 | 3 | 3 | 3 | 2 | 3 | 3 |
|  | AAPC | -1.01 (<.001 ) | -0.73 (<.001 ) | -1.33 (<.001 ) | -0.91 (<.001 ) | -1.14 (<.001 ) | -1.14 (<.001 ) | -1.25 (<.001 ) | -1.59 (<.001 ) | -1.24 (<.001 ) | -1.36 (<.001 ) |
| Chongqing | 2000 | 13 | 14 | 7 | 7 | 7 | 7 | 7 | 6 | 7 | 7 |
|  | 2019 | 8 | 9 | 4 | 5 | 4 | 4 | 4 | 3 | 4 | 4 |
|  | AAPC | -2.42 (<.001 ) | -2.15 (<.001 ) | -2.91 (<.001 ) | -2.50 (<.001 ) | -2.73 (<.001 ) | -2.73 (<.001 ) | -2.84 (<.001 ) | -3.12 (<.001 ) | -2.82 (<.001 ) | -2.93 (<.001 ) |
| Fujian | 2000 | 11 | 12 | 6 | 6 | 6 | 6 | 6 | 5 | 6 | 6 |
|  | 2019 | 7 | 8 | 4 | 4 | 4 | 4 | 4 | 3 | 4 | 3 |
|  | AAPC | -2.16 (<.001 ) | -1.89 (<.001 ) | -2.62 (<.001 ) | -2.19 (<.001 ) | -2.43 (<.001 ) | -2.43 (<.001 ) | -2.53 (<.001 ) | -2.84 (<.001 ) | -2.52 (<.001 ) | -2.65 (<.001 ) |
| Gansu | 2000 | 16 | 18 | 10 | 10 | 9 | 9 | 9 | 8 | 9 | 9 |
|  | 2019 | 11 | 12 | 6 | 6 | 6 | 6 | 5 | 5 | 5 | 5 |
|  | AAPC | -2.21 (<.001 ) | -1.94 (<.001 ) | -2.87 (<.001 ) | -2.45 (<.001 ) | -2.69 (<.001 ) | -2.69 (<.001 ) | -2.79 (<.001 ) | -3.07 (<.001 ) | -2.78 (<.001 ) | -2.88 (<.001 ) |
| Guangdong | 2000 | 10 | 11 | 6 | 6 | 6 | 6 | 6 | 5 | 6 | 5 |
|  | 2019 | 7 | 8 | 4 | 4 | 4 | 4 | 4 | 3 | 4 | 3 |
|  | AAPC | -2.10 (<.001 ) | -1.82 (<.001 ) | -2.52 (<.001 ) | -2.10 (<.001 ) | -2.33 (<.001 ) | -2.33 (<.001 ) | -2.43 (<.001 ) | -2.71 (<.001 ) | -2.42 (<.001 ) | -2.53 (<.001 ) |
| Guangxi | 2000 | 19 | 21 | 12 | 12 | 12 | 12 | 11 | 10 | 12 | 11 |
|  | 2019 | 12 | 14 | 8 | 9 | 8 | 8 | 8 | 7 | 8 | 7 |
|  | AAPC | -2.29 (<.001 ) | -2.02 (<.001 ) | -2.04 (<.001 ) | -1.62 (<.001 ) | -1.85 (<.001 ) | -1.85 (<.001 ) | -1.96 (<.001 ) | -2.36 (<.001 ) | -1.95 (<.001 ) | -2.13 (<.001 ) |
| Guizhou | 2000 | 29 | 31 | 18 | 18 | 18 | 18 | 18 | 16 | 18 | 16 |
|  | 2019 | 19 | 22 | 10 | 11 | 10 | 10 | 10 | 8 | 10 | 9 |
|  | AAPC | -2.12 (<.001 ) | -1.84 (<.001 ) | -3.14 (<.001 ) | -2.73 (<.001 ) | -2.96 (<.001 ) | -2.96 (<.001 ) | -3.07 (<.001 ) | -3.34 (<.001 ) | -3.05 (<.001 ) | -3.16 (<.001 ) |
| Hainan | 2000 | 13 | 14 | 7 | 8 | 7 | 7 | 7 | 6 | 7 | 7 |
|  | 2019 | 9 | 10 | 5 | 5 | 5 | 5 | 4 | 4 | 5 | 4 |
|  | AAPC | -2.05 (<.001 ) | -1.78 (<.001 ) | -2.53 (<.001 ) | -2.12 (<.001 ) | -2.35 (<.001 ) | -2.35 (<.001 ) | -2.46 (<.001 ) | -2.74 (<.001 ) | -2.44 (<.001 ) | -2.56 (<.001 ) |
| Hebei | 2000 | 12 | 13 | 7 | 7 | 7 | 7 | 6 | 6 | 6 | 6 |
|  | 2019 | 8 | 9 | 4 | 5 | 4 | 4 | 4 | 4 | 4 | 4 |
|  | AAPC | -1.80 (<.001 ) | -1.53 (<.001 ) | -2.29 (<.001 ) | -1.88 (<.001 ) | -2.11 (<.001 ) | -2.11 (<.001 ) | -2.21 (<.001 ) | -2.50 (<.001 ) | -2.20 (<.001 ) | -2.31 (<.001 ) |
| Heilongjiang | 2000 | 11 | 12 | 5 | 5 | 5 | 5 | 5 | 4 | 5 | 5 |
|  | 2019 | 5 | 6 | 3 | 4 | 3 | 3 | 3 | 3 | 3 | 3 |
|  | AAPC | -3.98 (<.001 ) | -3.77 (<.001 ) | -2.25 (<.001 ) | -1.84 (<.001 ) | -2.10 (<.001 ) | -2.10 (<.001 ) | -2.21 (<.001 ) | -2.45 (<.001 ) | -2.19 (<.001 ) | -2.29 (<.001 ) |
| Henan | 2000 | 17 | 19 | 9 | 9 | 9 | 9 | 8 | 8 | 8 | 8 |
|  | 2019 | 12 | 13 | 5 | 6 | 5 | 5 | 5 | 4 | 5 | 5 |
|  | AAPC | -2.03 (<.001 ) | -1.76 (<.001 ) | -2.65 (<.001 ) | -2.23 (<.001 ) | -2.47 (<.001 ) | -2.47 (<.001 ) | -2.57 (<.001 ) | -2.84 (<.001 ) | -2.56 (<.001 ) | -2.65 (<.001 ) |
| Hubei | 2000 | 15 | 17 | 8 | 8 | 7 | 7 | 7 | 7 | 7 | 7 |
|  | 2019 | 11 | 12 | 4 | 5 | 4 | 4 | 4 | 4 | 4 | 4 |
|  | AAPC | -1.90 (<.001 ) | -1.63 (<.001 ) | -2.94 (<.001 ) | -2.53 (<.001 ) | -2.76 (<.001 ) | -2.76 (<.001 ) | -2.87 (<.001 ) | -3.16 (<.001 ) | -2.85 (<.001 ) | -2.98 (<.001 ) |
| Hunan | 2000 | 20 | 22 | 10 | 10 | 9 | 9 | 9 | 8 | 9 | 9 |
|  | 2019 | 15 | 17 | 6 | 7 | 6 | 6 | 6 | 5 | 6 | 6 |
|  | AAPC | -1.66 (<.001 ) | -1.38 (<.001 ) | -2.27 (<.001 ) | -1.85 (<.001 ) | -2.08 (<.001 ) | -2.08 (<.001 ) | -2.19 (<.001 ) | -2.47 (<.001 ) | -2.17 (<.001 ) | -2.28 (<.001 ) |
| Jiangsu | 2000 | 10 | 11 | 6 | 6 | 6 | 6 | 6 | 5 | 6 | 5 |
|  | 2019 | 7 | 8 | 4 | 4 | 4 | 4 | 4 | 3 | 4 | 3 |
|  | AAPC | -2.14 (<.001 ) | -1.87 (<.001 ) | -2.41 (<.001 ) | -1.99 (<.001 ) | -2.22 (<.001 ) | -2.22 (<.001 ) | -2.33 (<.001 ) | -2.60 (<.001 ) | -2.31 (<.001 ) | -2.42 (<.001 ) |
| Jiangxi | 2000 | 15 | 17 | 9 | 9 | 9 | 9 | 9 | 8 | 9 | 8 |
|  | 2019 | 9 | 11 | 5 | 5 | 5 | 5 | 5 | 4 | 5 | 4 |
|  | AAPC | -2.53 (<.001 ) | -2.26 (<.001 ) | -3.17 (<.001 ) | -2.75 (<.001 ) | -2.99 (<.001 ) | -2.99 (<.001 ) | -3.09 (<.001 ) | -3.36 (<.001 ) | -3.07 (<.001 ) | -3.18 (<.001 ) |
| Jilin | 2000 | 11 | 12 | 6 | 6 | 6 | 6 | 6 | 5 | 6 | 6 |
|  | 2019 | 8 | 9 | 4 | 4 | 4 | 4 | 4 | 3 | 4 | 3 |
|  | AAPC | -1.91 (<.001 ) | -1.64 (<.001 ) | -2.41 (<.001 ) | -1.97 (<.001 ) | -2.22 (<.001 ) | -2.22 (<.001 ) | -2.33 (<.001 ) | -2.63 (<.001 ) | -2.32 (<.001 ) | -2.43 (<.001 ) |
| Liaoning | 2000 | 8 | 8 | 5 | 5 | 5 | 5 | 5 | 4 | 5 | 4 |
|  | 2019 | 5 | 6 | 3 | 3 | 3 | 3 | 3 | 2 | 3 | 3 |
|  | AAPC | -2.02 (<.001 ) | -1.74 (<.001 ) | -2.46 (<.001 ) | -2.04 (<.001 ) | -2.28 (<.001 ) | -2.28 (<.001 ) | -2.38 (<.001 ) | -2.68 (<.001 ) | -2.38 (<.001 ) | -2.50 (<.001 ) |
| Inner Mongolia | 2000 | 10 | 11 | 5 | 5 | 5 | 5 | 5 | 4 | 5 | 4 |
|  | 2019 | 4 | 5 | 2 | 2 | 2 | 2 | 2 | 2 | 2 | 2 |
|  | AAPC | -4.98 (<.001 ) | -4.64 (<.001 ) | -4.36 (<.001 ) | -3.94 (<.001 ) | -4.15 (<.001 ) | -4.15 (<.001 ) | -4.25 (<.001 ) | -4.61 (<.001 ) | -4.24 (<.001 ) | -4.43 (<.001 ) |
| Ningxia | 2000 | 10 | 11 | 5 | 5 | 5 | 5 | 5 | 4 | 5 | 5 |
|  | 2019 | 4 | 4 | 2 | 2 | 2 | 2 | 2 | 2 | 2 | 2 |
|  | AAPC | -5.18 (<.001 ) | -4.86 (<.001 ) | -5.21 (<.001 ) | -4.77 (<.001 ) | -5.02 (<.001 ) | -5.02 (<.001 ) | -5.11 (<.001 ) | -5.38 (<.001 ) | -5.10 (<.001 ) | -5.25 (<.001 ) |
| Qinghai | 2000 | 17 | 18 | 10 | 10 | 10 | 10 | 10 | 9 | 10 | 9 |
|  | 2019 | 11 | 13 | 6 | 6 | 6 | 6 | 6 | 5 | 6 | 5 |
|  | AAPC | -2.15 (<.001 ) | -1.88 (<.001 ) | -2.93 (<.001 ) | -2.51 (<.001 ) | -2.75 (<.001 ) | -2.75 (<.001 ) | -2.85 (<.001 ) | -3.12 (<.001 ) | -2.84 (<.001 ) | -2.93 (<.001 ) |
| Shaanxi | 2000 | 12 | 14 | 7 | 7 | 7 | 7 | 7 | 6 | 7 | 6 |
|  | 2019 | 8 | 9 | 4 | 4 | 4 | 4 | 4 | 3 | 4 | 4 |
|  | AAPC | -2.35 (<.001 ) | -2.08 (<.001 ) | -2.89 (<.001 ) | -2.47 (<.001 ) | -2.71 (<.001 ) | -2.71 (<.001 ) | -2.80 (<.001 ) | -3.08 (<.001 ) | -2.80 (<.001 ) | -2.90 (<.001 ) |
| Shandong | 2000 | 11 | 12 | 3 | 3 | 3 | 3 | 3 | 3 | 3 | 3 |
|  | 2019 | 6 | 7 | 2 | 2 | 2 | 2 | 2 | 2 | 2 | 2 |
|  | AAPC | -3.07 (<.001 ) | -2.80 (<.001 ) | -2.46 (<.001 ) | -2.04 (<.001 ) | -2.27 (<.001 ) | -2.27 (<.001 ) | -2.38 (<.001 ) | -2.68 (<.001 ) | -2.37 (<.001 ) | -2.49 (<.001 ) |
| Shanghai | 2000 | 8 | 8 | 4 | 4 | 4 | 4 | 4 | 4 | 4 | 4 |
|  | 2019 | 6 | 7 | 3 | 4 | 3 | 3 | 3 | 3 | 3 | 3 |
|  | AAPC | -0.82 (<.001 ) | -0.54 (<.001 ) | -1.18 (<.001 ) | -0.71 (<.001 ) | -1.00 (<.001 ) | -1.00 (<.001 ) | -1.10 (<.001 ) | -1.35 (<.001 ) | -1.09 (<.001 ) | -1.18 (<.001 ) |
| Shanxi | 2000 | 13 | 14 | 7 | 7 | 7 | 7 | 7 | 6 | 7 | 6 |
|  | 2019 | 8 | 10 | 4 | 5 | 4 | 4 | 4 | 4 | 4 | 4 |
|  | AAPC | -2.21 (<.001 ) | -1.94 (<.001 ) | -2.70 (<.001 ) | -2.29 (<.001 ) | -2.52 (<.001 ) | -2.52 (<.001 ) | -2.63 (<.001 ) | -2.90 (<.001 ) | -2.58 (<.001 ) | -2.73 (<.001 ) |
| Sichuan | 2000 | 16 | 17 | 9 | 9 | 9 | 9 | 9 | 8 | 9 | 8 |
|  | 2019 | 6 | 7 | 3 | 3 | 3 | 3 | 3 | 3 | 3 | 3 |
|  | AAPC | -4.91 (<.001 ) | -4.65 (<.001 ) | -5.41 (<.001 ) | -5.01 (<.001 ) | -5.23 (<.001 ) | -5.23 (<.001 ) | -5.34 (<.001 ) | -5.59 (<.001 ) | -5.32 (<.001 ) | -5.37 (<.001 ) |
| Tianjin | 2000 | 8 | 9 | 5 | 5 | 4 | 4 | 4 | 4 | 4 | 4 |
|  | 2019 | 6 | 7 | 3 | 3 | 3 | 3 | 3 | 3 | 3 | 3 |
|  | AAPC | -1.69 (<.001 ) | -1.43 (<.001 ) | -2.10 (<.001 ) | -1.68 (<.001 ) | -1.91 (<.001 ) | -1.91 (<.001 ) | -2.02 (<.001 ) | -2.30 (<.001 ) | -2.01 (<.001 ) | -2.16 (<.001 ) |
| Xinjiang | 2000 | 15 | 16 | 9 | 9 | 9 | 9 | 8 | 8 | 9 | 8 |
|  | 2019 | 10 | 11 | 5 | 6 | 5 | 5 | 5 | 4 | 5 | 5 |
|  | AAPC | -2.07 (<.001 ) | -1.80 (<.001 ) | -2.73 (<.001 ) | -2.32 (<.001 ) | -2.55 (<.001 ) | -2.55 (<.001 ) | -2.65 (<.001 ) | -2.92 (<.001 ) | -2.64 (<.001 ) | -2.75 (<.001 ) |
| Xizang | 2000 | 20 | 22 | 13 | 13 | 13 | 13 | 13 | 11 | 13 | 12 |
|  | 2019 | 16 | 19 | 9 | 10 | 9 | 9 | 9 | 8 | 9 | 8 |
|  | AAPC | -1.15 (<.001 ) | -0.88 (<.001 ) | -1.88 (<.001 ) | -1.45 (<.001 ) | -1.69 (<.001 ) | -1.69 (<.001 ) | -1.80 (<.001 ) | -2.08 (<.001 ) | -1.79 (<.001 ) | -1.89 (<.001 ) |
| Yunnan | 2000 | 19 | 21 | 13 | 13 | 13 | 13 | 12 | 11 | 12 | 11 |
|  | 2019 | 9 | 10 | 5 | 5 | 5 | 5 | 5 | 4 | 5 | 4 |
|  | AAPC | -4.20 (<.001 ) | -3.92 (<.001 ) | -5.11 (<.001 ) | -4.70 (<.001 ) | -4.93 (<.001 ) | -4.93 (<.001 ) | -5.02 (<.001 ) | -5.28 (<.001 ) | -5.02 (<.001 ) | -5.11 (<.001 ) |
| Zhejiang | 2000 | 6 | 7 | 4 | 4 | 4 | 4 | 4 | 4 | 4 | 4 |
|  | 2019 | 3 | 3 | 2 | 2 | 2 | 2 | 2 | 2 | 2 | 2 |
|  | AAPC | -4.13 (<.001 ) | -3.85 (<.001 ) | -4.04 (<.001 ) | -3.65 (<.001 ) | -3.85 (<.001 ) | -3.85 (<.001 ) | -3.99 (<.001 ) | -4.36 (<.001 ) | -3.95 (<.001 ) | -4.16 (<.001 ) |
| Hong Kong | 2000 | 3 | 3 | 3 | 3 | 3 | 3 | 3 | 2 | 3 | 2 |
|  | 2019 | 3 | 3 | 2 | 3 | 2 | 2 | 2 | 2 | 2 | 2 |
|  | AAPC | -1.12 (<.001 ) | -0.84 (<.001 ) | -1.01 (<.001 ) | -0.56 (<.001 ) | -0.80 (<.001 ) | -0.80 (<.001 ) | -0.91 (<.001 ) | -1.18 (<.001 ) | -0.89 (<.001 ) | -1.00 (<.001 ) |
| Macao | 2000 | 9 | 10 | 5 | 5 | 5 | 5 | 5 | 4 | 5 | 4 |
|  | 2019 | 7 | 8 | 4 | 4 | 4 | 4 | 4 | 3 | 4 | 3 |
|  | AAPC | -1.01 (<.001 ) | -0.75 (<.001 ) | -1.35 (<.001 ) | -0.91 (<.001 ) | -1.16 (<.001 ) | -1.16 (<.001 ) | -1.25 (<.001 ) | -1.53 (<.001 ) | -1.24 (<.001 ) | -1.34 (<.001 ) |
