# Supplementary material for: Temporal Trends in the Prevalence of Child Undernutrition in China From 2000 to 2019, With Projections of Prevalence in 2030: Cross-Sectional Analysis
Source: JMIR Public Health Surveill. 2024 Oct 9;10:e58564. doi: 10.2196/58564 (PMC11499720; doi:10.2196/58564)
Supplement: Multimedia Appendix 7 [file publichealth_v10i1e58564_app7.docx]

**Multimedia Appendix 7.** Projections of stunting and wasting by 2025 and 2030.

|  | **Stunting** | | **Wasting** | |
| --- | --- | --- | --- | --- |
| **Projection** | **2025** | **2030** | **2025** | **2030** |
| Anhui | 9.9% | 8.3% | 3.1% | 3.0% |
| Beijing | 7.4% | 7.2% | 2.9% | 2.9% |
| Chongqing | 10.4% | 10.0% | 3.1% | 3.1% |
| Fujian | 9.2% | 8.8% | 3.0% | 3.0% |
| Gansu | 13.6% | 13.2% | 3.3% | 3.3% |
| Guangdong | 9.0% | 8.7% | 3.0% | 3.0% |
| Guangxi | 16.1% | 15.6% | 5.1% | 5.1% |
| Guizhou | 25.7% | 25.8% | 4.0% | 4.0% |
| Hainan | 11.5% | 11.2% | 3.1% | 3.1% |
| Hebei | 10.6% | 10.3% | 3.1% | 3.1% |
| Heilongjiang | 6.6% | 6.5% | 2.8% | 2.8% |
| Henan | 15.6% | 15.6% | 2.7% | 2.7% |
| Hubei | 13.6% | 13.0% | 2.8% | 2.8% |
| Hunan | 19.4% | 19.1% | 4.2% | 4.2% |
| Jiangsu | 8.6% | 8.5% | 2.6% | 2.6% |
| Jiangxi | 12.1% | 11.8% | 3.2% | 3.2% |
| Jilin | 9.7% | 9.3% | 3.0% | 3.0% |
| Liaoning | 6.9% | 6.8% | 4.1% | 4.1% |
| Inner Mongolia | 5.4% | 5.4% | 2.2% | 2.2% |
| Ningxia | 4.8% | 4.7% | 2.6% | 2.6% |
| Qinghai | 14.5% | 14.1% | 3.3% | 3.3% |
| Shaanxi | 10.4% | 10.1% | 3.1% | 3.1% |
| Shandong | 7.3% | 6.9% | 2.1% | 2.1% |
| Shanghai | 8.5% | 8.4% | 2.9% | 2.9% |
| Shanxi | 10.7% | 10.4% | 3.1% | 3.1% |
| Sichuan | 8.4% | 8.5% | 3.1% | 3.1% |
| Tianjin | 7.8% | 7.6% | 2.9% | 2.9% |
| Xinjiang | 13.0% | 12.6% | 3.2% | 3.2% |
| Xizang | 20.3% | 19.2% | 3.6% | 3.6% |
| Yunnan | 11.6% | 11.6% | 4.0% | 3.9% |
| Zhejiang | 3.7% | 3.6% | 2.7% | 2.7% |
| Hong Kong | 3.5% | 3.5% | 1.8% | 1.8% |
| Macao | 9.4% | 9.1% | 3.1% | 3.1% |
